# Supplementary material for: Sociodemographic Disparities in Adults with Kidney Failure: A Meta-Analysis
Source: Diseases. 2024 Jan 12;12(1):23. doi: 10.3390/diseases12010023 (PMC10813962; doi:10.3390/diseases12010023)
Supplement: Supplementary file 1 [file diseases-12-00023-s001.zip › diseases-2800181-supplementary.pdf]

Supplementary Materials

Contents

Supplementary S1: Search strategy 3
Supplementary S2: Excluded studies 7
Supplementary S3: Study characteristics 18
Supplementary S4: Risk of bias assessment 39
Supplementary S5: Forest plots 45
5.1. Arteriovenous fistula/graft *vs*. Central venous catheter 45
5.2. Arteriovenous fistula *vs*. Arteriovenous graft 47
5.3. Successful arteriovenous fistula use 49
5.4. Primary arteriovenous fistula patency 50
5.5. Transition from central venous catheter to arteriovenous fistula/graft 51
5.6. Home dialysis 53
5.7. Peritoneal dialysis 54
5.8. Dialysis survival 56
5.9. Waitlisting 61
5.10. Kidney transplantation 65
5.11. Living-donor transplantation 70
5.12. Deceased-donor transplantation 73
5.13. Preemptive transplantation 76
5.14. Graft survival 77
5.15. Kidney transplantation survival 80
Supplementary S6: Funnel plots 82
6.1. Arteriovenous fistula/graft *vs*. Central venous catheter 82
6.2. Arteriovenous fistula *vs*. Arteriovenous graft 84
6.3. Successful arteriovenous fistula use 86
6.4. Primary arteriovenous fistula patency 87
6.5. Transition from central venous catheter to arteriovenous fistula/graft 88
6.6. Home dialysis 90
6.7. Peritoneal dialysis 91
6.8. Dialysis survival 94
6.9. Waitlisting 98

6.10. Kidney transplantation 101
6.11. Living-donor transplantation 104
6.12. Deceased-donor transplantation 107
6.13. Preemptive transplantation 110
6.14. Graft survival 112
6.15. Kidney transplantation survival 115
Supplementary S7: Subgroup analyses 117
Supplementary S8: Sensitivity analysis 130
Supplementary S9: Summary of findings 131
Supplementary S10: PRISMA checklist 135

Supplementary S1: Search strategy

1. "Continental Population Groups"[Mesh]

2. "Ethnic Groups"[Mesh]

3. "Ethnicity"[Mesh]

4. "Racial Groups"[Mesh]

5. "Race Factors"[Mesh]

6. "Asians"[Mesh]

7. "Whites"[Mesh]

8. "Native Hawaiian or Other Pacific Islander"[Mesh]

9. "Blacks"[Mesh]

10. "Hispanic or Latino"[Mesh]

11. "Minority Groups"[Mesh]

12. (race* OR racial OR ethnic* OR white* OR black* OR asian* OR native* OR indigenous* OR hispanic* OR latin* OR minorit*) [tiab]

13. OR/1-12

14. "Gender Equity"[Mesh]

15. "Sex"[Mesh]

16. "Sexual and Gender Minorities"[Mesh]

17. (gender OR sex*) [tiab]

18. OR/15-17

19. "Socioeconomic Factors"[Mesh]

20. "Poverty"[Mesh]

21. "Income"[Mesh]

22. "Education"[Mesh]

23. "Educational Status"[Mesh]

24. "Unemployment"[Mesh]

25. (social* OR economic* OR socioeconomic OR socio-economic OR income OR education* OR occupation* OR employ* OR unemploy* OR poverty OR poor) [tiab]

26. OR/19-25

27. 13 OR 18 OR 26

28. "Health Disparity, Minority and Vulnerable Populations"[Mesh]

29. "Healthcare Disparities"[Mesh]

30. (disparit* OR inequit* OR inequality* OR discrepan* OR inconsisten*) [tiab]

31. OR/28-30

32. "Kidney Failure, Chronic"[Mesh]

33. "Renal Dialysis"[Mesh]

34. "Kidney Transplantation"[Mesh]

35. (“end-stage renal disease” OR “end-stage kidney disease” OR dialysis OR hemodialysis OR “peritoneal dialysis” OR “kidney transplant*” OR “renal transplant*” OR “arteriovenous fistula” OR “AV fistula” OR “arteriovenous graft” OR “AV graft”) [tiab]

36. OR/32-35

37. 27 AND 31 AND 36

PubMed search

("Continental Population Groups"[Mesh] OR "Ethnic Groups"[Mesh] OR "Ethnicity"[Mesh] OR "Racial Groups"[Mesh] OR "Race Factors"[Mesh] OR "Asians"[Mesh] OR "Whites"[Mesh] "Native Hawaiian or Other Pacific Islander"[Mesh] OR "Blacks"[Mesh] OR "Blacks"[Mesh] OR "Hispanic or Latino"[Mesh] OR (race* OR racial OR ethnic* OR white* OR black* OR asian* OR native* OR indigenous* OR hispanic* OR latin* OR minorit*) [tiab]) OR ("Gender Equity"[Mesh] OR "Sex"[Mesh] OR "Sexual and Gender Minorities"[Mesh] OR(gender OR sex*) [tiab]) OR ("Socioeconomic Factors"[Mesh] OR"Poverty"[Mesh] OR "Income"[Mesh] OR "Education"[Mesh] OR "Educational Status"[Mesh] OR "Unemployment"[Mesh] OR (social* OR economic* OR socioeconomic OR socio-economic OR income OR education* OR occupation* OR employ* OR unemploy* OR poverty OR poor) [tiab]) AND ("Health Disparity, Minority and Vulnerable Populations"[Mesh] OR "Healthcare Disparities"[Mesh] OR (disparit* OR inequit* OR inequality* OR discrepan* OR inconsisten*) [tiab]) AND ("Kidney Failure, Chronic"[Mesh] OR "Renal Dialysis"[Mesh] OR "Kidney Transplantation"[Mesh] OR (“end-stage renal disease” OR “end-stage kidney disease” OR dialysis OR hemodialysis OR “peritoneal dialysis” OR “kidney transplant*” OR “renal transplant*” OR “arteriovenous fistula” OR “AV fistula” OR “arteriovenous graft” OR “AV graft”) [tiab])

Result (20 Feb 2022): 3,315 records

Scopus search

(race* OR racial OR ethnic* OR white* OR black* OR asian* OR native* OR indigenous* OR hispanic* OR latin* OR minorit* OR gender OR sex OR social* OR economic* OR socioeconomic OR socio-economic OR income OR education* OR occupation* OR employ* OR unemploy* OR poverty OR poor) AND (disparit* OR inequit* OR inequality* OR discrepan* OR inconsisten*) AND (“end-stage renal disease” OR “end-stage kidney disease” OR dialysis OR hemodialysis OR “peritoneal dialysis” OR “kidney transplant*” OR “renal transplant*” OR “arteriovenous fistula” OR “AV fistula” OR “arteriovenous graft” OR “AV graft”)

Result (20 Feb 2022): 2,416 records

Web of Science search

(race* OR racial OR ethnic* OR white* OR black* OR asian* OR native* OR indigenous* OR hispanic* OR latin* OR minorit* OR gender OR sex OR social* OR economic* OR socioeconomic OR socio-economic OR income OR education* OR occupation* OR employ* OR unemploy* OR poverty OR poor) AND (disparit* OR inequit* OR inequality* OR discrepan* OR inconsisten*) AND (“end-stage renal disease” OR “end-stage kidney disease” OR dialysis OR hemodialysis OR “peritoneal dialysis” OR “kidney transplant*” OR “renal transplant*” OR “arteriovenous fistula” OR “AV fistula” OR “arteriovenous graft” OR “AV graft”)

Result (20 Feb 2022): 2,657 records

CENTRAL search

(race* OR racial OR ethnic* OR white* OR black* OR asian* OR native* OR indigenous* OR hispanic* OR latin* OR minorit* OR gender OR sex OR social* OR economic* OR socioeconomic OR socio-economic OR income OR education* OR occupation* OR employ* OR unemploy* OR poverty OR poor) AND (disparit* OR inequit* OR inequality* OR discrepan* OR inconsisten*) AND (“end-stage renal disease” OR “end-stage kidney disease” OR dialysis OR hemodialysis OR “peritoneal dialysis” OR “kidney transplant*” OR “renal transplant*” OR “arteriovenous fistula” OR “AV fistula” OR “arteriovenous graft” OR “AV graft”)

Result (20 Feb 2022): 100 records

Supplementary S2: Excluded studies

Suppl. Table S1. List of excluded studies with reasons

Study	Reason for exclusion
**2021; Garcia-Garcia**	Descriptive study
**2021; van Amstel**	Different exposure
**2021; Wong**	No outcome of interest
**2021; Ku**	Different exposure
**2021; Seipp**	No outcome of interest
**2021; Davidovits**	Different exposure
**2021; Anand**	No outcome of interest
**2021; Plumb**	Pediatric population
**2020; Grossi**	Evaluation of immigration
**2020; Dunlop**	No outcome of interest
**2020; Golestaneh**	No outcome of interest
**2020; Weigert**	No outcome of interest
**2020; Nguyen**	Different exposure
**2020; Williams**	Overlapping populations
**2020; Bailey**	No outcome of interest
**2020; Patzer**	Different exposure
**2020; Ku**	Descriptive study
**2020; Riad**	Pediatric population
**2020; Bonthuis**	Pediatric population
**2020; Amaral**	Pediatric population
**2019; Godara**	No outcome of interest
**2019; Mıhçıokur**	No outcome of interest
**2019; Mustian**	Different exposure
**2019; Pletcher**	Different exposure
**2019; Schouten**	Evaluation of immigration
**2019; Shah**	Descriptive study
**2019; Hamoda**	No outcome of interest
**2019; Vedadi**	No outcome of interest
**2019;Yan**	Different exposure
**2019; Lee**	Overlapping populations
**2019; Arvelakis**	No outcome of interest
**2019; Kim**	No outcome of interest
**2019; Ahearn**	Pediatric population
**2018; Markell**	Overlapping populations
**2018; Wilkins**	No outcome of interest
**2018; Khanal**	Overlapping populations
**2018; Gillis**	Different exposure
**2018; Gander**	Overlapping populations

**2018; Matter**	No outcome of interest
**2018; Miller**	Different exposure
**2017; Derrett**	No outcome of interest
**2017; Woo**	No outcome of interest
**2017; Laster**	Overlapping populations
**2017; Pecoits-Filho**	Overlapping populations
**2017; Kwan**	No outcome of interest
**2017; Tahir**	No outcome of interest
**2017; Wu**	Different exposure
**2017; Taber**	Overlapping populations
**2017; Ku**	Pediatric population
**2016; Nogueira**	Different exposure
**2016; Peracha**	No outcome of interest
**2016; Taber**	No outcome of interest
**2016; Ng**	No outcome of interest
**2016; Tjaden**	Pediatric population
**2016; Hogan**	Pediatric population
**2015; Zarkowsky**	Overlapping populations
**2015; Vogelzang**	No outcome of interest
**2015; Tang**	No outcome of interest
**2015; Patzer**	No outcome of interest
**2015; Saunders**	Different exposure
**2015; Monson**	No outcome of interest
**2015; Patzer**	Pediatric population
**2015; Francis**	Pediatric population
**2014; Vavallo**	Different exposure
**2014; Rhee**	Overlapping populations
**2014; Goldfarb-Rumyantzev**	Overlapping populations
**2014; Siracuse**	Overlapping populations
**2014; Hogan**	No outcome of interest
**2014[a]; Grace**	Pediatric population
**2014[b]; Grace**	Pediatric population
**2013; Fissell**	No outcome of interest
**2013; Prakash**	No outcome of interest
**2013; Gill**	Overlapping populations
**2013; Grams**	Overlapping populations
**2013; Joshi**	No outcome of interest
**2013; Andreoni**	Different exposure
**2013; Mitsnefes**	Pediatric population
**2013; Patzer**	Pediatric population
**2012; Liu**	No outcome of interest
**2012; Schaefer**	Different exposure
**2012; Arce**	Overlapping populations

**2012; Johansen**	Overlapping populations
**2012; Hall**	Overlapping populations
**2012; Myaskovsky**	No outcome of interest
**2012; Molnar**	Different exposure
**2012; Goldfarb-Rumyantzev**	Overlapping populations
**2012; Kutner**	No outcome of interest
**2012; Amaral**	Overlapping populations
**2012; Shatat**	Pediatric population
**2012; Patzer**	Pediatric population
**2011; Kucirka**	Overlapping populations
**2011[a]; Schold**	No outcome of interest
**2011[b]; Schold**	Overlapping populations
**2011; Nguyen**	Pediatric population
**2010; Kalantar-Zadeh**	Different exposure
**2010; Walker**	No outcome of interest
**2010; Fan**	Overlapping populations
**2009; Frankenfield**	Different exposure
**2009; Gore**	Overlapping populations
**2009; Segev**	Different exposure
**2009; Dudley**	Different exposure
**2009; Chavers**	Pediatric population
**2008; van den Beukel**	Evaluation of immigration
**2008; Naghibi**	No outcome of interest
**2008; Peterson**	No outcome of interest
**2008; Keith**	Overlapping populations
**2005; Murthy**	Overlapping populations
**2005; Hall**	Different exposure
**2004; Prasad**	No outcome of interest
**2003; Drukker**	No outcome of interest
**2003; Frankenfield**	Overlapping populations
**2002; Pisoni**	No outcome of interest
**2000; Epstein**	No outcome of interest
**1999; Korbet**	No outcome of interest
**1999; Isaacs**	No outcome of interest
**1997; Bloembergen**	No outcome of interest
**1996; Bloembergen**	No outcome of interest
**1995; Nee**	Overlapping populations
**1990; Port**	No outcome of interest

Excluded studies

1. Ahearn P, Johansen KL, Tan JC, McCulloch CE, Grimes BA, Ku E. Sex Disparity in Deceased-Donor Kidney Transplant Access by Cause of Kidney Disease. Clin J Am Soc Nephrol [Internet]. 2021 Feb 8 [cited 2022 May 29];16(2):241–50. Available from: https://pubmed.ncbi.nlm.nih.gov/33500250/

2. Amaral S, McCulloch CE, Black E, Winnicki E, Lee B, Roll GR, et al. Trends in Living Donation by Race and Ethnicity Among Children With End-stage Renal Disease in the United States, 1995-2015. Transplant direct [Internet]. 2020 [cited 2022 Jun 4];6(7). Available from: https://pubmed.ncbi.nlm.nih.gov/32766425/

3. Amaral S, Patzer RE, Kutner N, McClellan W. Racial disparities in access to pediatric kidney transplantation since share 35. J Am Soc Nephrol [Internet]. 2012 Jun [cited 2022 Feb 19];23(6):1069–77. Available from: https://pubmed.ncbi.nlm.nih.gov/22539831/

4. Anand A, Malik TH, Dunson J, McDonald MF, Christmann CR, Galvan NTN, et al. Factors associated with long-term graft survival in pediatric kidney transplant recipients. Pediatr Transplant [Internet]. 2021 Jun 1 [cited 2022 Feb 19];25(4). Available from: https://pubmed.ncbi.nlm.nih.gov/33704871/

5. Andreoni KA, Forbes R, Andreoni RM, Phillips G, Stewart H, Ferris M. Age-Related Kidney Transplant Outcomes: Health Disparities Amplified in Adolescence. JAMA Intern Med [Internet]. 2013 Sep 9 [cited 2022 Feb 19];173(16):1524–32. Available from: https://jamanetwork.com/journals/jamainternalmedicine/fullarticle/1722507

6. Arce CM, Mitani AA, Goldstein BA, Winkelmayer WC. Hispanic Ethnicity and Vascular Access Use in Patients Initiating Hemodialysis in the United States. Clin J Am Soc Nephrol [Internet]. 2012 Feb 1 [cited 2022 Jan 26];7(2):289. Available from: /pmc/articles/PMC3280024/

7. Arvelakis A, Lerner S, Wadhera V, Delaney V, Ames S, Benvenisty A, et al. Different outcomes after kidney transplantation between African Americans and Whites: A matter of income? A single-center study. Clin Transplant [Internet]. 2019 Nov 1 [cited 2022 Feb 5];33(11):e13725. Available from: https://onlinelibrary.wiley.com/doi/full/10.1111/ctr.13725

8. Bailey PK, Caskey FJ, MacNeill S, Tomson CRV, Dor FJMF, Ben-Shlomo Y. Mediators of Socioeconomic Inequity in Living-donor Kidney Transplantation: Results From a UK Multicenter Case-Control Study. Transplant direct [Internet]. 2020 [cited 2022 Feb 5];6(4). Available from: https://pubmed.ncbi.nlm.nih.gov/32309626/

9. Bloembergen WE, Port FK, Mauger EA, Briggs JP, Leichtman AB. Gender discrepancies in living related renal transplant donors and recipients. J Am Soc Nephrol. 1996 Aug;7(8):1139–44.

10. Bloembergen WE, Mauger EA, Wolfe RA, Port FK. Association of gender and access to cadaveric renal transplantation. Am J Kidney Dis [Internet]. 1997 [cited 2022 Feb 19];30(6):733–8. Available from: https://pubmed.ncbi.nlm.nih.gov/9398115/

11. Bonthuis M, Cuperus L, Chesnaye NC, Akman S, Melgar AA, Baiko S, et al. Results in the ESPN/ERA-EDTA Registry suggest disparities in access to kidney transplantation but little variation in graft survival of children across Europe. Kidney Int. 2020 Aug;98(2):464–75.

12. Chavers BM, Snyder JJ, Skeans MA, Weinhandl ED, Kasiske BL. Racial disparity trends for graft failure in the US pediatric kidney transplant population, 1980-2004. Am J Transplant [Internet]. 2009 Mar [cited 2022 Jun 4];9(3):543–9. Available from: https://pubmed.ncbi.nlm.nih.gov/19260833/

13. Davidovits M, Reisman L, Cleper R, Bar-Nathan N, Krause I, Dagan A, et al. Long-term outcomes during 37 years of pediatric kidney transplantation: a cohort study comparing ethnic groups. Pediatr Nephrol [Internet]. 2021 Jul 1 [cited 2022 Feb 19];36(7):1881–8. Available from: https://pubmed.ncbi.nlm.nih.gov/33459932/

14. Derrett S, Samaranayaka A, Schollum JBW, McNoe B, Marshall MR, Williams S, et al. Predictors of Health Deterioration Among Older Adults After 12 Months of Dialysis Therapy: A Longitudinal Cohort Study From New Zealand. Am J Kidney Dis. 2017 Dec 1;70(6):798–806.

15. Drukker A, Feinstein S, Rinat C, Rotem-Braun A, Frishberg Y. Cadaver-Donor Renal Transplantation of Children in Israel (1990–2001): Racial Disparities in Health Care Delivery? Pediatrics [Internet]. 2003 Aug 1 [cited 2022 Jan 26];112(2):341–4. Available from: /pediatrics/article/112/2/341/63323/Cadaver-Donor-Renal-Transplantation-of-Children-in

16. Dudley CRK, Johnson RJ, Thomas HL, Ravanan R, Ansell D. Factors that influence access to the national renal transplant waiting list. Transplantation [Internet]. 2009 Jul [cited 2022 Feb 19];88(1):96–102. Available from: https://journals.lww.com/transplantjournal/Fulltext/2009/07150/Factors_That_Influence_Access_to_the_National.16.aspx

17. Dunlop WA, Secombe PJ, Agostino J, Haren F van. Characteristics and outcomes of Aboriginal and Torres Strait Islander patients with dialysis-dependent kidney disease in Australian Intensive Care Units. Intern Med J [Internet]. 2020 Oct 4 [cited 2022 Jan 26]; Available from: https://onlinelibrary.wiley.com/doi/full/10.1111/imj.15077

18. Epstein AM, Ayanian JZ, Keogh JH, Noonan SJ, Armistead N, Cleary PD, et al. Racial Disparities in Access to Renal Transplantation — Clinically Appropriate or Due to Underuse or Overuse? N Engl J Med [Internet]. 2000 Nov 23 [cited 2022 Jan 26];343(21):1537. Available from: /pmc/articles/PMC4598055/

19. Fan PY, Ashby VB, Fuller DS, Boulware LE, Kao A, Norman SP, et al. Access and Outcomes Among Minority Transplant Patients, 1999–2008, with a Focus on Determinants of Kidney Graft Survival. Am J Transplant [Internet]. 2010 Apr [cited 2022 Feb 19];10(4 0 2):1090. Available from: /pmc/articles/PMC3644053/

20. Fissell RB, Fuller DS, Morgenstern H, Gillespie BW, Mendelssohn DC, Rayner HC, et al. Hemodialysis patient preference for type of vascular access: Variation and predictors across countries in the DOPPS. J Vasc Access [Internet]. 2013 Jul 4 [cited 2022 Jan 26];14(3):264–72. Available from: https://journals.sagepub.com/doi/10.5301/jva.5000140

21. Francis A, Didsbury M, Lim WH, Kim S, White S, Craig JC, et al. The impact of socioeconomic status and geographic remoteness on access to pre-emptive kidney transplantation and transplant outcomes among children. Pediatr Nephrol [Internet]. 2016 Dec 21 [cited 2022 Jun 4];31(6):1011–9. Available from: https://pubmed.ncbi.nlm.nih.gov/26692022/

22. Frankenfield DL, Krishnan SM, Ashby VB, Shearon TH, Rocco M V., Saran R. Differences in mortality among Mexican-American, Puerto Rican, and Cuban-American dialysis patients in the United States. Am J Kidney Dis [Internet]. 2009 Apr [cited 2022 Jan 26];53(4):647–57. Available from: https://pubmed.ncbi.nlm.nih.gov/19150157/

23. Frankenfield DL, Rocco M V., Roman SH, McClellan WM. Survival advantage for adult Hispanic hemodialysis patients? Findings from the end-stage renal disease clinical performance measures project. J Am Soc Nephrol [Internet]. 2003 Jan 1 [cited 2022 Jan 26];14(1):180–6. Available from: https://pubmed.ncbi.nlm.nih.gov/12506150/

24. Gander JC, Zhang X, Plantinga L, Paul S, Basu M, Pastan SO, et al. Racial disparities in preemptive referral for kidney transplantation in Georgia. Clin Transplant [Internet]. 2018 Sep 1 [cited 2022 Feb 18];32(9):e13380. Available from: https://onlinelibrary.wiley.com/doi/full/10.1111/ctr.13380

25. Garcia-Garcia G, Tonelli M, Ibarra-Hernandez M, Chavez-Iñiguez JS, Oseguera-Vizcaino MC. Access to kidney transplantation in Mexico, 2007–2019: a call to end disparities in transplant care. BMC Nephrol [Internet]. 2021 Dec 1 [cited 2022 Jan 26];22(1):1–12. Available from: https://bmcnephrol.biomedcentral.com/articles/10.1186/s12882-021-02294-1

26. Gill J, Dong J, Rose C, Johnston O, Landsberg D, Gill J. The effect of race and income on living kidney donation in the United States. J Am Soc Nephrol [Internet]. 2013 Nov 1 [cited 2022 Feb 18];24(11):1872–9. Available from: https://jasn.asnjournals.org/content/24/11/1872

27. Gillis KA, Lees JS, Ralston MR, Glen JA, Stevenson KS, McManus SK, et al. Interaction between socioeconomic deprivation and likelihood of pre-emptive transplantation: influence of competing risks and referral characteristics – a retrospective study. Transpl Int [Internet]. 2019 Feb 1 [cited 2022 Feb 18];32(2):153–62. Available from: https://onlinelibrary.wiley.com/doi/full/10.1111/tri.13336

28. Godara S, Jeswani J. Women donate, men receive: gender disparity among renal donors. Saudi J Kidney Dis Transpl [Internet]. 2019 Nov 1 [cited 2022 Jan 26];30(6):1439–41. Available from: https://pubmed.ncbi.nlm.nih.gov/31929292/

29. Goldfarb-Rumyantzev AS, Sandhu GS, Baird B, Barenbaum A, Yoon JH, Dimitri N, et al. Effect of education on racial disparities in access to kidney transplantation. Clin Transplant [Internet]. 2012 Jan [cited 2022 Feb 19];26(1):74–81. Available from: https://pubmed.ncbi.nlm.nih.gov/21198857/

30. Goldfarb-Rumyantzev AS, Syed W, Patibandla BK, Narra A, Desilva R, Chawla V, et al. Geographic disparities in arteriovenous fistula placement in patients approaching hemodialysis in the United States. Hemodial Int [Internet]. 2014 Jul 1 [cited 2022 Jan 26];18(3):686–94. Available from: https://onlinelibrary.wiley.com/doi/full/10.1111/hdi.12141

31. Golestaneh L, Cavanaugh KL, Lo Y, Karaboyas A, Melamed ML, Johns TS, et al. Community Racial Composition and Hospitalization Among Patients Receiving In-Center Hemodialysis. Am J Kidney Dis [Internet]. 2020 Dec 1 [cited 2022 Jan 26];76(6):754. Available from: /pmc/articles/PMC7844565/

32. Gore JL, Danovitch GM, Litwin MS, Pham PTT, Singer JS. Disparities in the utilization of live donor renal transplantation. Am J Transplant [Internet]. 2009 May [cited 2022 Feb 19];9(5):1124–33. Available from: https://pubmed.ncbi.nlm.nih.gov/19422338/

33. Grace BS, Kara T, Kennedy SE, McDonald SP. Racial disparities in pediatric kidney transplantation in New Zealand. Pediatr Transplant [Internet]. 2014 [cited 2022 Jun 4];18(7):689–97. Available from: https://pubmed.ncbi.nlm.nih.gov/25039826/

34. Grace BS, Kennedy SE, Clayton PA, McDonald SP. Racial disparities in paediatric kidney transplantation. Pediatr Nephrol [Internet]. 2014 Jan [cited 2022 Jun 4];29(1):125–32. Available from: https://pubmed.ncbi.nlm.nih.gov/23928908/

35. Grams ME, Po-Han BC, Coresh J, Segev DL. Preemptive deceased donor kidney transplantation: considerations of equity and utility. Clin J Am Soc Nephrol [Internet]. 2013 Apr 5 [cited 2022 Feb 18];8(4):575–82. Available from: https://pubmed.ncbi.nlm.nih.gov/23371953/

36. Grossi AA, Maggiore U, Puoti F, Grossi PA, Picozzi M, Cardillo M. Association of immigration background with kidney graft function in a publicly funded health system: a nationwide retrospective cohort study in Italy. Transpl Int [Internet]. 2020 Nov 1 [cited 2022 Jan 26];33(11):1405–16. Available from: https://onlinelibrary.wiley.com/doi/full/10.1111/tri.13688

37. Hall EC, James NT, Garonzik Wang JM, Berger JC, Montgomery RA, Dagher NN, et al. Center-Level Factors and Racial Disparities in Living Donor Kidney Transplantation. Am J Kidney Dis [Internet]. 2012 Jun 1 [cited 2022 Feb 18];59(6):849–57. Available from: http://www.ajkd.org/article/S0272638612001084/fulltext

38. Hall YN, Sugihara JG, Go AS, Chertow GM. Differential mortality and transplantation rates among Asians and Pacific Islanders with ESRD. J Am Soc Nephrol [Internet]. 2005 [cited 2022 Feb 19];16(12):3711–20. Available from: https://pubmed.ncbi.nlm.nih.gov/16236803/

39. Hamoda RE, McPherson LJ, Lipford K, Jacob Arriola K, Plantinga L, Gander JC, et al. Association of sociocultural factors with initiation of the kidney transplant evaluation process. Am J Transplant [Internet]. 2020 Jan 1 [cited 2022 Jan 26];20(1):190–203. Available from: https://pubmed.ncbi.nlm.nih.gov/31278832/

40. Hogan J, Couchoud C, Bonthuis M, Groothoff JW, Jager KJ, Schaefer F, et al. Gender Disparities in Access to Pediatric Renal Transplantation in Europe: Data From the ESPN/ERA-EDTA Registry. Am J Transplant [Internet]. 2016 Jul 1 [cited 2022 Jun 4];16(7):2097–105. Available from: https://pubmed.ncbi.nlm.nih.gov/26783738/

41. Hogan J, Savoye E, Macher MA, Bachetta J, Garaix F, Lahoche A, et al. Rapid access to renal transplant waiting list in children: impact of patient and centre characteristics in France. Nephrol Dial Transplant [Internet]. 2014 Oct 1 [cited 2022 Feb 19];29(10):1973–9. Available from: https://academic.oup.com/ndt/article/29/10/1973/1900073

42. Isaacs RB, Nock SL, Spencer CE, Connors J, Wang XQ, Sawyer R, et al. Racial disparities in renal transplant outcomes. Am J Kidney Dis [Internet]. 1999 [cited 2022 Feb 19];34(4):706–12. Available from: https://pubmed.ncbi.nlm.nih.gov/10516353/

43. Johansen KL, Zhang R, Huang Y, Patzer RE, Kutner NG. Association of race and insurance type with delayed assessment for kidney transplantation among patients initiating dialysis in the United States. Clin J Am Soc Nephrol [Internet]. 2012 Sep 1 [cited 2022 Feb 18];7(9):1490–7. Available from: https://pubmed.ncbi.nlm.nih.gov/22837273/

44. Joshi S, Gaynor JJ, Bayers S, Guerra G, Eldefrawy A, Chediak Z, et al. Disparities among Blacks, Hispanics, and Whites in time from starting dialysis to kidney transplant waitlisting. Transplantation [Internet]. 2013 Jan 27 [cited 2022 Feb 18];95(2):309–18. Available from: https://pubmed.ncbi.nlm.nih.gov/23325005/

45. Kalantar-Zadeh K, Golan E, Shohat T, Streja E, Norris KC, Kopple JD. Survival Disparities within American and Israeli Dialysis Populations: Learning from Similarities and Distinctions across Race and Ethnicity. Semin Dial [Internet]. 2010 Nov [cited 2022 Jan 26];23(6):586. Available from: /pmc/articles/PMC3618910/

46. Keith D, Ashby VB, Port FK, Leichtman AB. Insurance Type and Minority Status Associated with Large Disparities in Prelisting Dialysis among Candidates for Kidney Transplantation. Clin J Am Soc Nephrol [Internet]. 2008 Mar 1 [cited 2022 Feb 19];3(2):463–70. Available from: https://cjasn.asnjournals.org/content/3/2/463

47. Khanal N, Lawton PD, Cass A, McDonald SP. Disparity of access to kidney transplantation by Indigenous and non-Indigenous Australians. Med J Aust [Internet]. 2018 Sep 1 [cited 2022 Feb 18];209(6):261–6. Available from: https://onlinelibrary.wiley.com/doi/full/10.5694/mja18.00304

48. Kim SJ, Gill JS, Knoll G, Campbell P, Cantarovich M, Cole E, et al. Referral for kidney transplantation in Canadian provinces. J Am Soc Nephrol [Internet]. 2019 Sep 1 [cited 2022 Feb 5];30(9):1708–21. Available from: /pmc/articles/PMC6727260/

49. Korbet SM, Shih D, Cline KN, Vonesh EF. Racial differences in survival in an urban peritoneal dialysis program. Am J Kidney Dis. 1999 Oct 1;34(4):713–20.

50. Ku E, Ku E, Ku E, Lee BK, McCulloch CE, Roll GR, et al. Racial and ethnic disparities in kidney transplant access within a theoretical context of medical eligibility. Transplantation [Internet]. 2020 [cited 2022 Feb 5];1437–44. Available from: https://journals.lww.com/transplantjournal/Fulltext/2020/07000/Racial_and_Ethnic_Disparities_in_Kidney_Transplant.24.aspx

51. Ku E, McCulloch CE, Adey DB, Li L, Johansen KL. Racial Disparities in Eligibility for Preemptive Waitlisting for Kidney Transplantation and Modification of eGFR Thresholds to Equalize Waitlist Time. J Am Soc Nephrol [Internet]. 2021 Mar 1 [cited 2022 Feb 5];32(3):677–85. Available from: https://jasn.asnjournals.org/content/32/3/677

52. Ku E, McCulloch CE, Grimes BA, Johansen KL. Racial and Ethnic Disparities in Survival of Children with ESRD. J Am Soc Nephrol [Internet]. 2017 May 1 [cited 2022 Jun 4];28(5):1584–91. Available from: https://pubmed.ncbi.nlm.nih.gov/28034898/

53. Kucirka LM, Grams ME, Lessler J, Hall EC, James N, Massie AB, et al. Age and Racial Disparities in Dialysis Survival. JAMA [Internet]. 2011 Aug 10 [cited 2022 Jan 26];306(6):620. Available from: /pmc/articles/PMC3938098/

54. Kutner NG, Zhang R, Huang Y, Johansen KL. Impact of Race on Predialysis Discussions and Kidney Transplant Preemptive Wait-Listing. Am J Nephrol [Internet]. 2012 May [cited 2022 Feb 19];35(4):305–11. Available from: https://www.karger.com/Article/FullText/336891

55. Kwan JM, Hajjiri Z, Chen YF, Metwally A, Perkins DL, Finn PW. Donor and Recipient Ethnicity Impacts Renal Graft Adverse Outcomes. J Racial Ethn Heal Disparities [Internet]. 2018 Oct 1 [cited 2022 Feb 18];5(5):1003–13. Available from: https://link.springer.com/article/10.1007/s40615-017-0447-9

56. Laster M, Soohoo M, Hall C, Streja E, Rhee CM, Ravel VA, et al. Racial-Ethnic Disparities in Mortality and Kidney Transplant Outcomes Among Pediatric Dialysis Patients. Pediatr Nephrol [Internet]. 2017 Apr 1 [cited 2022 Jan 26];32(4):685. Available from: /pmc/articles/PMC5392236/

57. Lee T, Qian J, Thamer M, Allon M. Gender Disparities in Vascular Access Surgical Outcomes in Elderly Hemodialysis Patients. Am J Nephrol [Internet]. 2019 Jan 1 [cited 2022 Jan 26];49(1):11–9. Available from: https://pubmed.ncbi.nlm.nih.gov/30544112/

58. Liu G, Li X, Liu T, Zhao X, Zhang S, Wang J, et al. Gender disparity of living donor renal transplantation in East China. Clin Transplant [Internet]. 2013 Jan 1 [cited 2022 Jan 26];27(1):98–103. Available from: https://onlinelibrary.wiley.com/doi/full/10.1111/ctr.12003

59. Markell M, Brar A, Stefanov DG, Salifu MO. Gender disparity in fistula use at initiation of hemodialysis varies markedly across ESRD networks—Analysis of USRDS data. Hemodial Int [Internet]. 2018 Apr 1 [cited 2022 Jan 26];22(2):168–75. Available from: https://onlinelibrary.wiley.com/doi/full/10.1111/hdi.12579

60. Matter YE, Elhadedy MA, Abbas TM, Zahab MA, Fouda MA, Refaie AF, et al. Impact of Sex Disparities on Outcomes of Living-Donor Kidney Transplant in Egypt: Data of 979 Patients. Exp Clin Transplant [Internet]. 2018 Apr 1 [cited 2022 Feb 18];16(2):133–7. Available from: https://pubmed.ncbi.nlm.nih.gov/28760116/

61. Mıhçıokur S, Soy EHA, Türkçelik E, Akın A, Haberal M. Gender Disparity and the Relationship Between Living Donors and Recipients in Kidney Transplants in an Organ Transplant Center in Turkey. Exp Clin Transplant [Internet]. 2019 [cited 2022 Jan 26];17(Suppl 1):246–9. Available from: https://pubmed.ncbi.nlm.nih.gov/30777567/

62. Miller R, Akateh C, Thompson N, Tumin D, Hayes D, Black SM, et al. County socioeconomic characteristics and pediatric renal transplantation outcomes. Pediatr Nephrol [Internet]. 2018 Jul 1 [cited 2022 Feb 19];33(7):1227–34. Available from: https://pubmed.ncbi.nlm.nih.gov/29532229/

63. Mitsnefes MM, Laskin BL, Dahhou M, Zhang X, Foster BJ. Mortality risk among children initially treated with dialysis for end-stage kidney disease, 1990-2010. JAMA [Internet]. 2013 May 8 [cited 2022 Jun 4];309(18):1921–9. Available from: https://pubmed.ncbi.nlm.nih.gov/23645144/

64. Molnar MZ, Langer RM, Remport A, Czira ME, Rajczy K, Kalantar-Zadeh K, et al. Roma ethnicity and clinical outcomes in kidney transplant recipients. Int Urol Nephrol [Internet]. 2012 Jun [cited 2022 Feb 19];44(3):945–54. Available from: https://pubmed.ncbi.nlm.nih.gov/22116678/

65. Monson RS, Kemerley P, Walczak D, Benedetti E, Oberholzer J, Danielson KK. Disparities in completion rates of the medical prerenal transplant evaluation by race or ethnicity and gender. Transplantation [Internet]. 2015 Jan 15 [cited 2022 Feb 18];99(1):236–42. Available from: https://journals.lww.com/transplantjournal/Fulltext/2015/01150/Disparities_in_Completion_Rates_of_the_Medical.39.aspx

66. Murthy BVR, Molony DA, Stack AG. Survival Advantage of Hispanic Patients Initiating Dialysis in the United States Is Modified by Race. J Am Soc Nephrol [Internet]. 2005 Mar 1 [cited 2022 Jan 26];16(3):782–90. Available from: https://jasn.asnjournals.org/content/16/3/782

67. Mustian MN, Kumar V, Stegner K, Mompoint-Williams D, Hanaway M, Deierhoi MH, et al. Mitigating Racial and Gender Disparities in Access to Living Donor Kidney Transplantation: Impact of the Nation's Longest Single-Center Kidney Chain. Ann Surg [Internet]. 2019 Oct 1 [cited 2022 Jan 26];270(4):639. Available from: /pmc/articles/PMC6788625/

68. Myaskovsky L, Almario Doebler D, Posluszny DM, Dew MA, Unruh M, Fried LF, et al. Perceived discrimination predicts longer time to be accepted for kidney transplant. Transplantation [Internet]. 2012 Feb 27 [cited 2022 Feb 19];93(4):423–9. Available from: https://pubmed.ncbi.nlm.nih.gov/22228417/

69. Naghibi O, Naghibi M, Nazemian F. Saudi Journal of Kidney Diseases and Transplantation. Saudi J Kidney Dis Transplant [Internet]. 2008 [cited 2022 Jan 26];19(4):545. Available from: https://www.sjkdt.org/article.asp?issn=1319-2442;year=2008;volume=19;issue=4;spage=545;epage=550;aulast=Naghibi

70. Nee R, Martinez-Osorio J, Yuan CM, Little DJ, Watson MA, Agodoa L, et al. Survival Disparity of African American Versus Non-African American Patients With ESRD Due to SLE. Am J Kidney Dis [Internet]. 2015 Oct 1 [cited 2022 Jan 26];66(4):630–7. Available from: https://pubmed.ncbi.nlm.nih.gov/26002293/

71. Ng DK, Moxey-Mims M, Warady BA, Furth SL, Muñoz A. Racial differences in renal replacement therapy initiation among children with a nonglomerular cause of chronic kidney disease. Ann Epidemiol [Internet]. 2016 Nov 1 [cited 2022 Feb 19];26(11):780-787.e1. Available from: https://pubmed.ncbi.nlm.nih.gov/27789133/

72. Nguyen S, Martz K, Stablein D, Neu A. Wait list status of pediatric dialysis patients in North America. Pediatr Transplant [Internet]. 2011 Jun [cited 2022 Jun 4];15(4):376–83. Available from: https://pubmed.ncbi.nlm.nih.gov/21466638/

73. Nguyen T, Sise ME, Delgado C, Williams W, Reese P, Goldberg D. Race, Education, and Gender Disparities in Transplantation of Kidneys from Hepatitis C Viremic Donors. Transplantation [Internet]. 2021 [cited 2022 Jan 27];1850–7. Available from: https://journals.lww.com/transplantjournal/Fulltext/2021/08000/Race,_Education,_and_Gender_Disparities_in.30.aspx

74. Nogueira PCK, de Carvalho MFC, de Santis Feltran L, Konstantyner T, Sesso R. Inequality in pediatric kidney transplantation in Brazil. Pediatr Nephrol [Internet]. 2016 Mar 1 [cited 2022 Jan 26];31(3):501–7. Available from: https://link.springer.com/article/10.1007/s00467-015-3226-z

75. Patzer RE, Amaral S, Klein M, Kutner N, Perryman JP, Gazmararian JA, et al. Racial disparities in pediatric access to kidney transplantation: does socioeconomic status play a role? Am J Transplant [Internet]. 2012 [cited 2022 Jun 4];12(2):369–78. Available from: https://pubmed.ncbi.nlm.nih.gov/22226039/

76. Patzer RE, Sayed BA, Kutner N, McClellan WM, Amaral S. Racial and ethnic differences in pediatric access to preemptive kidney transplantation in the United States. Am J Transplant [Internet]. 2013 Jul [cited 2022 Jun 4];13(7):1769–81. Available from: https://pubmed.ncbi.nlm.nih.gov/23731389/

77. Patzer RE, McPherson L, Wang Z, Plantinga LC, Paul S, Ellis M, et al. Dialysis facility referral and start of evaluation for kidney transplantation among patients treated with dialysis in the Southeastern United States. Am J Transplant [Internet]. 2020 Aug 1 [cited 2022 Feb 5];20(8):2113–25. Available from: https://pubmed.ncbi.nlm.nih.gov/31981441/

78. Patzer RE, Mohan S, Kutner N, Mcclellan WM, Amaral S. Racial and ethnic disparities in pediatric renal allograft survival in the United States. Kidney Int [Internet]. 2015 Mar 1 [cited 2022 Jun 4];87(3):584–92. Available from: https://pubmed.ncbi.nlm.nih.gov/25337773/

79. Patzer RE, Plantinga LC, Paul S, Gander J, Krisher J, Sauls L, et al. Variation in Dialysis Facility Referral for Kidney Transplantation Among Patients With End-Stage Renal Disease in Georgia. JAMA [Internet]. 2015 Aug 11 [cited 2022 Feb 18];314(6):582–94. Available from: https://pubmed.ncbi.nlm.nih.gov/26262796/

80. Pecoits-Filho R, Ribeiro SC, Kirk A, Da Silva HS, Pille A, Falavinha RS, et al. Racial and social disparities in the access to automated peritoneal dialysis - results of a national PD cohort. Sci Reports 2017 71 [Internet]. 2017 Jul 12 [cited 2022 Jan 26];7(1):1–7. Available from: https://www.nature.com/articles/s41598-017-05544-1

81. Peracha J, Hayer M, Sharif A. Gender Disparity in Living-Donor Kidney Transplant Among Minority Ethnic Groups. Exp Clin Transpl. 2016;14(2):139–45.

82. Peterson WJ, Barker J, Allon M. Disparities in Fistula Maturation Persist Despite Preoperative Vascular Mapping. Clin J Am Soc Nephrol [Internet]. 2008 Mar 1 [cited 2022 Jan 26];3(2):437–41. Available from: https://cjasn.asnjournals.org/content/3/2/437

83. Pisoni RL, Young EW, Dykstra DM, Greenwood RN, Hecking E, Gillespie B, et al. Vascular access use in Europe and the United States: Results from the DOPPS. Kidney Int. 2002 Jan 1;61(1):305–16.

84. Pletcher J, Koizumi N, Nayebpour M, Alam Z, Ortiz J. Improved outcomes after live donor renal transplantation for septuagenarians. Clin Transplant [Internet]. 2020 Mar 1 [cited 2022 Jan 26];34(3):e13808. Available from: https://onlinelibrary.wiley.com/doi/full/10.1111/ctr.13808

85. Ploos van Amstel S, Noordzij M, Borzych-Duzalka D, Chesnaye NC, Xu H, Rees L, et al. Mortality in Children Treated With Maintenance Peritoneal Dialysis: Findings From the International Pediatric Peritoneal Dialysis Network Registry. Am J Kidney Dis. 2021 Sep 1;78(3):380–90.

86. Plumb LA, Sinha MD, Casula A, Inward CD, Marks SD, Caskey FJ, et al. Associations between Deprivation, Geographic Location, and Access to Pediatric Kidney Care in the United Kingdom. Clin J Am Soc Nephrol [Internet]. 2021 [cited 2022 Jun 4];16(2):194–203. Available from: https://pubmed.ncbi.nlm.nih.gov/33468533/

87. Port FK, Wolfe RA, Levin NW, Guire KE, Ferguson CW. Income and survival in chronic dialysis patients. ASAIO Trans [Internet]. 1990 Jul [cited 2022 Jan 26];36(3). Available from: https://pubmed.ncbi.nlm.nih.gov/2252648/

88. Prakash S, Perzynski AT, Austin PC, Wu CF, Lawless ME, Paterson JM, et al. Neighborhood socioeconomic status and barriers to peritoneal dialysis: A mixed methods study. Clin J Am Soc Nephrol [Internet]. 2013 Oct 7 [cited 2022 Jan 26];8(10):1741–9. Available from: /pmc/articles/PMC3789354/

89. Prasad S, Singh S, Duncan N, Cairns TDH, Griffith M, Hakim N, et al. Ethnicity and survival on dialysis in west London. Kidney Int. 2004 Dec 1;66(6):2416–21.

90. Rhee CM, Lertdumrongluk P, Streja E, Park J, Moradi H, Lau WL, et al. Impact of Age, Race and Ethnicity on Dialysis Patient Survival and Kidney Transplantation Disparities. Am J Nephrol [Internet]. 2014 [cited 2022 Jan 26];39(3):183–94. Available from: https://www.karger.com/Article/FullText/358497

91. Riad S, Jackson S, Chinnakotla S, Verghese P. Primary pediatric deceased-donor kidney transplant recipients outcomes by immunosuppression induction received in the United States. Pediatr Transplant [Internet]. 2021 Aug 1 [cited 2022 Jun 4];25(5). Available from: https://pubmed.ncbi.nlm.nih.gov/33314638/

92. Saunders MR, Lee H, Alexander GC, Tak HJ, Thistlethwaite JR, Ross LF. Racial disparities in reaching the renal transplant waitlist: is geography as important as race? Clin Transplant [Internet]. 2015 Jun 1 [cited 2022 Feb 18];29(6):531–8. Available from: https://onlinelibrary.wiley.com/doi/full/10.1111/ctr.12547

93. Schaefer F, Borzych-Duzalka D, Azocar M, Munarriz RL, Sever L, Aksu N, et al. Impact of global economic disparities on practices and outcomes of chronic peritoneal dialysis in children: Insights from the international pediatric peritoneal dialysis network registry. Perit Dial Int [Internet]. 2012 Jul 1 [cited 2022 Jan 26];32(4):399–409. Available from: https://journals.sagepub.com/doi/10.3747/pdi.2012.00126

94. Schold JD, Gregg JA, Harman JS, Hall AG, Patton PR, Meier-Kriesche HU. Barriers to evaluation and wait listing for kidney transplantation. Clin J Am Soc Nephrol [Internet]. 2011 Jul 1 [cited 2022 Feb 19];6(7):1760–7. Available from: https://pubmed.ncbi.nlm.nih.gov/21597030/

95. Schold JD, Srinivas TR, Braun WE, Shoskes DA, Nurko S, Poggio ED. The relative risk of overall graft loss and acute rejection among African American renal transplant recipients is attenuated with advancing age. Clin Transplant [Internet]. 2011 Sep [cited 2022 Feb 19];25(5):721–30. Available from: https://pubmed.ncbi.nlm.nih.gov/20964717/

96. Schouten RW, Haverkamp GL, Loosman WL, Chandie Shaw PK, van Ittersum FJ, Smets YFC, et al. Ethnic Differences in the Association of Depressive Symptoms with Clinical Outcome in Dialysis Patients. J racial Ethn Heal disparities [Internet]. 2019 Oct 1 [cited 2022 Jan 26];6(5):990–1000. Available from: https://pubmed.ncbi.nlm.nih.gov/31215016/

97. Segev DL, Kucirka LM, Oberai PC, Parekh RS, Boulware LE, Powe NR, et al. Age and Comorbidities Are Effect Modifiers of Gender Disparities in Renal Transplantation. J Am Soc Nephrol [Internet]. 2009 Mar [cited 2022 Feb 19];20(3):621. Available from: /pmc/articles/PMC2653677/

98. Seipp R, Zhang N, Nair SS, Khamash H, Sharma A, Leischow S, et al. Patient and allograft outcomes after kidney transplant for the Indigenous patients in the United States. PLoS One [Internet]. 2021 Feb 1 [cited 2022 Feb 5];16(2):e0244492. Available from: https://journals.plos.org/plosone/article?id=10.1371/journal.pone.0244492

99. Shah S, Leonard AC, Meganathan K, Christianson AL, Thakar C V. Temporal Trends in Incident Mortality in Dialysis Patients: Focus on Sex and Racial Disparities. Am J Nephrol [Internet]. 2019 Mar 1 [cited 2022 Jan 26];49(3):241–53. Available from: https://www.karger.com/Article/FullText/497446

100. Shatat IF, Taber DJ, Shrivastava S, Weimert NA, Sas DJ, Chavin KD, et al. Graft outcomes in pediatric kidney transplantation: focus on the role of race. Saudi J Kidney Dis Transpl [Internet]. 2012 [cited 2022 Jun 4];23(4):684–92. Available from: https://pubmed.ncbi.nlm.nih.gov/22805378/

101. Siracuse JJ, Gill HL, Epelboym I, Wollstein A, Kotsurovskyy Y, Catz D, et al. Effect of Race and Insurance Status on Outcomes after Vascular Access Placement for Hemodialysis. Ann Vasc Surg. 2014 May 1;28(4):964–9.

102. Sule S, Fivush B, Neu A, Furth S. Increased risk of death in African American patients with end-stage renal disease secondary to lupus. Clin Kidney J [Internet]. 2014 Feb [cited 2022 Jan 26];7(1):40. Available from: /pmc/articles/PMC4389169/

103. Taber DJ, Egede LE, Baliga PK. Outcome disparities between African Americans and Caucasians in contemporary kidney transplant recipients. Am J Surg [Internet]. 2017 Apr 1 [cited 2022 Feb 18];213(4):666–72. Available from: http://www.americanjournalofsurgery.com/article/S000296101630900X/fulltext

104. Taber DJ, Hamedi M, Rodrigue JR, Gebregziabher MG, Srinivas TR, Baliga PK, et al. Quantifying the Race Stratified Impact of Socioeconomics on Graft Outcomes in Kidney Transplant Recipients. Transplantation [Internet]. 2016 Jun 21 [cited 2022 Feb 18];100(7):1550–7. Available from: https://pubmed.ncbi.nlm.nih.gov/26425875/

105. Tahir S, Gillott H, Jackson-Spence F, Nath J, Mytton J, Evison F, et al. Do outcomes after kidney transplantation differ for black patients in England versus New York State? A comparative, population-cohort analysis. BMJ Open [Internet]. 2017 May 1 [cited 2022 Feb 18];7(5). Available from: https://pubmed.ncbi.nlm.nih.gov/28487457/

106. Tang W, Grace B, McDonald SP, Hawley CM, Badve S V., Boudville NC, et al. Socio-Economic Status and Peritonitis in Australian Non-Indigenous Peritoneal Dialysis Patients. Perit Dial Int [Internet]. 2015 Jul 1 [cited 2022 Jan 26];35(4):450. Available from: /pmc/articles/PMC4520728/

107. Tjaden LA, Noordzij M, Van Stralen KJ, Kuehni CE, Raes A, Cornelissen EAM, et al. Racial Disparities in Access to and Outcomes of Kidney Transplantation in Children, Adolescents, and Young Adults: Results From the ESPN/ERA-EDTA (European Society of Pediatric Nephrology/European Renal Association-European Dialysis and Transplant Association) Registry. Am J Kidney Dis [Internet]. 2016 Feb 1 [cited 2022 Jun 4];67(2):293–301. Available from: https://pubmed.ncbi.nlm.nih.gov/26561356/

108. Van Den Beukel TO, Dekker FW, Siegert CEH. Increased survival of immigrant compared to native dialysis patients in an urban setting in the Netherlands. Nephrol Dial Transplant [Internet]. 2008 Nov 1 [cited 2022 Jan 26];23(11):3571–7. Available from: https://academic.oup.com/ndt/article/23/11/3571/1938681

109. Vavallo A, Lucarelli G, Spilotros M, Bettocchi C, Palazzo S, Selvaggi FP, et al. Impact of donor-recipient gender on kidney graft and patient survival: Short- and long-term outcomes. World J Urol [Internet]. 2014 Aug 2 [cited 2022 Jan 26];32(3):709–14. Available from: https://link.springer.com/article/10.1007/s00345-013-1137-9

110. Vogelzang JL, Van Stralen KJ, Noordzij M, Diez JA, Carrero JJ, Couchoud C, et al. Mortality from infections and malignancies in patients treated with renal replacement therapy: data from the ERA-EDTA registry. Nephrol Dial Transplant [Internet]. 2015 Jun 1 [cited 2022 Jan 26];30(6):1028–37. Available from: https://academic.oup.com/ndt/article/30/6/1028/2324882

111. Walker DR, Inglese GW, Sloand JA, Just PM. Dialysis Facility and Patient Characteristics Associated with Utilization of Home Dialysis. Clin J Am Soc Nephrol [Internet]. 2010 Sep 1 [cited 2022 Jan 26];5(9):1649–54. Available from: https://cjasn.asnjournals.org/content/5/9/1649

112. Weigert A, Drozdz M, Silva F, Frazão J, Alsuwaida A, Krishnan M, et al. Influence of gender and age on haemodialysis practices: a European multicentre analysis. Clin Kidney J [Internet]. 2020 Aug 13 [cited 2022 Jan 26];13(2):217. Available from: /pmc/articles/PMC7147302/

113. Wilkins LJ, Nyame YA, Gan V, Lin S, Greene DJ, Flechner SM, et al. A Contemporary Analysis of Outcomes and Modifiable Risk Factors of Ethnic Disparities in Kidney Transplantation. J Natl Med Assoc. 2019 Apr 1;111(2):202–9.

114. Williams N, Korneffel K, Koizumi N, Ortiz J. African American polycystic kidney patients receive higher risk kidneys, but do not face increased risk for graft failure or post-transplant mortality. Am J Surg [Internet]. 2021 May 1 [cited 2022 Jan 27];221(5):1093–103. Available from: http://www.americanjournalofsurgery.com/article/S0002961020305973/fulltext

115. Wong YHS, Wong G, Johnson DW, McDonald S, Clayton P, Boudville N, et al. Socio-economic disparity, access to care and patient-relevant outcomes after kidney allograft failure. Transpl Int [Internet]. 2021 Nov 1 [cited 2022 Feb 5];34(11):2329–40. Available from: https://pubmed.ncbi.nlm.nih.gov/34339557/

116. Woo K, Gascue L, Goldman DP, Romley JA. Variations in outcomes of hemodialysis vascular access by race/ethnicity in the elderly. J Vasc Surg [Internet]. 2017 Mar 1 [cited 2022 Jan 26];65(3):783-792.e4. Available from: https://pubmed.ncbi.nlm.nih.gov/28027805/

117. Wu DA, Robb ML, Watson CJE, Forsythe JLR, Tomson CRV, Cairns J, et al. Barriers to living donor kidney transplantation in the United Kingdom: a national observational study. Nephrol Dial Transplant [Internet]. 2017 May 1 [cited 2022 Feb 18];32(5):890–900. Available from: https://pubmed.ncbi.nlm.nih.gov/28379431/

118. Yan G, Shen JI, Harford R, Yu W, Nee R, Clark MJ, et al. Racial and Ethnic Variations in Mortality Rates for Patients Undergoing Maintenance Dialysis Treated in US Territories Compared with the US 50 States. Clin J Am Soc Nephrol [Internet]. 2020 Jan 1 [cited 2022 Jan 26];15(1):101–8. Available from: https://pubmed.ncbi.nlm.nih.gov/31857376/

119. Zarkowsky DS, Arhuidese IJ, Hicks CW, Canner JK, Qazi U, Obeid T, et al. Racial/Ethnic Disparities Associated With Initial Hemodialysis Access. JAMA Surg [Internet]. 2015 Jun 1 [cited 2022 Jan 26];150(6):529–36. Available from: https://jamanetwork.com/journals/jamasurgery/fullarticle/2280714

Supplementary S3: Study characteristics

Suppl. Table S2. Methodological characteristics of the included studies

Study	Country	Design	Data source	Sample size	Time period	Population	Male sex (%)	Peritoneal dialysis (%)	Diabetes mellitus (%)
**2021; Qian**	USA	Retrospective cohort	USRDS	14,077	2010-2012	Hemodialysis, start with CVC, age ≥67 years	53	0	65
**2021; MacRae**	Canada	Prospective cohort	5 Canadian dialysis programs	2,375	2004-2012	Hemodialysis	60.9	0	56.1
**2021; Pisoni**	International	Prospective cohort	DOPPS study	2,040	2009-2015	Hemodialysis	60.8	0	60
**2021; Schellartz**	Germany	Retrospective cohort	Health insurance data	4,727	2012-2016	Hemodialysis/ Peritoneal dialysis	58	3.7	N/A
**2021; Barth**	Hungary	Retrospective cohort	Study data	254	2020	ESKD, age ≤75 years	65	11	20
**2021; Wesselman**	USA	Prospective cohort	Study data	1,056	2010-2012	ESKD	62	10	N/A
**2021; Cirillo**	Italy	Retrospective cohort	Campania province data	11,716	2014-2018	ESKD	61.5	1.4	N/A
**2021; Ahearn**	USA	Retrospective cohort	USRDS	1,478,037	2005-2017	ESKD	57	N/A	33.4
**2021; Rita**	Switzerland	Prospective cohort	Swiss Transplant Cohort Study	1,985	2008-2017	Living/deceased-donor transplantation	64	-	N/A
**2021; Wiley**	USA	Retrospective cohort	Study data	623	2000-2018	Living/deceased-donor transplantation	60	-	33
**2021; Belyaev**	New Zealand	Retrospective cohort	ANZDATA	837	2008-2018	Living/deceased-donor transplantation	63.3	-	35.2
**2021; Park**	South Korea	Retrospective cohort	Health insurance data	12,564	2007-2015	ESKD, age ≤70 years	59.1	15.6	33.9
**2020; Arya**	USA	Retrospective cohort	USRDS	74,194	2010-2013	Hemodialysis, start with CVC, age ≥66 years	53	0	49
**2020; Arhuidese**	USA	Retrospective cohort	USRDS	359,942	2007-2014	Hemodialysis	57.2	0	54
**2020; Copeland**	USA	Retrospective cohort	Optum Clinformatics Data Mart	7,584	2012-2016	Hemodialysis, start with CVC	57	0	83
**2020; Krishnasamy**	Australia	Retrospective cohort	ANZDATA	11,844	2002-2012	Hemodialysis	62.2	0	31.2

**2020; Yolgosteren**	Turkey	Retrospective cohort	Study data	349	2008	Hemodialysis, start with AVF	57.5	0	34.8
**2020; Ku**	USA	Prospective cohort	CRIC study	678	2003-2013	Hemodialysis/ Peritoneal dialysis	60.6	13.9	70.4
**2020; Hayat**	New Zealand	Prospective cohort	New Zealand Peritoneal Dialysis Registry	6,379	1995-2014	Peritoneal dialysis	54.7	100	47.5
**2020; Vogel**	USA	Retrospective cohort	USRDS	1,324,0 37	2000-2014	Hemodialysis/ Peritoneal dialysis	56.1	7.7	47.8
**2020; Shen**	USA	Retrospective cohort	USRDS	523,52 6	2005-2013	Hemodialysis/ Peritoneal dialysis/ Home dialysis	57.6	8	45.6
**2020; Melk**	Germany	Retrospective cohort	Health insurance data	8,921	2005-2013	ESKD	55.9	N/A	50.9
**2020; Park**	South Korea	Retrospective cohort	Health insurance data	12,889	2007-2015	ESKD, age ≤70 years	59.1	10.4	55.9
**2020; Pruthi**	United Kingdom	Prospective cohort	ATTOM study	2,676	2011-2013	ESKD, age ≤75 years	64	N/A	40
**2020; Murphy**	USA	Prospective cohort	Scientific Registry of Transplant Recipients	3,013	2009-2018	ESKD	61	11	40
**2020; Ng**	USA	Prospective cohort	Study data	1,055	2010-2018	ESKD	61.4	N/A	N/A
**2019; Nee**	USA	Retrospective cohort	USRDS	56,194	2007-2014	Hemodialysis/ Peritoneal dialysis	49.5	0.5	63.3
**2019; Howson**	Australia	Retrospective cohort	ANZDATA	616	2000-2010	Living/deceased-donor transplantation	62.8	-	15.1
**2019; King**	USA	Prospective cohort	Scientific Registry of Transplant Recipients	157,07 3	2000-2018	Living/deceased-donor transplantation	60.6	-	28.1
**2019; Myaskovsky**	USA	Prospective cohort	Veterans Affairs Kidney Transplant	611	2010-2012	ESKD	96.9	10.3	N/A
**2019; Ke**	Canada	Retrospective cohort	Health insurance data	396,59 3	1997-2011	ESKD	47.9	N/A	100
**2019; Naylor**	Canada	Retrospective cohort	CORR	4,414	2004-2014	Living/deceased-donor transplantation	63.5	28	32.8
**2018; Shah**	USA	Retrospective cohort	USRDS	885,69 9	2004-2014	Hemodialysis	56.3	0	57
**2018; Huria**	New Zealand	Retrospective cohort	ANZDATA	4,781	2002-2011	Hemodialysis/ Peritoneal dialysis/ Living/deceased-donor transplantation	59.7	32	45

**2018; Lin**	USA	Retrospective cohort	USRDS	5,295	2010-2013	Hemodialysis	53.6	0	60.2
**2018; Krishnasamy**	Australia	Retrospective cohort	ANZDATA	20,810	2004-2013	Hemodialysis/ Peritoneal dialysis	62.5	31.5	42.1
**2018; Keddis**	USA	Retrospective cohort	Study data	600	2012-2016	ESKD	59.2	16.9	60.2
**2018; Williams**	United Kingdom	Retrospective cohort	Study data	1,066	2007-2017	Living/deceased-donor transplantation	59.8	-	N/A
**2018; Sypek**	Australia	Retrospective cohort	ANZDATA	21,213	2006-2015	ESKD	61.8	N/A	35.1
**2018; Liu**	USA	Retrospective cohort	Study data	483	2006-2012	Living/deceased-donor transplantation	61.2	-	31.4
**2018; Chatelet**	France	Retrospective cohort	Cristal database	8,701	2010-2014	Living/deceased-donor transplantation	63.8	10.6	16.8
**2018[a]; Zhang**	USA	Retrospective cohort	USRDS	1,253,1 00	2005-2015	ESKD	56.9	N/A	52.5
**2018[b]; Zhang**	Sweden	Retrospective cohort	Swedish Renal Register	13,982	1995-2013	ESKD	65.6	N/A	33.2
**2018; Peng**	USA	Retrospective cohort	USRDS	3,775	2005-2009	ESKD	55.7	N/A	47.9
**2018; Purnell**	USA	Retrospective cohort	Scientific Registry of Transplant Recipients	453,16 2	1995-2014	ESKD	61	N/A	N/A
**2017; Trivedi**	USA	Retrospective cohort	Medicare Limited Data Set	2,693	2009	Hemodialysis, start with AVF/AVG	56	0	N/A
**2017; McGill**	USA	Retrospective cohort	USRDS	18,883	2010-2011	Hemodialysis, start with CVC, age ≥67 years	51.8	0	45.6
**2017; McKercher**	Australia	Retrospective cohort	ANZDATA	21,832	2001-2013	Hemodialysis	59.9	0	37.7
**2017; Pisoni**	International	Prospective cohort	DOPPS study	4,692	1996-2015	Hemodialysis	61	0	52.4
**2017; Wilmink**	United Kingdom	Retrospective cohort	PROTON	500	2003-2011	Hemodialysis	N/A	0	N/A
**2017; Gulcan**	Turkey	Retrospective cohort	Study data	322	2000-2012	Peritoneal dialysis	48.4	100	N/A
**2017; Kim**	South Korea	Retrospective cohort	Seoul National University Hospital registry	655	2000-2012	Peritoneal dialysis	60.9	100	35.6
**2017; Chan**	Australia	Retrospective cohort	ANZDATA	9,766	1994-2013	Peritoneal dialysis	57	100	29
**2017; Wallace**	USA	Retrospective cohort	Centers for Medicare/ Medicaid Services	369,16 4	2012	Hemodialysis/ Peritoneal dialysis	N/A	N/A	N/A

**2017; Trinh**	Canada	Retrospective cohort	CORR	61,237	1996-2012	Hemodialysis/ Peritoneal dialysis	59	15	24
**2017; Imanishi**	Japan	Prospective cohort	DOPPS	7,974	1999-2011	Hemodialysis	62	100	31
**2017; Gill**	USA	Retrospective cohort	Scientific Registry of Transplant Recipients	77,607	2006-2016	Living/deceased-donor transplantation	61.9	-	23.3
**2017; Zhang**	Sweden	Retrospective cohort	Swedish Renal Register	16,215	1995-2013	Hemodialysis/ Peritoneal dialysis	65.4	N/A	31.3
**2017; Choi**	South Korea	Retrospective cohort	Korean National Health Insurance Cohort Data	1,792	2002-2013	Hemodialysis/ Peritoneal dialysis	56.8	87.4	53.5
**2017; Taber**	USA	Retrospective cohort	USRDS, Veteran Affairs	4,918	2001-2007	Living/deceased-donor transplantation	97.6	-	38.1
**2016; Tamayo Isla**	South Africa	Retrospective cohort	PKDC	340	2007-2014	Hemodialysis/ Peritoneal dialysis	52.1	42.9	10.3
**2016; Liu**	China	Retrospective cohort	Study data	1,778	2006-2013	Peritoneal dialysis	59.5	100	25.3
**2016; Shabankhani**	Iran	Retrospective cohort	Study data	500	2007-2013	Hemodialysis	53.6	100	46.2
**2016; Mucsi**	Canada	Retrospective cohort	Study data	1,769	2003-2012	ESKD	60	N/A	38
**2016; Kihal-Talantikite**	France	Retrospective cohort	REIN registry	2,006	2004-2009	Hemodialysis/ Peritoneal dialysis	60.6	12.5	26
**2016; Molmenti**	USA	Retrospective cohort	United Network for Organ Sharing	88,284	1999-2006	Living/deceased-donor transplantation	60.2	-	28.6
**2015; Nee**	USA	Retrospective cohort	USRDS	669,20 6	2007-2012	Hemodialysis	46.7	0	50.4
**2015; Lawton**	Australia	Retrospective cohort	ANZDATA	15,984	1995-2004	Hemodialysis/ Peritoneal dialysis	58.3	25.2	27.7
**2015; van den Beukel**	Denmark	Retrospective cohort	Danish Nephrology Registry	9,170	1995-2010	Hemodialysis/ Peritoneal dialysis	63.6	30.8	23.4
**2015; Park**	South Korea	Prospective cohort	26 centers	946	2009-2013	Hemodialysis	61.4	0	58.1
**2015; Wang**	China	Prospective cohort	Study data	564	2008-2011	Continuous ambulatory peritoneal dialysis, age <80 years	52.8	100	13.5
**2015; Ward**	Ireland	Prospective cohort	Study data	1,794	1990-2009	Hemodialysis	61.2	100	11.9
**2015; Ilori**	USA	Retrospective cohort	United Network for Organ Sharing	44,013	1996-2010	Living/deceased-donor transplantation, age ≥60 years	62.5	-	33.7

**2015; Matsuoka**	USA	Retrospective cohort	Study data	720	2004-2013	Living/deceased-donor transplantation	63.3	-	27.2
**2015; Ward**	Ireland	Retrospective cohort	Irish national transplantation center	1,944	1990-2009	Living/deceased-donor transplantation	62.6	-	N/A
**2015; Arce**	USA	Retrospective cohort	USRDS	105,520	1996-2010	ESKD	62.2	17.4	36
**2014; Goldfarb-Rumyantzev**	USA	Retrospective cohort	USRDS	109,390	2005-2008	Hemodialysis	53.1	0	40.2
**2014; Cole**	United Kingdom	Retrospective cohort	Renalware	1,340	1996-2008	Hemodialysis/ Peritoneal dialysis	61	1.6	28.4
**2014; Burrows**	USA	Retrospective cohort	USRDS	510,666	1995-2009	Hemodialysis	51.2	0	100
**2014; Prasad**	India	Prospective cohort	Study data	328	2005-2009	Peritoneal dialysis, age <75 years	73.8	100	53.7
**2014; Grace**	Australia	Retrospective cohort	ANZDATA	23,281	2000-2011	Hemodialysis/ Peritoneal dialysis	61.7	47.7	38.4
**2014; Kim**	South Korea	Prospective cohort	Study data	495	2009	Peritoneal dialysis	61.4	100	55.2
**2014; Yang**	China	Retrospective cohort	SSOP study	2,264	2011	Peritoneal dialysis	49.9	100	38.8
**2014; de Moraes**	Brazil	Prospective cohort	Study data	3,311	2004-2011	Peritoneal dialysis	47	100	41
**2014; Teixeira**	Brazil	Retrospective cohort	Study data	162	2007-2011	Hemodialysis	55.6	100	33.3
**2013; Yan**	USA	Retrospective cohort	USRDS	1,282,201	1995-2009	Hemodialysis/ Peritoneal dialysis	54.6	N/A	49.7
**2013; Chang**	South Korea	Prospective cohort	Study data	441	2000-2005	Peritoneal dialysis	54.4	100	51.5
**2013; Ros**	Sweden	Retrospective cohort	Andalusian SICATA Registry	1,458	1999-2010	Peritoneal dialysis	57.1	100	19.4
**2013; Fernades**	Brazil	Prospective cohort	BRAZPD	2,159	2004-2007	Peritoneal dialysis	36.3	100	34.1
**2013; Kimmel**	USA	Retrospective cohort	USRDS	589,036	2000-2008	Hemodialysis	54.9	0	55.2
**2013; Arce**	USA	Retrospective cohort	USRDS	417,801	1995-2007	ESKD age ≤75 years	56.9	10.4	55.9
**2013; Purnell**	USA	Retrospective cohort	USRDS, United Network for Organ Sharing	208,736	2005-2008	ESKD, age ≤70 years	57	N/A	55.8
**2013; Waterman**	USA	Prospective cohort	Study data	695	2004-2007	ESKD	58.7	N/A	30.6

**2013; Promislow**	Canada	Retrospective cohort	CORR	30,688	2000-2009	ESKD	59.4	21.5	36.8
**2012; Sood**	Canada	Retrospective cohort	CORR	31,576	2000-2009	Hemodialysis/ Peritoneal dialysis	60.7	23.9	45.7
**2012; Woo**	USA	Retrospective cohort	Southern California Permanente Medical Group Renal Database	5,821	2000-2008	Hemodialysis	59	0	61
**2012; van den Beukel**	Netherlands	Prospective cohort	NECOSAD-II	1,944	1997-2007	Hemodialysis/ Peritoneal dialysis	62.5	40	14.2
**2012; Marinovich**	Argentina	Retrospective cohort	National Registry of Chronic Dialysis	13,466	2004-2008	Hemodialysis	57.2	0	35.9
**2012; Xu**	China	Retrospective cohort	Study data	2,171	2011	Peritoneal dialysis	49.5	100	37.6
**2012; Huang**	Taiwan	Prospective cohort	Study data	935	2008	Hemodialysis	50.6	100	15.1
**2012; Lockridge**	Sweden	Prospective cohort	Study data	191	1996-2009	Home dialysis	62.3	100	22.5
**2012; Neri**	Italy	Retrospective cohort	MigliorDialisi dataset	1,238	2008-2011	ESKD	63.6	0	N/A
**2012; Couchoud**	France	Retrospective cohort	REIN registry	14,883	2002-2009	ESKD, age <75 years	63.8	N/A	37.4
**2012; Goldfarb-Rumyantzev**	USA	Retrospective cohort	USRDS, United Network for Organ Sharing	79,223	1990-2007	Living/deceased-donor transplantation	61.4	-	31
**2012; Udayaraj**	United Kingdom	Retrospective cohort	UK Renal Registry	12,282	1997-2004	Hemodialysis/ Peritoneal dialysis, age <70 years	60.6	N/A	N/A
**2012; Patzer**	USA	Retrospective cohort	USRDS, United Network for Organ Sharing	2,291	2005-2007	ESKD	57.3	N/A	38.4
**2012; Garg**	USA	Retrospective cohort	Organ Transplant Tracking Record	533	2001-2009	Living/deceased-donor transplantation	58.9	-	52.2
**2011; Lopez-Vargas**	Australia/New Zealand	Prospective cohort	9 centers	3,099	2008	Hemodialysis	64	0	45
**2011; Carrero**	9 European countries	Retrospective cohort	ERA-EDTA	108,963	1994-2004	Hemodialysis/ Peritoneal dialysis	60.9	17.7	20.9
**2011; Chidambaram**	Canada	Retrospective cohort	Ontario Health Insurance Plan	5,162	1995-2005	Peritoneal dialysis	56.2	100	52.5
**2011; Bastos**	Brazil	Prospective cohort	BRAZPD	1,952	2004-2007	Peritoneal dialysis	46	100	41
**2011; Machado**	Brazil	Prospective cohort	National Database for Renal Replacement Therapy	14,111	2000-2004	Hemodialysis/ Peritoneal dialysis	57.1	11.1	17.5
**2011; Hall**	USA	Retrospective cohort	USRDS	503,090	1995-2006	Hemodialysis/ Peritoneal dialysis, age <65 years	53.4	N/A	51.9

**2010; Chung**	South Korea	Retrospective cohort	Study data	213	1994-2005	Peritoneal dialysis	55.9	100	55.4
**2010; Huang**	Taiwan	Retrospective cohort	Study data	200	2001-2007	Hemodialysis/ Peritoneal dialysis	53	51	N/A
**2010; Sood**	Canada	Prospective cohort	Manitoba Renal Program	727	1997-2007	Peritoneal dialysis	55.3	100	44.6
**2010; Sandhu**	USA	Retrospective cohort	USRDS	3,396	1990-1999	ESKD	54.2	N/A	50.4
**2010; Udayaraj**	United Kingdom	Retrospective cohort	UK Renal Registry	11,299	1997-2004	Hemodialysis/ Peritoneal dialysis, age <70 years	61.3	N/A	N/A
**2010; Ng**	United Kingdom	Prospective cohort	Study data	555	1995-2006	Living/deceased-donor transplantation	53.4	-	N/A
**2010; Ravanan**	United Kingdom	Prospective cohort	UK Renal Registry	7,863	2003-2005	Hemodialysis/ Peritoneal dialysis	60.4	N/A	22.3
**2009; Roderick**	United Kingdom	Prospective cohort	UK Renal Registry	30,561	1997-2006	Hemodialysis/ Peritoneal dialysis	62	25.8	18.3
**2009; de Mutsert**	Netherlands	Prospective cohort	NECOSAD-II	700	1997-2007	Hemodialysis/ Peritoneal dialysis	60.5	35.1	15
**2009; Eisenstein**	USA	Retrospective cohort	USRDS	186,424	1996-1999	Hemodialysis	52.4	100	42.2
**2009; Yeates**	USA/ United Kingdom/ Australia/ New Zealand	Retrospective cohort	USRDS, CORR, ANZDATA	312,507	1995-2005	Hemodialysis/ Peritoneal dialysis	57.6	N/A	N/A
**2009; Feyssa**	USA	Retrospective cohort	Study data	2,130	1995-2003	Living/deceased-donor transplantation	60.8	-	38.8
**2009; Patzer**	USA	Retrospective cohort	United Network for Organ Sharing	35,346	1998-2002	Hemodialysis	50.1	0	44.5
**2008; Wolf**	USA	Prospective cohort	ArMORR study	9,303	2004-2005	Hemodialysis	54.7	0	42.7
**2008; Sanabria**	Colombia	Retrospective cohort	Dialysis outcomes in Colombia study	923	2001-2003	Hemodialysis/ Peritoneal dialysis	56.9	47.3	40.8
**2008; Hall**	USA	Retrospective cohort	USRDS	22,152	1995-2003	Hemodialysis/ Peritoneal dialysis	54.6	9.4	41.6
**2008; Schaeffner**	USA	Prospective cohort	Dialysis Morbidity and Mortality Study Wave 2	3,245	1996-1997	Hemodialysis/ Peritoneal dialysis	53.3	N/A	49.7
**2007; Gill**	USA	Retrospective cohort	USRDS	509,391	1995-2004	ESKD, age ≤70 years	54.7	N/A	48.1
**2006; Robinson**	USA	Prospective cohort	DOPPS study	6,677	1996-2001	Hemodialysis	54.6	0	40.4

**2006; Caskey**	United Kingdom	Retrospective cohort	UK Renal Registry	10,392	1997-2004	Hemodialysis/ Peritoneal dialysis	62.1	34.8	17.3
**2006; Hemmelgarn**	Canada	Retrospective cohort	CORR	10,338	1990-2000	Hemodialysis/ Peritoneal dialysis	60.4	29.3	6.9
**2006; Bayat**	France	Retrospective cohort	CRISTAL Registry	1,495	1997-2003	ESKD	58.6	15.8	34.1
**2005; Chung**	South Korea, Sweden	Retrospective cohort	Study data	238	1994-2000	Peritoneal dialysis	57	100	49
**2005; Mircescu**	Romania	Retrospective cohort	Study data	412	1989-2003	Peritoneal dialysis	50.2	100	22.6
**2005; Kutner**	USA	Retrospective cohort	DMMS Wave 2	868	1993-1994	Hemodialysis/ Peritoneal dialysis	52.8	47.6	44.3
**2005; Press**	USA	Retrospective cohort	United Network for Organ Sharing	4,471	1990	Living/deceased-donor transplantation	58.9	-	21.2
**2005; Pallet**	France	Retrospective cohort	Donnees Informatisees VAlidees En Transplantation	1,092	1987-2003	Living/deceased-donor transplantation	59.2	-	N/A
**2005; Chakkera**	USA	Retrospective cohort	Veterans Affairs Kidney Transplant, USRDS	79,361	1991-2001	Living/deceased-donor transplantation	60.3	-	33.8
**2004; Prasad**	United Kingdom	Prospective cohort	Study data	474	1996-2001	Hemodialysis/ Peritoneal dialysis	61.9	9.1	32.7
**2004; Jager**	Netherlands	Prospective cohort	NECOSAD study	1,347	1997-2001	Hemodialysis/ Peritoneal dialysis	61	34.6	15
**2004; Yeates**	Canada	Retrospective cohort	CORR	25,632	1990-1998	Hemodialysis/ Peritoneal dialysis	59.6	N/A	29.1
**2004; Sequist**	USA	Retrospective cohort	Intermountain ESRD Network #15	1,335	1994-1995	Hemodialysis/ Peritoneal dialysis	52.1	21	54.5
**2004; Villar**	France	Retrospective cohort	Study data	549	1995-1998	ESKD	63.2	20.2	31.9
**2003; Wang**	Canada	Retrospective cohort	Study data	116	1996-1999	Peritoneal dialysis	64.7	100	36.2
**2003; Cass**	Australia	Retrospective cohort	ANZDATA	5,322	1993-1998	Hemodialysis/ Peritoneal dialysis, age <65 years	56.7	N/A	22.1
**2003; Oniscu**	Scotland	Prospective cohort	Scottish Renal Registry	4,523	1989-1999	Hemodialysis/ Peritoneal dialysis	58.3	29.6	16.3
**2002; Reddan**	USA	Retrospective cohort	ESRD Core Indicators/ CPM Project	8,154	1999	Hemodialysis	53	0	40
**2002; Pisoni**	International	Prospective cohort	DOPPS study	2,073	1996-2000	Hemodialysis	55	0	36
**2002; Iseki**	Japan	Prospective cohort	Okinawa Dialysis Study	1,243	1991-2001	Hemodialysis	58.3	0	17.8

**2002; Stack**	USA	Retrospective cohort	USRDS	3,793	1996-1997	Hemodialysis/ Peritoneal dialysis	53	48.4	43
**2001; Winkelmayer**	USA	Retrospective cohort	New Jersey Medicare/Medicaid claims	3,014	1990-1996	Hemodialysis/ Peritoneal dialysis	56.2	22.2	29
**2000; Stehman-Breen**	USA	Prospective cohort	USRDS	1,449	1993-1994	Hemodialysis	53	0	51
**2000; Allon**	USA	Prospective cohort	HEMO study	1,824	1995-1999	Hemodialysis, age <80 years	48.6	0	43.6
**2000; Schaubel**	Canada	Retrospective cohort	CORR	33,589	1981-1996	Hemodialysis/ Peritoneal dialysis	59.9	35.2	23.8
**2000; Garg**	USA	Prospective cohort	United Network for Organ Sharing	7,594	1986-1993	ESKD	51.6	N/A	34.8
**1999; Chandna**	United Kingdom	Retrospective cohort	Study data	292	1992-1996	Hemodialysis/ Peritoneal dialysis	66	N/A	20
**1996; Bleyer**	USA	Retrospective cohort	Study data	550	1990-1993	Hemodialysis/ Peritoneal dialysis	N/A	21.8	44.2
**1996; Medina**	USA	Prospective cohort	32 centers	584	1988-1991	Hemodialysis, diabetic nephropathy	42.1	0	100
**1995; Barker-Cummings**	USA	Retrospective cohort	ESRD Network	10,726	1989-1991	Hemodialysis/ Peritoneal dialysis	49.6	21.8	32.9
**1994; Cowie**	USA	Retrospective cohort	Michigan Kidney Registry	594	1974-1983	Hemodialysis, age <65 years, diabetic nephropathy	54.4	0	100
**1994; Puqh**	USA	Retrospective cohort	Texas Kidney Health Program	11,978	1975-1985	Hemodialysis/ Peritoneal dialysis, age ≥20 years	N/A	6.1	27.2
**1992; Soucie**	USA	Prospective cohort	Study data	8,315	1989-1990	Hemodialysis	47	0	19.6

N/A: not applicable; ESKD: end-stage kidney disease; USRDS: United States Renal Data System; CORR: Canadian Organ Replacement Register; ANZDATA: Australia and New Zealand Dialysis and Transplant Register; BRAZPD: Brazilian Peritoneal Dialysis Multicenter Study; DMMS: Dialysis Morbidity and Mortality Study; DOPPS: Dialysis Outcomes and Practice Patterns Study; REIN: Renal Epidemiology and Information Network; SSOP: Socioeconomic Status on the Outcome of Peritoneal Dialysis; PKDC: Polokwane Kidney and Dialysis Centre; ATTOM: Access to Transplantation and Transplant Outcome Measures; NECOSAD: Netherlands Cooperative Study on the Adequacy of Dialysis; CPM: Clinical Performance Measures; ERDA-EDTA: European Renal Association-European Dialysis and Transplant Association; ATTOM: Access to Transplantation and Transplant Outcome Measures; ArMORR: Accelerated Mortality on Renal Replacement; CRIC: Chronic Renal Insufficiency Cohort; HEMO: Hemodialysis

Suppl. Table S3. Definitions of income, education and occupation in the included studies

Study	Income	Education	Occupation
**1992; Soucie**	-	>12 vs. <8 years	-
**1995; Barker-Cummings**	-	>12 vs. <8 years	Employed vs. unemployed
**2000; Stehman-Breen**	-	College vs. high school graduate	Employed vs. unemployed
**2000; Allon**	≥$7,500 vs. <$7,500 per year	College vs. high school graduate	-
**2003; Oniscu**	Area-level deprivation index		
**2004; Jager**	-	High vs. low	Employed vs. unemployed
**2005; Kutner**	-	College vs. high school graduate	Employed vs. unemployed
**2006; Caskey**	Area-level Townsend Index deprivation score		
**2007; Gill**	ZIP-code median household income	-	-
**2008; Sanabria**	Highest vs. lowest	Postgraduate vs. illiterate	-
**2008; Schaeffner**	-	College graduate vs. <high school	-
**2009; Eisenstein**	ZIP-code median household income	-	-
**2009; Feyssa**	-	-	Employed vs. unemployed
**2010; Huang**	-	High vs. low	Employed vs. unemployed
**2010; Sandhu**	Social adaptability index	-	-
**2011; Bastos**	Family income	-	-
**2011; Machado**	Area-level Human Development Index		
**2012; Marinovich**	Income vs. no income	-	-
**2012; Xu**	>$6,320 vs. <$3,160	College vs. primary school	-
**2012; Huang**	-	≥High-school vs. <high-school	-
**2012; Lockridge**	-	>High-school vs. <high-school	-
**2012; Neri**	-	-	Employed vs. unemployed
**2012; Udayaraj**	Area-level Townsend Index	-	-
**2012; Goldfarb-Rumyantzev**	-	College graduate vs. <high-school	-
**2012; Garg**	Social adaptability index	-	-

**2013; Kimmel**	ZIP-code median household income	-	-
**2014; Goldfarb-Rumyantzev**	-	-	Full time vs. unemployed
**2014; Grace**	Area-level Index of Relative Socio-Economic Advantage and Disadvantage		
**2014; Yang**	-	>High-school vs. primary school	-
**2014; Teixeira**	-	>8 vs. ≤8 years	-
**2015; Nee**	ZIP-code median household income	-	-
**2015; Wang**	-	≥ Primary school vs. illiterate	-
**2015; Ward**	Area-level 2011 Pobal Haase Pratschke Deprivation Index		
**2015b; Ward**	Area-level 2011 Pobal Haase Pratschke Deprivation Index		
**2016; Shabankhani**	-	-	Employed vs. unemployed
**2016; Kihal-Talantikite**	-	-	Employed vs. unemployed
**2016; Molmenti**	-	College graduate vs. <high-school	-
**2017; Wimink**	Area-level deprivation index		
**2017; Gulcan**	-	-	Employed vs. unemployed
**2017; Kim**	-	≥High-school vs. <middle-school	-
**2017; Chan**	Area-level Index of Relative Socio-Economic Advantage and Disadvantage		
**2017; Imanishi**	-	University graduate vs. <high-school	Employed vs. unemployed
**2017; Zhang**	Disposable income	>12 vs. ≤9 years	-
**2017; Choi**	High vs. medical aid	-	-
**2017; Taber**	-	College graduate vs. <high-school	-
**2018; Lin**	-	-	Employed vs. unemployed
**2018; Krishnashamy**	Area-level Index of Relative Socio-Economic Advantage and Disadvantage		
**2018; Keddis**	Above vs. below poverty	≥High-school vs. <high-school	
**2018; Peng**	-	-	Employed vs. unemployed
**2018b; Zhang**	Individual disposable income	>High school vs. <high school	-
**2018; Chatelet**	European Deprivation Index		
**2019; Nee**	ZIP-code median household income	-	-

**2019; Ke**	Area-level household income	-	-
**2019; King**	-	>High-school vs. ≤high-school	-
**2019; Naylor**	Area-level median household income	-	-
**2020; Krishnashamy**	Area-level Index of Relative Socio-Economic Advantage and Disadvantage		
**2020; Yolgosteren**	-	College vs. primary school	-
**2020; Hayat**	Area-level Zealand Deprivation Index		
**2020; Pruthi**	-	≥High-school vs. <high-school	Full/part time vs. unemployed
**2020; Ng**	Household ≥$25,000 vs. <$25,000 per year	≥High-school vs. <high-school	-
**2020; Park**	-	-	Employed vs. unemployed
**2021; Barth**	Active vs. inactive	-	-
**2021; Wesselman**	Household ≥$50,000 vs. <$50,000 per year	-	-
**2021; Belyaev**	Area-level Zealand Deprivation Index		
**2021; Rita**	-	>High-school vs. ≤high-school	-
**2021; Park**	Highest income vs. aided	-	-

Included studies

1. Ahearn P, Johansen KL, McCulloch CE, Grimes BA, Ku E. Sex Disparities in Risk of Mortality Among Children With ESRD. Am J Kidney Dis. 2019;73(2):156-162. doi:10.1053/J.AJKD.2018.07.019.

2. Allon M, Ornt DB, Schwab SJ, et al. Factors associated with the prevalence of arteriovenous fistulas in hemodialysis patients in the HEMO study. Hemodialysis (HEMO) Study Group. Kidney Int. 2000;58(5):2178-2185. doi:10.1111/J.1523-1755.2000.00391.X.

3. Arce CM, Goldstein BA, Mitani AA, Lenihan CR, Winkelmayer WC. Differences in access to kidney transplantation between Hispanic and non-Hispanic whites by geographic location in the United States. Clin J Am Soc Nephrol. 2013;8(12):2149-2157. doi:10.2215/CJN.01560213.

4. Arce CM, Lenihan CR, Montez-Rath ME, Winkelmayer WC. Comparison of longer-term outcomes after kidney transplantation between Hispanic and non-Hispanic whites in the United States. Am J Transplant. 2015;15(2):499-507. doi:10.1111/AJT.13043.

5. Arhuidese IJ, AJi EA, Muhammad R, Dhaliwal J, Shukla AJ, Malas MB. Racial differences in utilization and outcomes of hemodialysis access in the Unites States. J Vasc Surg. 2020;71(5):1664-1673. doi:10.1016/J.JVS.2019.07.092.

6. Arya S, Melanson TA, George EL, et al. Racial and Sex Disparities in Catheter Use and Dialysis Access in the United States Medicare Population. J Am Soc Nephrol. 2020;31(3):625-636. doi:10.1681/ASN.2019030274.

7. Barker Cummings C, Mcclellan W, Soucie JM, Krisher J. Ethnic Differences in the Use of Peritoneal Dialysis as Initial Treatment for End-Stage Renal Disease. JAMA. 1995;274(23):1858-1862. doi:10.1001/JAMA.1995.03530230044028.

8. Barth A, Szőllősi GJ, Nemes B. Factors Affecting Access to the Kidney Transplant Waiting List in Eastern Hungary. Transplant Proc. 2021;53(5):1418-1422. doi:10.1016/J.TRANSPROCEED.2021.01.044.

9. Bastos K de A, Qureshi AR, Lopes AA, et al. Family income and survival in Brazilian Peritoneal Dialysis Multicenter Study Patients (BRAZPD): time to revisit a myth? Clin J Am Soc Nephrol. 2011;6(7):1676-1683. doi:10.2215/CJN.09041010.

10. Bayat S, Frimat L, Thilly N, Loos C, Briançon S, Kessler M. Medical and non-medical determinants of access to renal transplant waiting list in a French community-based network of care. Nephrol Dial Transplant. 2006;21(10):2900-2907. doi:10.1093/NDT/GFL329.

11. Belyaev AM, Henry L, Dittmer I, MuthuKumaraswamy C, Davies CE, Bergin CJ. Socioeconomic inequality: accessibility and outcomes after renal transplantation in New Zealand. ANZ J Surg. 2021;91(12):2656-2662. doi:10.1111/ANS.16997.

12. Van Den Beukel TO, Hommel K, Kamper AL, et al. Differences in survival on chronic dialysis treatment between ethnic groups in Denmark: a population-wide, national cohort study. Nephrol Dial Transplant. 2016;31(7):1160-1167. doi:10.1093/NDT/GFV359.

13. Van Den Beukel TO, Verduijn M, Le Cessie S, et al. The role of psychosocial factors in ethnic differences in survival on dialysis in the Netherlands. Nephrol Dial Transplant. 2012;27(6):2472-2479. doi:10.1093/NDT/GFR631.

14. Bleyer AJ, Tell GS, Evans GW, Ettinger WH, Burkart JM. Survival of patients undergoing renal replacement therapy in one center with special emphasis on racial differences. Am J Kidney Dis. 1996;28(1):72-81. doi:10.1016/S0272-6386(96)90133-X.

15. Burrows NR, Cho P, Bullard KMK, Narva AS, Eggers PW. Survival on dialysis among American Indians and Alaska Natives with diabetes in the United States, 1995-2010. Am J Public Health. 2014;104 Suppl 3(Suppl 3). doi:10.2105/AJPH.2014.301942.

16. Carrero JJ, de Jager DJ, Verduijn M, et al. Cardiovascular and noncardiovascular mortality among men and women starting dialysis. Clin J Am Soc Nephrol. 2011;6(7):1722-1730. doi:10.2215/CJN.11331210.

17. Caskey FJ, Roderick P, Steenkamp R, et al. Social deprivation and survival on renal replacement therapy in England and Wales. Kidney Int. 2006;70(12):2134-2140. doi:10.1038/SJ.KI.5001999.

18. Cass A, Cunningham J, Snelling P, Wang Z, Hoy W. Renal transplantation for Indigenous Australians: identifying the barriers to equitable access. Ethn Health. 2003;8(2):111-119. doi:10.1080/13557850303562.

19. Chakkera HA, O'Hare AM, Johansen KL, et al. Influence of race on kidney transplant outcomes within and outside the Department of Veterans Affairs. J Am Soc Nephrol. 2005;16(1):269-277. doi:10.1681/ASN.2004040333.

20. Chan S, Cho Y, Koh YH, et al. Association of Socio-Economic Position with Technique Failure and Mortality in Australian Non-Indigenous Peritoneal Dialysis Patients. Perit Dial Int. 2017;37(4):397-406. doi:10.3747/PDI.2016.00209.

21. Chandna SM, Schulz J, Lawrence C, Greenwood RN, Farrington K. Is there a rationale for rationing chronic dialysis? A hospital based cohort study of factors affecting survival and morbidity. BMJ. 1999;318(7178):217-223. doi:10.1136/BMJ.318.7178.217.

22. Chang TI, Oh HJ, Kang EW, et al. A low serum bicarbonate concentration as a risk factor for mortality in peritoneal dialysis patients. PLoS One. 2013;8(12). doi:10.1371/JOURNAL.PONE.0082912.

23. Châtelet V, Bayat-Makoei S, Vigneau C, Launoy G, Lobbedez T. Renal transplantation outcome and social deprivation in the French healthcare system: a cohort study using the European Deprivation Index. Transpl Int. 2018;31(10):1089-1098. doi:10.1111/TRI.13161.

24. Chidambaram M, Bargman JM, Quinn RR, Austin PC, Hux JE, Laupacis A. Patient and physician predictors of peritoneal dialysis technique failure: a population based, retrospective cohort study. Perit Dial Int. 2011;31(5):565-573. doi:10.3747/PDI.2010.00096.

25. Choi Y, Shin J, Park JT, Cho KH, Park EC, Kim TH. Disparities in Kidney Transplantation Access among Korean Patients Initiating Dialysis: A Population-Based Cohort Study Using National Health Insurance Data (2003-2013). Am J Nephrol. 2017;45(1):32-39. doi:10.1159/000450979.

26. Chung SH, Han DC, Noh H, et al. Risk factors for mortality in diabetic peritoneal dialysis patients. Nephrol Dial Transplant. 2010;25(11):3742-3748. doi:10.1093/NDT/GFQ233.

27. Chung SH, Heimbürger O, Lindholm B, Lee HB. Peritoneal dialysis patient survival: a comparison between a Swedish and a Korean centre. Nephrol Dial Transplant. 2005;20(6):1207-1213. doi:10.1093/NDT/GFH772.

28. Cirillo M, Palladino R, Ciacci C, et al. Kidney Replacement Treatment in South-Western Italy (Campania): Population-Based Study on Gender and Residence Inequalities in Health Care Access. J Clin Med. 2021;10(3):1-11. doi:10.3390/JCM10030449.

29. Cole N, Bedford M, Cai A, Jones C, Cairns H, Jayawardene S. Black ethnicity predicts better survival on dialysis despite greater deprivation and co-morbidity: a UK study. Clin Nephrol. 2014;82(2):77-82. doi:10.5414/CN108247.

30. Copeland TP, Hye RJ, Lawrence PF, Woo K. Association of Race and Ethnicity with Vascular Access Type Selection and Outcomes. Ann Vasc Surg. 2020;62:142-147. doi:10.1016/J.AVSG.2019.08.068.

31. Couchoud C, Bayat S, Villar E, Jacquelinet C, Ecochard R. A new approach for measuring gender disparity in access to renal transplantation waiting lists. Transplantation. 2012;94(5):513-519. doi:10.1097/TP.0B013E31825D156A.

32. Cowie CC, Port FK, Rust KF, Harris MI. Differences in survival between black and white patients with diabetic end-stage renal disease. Diabetes Care. 1994;17(7):681-687. doi:10.2337/DIACARE.17.7.681.

33. Eisenstein EL, Sun JL, Anstrom KJ, et al. Do income level and race influence survival in patients receiving hemodialysis? Am J Med. 2009;122(2):170-180. doi:10.1016/J.AMJMED.2008.08.025.

34. Fernandes NMDS, Hoekstra T, Van Den Beukel TO, et al. Association of ethnicity and survival in peritoneal dialysis: a cohort study of incident patients in Brazil. Am J Kidney Dis. 2013;62(1):89-96. doi:10.1053/J.AJKD.2013.02.364.

35. Feyssa E, Jones-Burton C, Ellison G, Philosophe B, Howell C. Racial/ethnic disparity in kidney transplantation outcomes: influence of donor and recipient characteristics. J Natl Med Assoc. 2009;101(2):111-115. doi:10.1016/S0027-9684(15)30822-1.

36. Garg J, Karim M, Tang H, et al. Social adaptability index predicts kidney transplant outcome: a single-center retrospective analysis. Nephrol Dial Transplant. 2012;27(3):1239-1245. doi:10.1093/NDT/GFR445.

37. Garg PP, Furth SL, Fivush BA, Powe NR. Impact of gender on access to the renal transplant waiting list for pediatric and adult patients. J Am Soc Nephrol. 2000;11(5):958-964. doi:10.1681/ASN.V115958.

38. Gill JS, Hussain S, Rose C, Hariharan S, Tonelli M. Access to kidney transplantation among patients insured by the United States Department of Veterans Affairs. J Am Soc Nephrol. 2007;18(9):2592-2599. doi:10.1681/ASN.2007010050.

39. Gill JS, Rose C, Joffres Y, Landsberg D, Gill J. Variation in Dialysis Exposure Prior to Nonpreemptive Living Donor Kidney Transplantation in the United States and Its Association With Allograft Outcomes. Am J Kidney Dis. 2018;71(5):636-647. doi:10.1053/J.AJKD.2017.11.012.

40. Goldfarb-Rumyantzev AS, Sandhu GS, Barenbaum A, et al. Education is associated with reduction in racial disparities in kidney transplant outcome. Clin Transplant. 2012;26(6):891-899. doi:10.1111/J.1399-0012.2012.01662.X.

41. Goldfarb-Rumyantzev AS, Syed W, Patibandla BK, et al. Geographic disparities in arteriovenous fistula placement in patients approaching hemodialysis in the United States. Hemodial Int. 2014;18(3):686-694. doi:10.1111/HDI.12141.

42. Grace BS, Clayton PA, Gray NA, McDonald SP. Socioeconomic differences in the uptake of home dialysis. Clin J Am Soc Nephrol. 2014;9(5):929-935. doi:10.2215/CJN.08770813.

43. Gulcan E, Kidir V, Keles M, Cankaya E, Uyanik A, Saatci F. Factors affecting patient survival and technical survival in patients undergoing peritoneal dialysis. Int J Clin Exp Med. 2017;10(1):1004-1014.

44. Hall YN, Choi AI, Xu P, O'Hare AM, Chertow GM. Racial ethnic differences in rates and determinants of deceased donor kidney transplantation. J Am Soc Nephrol. 2011;22(4):743-751. doi:10.1681/ASN.2010080819.

45. Hall YN, O'Hare AM, Young BA, Boyko EJ, Chertow GM. Neighborhood poverty and kidney transplantation among US Asians and Pacific Islanders with end-stage renal disease. Am J Transplant. 2008;8(11):2402-2409. doi:10.1111/J.1600-6143.2008.02413.X.

46. Hayat A, Saweirs W. Predictors of technique failure and mortality on peritoneal dialysis: An analysis of New Zealand peritoneal dialysis registry data. Nephrology (Carlton). 2021;26(6):530-540. doi:10.1111/NEP.13837.

47. Hemmelgarn BR, Chou S, Wiebe N, et al. Differences in use of peritoneal dialysis and survival among East Asian, Indo Asian, and white ESRD patients in Canada. Am J Kidney Dis. 2006;48(6):964-971. doi:10.1053/J.AJKD.2006.08.021.

48. Howson P, Irish AB, D'Orsogna L, et al. Allograft and Patient Outcomes Between Indigenous and Nonindigenous Kidney Transplant Recipients. Transplantation. 2020;104(4):847-855. doi:10.1097/TP.0000000000002891.

49. Huang HC, Wang JY, Chang CC, Chiu PF, Chiang MC, Yang Y. Nonclinical factors associated with treatment with peritoneal dialysis in ESRD patients in Taiwan. Perit Dial Int. 2010;30(6):638-643. doi:10.3747/PDI.2009.00112.

50. Huang WH, Lin JL, Lin-Tan DT, Chen KH, Hsu CW, Yen TH. Education level is associated with mortality in male patients undergoing maintenance hemodialysis. Blood Purif. 2013;35(4):316-326. doi:10.1159/000351613.

51. Huria T, Palmer S, Beckert L, Williman J, Pitama S. Inequity in dialysis related practices and outcomes in Aotearoa/New Zealand: a Kaupapa Māori analysis. Int J Equity Health. 2018;17(1). doi:10.1186/S12939-018-0737-9.

52. Ilori TO, Adedinsewo DA, Odewole O, et al. Racial and Ethnic Disparities in Graft and Recipient Survival in Elderly Kidney Transplant Recipients. J Am Geriatr Soc. 2015;63(12):2485-2493. doi:10.1111/JGS.13845.

53. Imanishi Y, Fukuma S, Karaboyas A, et al. Associations of employment status and educational levels with mortality and hospitalization in the dialysis outcomes and practice patterns study in Japan. PLoS One. 2017;12(3). doi:10.1371/JOURNAL.PONE.0170731.

54. Iseki K, Yamazato M, Tozawa M, Takishita S. Hypocholesterolemia is a significant predictor of death in a cohort of chronic hemodialysis patients. Kidney Int. 2002;61(5):1887-1893. doi:10.1046/J.1523-1755.2002.00324.X.

55. Isla RAT, Ameh OI, Mapiye D, et al. Baseline Predictors of Mortality among Predominantly Rural-Dwelling End-Stage Renal Disease Patients on Chronic Dialysis Therapies in Limpopo, South Africa. PLoS One. 2016;11(6). doi:10.1371/JOURNAL.PONE.0156642.

56. Jager KJ, Korevaar JC, Dekker FW, Krediet RT, Boeschoten EW. The effect of contraindications and patient preference on dialysis modality selection in ESRD patients in The Netherlands. Am J Kidney Dis. 2004;43(5):891-899. doi:10.1053/J.AJKD.2003.12.051.

57. Ke C, Kim SJ, Shah BR, et al. Impact of Socioeconomic Status on Incidence of End-Stage Renal Disease and Mortality After Dialysis in Adults With Diabetes. Can J diabetes. 2019;43(7):483-489.e4. doi:10.1016/J.JCJD.2019.04.006.

58. Keddis MT, Sharma A, Ilyas M, et al. Transplant center assessment of the inequity in the kidney transplant process and outcomes for the Indigenous American patients. PLoS One. 2018;13(11). doi:10.1371/JOURNAL.PONE.0207819.

59. Kihal-Talantikite W, Vigneau C, Deguen S, Siebert M, Couchoud C, Bayat S. Influence of Socio-Economic Inequalities on Access to Renal Transplantation and Survival of Patients with End-Stage Renal Disease. PLoS One. 2016;11(4). doi:10.1371/JOURNAL.PONE.0153431.

60. Kim HJ, Lee J, Park M, et al. Lower Education Level Is a Risk Factor for Peritonitis and Technique Failure but Not a Risk for Overall Mortality in Peritoneal Dialysis under Comprehensive Training System. PLoS One. 2017;12(1). doi:10.1371/JOURNAL.PONE.0169063.

61. Kim HW, Kim SH, Kim YO, et al. The Impact of Timing of Dialysis Initiation on Mortality in Patients with Peritoneal Dialysis. Perit Dial Int. 2015;35(7):703-711. doi:10.3747/PDI.2013.00328.

62. Kimmel PL, Fwu CW, Eggers PW. Segregation, income disparities, and survival in hemodialysis patients. J Am Soc Nephrol. 2013;24(2):293-301. doi:10.1681/ASN.2012070659.

63. King KL, Husain SA, Jin Z, Brennan C, Mohan S. Trends in Disparities in Preemptive Kidney Transplantation in the United States. Clin J Am Soc Nephrol. 2019;14(10):1500-1511. doi:10.2215/CJN.03140319.

64. Krishnasamy R, Gray NA. Low socio-economic status adversely effects dialysis survival in Australia. Nephrology (Carlton). 2018;23(5):453-460. doi:10.1111/NEP.13053.

65. Krishnasamy R, Jegatheesan D, Lawton P, Gray NA. Socioeconomic status and dialysis quality of care. Nephrology (Carlton). 2020;25(5):421-428. doi:10.1111/NEP.13629.

66. Ku E, Yang W, McCulloch CE, et al. Race and Mortality in CKD and Dialysis: Findings From the Chronic Renal Insufficiency Cohort (CRIC) Study. Am J Kidney Dis. 2020;75(3):394-403. doi:10.1053/J.AJKD.2019.08.011.

67. Kutner NG, Zhang R, Barnhart H, Collins AJ. Health status and quality of life reported by incident patients after 1 year on haemodialysis or peritoneal dialysis. Nephrol Dial Transplant. 2005;20(10):2159-2167. doi:10.1093/NDT/GFH973.

68. Lawton PD, Cunningham J, Zhao Y, et al. Survival of Indigenous Australians receiving renal replacement therapy: closing the gap? Med J Aust. 2015;202(4):200-205. doi:10.5694/MJA14.00664.

69. Lin E, Mell MW, Winkelmayer WC, Erickson KF. Health insurance in the first 3 months of hemodialysis and early vascular access. Clin J Am Soc Nephrol. 2018;13(12):1866-1875. doi:10.2215/CJN.06660518/-/DCSUPPLEMENTAL.

70. Liu A, Woodside KJ, Augustine JJ, Sarabu N. Racial disparity in kidney transplant survival relates to late rejection and is independent of steroid withdrawal. Clin Transplant. 2018;32(9). doi:10.1111/CTR.13381.

71. Liu X, Huang R, Wu H, et al. Patient characteristics and risk factors of early and late death in incident peritoneal dialysis patients. Sci Rep. 2016;6. doi:10.1038/SREP32359.

72. Lockridge R, Ting G, Kjellstrand CM. Superior patient and technique survival with very high standard Kt/V in quotidian home hemodialysis. Hemodial Int. 2012;16(3):351-362. doi:10.1111/J.1542-4758.2012.00696.X.

73. Lopez-Vargas PA, Craig JC, Gallagher MP, et al. Barriers to timely arteriovenous fistula creation: a study of providers and patients. Am J Kidney Dis. 2011;57(6):873-882. doi:10.1053/J.AJKD.2010.12.020.

74. Machado EL, Caiaffa WT, César CC, et al. Iniquities in the access to renal transplant for patients with end-stage chronic renal disease in Brazil. Cad Saude Publica. 2011;27 Suppl 2(SUPPL.2):284-297. doi:10.1590/S0102-311X2011001400015.

75. MacRae JM, Clarke A, Ahmed SB, et al. Sex differences in the vascular access of hemodialysis patients: a cohort study. Clin Kidney J. 2021;14(5):1412-1418. doi:10.1093/CKJ/SFAA132.

76. Marinovich S, Lavorato C, Rosa-Diez G, Bisigniano L, Fernández V, Hansen-Krogh D. The lack of income is associated with reduced survival in chronic haemodialysis. Nefrologia. 2012;32(1):79-88. doi:10.3265/NEFROLOGIA.PRE2011.NOV.11110.

77. Matsuoka L, Alicuben E, Woo K, et al. Kidney transplantation in the Hispanic population. Clin Transplant. 2016;30(2):118-123. doi:10.1111/CTR.12662.

78. McGill RL, Lacson E. Sex, race, and hemodialysis vascular access processes. J Vasc Access. 2017;18(2):132-138. doi:10.5301/JVA.5000657.

79. McKercher C, Jose MD, Grace B, Clayton PA, Walter M. Gender differences in the dialysis treatment of Indigenous and non-Indigenous Australians. Aust N Z J Public Health. 2017;41(1):15-20. doi:10.1111/1753-6405.12621.

80. Medina RA, Pugh JA, Monterrosa A, Cornell J. Minority advantage in diabetic end-stage renal disease survival on hemodialysis: due to different proportions of diabetic type? Am J Kidney Dis. 1996;28(2):226-234. doi:10.1016/S0272-6386(96)90306-6.

81. Melk A, Schmidt BMW, Geyer S, Epping J. Sex disparities in dialysis initiation, access to waitlist, transplantation and transplant outcome in German patients with renal disease-A population based analysis. PLoS One. 2020;15(11). doi:10.1371/JOURNAL.PONE.0241556.

82. Mircescu G, Garneata L, Florea L, et al. The success story of peritoneal dialysis in Romania: Analysis of differences in mortality by dialysis modality and influence of risk factors in a national cohort. Perit Dial Int. 2006;26(2):266-275. doi:10.1177/089686080602600224.

83. Molmenti EP, Alex A, Rosen L, et al. Recipient Criteria Predictive of Graft Failure in Kidney Transplantation. Int J Angiol. 2016;25(1):29-38. doi:10.1055/S-0035-1563605.

84. de Moraes TP, Figueiredo AE, de Campos LG, Olandoski M, Barretti P, Pecoits-Filho R. Characterization of the BRAZPD II cohort and description of trends in peritoneal dialysis outcome across time periods. Perit Dial Int. 2014;34(7):714-723. doi:10.3747/PDI.2013.00282.

85. Mucsi I, Bansal A, Famure O, et al. Ethnic Background Is a Potential Barrier to Living Donor Kidney Transplantation in Canada: A Single-Center Retrospective Cohort Study. Transplantation. 2017;101(4):e142-e151. doi:10.1097/TP.0000000000001658.

86. Murphy KA, Jackson JW, Purnell TS, et al. Association of Socioeconomic Status and Comorbidities with Racial Disparities during Kidney Transplant Evaluation. Clin J Am Soc Nephrol. 2020;15(6):843-851. doi:10.2215/CJN.12541019.

87. de Mutsert R, Grootendorst DC, Indemans F, Boeschoten EW, Krediet RT, Dekker FW. Association between serum albumin and mortality in dialysis patients is partly explained by inflammation, and not by malnutrition. J Ren Nutr. 2009;19(2):127-135. doi:10.1053/J.JRN.2008.08.003.

88. Myaskovsky L, Kendall K, Li X, et al. Unexpected Race and Ethnicity Differences in the US National Veterans Affairs Kidney Transplant Program. Transplantation. 2019;103(12):2701-2714. doi:10.1097/TP.0000000000002905.

89. Naylor KL, Knoll GA, Shariff SZ, et al. Socioeconomic Status and Kidney Transplant Outcomes in a Universal Healthcare System: A Population-based Cohort Study. Transplantation. 2019;103(5):1024-1035. doi:10.1097/TP.0000000000002383.

90. Nee R, Moon DS, Jindal RM, et al. Impact of Poverty and Health Care Insurance on Arteriovenous Fistula Use among Incident Hemodialysis Patients. Am J Nephrol. 2015;42(4):328-336. doi:10.1159/000441804.

91. Nee R, Thurlow JS, Norris KC, et al. Association of Race and Poverty With Mortality Among Nursing Home Residents on Maintenance Dialysis. J Am Med Dir Assoc. 2019;20(7):904-910. doi:10.1016/J.JAMDA.2019.02.013.

92. Neri L, Gallieni M, Rocca Rey LA, Bertoli SV, Andreucci V, Brancaccio D. Inequalities in transplant waiting list activation across Italian dialysis centers. Am J Nephrol. 2013;37(6):575-585. doi:10.1159/000351334.

93. Ng FL, Holt DW, Chang RWS, MacPhee IAM. Black renal transplant recipients have poorer long-term graft survival than CYP3A5 expressers from other ethnic groups. Nephrol Dial Transplant. 2010;25(2):628-634. doi:10.1093/NDT/GFP530.

94. Ng YH, Pankratz VS, Leyva Y, et al. Does Racial Disparity in Kidney Transplant Waitlisting Persist After Accounting for Social Determinants of Health? Transplantation. 2020;104(7):1445-1455. doi:10.1097/TP.0000000000003002.

95. Oniscu GC, Schalkwijk AAH, Johnson RJ, Brown H, Forsythe JLR. Equity of access to renal transplant waiting list and renal transplantation in Scotland: cohort study. BMJ. 2003;327(7426):1261-1263. doi:10.1136/BMJ.327.7426.1261.

96. Pallet N, Thervet E, Alberti C, et al. Kidney transplant in black recipients: are African Europeans different from African Americans? Am J Transplant. 2005;5(11):2682-2687. doi:10.1111/J.1600-6143.2005.01057.X.

97. Park KS, Ryu GW, Jhee JH, et al. Serum Ferritin Predicts Mortality Regardless of Inflammatory and Nutritional Status in Patients Starting Dialysis: A Prospective Cohort Study. Blood Purif. 2015;40(3):209-217. doi:10.1159/000438819.

98. Park S, Park GC, Park J, et al. Disparity in Accessibility to and Prognosis of Kidney Transplantation According to Economic Inequality in South Korea: A Widening Gap After Expansion of Insurance Coverage. Transplantation. 2021;105(2):404-412. doi:10.1097/TP.0000000000003256.

99. Park S, Park J, Kim M, et al. Socioeconomic dependency and kidney transplantation accessibility and outcomes: a nationwide observational cohort study in South Korea. J Nephrol. 2021;34(1):211-219. doi:10.1007/S40620-020-00876-0.

100. Patzer RE, Amaral S, Wasse H, Volkova N, Kleinbaum D, McClellan WM. Neighborhood poverty and racial disparities in kidney transplant waitlisting. J Am Soc Nephrol. 2009;20(6):1333-1340. doi:10.1681/ASN.2008030335.

101. Patzer RE, Perryman JP, Schrager JD, et al. The role of race and poverty on steps to kidney transplantation in the Southeastern United States. Am J Transplant. 2012;12(2):358-368. doi:10.1111/J.1600-6143.2011.03927.X.

102. Peng RB, Lee H, Ke ZT, Saunders MR. Racial disparities in kidney transplant waitlist appearance in Chicago: Is it race or place? Clin Transplant. 2018;32(5). doi:10.1111/CTR.13195.

103. Pisoni RL, Young EW, Dykstra DM, et al. Vascular access use in Europe and the United States: results from the DOPPS. Kidney Int. 2002;61(1):305-316. doi:10.1046/J.1523-1755.2002.00117.X.

104. Pisoni RL, Zepel L, Fluck R, et al. International Differences in the Location and Use of Arteriovenous Accesses Created for Hemodialysis: Results From the Dialysis Outcomes and Practice Patterns Study (DOPPS). Am J Kidney Dis. 2018;71(4):469-478. doi:10.1053/J.AJKD.2017.09.012.

105. Pisoni RL, Zepel L, Zhao J, et al. International Comparisons of Native Arteriovenous Fistula Patency and Time to Becoming Catheter-Free: Findings From the Dialysis Outcomes and Practice Patterns Study (DOPPS). Am J Kidney Dis. 2021;77(2):245-254. doi:10.1053/J.AJKD.2020.06.020.

106. Prasad N, Sinha A, Gupta A, et al. Effect of body mass index on outcomes of peritoneal dialysis patients in India. Perit Dial Int. 2014;34(4):399-408. doi:10.3747/PDI.2013.00056.

107. Prasad S, Singh S, Duncan N, et al. Ethnicity and survival on dialysis in west London. Kidney Int. 2004;66(6):2416-2421. doi:10.1111/J.1523-1755.2004.66017.X.

108. Press R, Carrasquillo O, Nickolas T, Radhakrishnan J, Shea S, Barr RG. Race/ethnicity, poverty status, and renal transplant outcomes. Transplantation. 2005;80(7):917-924. doi:10.1097/01.TP.0000173379.53347.31.

109. Promislow S, Hemmelgarn B, Rigatto C, et al. Young aboriginals are less likely to receive a renal transplant: a Canadian national study. BMC Nephrol. 2013;14. doi:10.1186/1471-2369-14-11.

110. Pruthi R, Robb MWL, Oniscu GC, et al. Inequity in Access to Transplantation in the United Kingdom. Clin J Am Soc Nephrol. 2020;15(6):830-842. doi:10.2215/CJN.11460919.

111. Puqh JA, Tuley MR, Basu S. Survival among Mexican-Americans, non-Hispanic whites, and African-Americans with end-stage renal disease: the emergence of a minority pattern of increased incidence and prolonged survival. Am J Kidney Dis. 1994;23(6):803-807. doi:10.1016/S0272-6386(12)80133-8.

112. Purnell TS, Luo X, Cooper LA, et al. Association of Race and Ethnicity With Live Donor Kidney Transplantation in the United States From 1995 to 2014. JAMA. 2018;319(1):49-61. doi:10.1001/JAMA.2017.19152.

113. Purnell TS, Xu P, Leca N, Hall YN. Racial differences in determinants of live donor kidney transplantation in the United States. Am J Transplant. 2013;13(6):1557-1565. doi:10.1111/AJT.12258.

114. Qian J, Lee T, Thamer M, Zhang Y, Crews DC, Allon M. Racial Disparities in the Arteriovenous Fistula Care Continuum in Hemodialysis Patients. Clin J Am Soc Nephrol. 2020;15(12):1796-1803. doi:10.2215/CJN.03600320.

115. Ravanan R, Udayaraj U, Ansell D, et al. Variation between centres in access to renal transplantation in UK: longitudinal cohort study. BMJ. 2010;341(7766):238. doi:10.1136/BMJ.C3451.

116. Reddan D, Klassen P, Frankenfield DL, et al. National profile of practice patterns for hemodialysis vascular access in the United States. J Am Soc Nephrol. 2002;13(8):2117-2124. doi:10.1097/01.ASN.0000022422.79790.A8.

117. Rita A, Michael K, Geest Sabina D, et al. Demographic, psychosocial and health disparities between living and deceased renal allograft recipients in Switzerland. Swiss Med Wkly. 2021;151(31). doi:10.4414/SMW.2021.20532.

118. Robinson BM, Joffe MM, Pisoni RL, Port FK, Feldman HI. Revisiting survival differences by race and ethnicity among hemodialysis patients: the Dialysis Outcomes and Practice Patterns Study. J Am Soc Nephrol. 2006;17(10):2910-2918. doi:10.1681/ASN.2005101078.

119. Roderick P, Byrne C, Casula A, et al. Survival of patients from South Asian and Black populations starting renal replacement therapy in England and Wales. Nephrol Dial Transplant. 2009;24(12):3774-3782. doi:10.1093/NDT/GFP348.

120. Ros S, Remón C, Qureshi AR, Quiros P, Lindholm B, Carrero JJ. Increased risk of fatal infections in women starting peritoneal dialysis. Perit Dial Int. 2013;33(5):487-494. doi:10.3747/PDI.2012.00243.

121. Sanabria M, Mũoz J, Trillos C, et al. Dialysis outcomes in Colombia (DOC) study: a comparison of patient survival on peritoneal dialysis vs hemodialysis in Colombia. Kidney Int Suppl. 2008;73(108). doi:10.1038/SJ.KI.5002619.

122. Sandhu GS, Khattak M, Rout P, et al. Social Adaptability Index: application and outcomes in a dialysis population. Nephrol Dial Transplant. 2011;26(8):2667-2674. doi:10.1093/NDT/GFQ789.

123. Schaeffner ES, Mehta J, Winkelmayer WC. Educational level as a determinant of access to and outcomes after kidney transplantation in the United States. Am J Kidney Dis. 2008;51(5):811-818. doi:10.1053/J.AJKD.2008.01.019.

124. Schaubel DE, Stewart DE, Morrison HI, et al. Sex Inequality in Kidney Transplantation Rates. Arch Intern Med. 2000;160(15):2349-2354. doi:10.1001/ARCHINTE.160.15.2349.

125. Schellartz I, Mettang S, Shukri A, Scholten N, Pfaff H, Mettang T. Early Referral to Nephrological Care and the Uptake of Peritoneal Dialysis. An Analysis of German Claims Data. Int J Environ Res Public Health. 2021;18(16). doi:10.3390/IJERPH18168359.

126. Sequist TD, Narva AS, Stiles SK, Karp SK, Cass A, Ayanian JZ. Access to renal transplantation among American Indians and Hispanics. Am J Kidney Dis. 2004;44(2):344-352. doi:10.1053/J.AJKD.2004.04.039.

127. Shabankhani B, Kazemnejad A, Zaeri F, Espahbodi F, Haji Ahmadi M, Mirkazemi R. Survival Factors in Patients With End-stage Renal Disease in Mazandaran Province, Iran - PubMed. Iran J Kidney Dis. 2016;10(2):79-84. https://pubmed.ncbi.nlm.nih.gov/26921749/. Accessed June 4, 2022.

128. Shah S, Leonard AC, Meganathan K, Christianson AL, Thakar C V. Gender and Racial Disparities in Initial Hemodialysis Access and Outcomes in Incident End-Stage Renal Disease Patients. Am J Nephrol. 2018;48(1):4-14. doi:10.1159/000490624.

129. Shen JI, Chen L, Vangala S, et al. Socioeconomic Factors and Racial and Ethnic Differences in the Initiation of Home Dialysis. Kidney Med. 2020;2(2):105-115. doi:10.1016/J.XKME.2019.11.006.

130. Sood MM, Hemmelgarn B, Rigatto C, et al. Association of modality with mortality among Canadian Aboriginals. Clin J Am Soc Nephrol. 2012;7(12):1988-1995. doi:10.2215/CJN.03650412.

131. Sood MM, Komenda P, Sood AR, et al. Adverse outcomes among Aboriginal patients receiving peritoneal dialysis. C Can Med Assoc J. 2010;182(13):1433. doi:10.1503/CMAJ.100105.

132. Soucie JM, Neylan JF, McClellan W. Race and sex differences in the identification of candidates for renal transplantation. Am J Kidney Dis. 1992;19(5):414-419. doi:10.1016/S0272-6386(12)80947-4.

133. Stack AG. Determinants of modality selection among incident US dialysis patients: results from a national study. J Am Soc Nephrol. 2002;13(5):1279-1287. doi:10.1681/ASN.V1351279.

134. Stehman-Breen CO, Sherrard DJ, Gillen D, Caps M. Determinants of type and timing of initial permanent hemodialysis vascular access. Kidney Int. 2000;57(2):639-645. doi:10.1046/J.1523-1755.2000.00885.X.

135. Sypek MP, Clayton PA, Lim W, et al. Access to waitlisting for deceased donor kidney transplantation in Australia. Nephrology (Carlton). 2019;24(7):758-766. doi:10.1111/NEP.13484.

136. Taber DJ, Gebregziabher M, Payne EH, Srinivas T, Baliga PK, Egede LE. Overall Graft Loss Versus Death-Censored Graft Loss: Unmasking the Magnitude of Racial Disparities in Outcomes Among US Kidney Transplant Recipients. Transplantation. 2017;101(2):402-410. doi:10.1097/TP.0000000000001119.

137. Teixeira FIR, Lopes MLH, Silva GA dos S, Santos RF. Survival of hemodialysis patients at a university hospital. J Bras Nefrol. 2015;37(1):64-71. doi:10.5935/0101-2800.20150010.

138. Trinh E, Na Y, Sood MM, Chan CT, Perl J. Racial Differences in Home Dialysis Utilization and Outcomes in Canada. Clin J Am Soc Nephrol. 2017;12(11):1841-1851. doi:10.2215/CJN.03820417.

139. Trivedi PS, Lind KE, Ray CE, Rochon PJ, Ryu RK. Race and Sex Disparities in Outcomes of Dialysis Access Maintenance Interventions. J Vasc Interv Radiol. 2018;29(4):476-481.e1. doi:10.1016/J.JVIR.2017.10.018.

140. Udayaraj U, Ben-Shlomo Y, Roderick P, et al. Social deprivation, ethnicity, and access to the deceased donor kidney transplant waiting list in England and Wales. Transplantation. 2010;90(3):279-285. doi:10.1097/TP.0B013E3181E346E3.

141. Udayaraj U, Ben-Shlomo Y, Roderick P, et al. Social deprivation, ethnicity, and uptake of living kidney donor transplantation in the United Kingdom. Transplantation. 2012;93(6):610-616. doi:10.1097/TP.0B013E318245593F.

142. Villar E, Rabilloud M, Berthoux F, Vialtel P, Labeeuw M, Pouteil-Noble C. A multicentre study of registration on renal transplantation waiting list of the elderly and patients with type 2 diabetes. Nephrol Dial Transplant. 2004;19(1):207-214. doi:10.1093/NDT/GFG500.

143. Vogel SL, Singh T, Astor BC, Waheed S. Gender differences in peritoneal dialysis initiation in the US end-stage renal disease population. Perit Dial Int. 2020;40(1):57-61. doi:10.1177/0896860819878656.

144. Wallace EL, Lea J, Chaudhary NS, et al. Home Dialysis Utilization Among Racial and Ethnic Minorities in the United States at the National, Regional, and State Level. Perit Dial Int. 2017;37(1):21-29. doi:10.3747/PDI.2016.00025.

145. Wang T, Tziviskou E, Chu M, et al. Differences in survival on peritoneal dialysis between oriental Asians and Caucasians: one center's experience. Int Urol Nephrol. 2003;35(2):267-274. doi:10.1023/B:UROL.0000020286.83411.D1.

146. Wang Z, Zhang Y, Xiong F, et al. Association between medical insurance type and survival in patients undergoing peritoneal dialysis. BMC Nephrol. 2015;16(1). doi:10.1186/S12882-015-0023-7.

147. Ward FL, O'Kelly P, Donohue F, et al. Influence of socioeconomic status on allograft and patient survival following kidney transplantation. Nephrology (Carlton). 2015;20(6):426-433. doi:10.1111/NEP.12410.

148. Ward FL, O'Kelly P, Donohue F, et al. The influence of socioeconomic status on patient survival on chronic dialysis. Hemodial Int. 2015;19(4):601-608. doi:10.1111/HDI.12295.

149. Waterman AD, Peipert JD, Hyland SS, Mccabe MS, Schenk EA, Liu J. Modifiable patient characteristics and racial disparities in evaluation completion and living donor transplant. Clin J Am Soc Nephrol. 2013;8(6):995-1002. doi:10.2215/CJN.08880812.

150. Wesselman H, Ford CG, Leyva Y, et al. Social Determinants of Health and Race Disparities in Kidney Transplant. Clin J Am Soc Nephrol. 2021;16(2):262-274. doi:10.2215/CJN.04860420.

151. Wiley HRL, Varilek BM, Saucedo-Crespo H, et al. Kidney Transplant Outcomes in Indigenous People of the Northern Great Plains of the United States. Transplant Proc. 2021;53(6):1872-1879. doi:10.1016/J.TRANSPROCEED.2021.05.003.

152. Williams A, Richardson C, McCready J, et al. Black Ethnicity is Not a Risk Factor for Mortality or Graft Loss After Kidney Transplant in the United Kingdom. Exp Clin Transplant. 2018;16(6):682-689. doi:10.6002/ECT.2018.0241.

153. Wilmink T, Wijewardane A, Lee K, et al. Effect of ethnicity and socioeconomic status on vascular access provision and performance in an urban NHS hospital. Clin Kidney J. 2017;10(1):62-67. doi:10.1093/CKJ/SFW099.

154. Winkelmayer WC, Glynn RJ, Levin R, Owen W. J, Avorn J. Late referral and modality choice in end-stage renal disease. Kidney Int. 2001;60(4):1547-1554. doi:10.1046/J.1523-1755.2001.00958.X.

155. Wolf M, Betancourt J, Chang Y, et al. Impact of activated vitamin D and race on survival among hemodialysis patients. J Am Soc Nephrol. 2008;19(7):1379-1388. doi:10.1681/ASN.2007091002.

156. Woo K, Yao J, Selevan D, Hye RJ. Influence of vascular access type on sex and ethnicity-related mortality in hemodialysis-dependent patients. Perm J. 2012;16(2):4-9. doi:10.7812/TPP/12-005.

157. Xu R, Han QF, Zhu TY, et al. Impact of individual and environmental socioeconomic status on peritoneal dialysis outcomes: a retrospective multicenter cohort study. PLoS One. 2012;7(11). doi:10.1371/JOURNAL.PONE.0050766.

158. Yan G, Norris KC, Yu AJ, et al. The relationship of age, race, and ethnicity with survival in dialysis patients. Clin J Am Soc Nephrol. 2013;8(6):953-961. doi:10.2215/CJN.09180912.

159. Yang ZK, Han QF, Zhu TY, et al. The associations between the family education and mortality of patients on peritoneal dialysis. PLoS One. 2014;9(5). doi:10.1371/JOURNAL.PONE.0095894.

160. Yeates KE, Cass A, Sequist TD, et al. Indigenous people in Australia, Canada, New Zealand and the United States are less likely to receive renal transplantation. Kidney Int. 2009;76(6):659-664. doi:10.1038/KI.2009.236.

161. Yeates KE, Schaubel DE, Cass A, Sequist TD, Ayanian JZ. Access to renal transplantation for minority patients with ESRD in Canada. Am J Kidney Dis. 2004;44(6):1083-1089. doi:10.1053/J.AJKD.2004.08.031.

162. Yolgösteren A. Relationship between hemodialysis patients' educational level and arteriovenous fistula patency. Vascular. 2020;28(5):604-608. doi:10.1177/1708538120918417.

163. Zhang X, Melanson TA, Plantinga LC, et al. Racial/ethnic disparities in waitlisting for deceased donor kidney transplantation 1 year after implementation of the new national kidney allocation system. Am J Transplant. 2018;18(8):1936-1946. doi:10.1111/AJT.14748.

164. Zhang Y, Gerdtham UG, Rydell H, Jarl J. Socioeconomic Inequalities in the Kidney Transplantation Process: A Registry-Based Study in Sweden. Transplant direct. 2018;4(2). doi:10.1097/TXD.0000000000000764.

165. Zhang Y, Jarl J, Gerdtham UG. Are There Inequities in Treatment of End-Stage Renal Disease in Sweden? A Longitudinal Register-Based Study on Socioeconomic Status-Related Access to Kidney Transplantation. Int J Environ Res Public Health. 2017;14(2). doi:10.3390/IJERPH14020119.

Supplementary S4: Risk of bias assessment

Suppl. Table S4. Outcomes of the ROBINS-I evaluation

Study	*Confounding*	*Selection of participants*	*Classification of exposures*	*Deviations from intended exposures*	*Missing data*	*Measurement of outcomes*	*Selection of the reported result*	*Overall*
**2021; Qian**	Low	Low	Low	Low	Low	Low	Low	**Low**
**2021; MacRae**	Low	Low	Low	Low	Low	Low	Low	**Low**
**2021; Pisoni**	Low	Low	Low	Low	Low	Low	Low	**Low**
**2021; Schellartz**	Moderate	Low	Moderate	Low	NI	Low	Low	**Moderate**
**2021; Barth**	Low	Low	Low	Low	Low	Low	Low	**Low**
**2021; Wesselman**	Low	Low	Low	Low	NI	Low	Low	**Low**
**2021; Cirillo**	Low	Low	Low	Low	NI	Low	Low	**Low**
**2021; Ahearn**	Low	Low	Low	Low	Low	Low	Low	**Low**
**2021; Rita**	Moderate	Low	Low	Low	Low	Low	Low	**Moderate**
**2021; Wiley**	Low	Low	Low	Low	Low	Low	Low	**Low**
**2021; Belyaev**	Moderate	Low	Low	Low	Low	Low	Low	**Moderate**
**2021; Park**	Low	Low	Low	Low	NI	Low	Low	**Low**
**2020; Arya**	Low	Low	Low	Low	Low	Low	Low	**Low**
**2020; Arhuidese**	Low	Low	Low	Low	Low	Low	Low	**Low**
**2020; Copeland**	Low	Low	Low	Low	Low	Low	Low	**Low**
**2020; Krishnasamy**	Low	Low	Low	Low	NI	Low	Low	**Low**
**2020; Yolgosteren**	Moderate	Low	Low	Low	NI	Low	Low	**Moderate**
**2020; Ku**	Low	Low	Low	Low	Low	Low	Low	**Low**
**2020; Hayat**	Low	Low	Low	Low	Low	Low	Low	**Low**
**2020; Vogel**	Low	Low	Low	Low	Low	Low	Low	**Low**
**2020; Shen**	Low	Low	Low	Low	Low	Low	Low	**Low**
**2020; Melk**	Moderate	Low	Low	Low	NI	Low	Low	**Moderate**

**2020; Park**	Low	Low	Moderate	Low	Low	Low	Low	**Moderate**
**2020; Pruthi**	Low	Low	Low	Low	Low	Low	Low	**Low**
**2020; Murphy**	Low	Low	Low	Low	Low	Low	Low	**Low**
**2020; Ng**	Low	Low	Low	Low	Low	Low	Low	**Low**
**2019; Nee**	Low	Low	Low	Low	Low	Low	Low	**Low**
**2019; Howson**	Low	Low	Low	Low	Low	Low	Low	**Low**
**2019; King**	Low	Low	Low	Low	NI	Low	Low	**Low**
**2019; Myaskovsky**	Low	Low	Low	Low	Low	Low	Low	**Low**
**2019; Ke**	Low	Low	Low	Low	Low	Low	Low	**Low**
**2019; Naylor**	Low	Low	Moderate	Low	Low	Low	Low	**Moderate**
**2018; Shah**	Low	Low	Low	Low	Low	Low	Low	**Low**
**2018; Huria**	Low	Low	Low	Low	Low	Low	Low	**Low**
**2018; Lin**	Low	Moderate	Low	Low	Low	Low	Low	**Moderate**
**2018; Krishnasamy**	Low	Low	Low	Low	Low	Low	Low	**Low**
**2018; Keddis**	Low	Moderate	Low	Low	Low	Low	Low	**Moderate**
**2018; Williams**	Moderate	Low	Low	Low	Low	Low	Low	**Moderate**
**2018; Sypek**	Low	Low	Low	Low	Low	Low	Low	**Low**
**2018; Liu**	Low	Low	Low	Low	Low	Low	Low	**Low**
**2018; Chatelet**	Low	Low	Low	Low	Low	Low	Low	**Low**
**2018[a]; Zhang**	Low	Low	Low	Low	NI	Low	Low	**Low**
**2018[b]; Zhang**	Low	Low	Low	Low	NI	Low	Low	**Low**
**2018; Peng**	Low	Low	Low	Low	Low	Low	Low	**Low**
**2018; Purnell**	Low	Low	Low	Low	NI	Low	Low	**Low**
**2017; Trivedi**	Low	Low	Low	Low	Low	Low	Low	**Low**
**2017; McGill**	Low	Low	Low	Low	Low	Low	Low	**Low**
**2017; McKercher**	Low	Low	Low	Low	Low	Low	Low	**Low**
**2017; Pisoni**	Low	Low	Low	Low	NI	Low	Low	**Low**
**2017; Wilmink**	Moderate	Low	Low	Low	NI	Low	Low	**Moderate**
**2017; Gulcan**	Moderate	Moderate	Low	Low	Low	Low	Low	**Moderate**
**2017; Kim**	Low	Low	Low	Low	Low	Low	Low	**Low**
**2017; Chan**	Low	Low	Low	Low	Low	Low	Low	**Low**
**2017; Wallace**	Moderate	Low	Moderate	Low	Low	Low	Low	**Moderate**

**2017; Trinh**	Low	Low	Low	Low	NI	Low	Low	**Low**
**2017; Imanishi**	Low	Low	Low	Low	NI	Low	Low	**Low**
**2017; Gill**	Low	Low	Low	Low	Low	Low	Low	**Low**
**2017; Zhang**	Low	Low	Low	Low	NI	Low	Low	**Low**
**2017; Choi**	Low	Low	Low	Low	Low	Low	Low	**Low**
**2017; Taber**	Low	Low	Low	Low	Low	Low	Low	**Low**
**2016; Tamayo Isla**	Serious	Low	Low	Low	NI	Low	Low	**Serious**
**2016; Liu**	Low	Low	Low	Low	Low	Low	Low	**Low**
**2016; Shabankhani**	Moderate	Moderate	Low	Low	Low	Low	Low	**Moderate**
**2016; Mucsi**	Low	Low	Low	Low	Low	Low	Low	**Low**
**2016; Kihal-Talantikite**	Low	Moderate	Moderate	Low	Low	Low	Low	**Moderate**
**2016; Molmenti**	Low	Moderate	Low	Low	NI	Low	Low	**Moderate**
**2015; Nee**	Low	Low	Low	Low	NI	Low	Low	**Low**
**2015; Lawton**	Low	Low	Low	Low	NI	Low	Low	**Low**
**2015; van den Beukel**	Low	Low	Low	Low	Low	Low	Low	**Low**
**2015; Park**	Serious	Low	Low	Low	NI	Low	Low	**Serious**
**2015; Wang**	Moderate	Moderate	Low	Low	Low	Low	Low	**Moderate**
**2015; Ward**	Low	Low	Low	Low	Low	Low	Low	**Low**
**2015; Ilori**	Low	Low	Low	Low	Low	Low	Low	**Low**
**2015; Matsuoka**	Low	Moderate	Low	Low	Low	Low	Low	**Moderate**
**2015; Ward**	Low	Low	Low	Low	Low	Low	Low	**Low**
**2015; Arce**	Low	Low	Low	Low	Low	Low	Low	**Low**
**2014; Goldfarb-Rumyantzev**	Low	Low	Low	Low	NI	Low	Low	**Low**
**2014; Cole**	Low	Low	Low	Low	NI	Low	Low	**Low**
**2014; Burrows**	Low	Low	Low	Low	Low	Low	Low	**Low**
**2014; Prasad**	Serious	Low	Low	Low	NI	Low	Low	**Serious**
**2014; Grace**	Moderate	Low	Low	Low	Low	Low	Low	**Moderate**
**2014; Kim**	Serious	Low	Low	Low	Low	Low	Low	**Serious**
**2014; Yang**	Low	Low	Low	Low	Low	Low	Low	**Low**

**2014; de Moraes**	Low	Low	Low	Low	NI	Low	Low	**Low**
**2014; Texeira**	Low	Moderate	Low	Low	Low	Low	Low	**Moderate**
**2013; Yan**	Low	Low	Low	Low	Low	Low	Low	**Low**
**2013; Chang**	Moderate	Low	Low	Low	NI	Low	Low	**Moderate**
**2013; Ros**	Low	Low	Low	Low	Low	Low	Low	**Low**
**2013; Fernadez**	Low	Low	Moderate	Low	Low	Low	Low	**Moderate**
**2013; Kimmel**	Low	Low	Low	Low	Low	Low	Low	**Low**
**2013; Arce**	Low	Low	Low	Low	NI	Low	Low	**Low**
**2013; Purnell**	Low	Low	Low	Low	Low	Low	Low	**Low**
**2013; Waterman**	Low	Moderate	Low	Low	Low	Low	Low	**Moderate**
**2013; PromisLow**	Low	Low	Low	Low	Low	Low	Low	**Low**
**2012; Sood**	Moderate	Low	Moderate	Low	Low	Low	Low	**Moderate**
**2012; Woo**	Moderate	Low	Low	Low	NI	Low	Low	**Moderate**
**2012; van den Beukel**	Low	Low	Low	Low	Low	Low	Low	**Low**
**2012; Marinovich**	Low	Low	Low	Low	NI	Low	Low	**Low**
**2012; Xu**	Low	Low	Low	Low	NI	Low	Low	**Low**
**2012; Huang**	Low	Low	Low	Low	Low	Low	Low	**Low**
**2012; Lockridge**	Moderate	Low	Low	Low	Low	Low	Low	**Moderate**
**2012; Neri**	Low	Moderate	Low	Low	Low	Low	Low	**Moderate**
**2012; Couchoud**	Low	Low	Low	Low	Low	Low	Low	**Low**
**2012; Goldfarb-Rumyantzev**	Low	Low	Low	Low	NI	Low	Low	**Low**
**2012; Udayaraj**	Low	Low	Moderate	Low	Low	Low	Low	**Moderate**
**2012; Patzer**	Low	Low	Moderate	Low	Low	Low	Low	**Moderate**
**2012; Garg**	Moderate	Low	Low	Low	Low	Low	Low	**Moderate**
**2011; Lopez-Vargas**	Low	Low	Low	Low	NI	Low	Low	**Low**
**2011; Carrero**	Moderate	Low	Low	Low	Low	Low	Low	**Moderate**
**2011; Chidambaram**	Low	Low	Low	Low	Low	Low	Low	**Low**
**2011; Bastos**	Low	Low	Low	Low	Low	Low	Low	**Low**
**2011; Machado**	Low	Low	Low	Low	Low	Low	Low	**Low**

**2011; Hall**	Low	Low	Low	Low	NI	Low	Low	**Low**
**2010; Chung**	Moderate	Low	Low	Low	NI	Low	Low	**Moderate**
**2010; Huang**	Moderate	Moderate	Low	Low	Low	Low	Low	**Moderate**
**2010; Sood**	Low	Low	Low	Low	Low	Low	Low	**Low**
**2010; Sandhu**	Low	Low	Low	Low	NI	Low	Low	**Low**
**2010; Udayaraj**	Low	Low	Moderate	Low	Low	Low	Low	**Moderate**
**2010; Ng**	Moderate	Low	Low	Low	Low	Low	Low	**Moderate**
**2010; Ravanan**	Moderate	Low	Low	Low	Low	Low	Low	**Moderate**
**2009; Roderick**	Low	Low	Low	Low	Low	Low	Low	**Low**
**2009; de Mutsert**	Serious	Low	Low	Low	Low	Low	Low	**Serious**
**2009; Eisenstein**	Low	Low	Low	Low	Low	Low	Low	**Low**
**2009; Yeates**	Low	Low	Low	Low	Low	Low	Low	**Low**
**2009; Feyssa**	Serious	Low	Low	Low	Low	Low	Low	**Serious**
**2009; Patzer**	Low	Low	Moderate	Low	Low	Low	Low	**Moderate**
**2008; Wolf**	Low	Low	Low	Low	Low	Low	Low	**Low**
**2008; Sanabria**	Low	Low	Low	Low	Low	Low	Low	**Low**
**2008; Hall**	Low	Low	Moderate	Low	Low	Low	Low	**Moderate**
**2008; Schaeffner**	Low	Low	Low	Low	Low	Low	Low	**Low**
**2007; Gill**	Low	Low	Low	Low	NI	Low	Low	**Low**
**2006; Robinson**	Low	Low	Low	Low	Low	Low	Low	**Low**
**2006; Caskey**	Low	Low	Low	Low	Low	Low	Low	**Low**
**2006; Hemmelgarn**	Low	Low	Low	Low	Low	Low	Low	**Low**
**2006; Bayat**	Low	Low	Low	Low	Low	Low	Low	**Low**
**2005; Chung**	Moderate	Low	Low	Low	NI	Low	Low	**Moderate**
**2005; Mircescu**	Low	Low	Low	Low	Low	Low	Low	**Low**
**2005; Kutner**	Serious	Low	Low	Low	Low	Low	Low	**Serious**
**2005; Press**	Moderate	Low	Low	Low	Low	Low	Low	**Moderate**
**2005; Pallet**	Moderate	Low	Low	Low	Low	Low	Low	**Moderate**
**2005; Chakkera**	Low	Low	Low	Low	Low	Low	Low	**Low**
**2004; Prasad**	Moderate	Low	Low	Low	NI	Low	Low	**Moderate**
**2004; Jager**	Moderate	Low	Low	Low	Low	Low	Low	**Moderate**
**2004; Yeates**	Low	Low	Low	Low	Low	Low	Low	**Low**

**2004; Sequist**	Low	Low	Low	Low	Low	Low	Low	**Low**
**2004; Villar**	Low	Low	Low	Low	Low	Low	Low	**Low**
**2003; Wang**	Serious	Low	Low	Low	NI	Low	Low	**Serious**
**2003; Cass**	Moderate	Low	Low	Low	NI	Low	Low	**Moderate**
**2003; Oniscu**	Low	Low	Low	Low	Low	Low	Low	**Low**
**2002; Reddan**	Moderate	Low	Low	Low	NI	Low	Low	**Moderate**
**2002; Pisoni**	Low	Low	Low	Low	NI	Low	Low	**Low**
**2002; Iseki**	Moderate	Low	Low	Low	NI	Low	Low	**Moderate**
**2002; Stack**	Low	Low	Low	Low	Low	Low	Low	**Low**
**2001; Winkelmayer**	Moderate	Low	Low	Low	NI	Low	Low	**Moderate**
**2000; Stehman-Breen**	Low	Low	Low	Low	NI	Low	Low	**Low**
**2000; Allon**	Low	Low	Low	Low	NI	Low	Low	**Low**
**2000; Schaubel**	Low	Low	Low	Low	Low	Low	Low	**Low**
**2000; Garg**	Low	Low	Low	Low	NI	Low	Low	**Low**
**1999; Chandna**	Low	Moderate	Low	Low	NI	Low	Low	**Moderate**
**1996; Bleyer**	Moderate	Low	Low	Low	NI	Low	Low	**Moderate**
**1996; Medina**	Moderate	Moderate	Low	Low	NI	Low	Low	**Moderate**
**1995; Barker-Cummings**	Moderate	Low	Low	Low	NI	Low	Low	**Moderate**
**1994; Cowie**	Moderate	Low	Low	Low	NI	Low	Low	**Moderate**
**1994; Puqh**	Low	Low	Low	Low	Low	Low	Low	**Low**
**1992; Soucie**	Moderate	Low	Low	Low	Low	Low	Low	**Moderate**

NI: no information

Supplementary S5: Forest plots

5.1. Arteriovenous fistula/graft *vs*. Central venous catheter

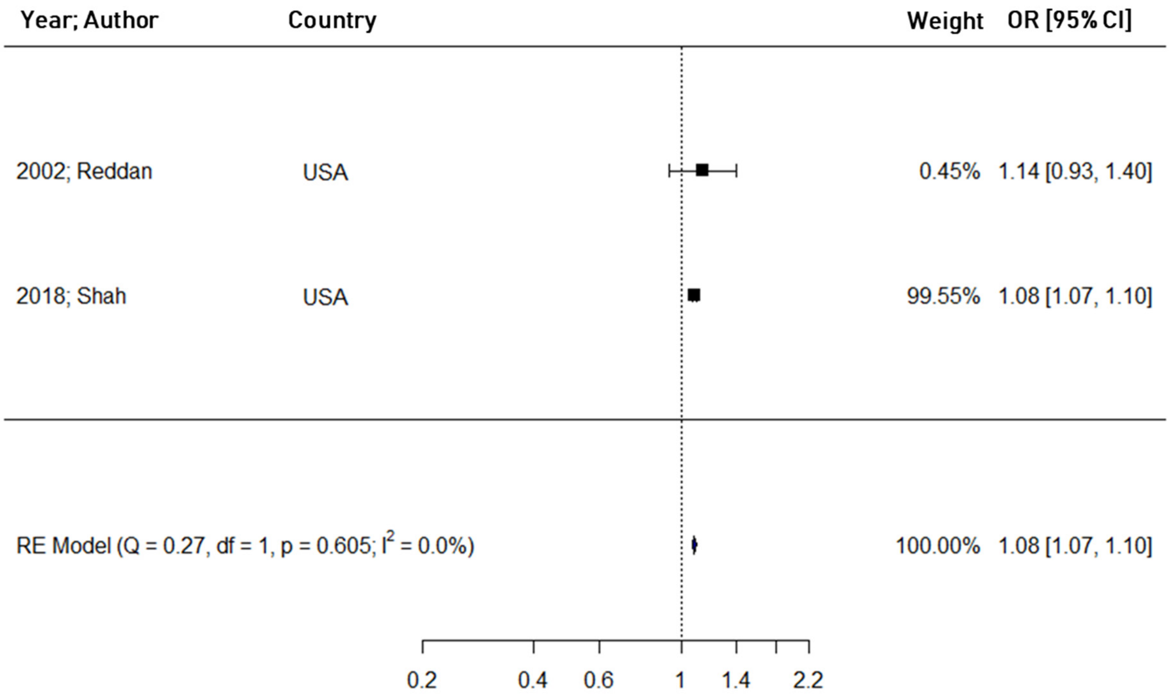

Suppl. Figure S1. Forest plot comparing Black and White patients (reference) regarding dialysis initiation with arteriovenous fistula or graft.

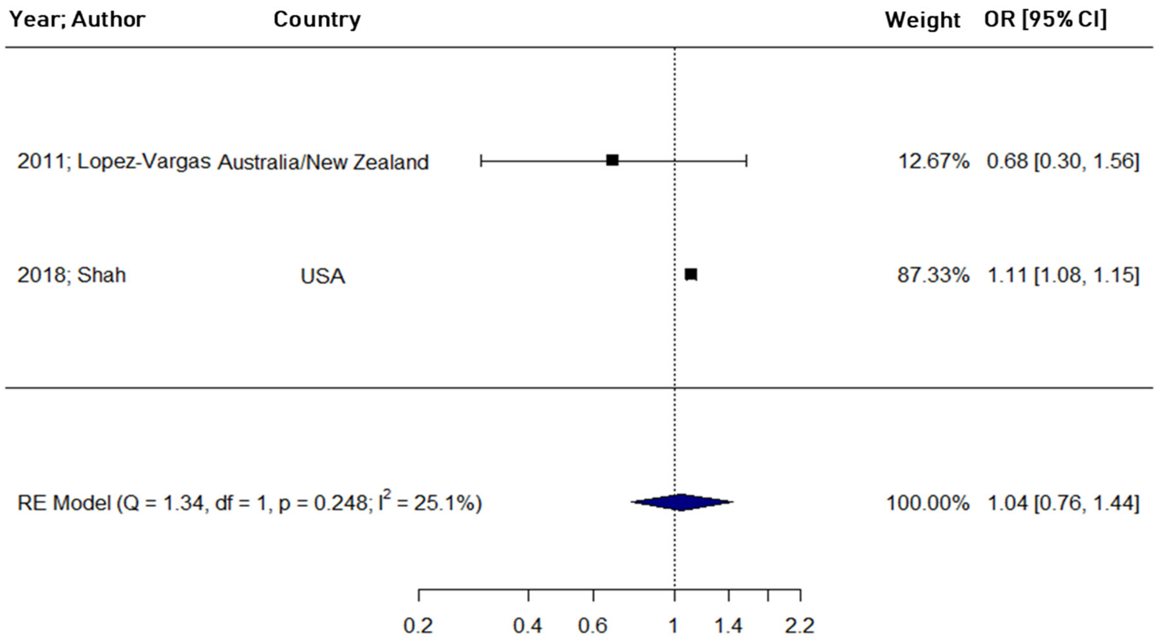

Suppl. Figure S2. Forest plot comparing Asian and White patients (reference) regarding dialysis initiation with arteriovenous fistula or graft.

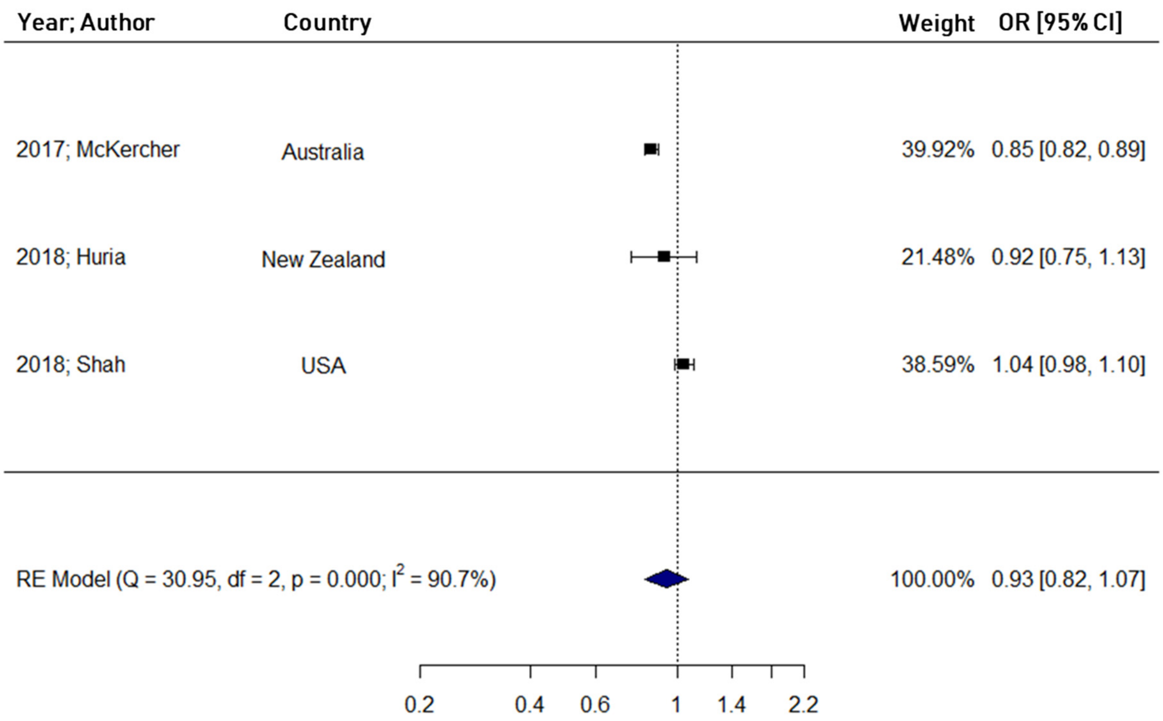

Suppl. Figure S3. Forest plot comparing Indigenous and White patients (reference) regarding dialysis initiation with arteriovenous fistula or graft.

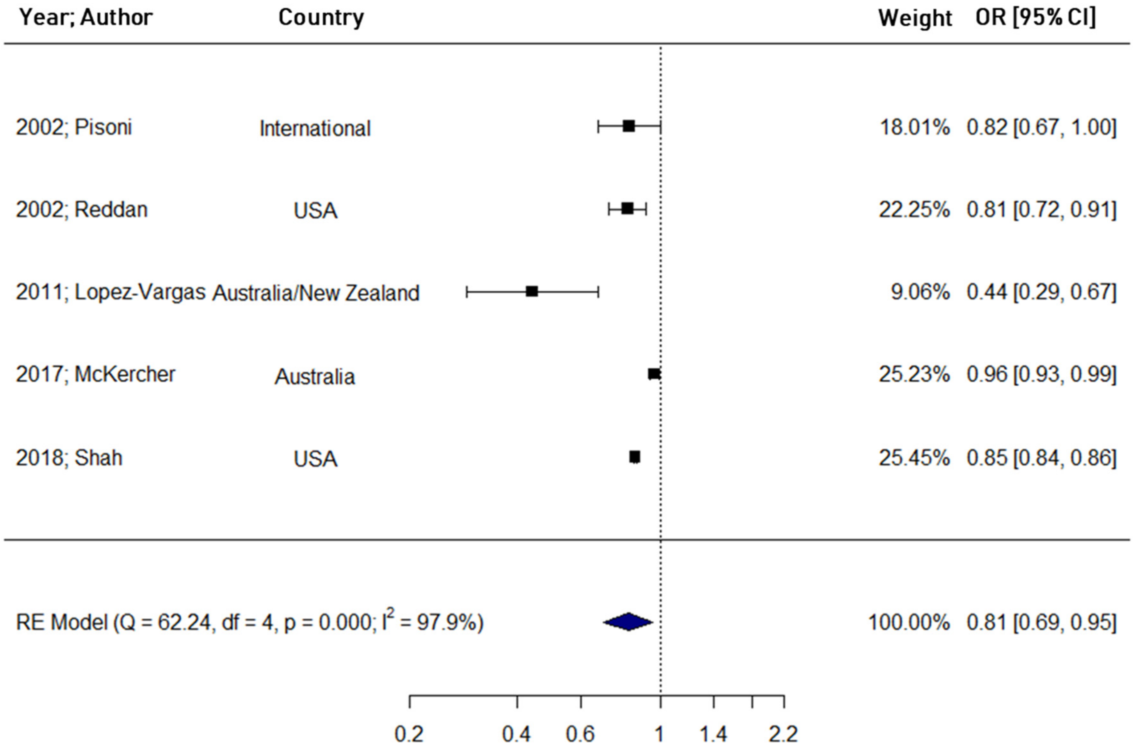

Suppl. Figure S4. Forest plot comparing female and male patients (reference) regarding dialysis initiation with arteriovenous fistula or graft.

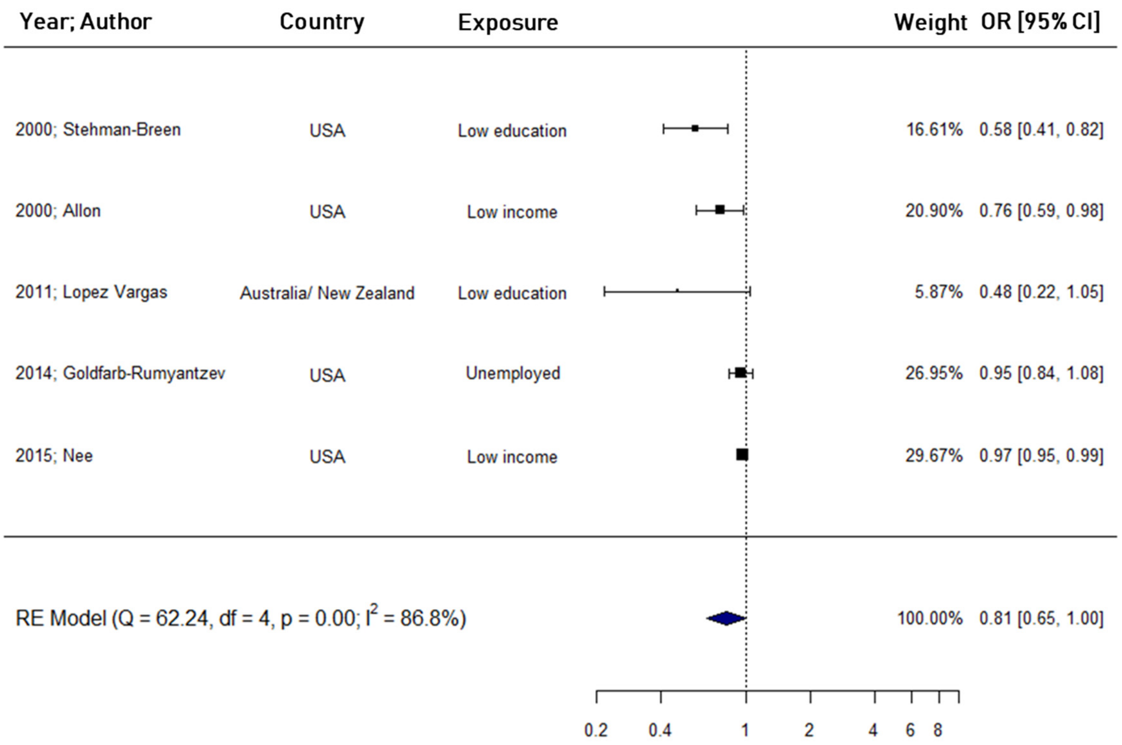

Suppl. Figure S5. Forest plot comparing low and high socioeconomic status patients (reference) regarding dialysis initiation with arteriovenous fistula or graft.

5.2. Arteriovenous fistula *vs*. Arteriovenous graft

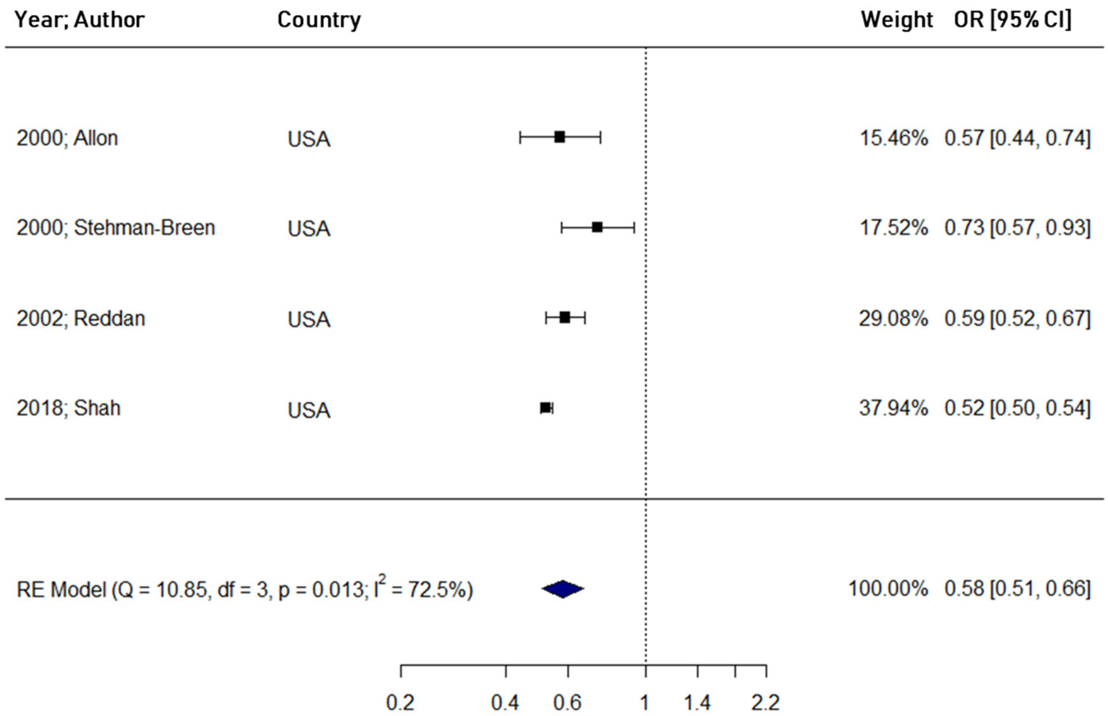

Suppl. Figure S6. Forest plot comparing Black and White patients (reference) regarding dialysis initiation with arteriovenous fistula *vs*. arteriovenous graft.

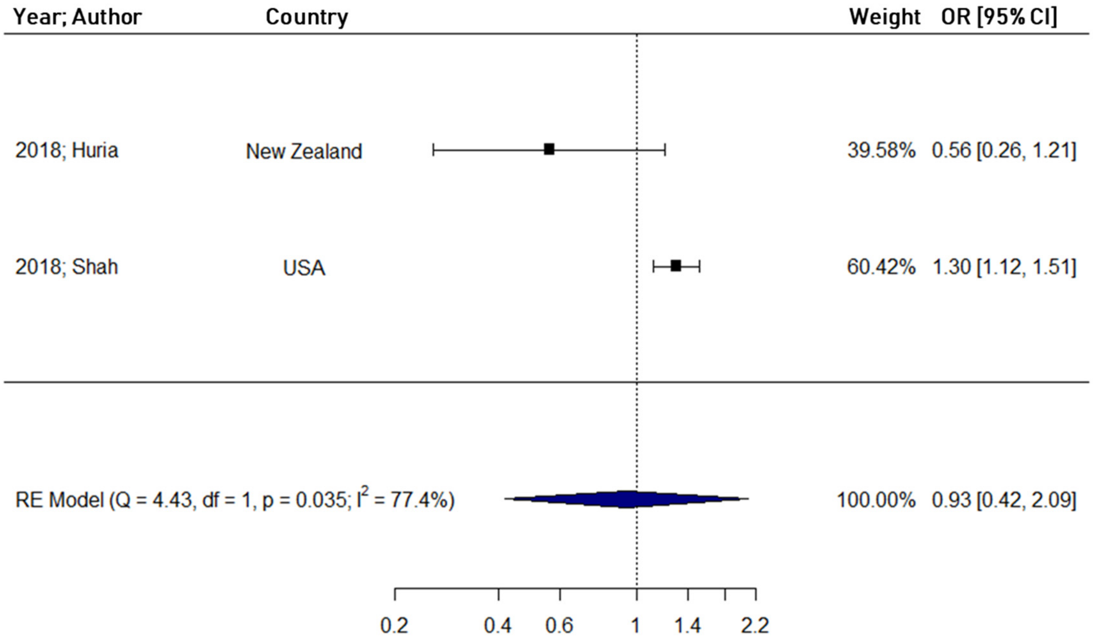

Suppl. Figure S7. Forest plot comparing Indigenous and White patients (reference) regarding dialysis initiation with arteriovenous fistula *vs*. arteriovenous graft.

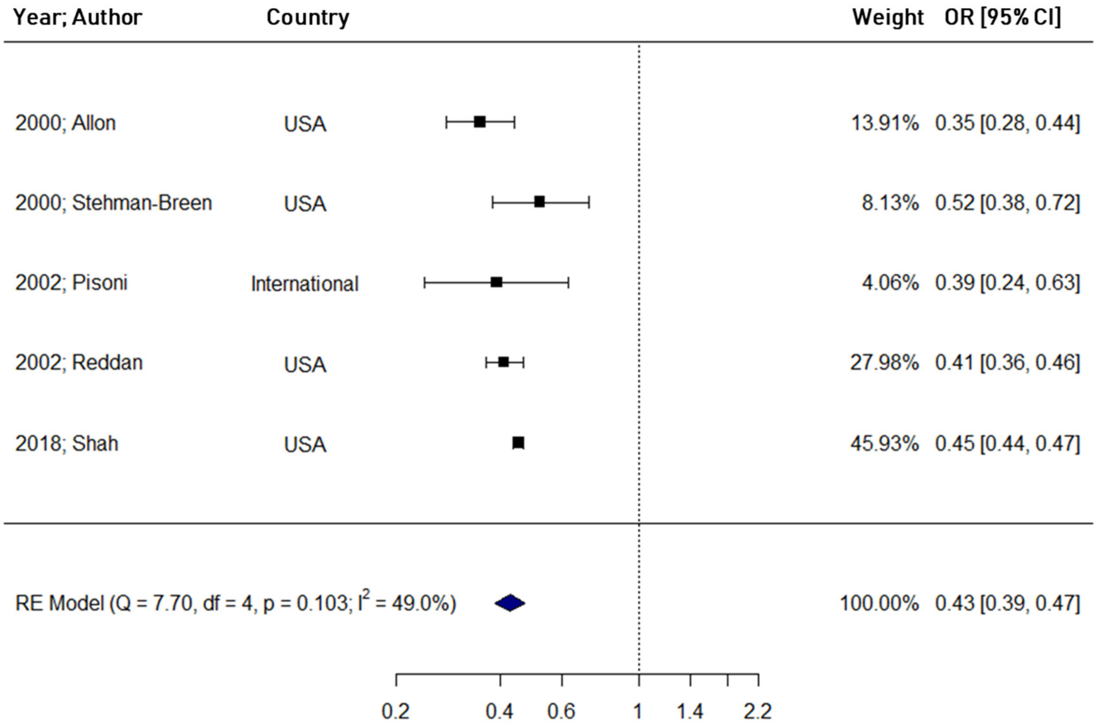

Suppl. Figure S8. Forest plot comparing female and male patients (reference) regarding dialysis initiation with arteriovenous fistula *vs*. arteriovenous graft.

5.3. Successful arteriovenous fistula use

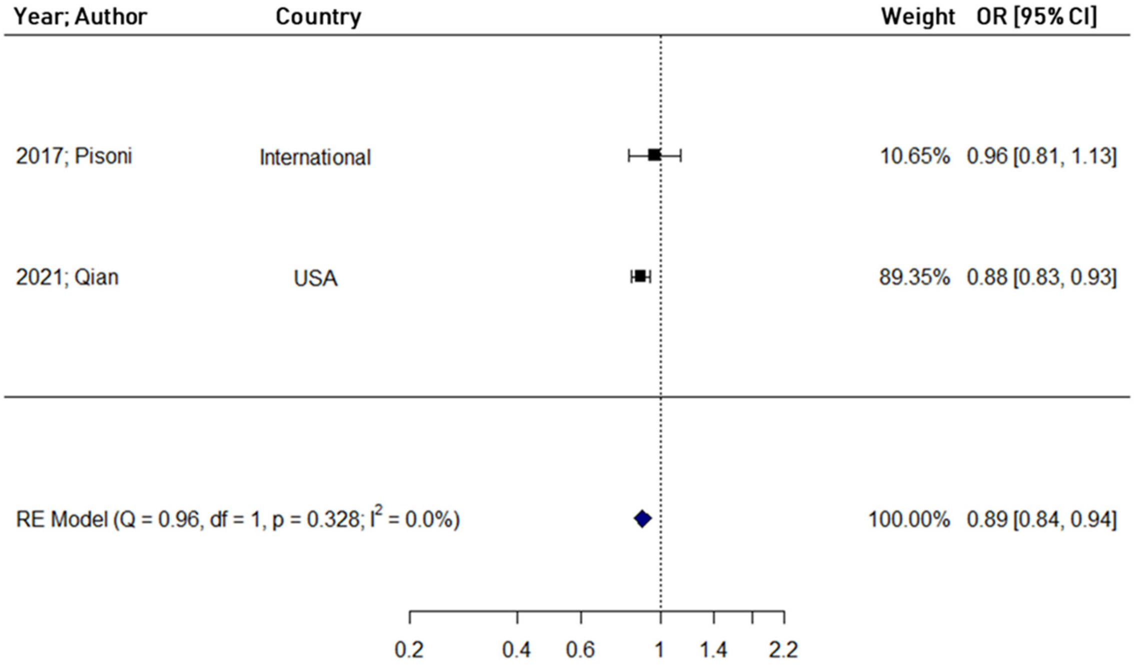

Suppl. Figure S9. Forest plot comparing Black and White patients (reference) regarding successful arteriovenous fistula use.

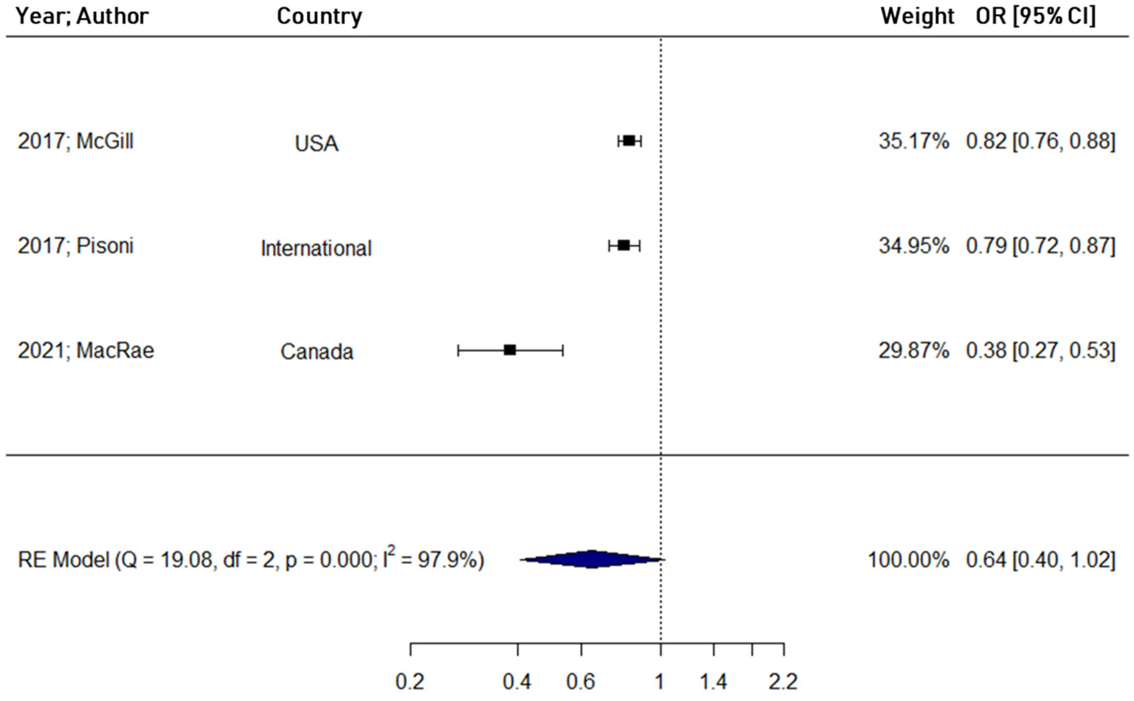

Suppl. Figure S10. Forest plot comparing female and male patients (reference) regarding successful arteriovenous fistula.

5.4. Primary arteriovenous fistula patency

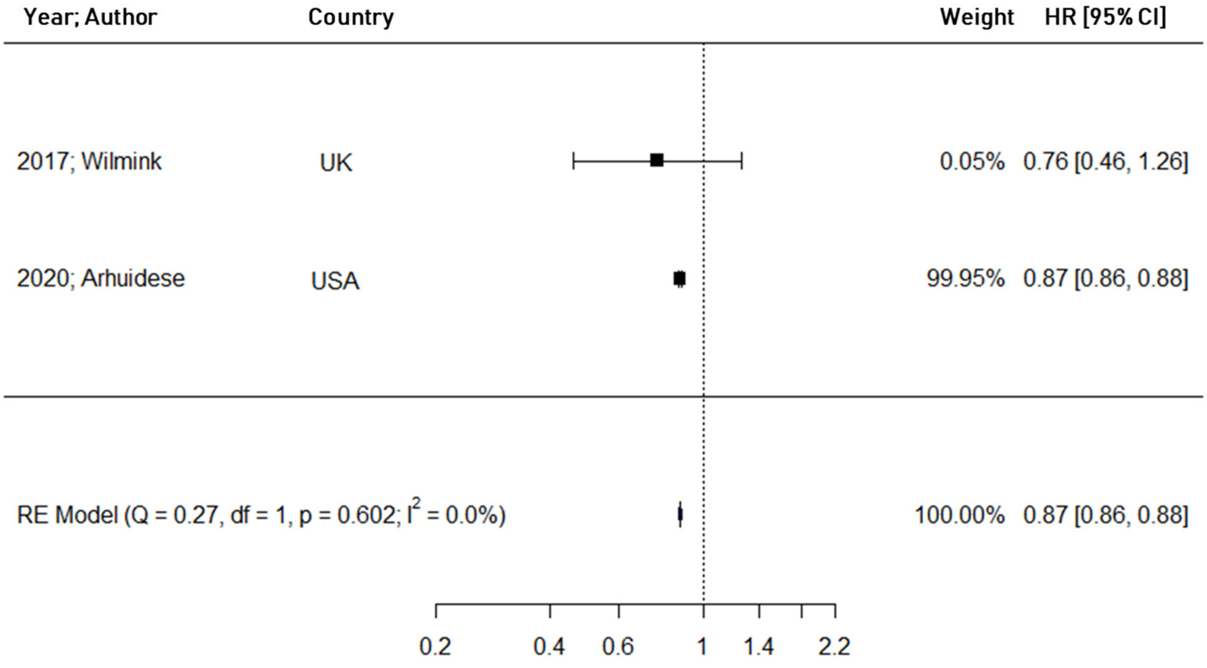

Suppl. Figure S11. Forest plot comparing Black and White patients (reference) regarding primary arteriovenous fistula patency.

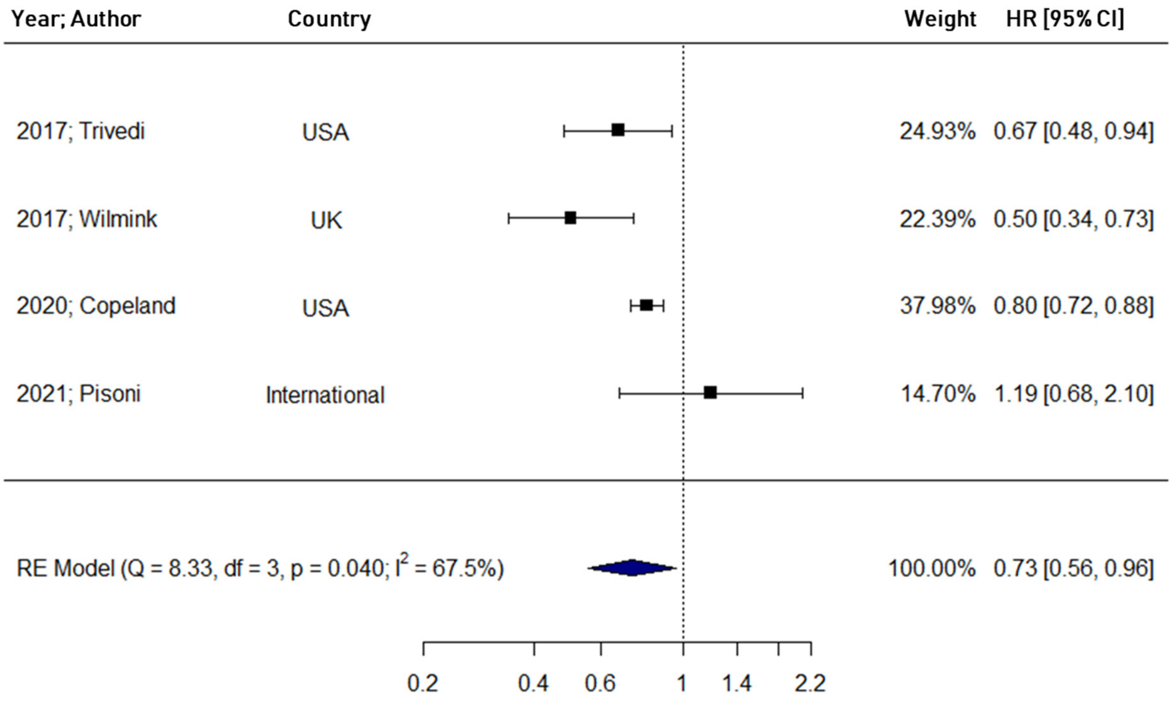

Suppl. Figure S12. Forest plot comparing female and male patients (reference) regarding primary arteriovenous fistula patency.

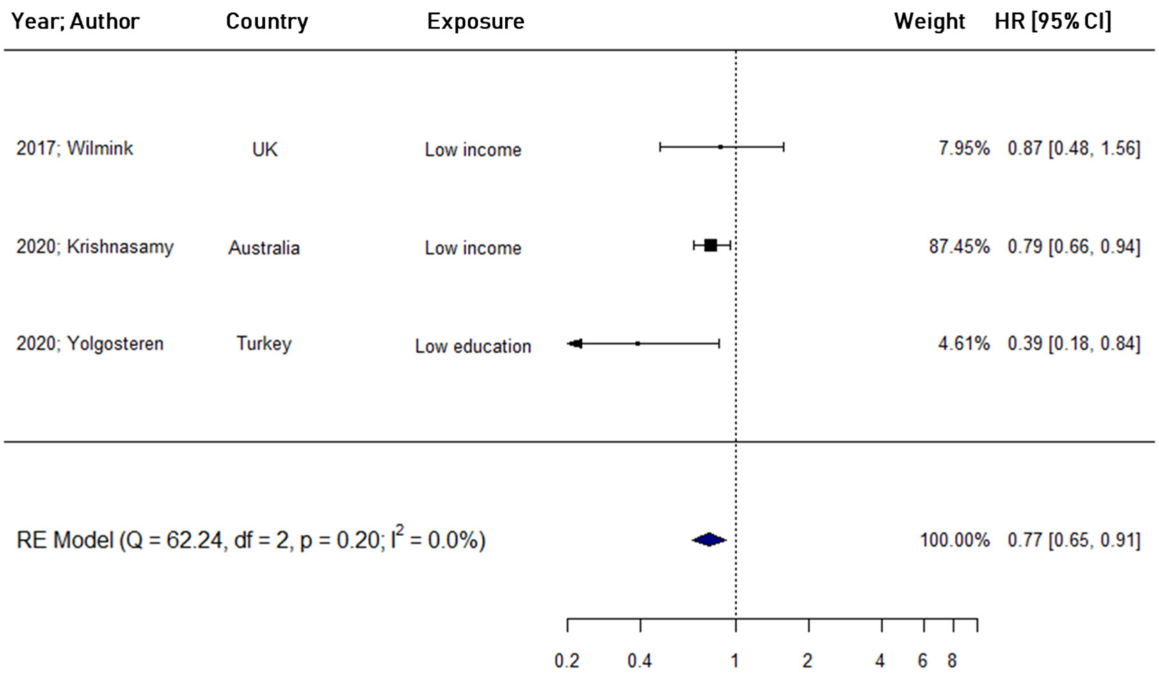

Suppl. Figure S13. Forest plot comparing low and high socioeconomic status patients (reference) regarding primary arteriovenous fistula patency.

5.5. Transition from central venous catheter to arteriovenous fistula/graft

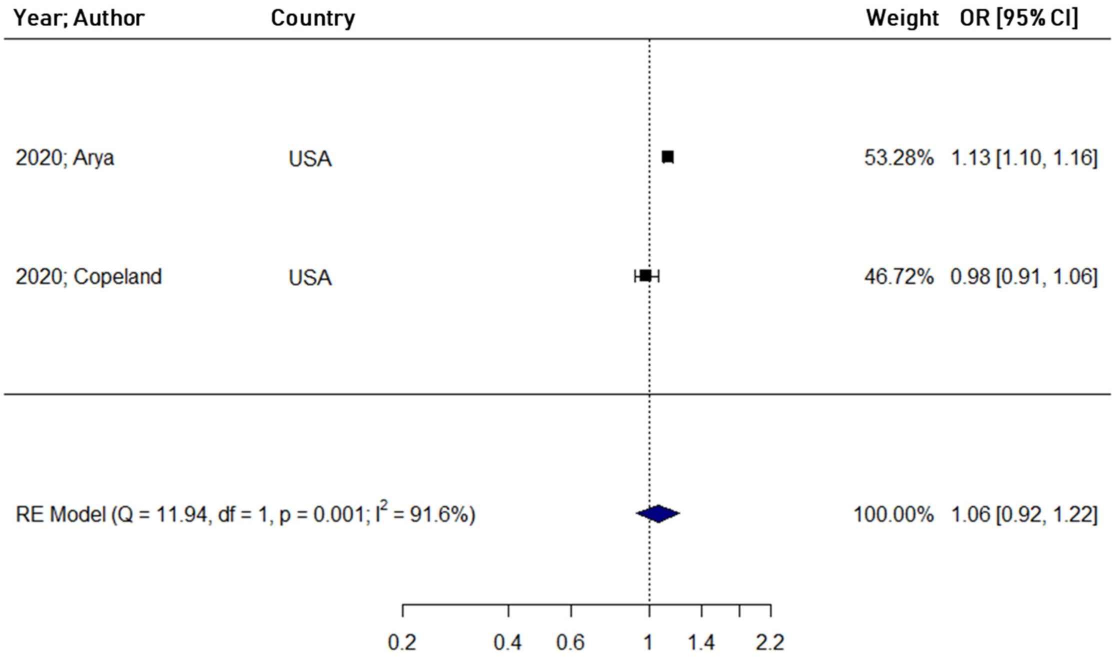

Suppl. Figure S14. Forest plot comparing Black and White patients (reference) regarding the transition from central venous catheter to arteriovenous fistula/graft.

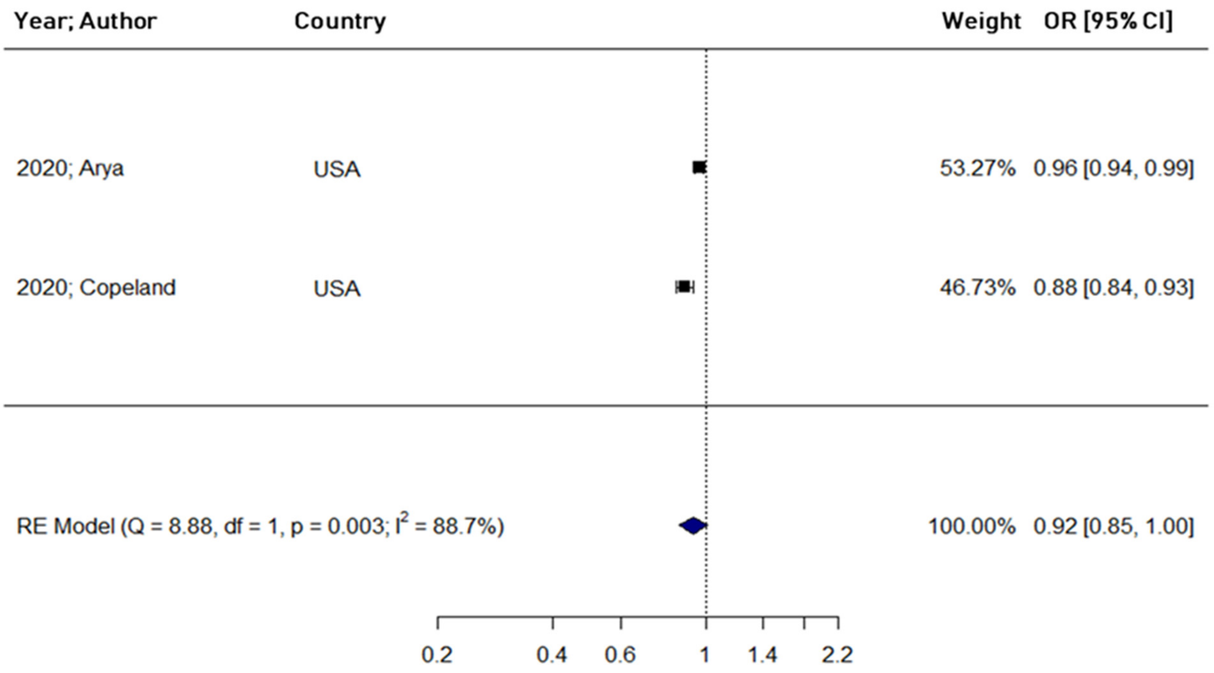

Suppl. Figure S15. Forest plot comparing female and male patients (reference) regarding the transition from central venous catheter to arteriovenous fistula/graft.

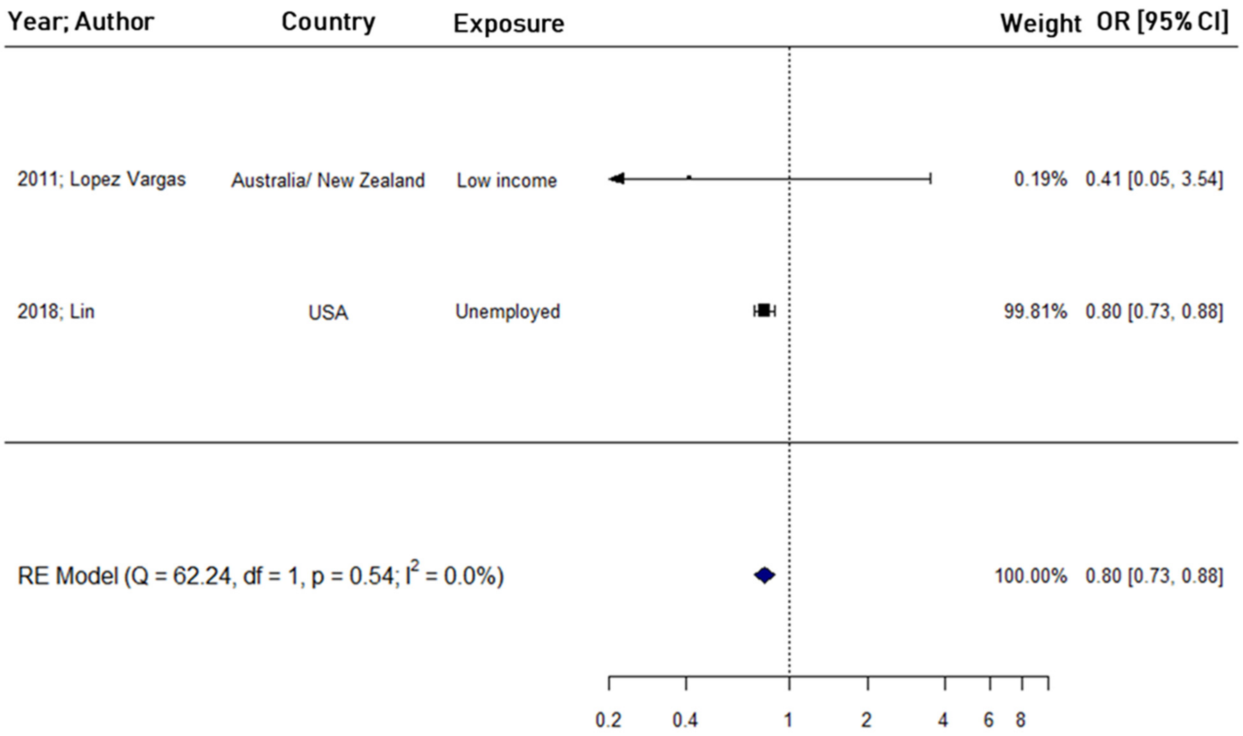

Suppl. Figure S16. Forest plot comparing low and high socioeconomic status patients (reference) the transition from central venous catheter to arteriovenous fistula/graft.

5.6. Home dialysis

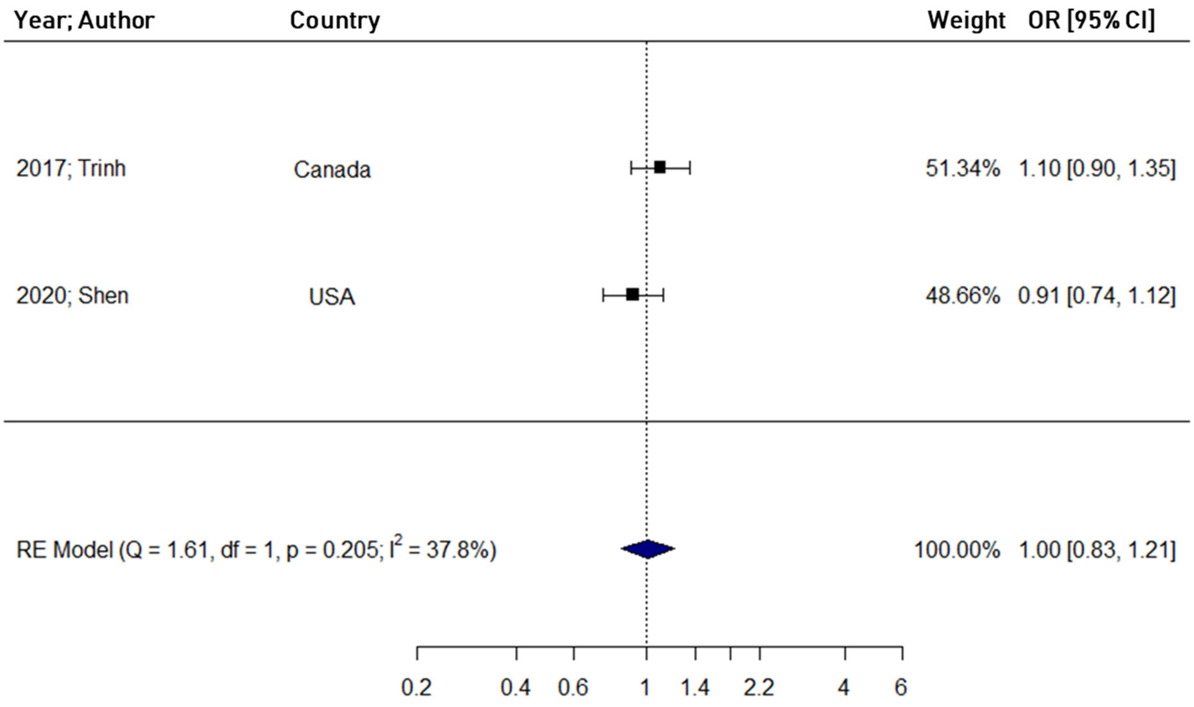

Suppl. Figure S17. Forest plot comparing Black and White patients (reference) regarding home dialysis.

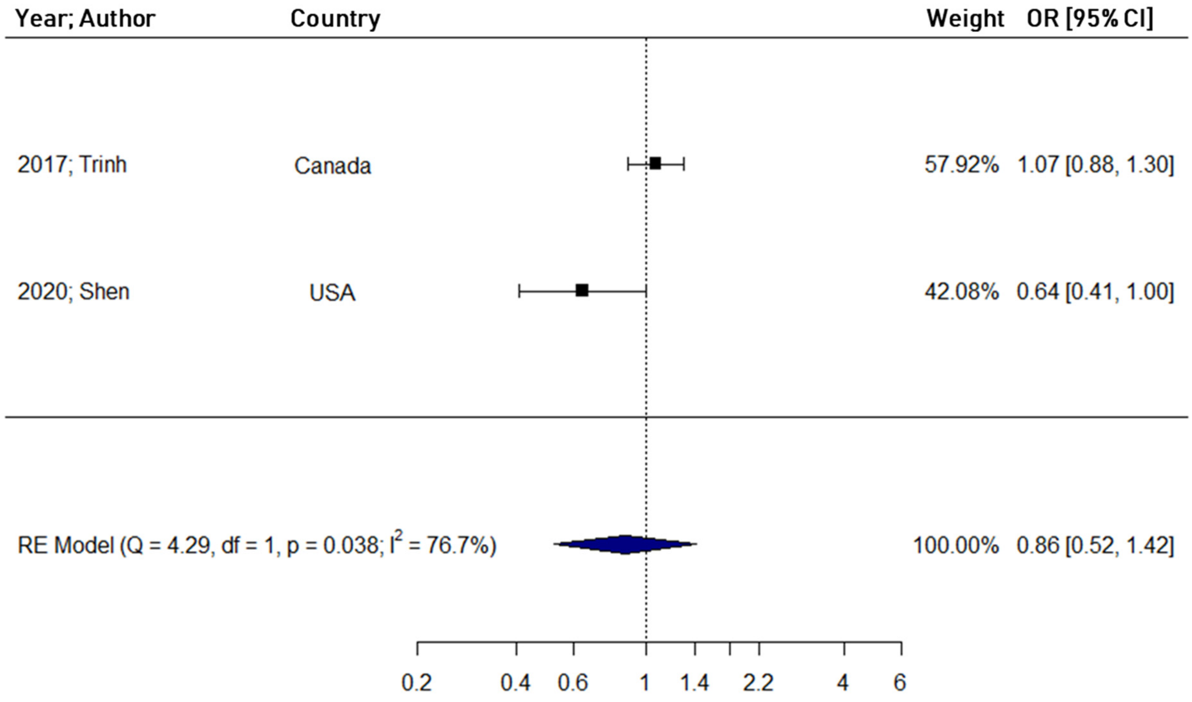

Suppl. Figure S18. Forest plot comparing Asian and White patients (reference) regarding home dialysis.

5.7. Peritoneal dialysis

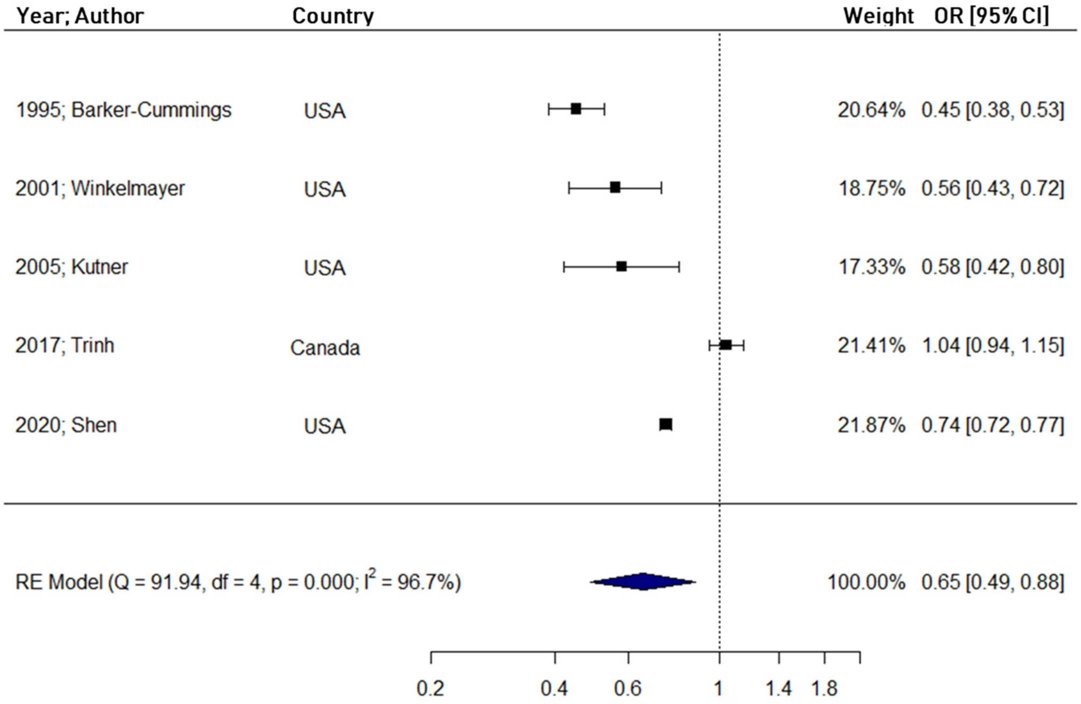

Suppl. Figure S19. Forest plot comparing Black and White patients (reference) regarding peritoneal dialysis use.

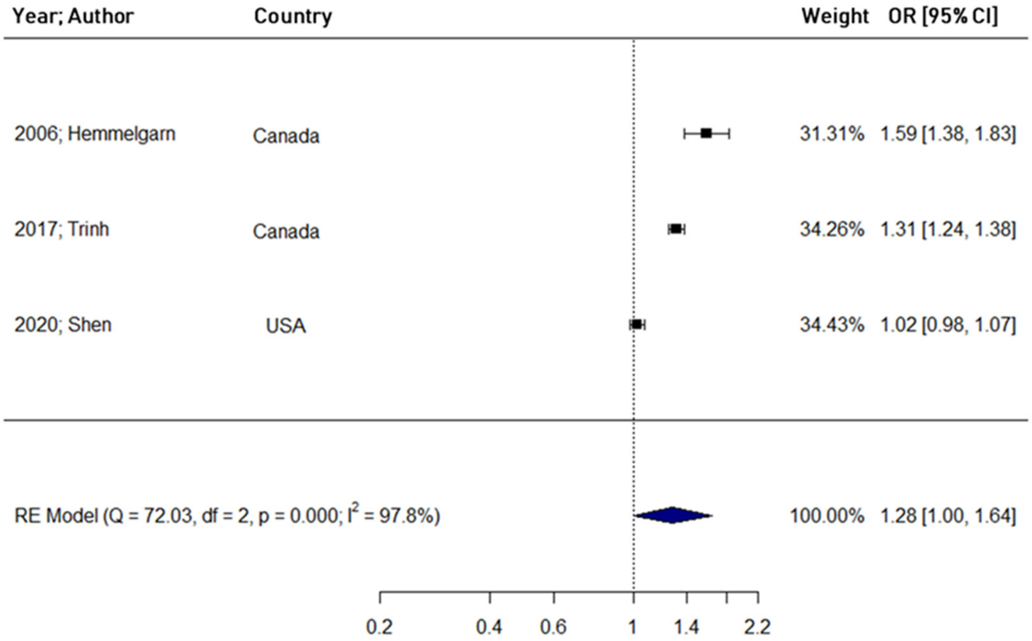

Suppl. Figure S20. Forest plot comparing Asian and White patients (reference) regarding peritoneal dialysis use.

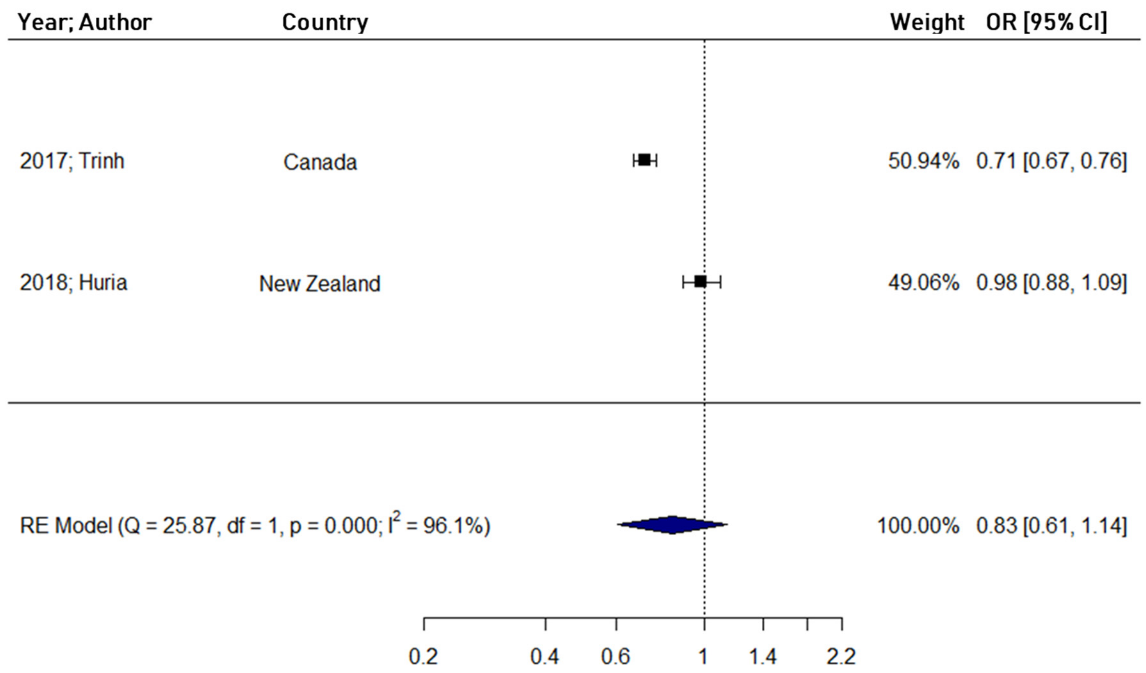

Suppl. Figure S21. Forest plot comparing Indigenous and White patients (reference) regarding peritoneal dialysis use.

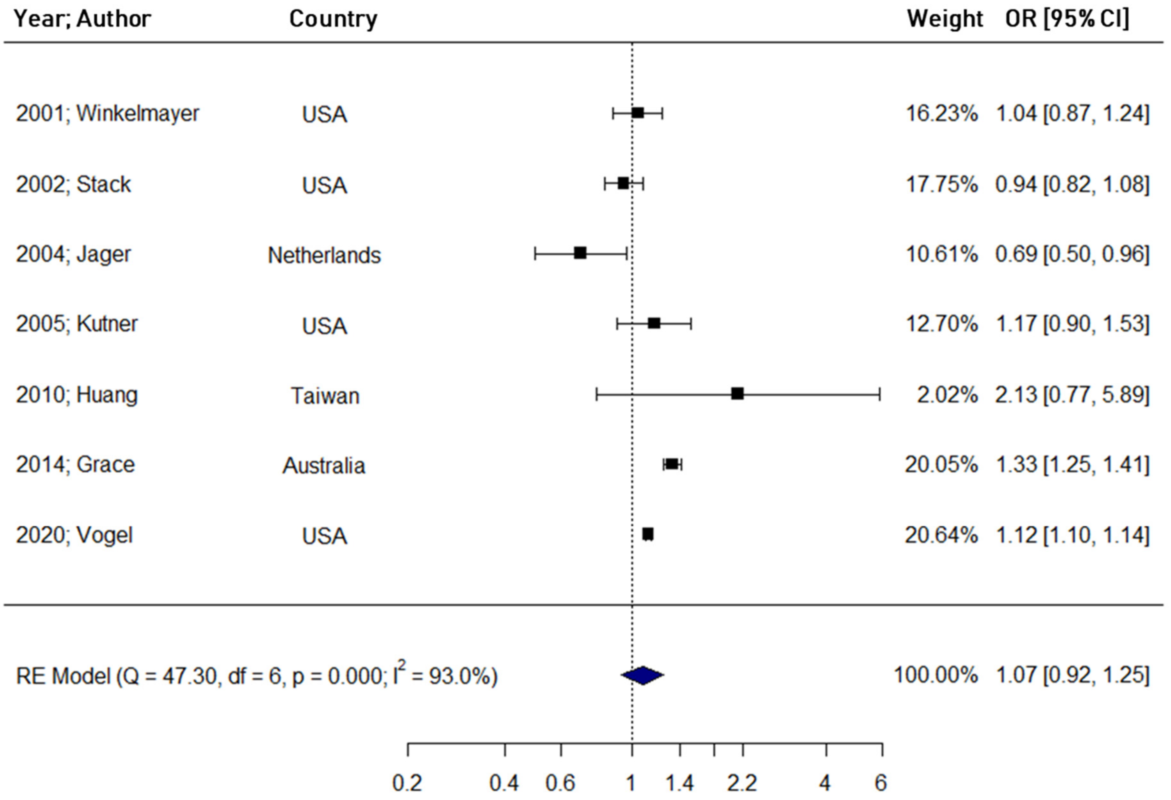

Suppl. Figure S22. Forest plot comparing female and male patients (reference) regarding peritoneal dialysis use.

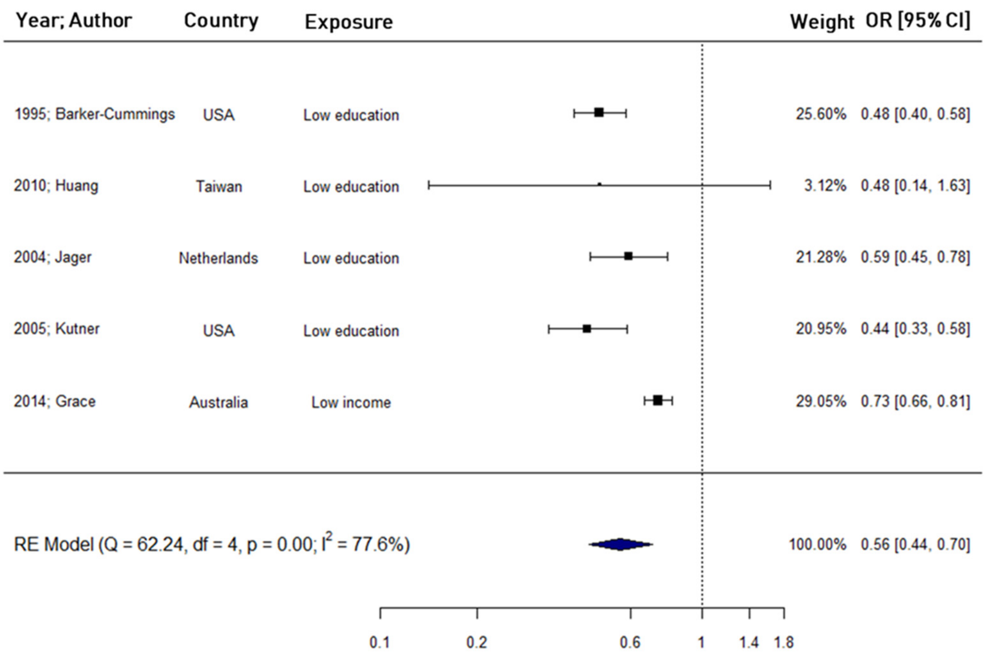

Suppl. Figure S23. Forest plot comparing low and high socioeconomic status patients (reference) regarding peritoneal dialysis use.

5.8. Dialysis survival

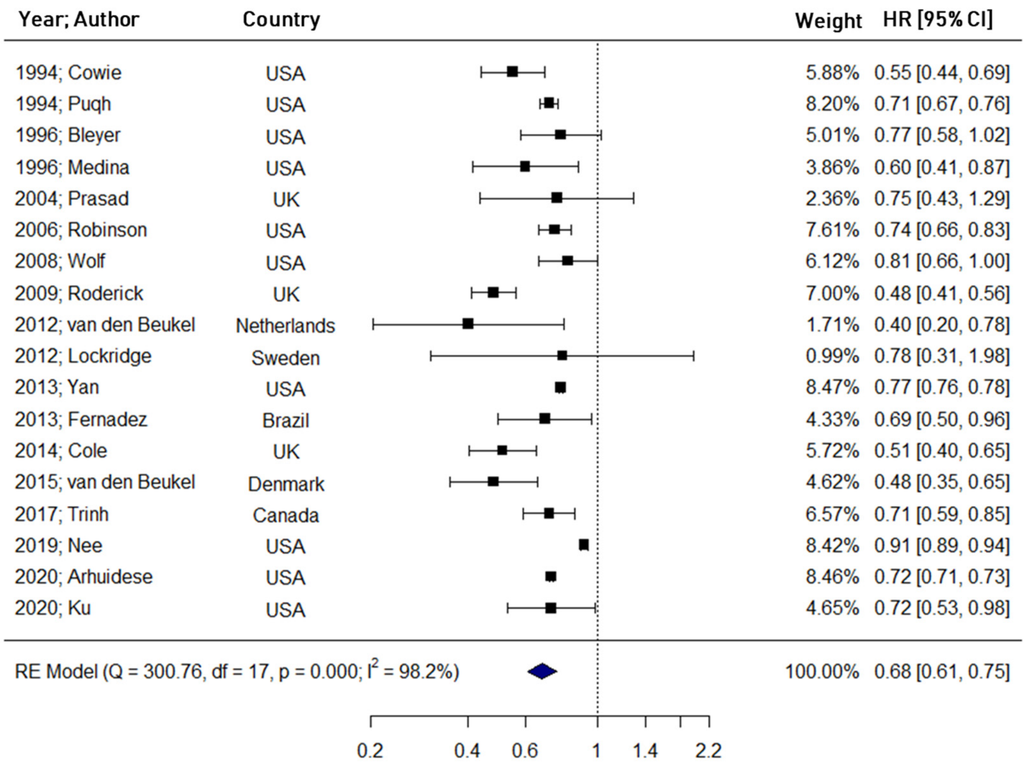

Suppl. Figure S24. Forest plot comparing Black and White patients (reference) regarding dialysis mortality.

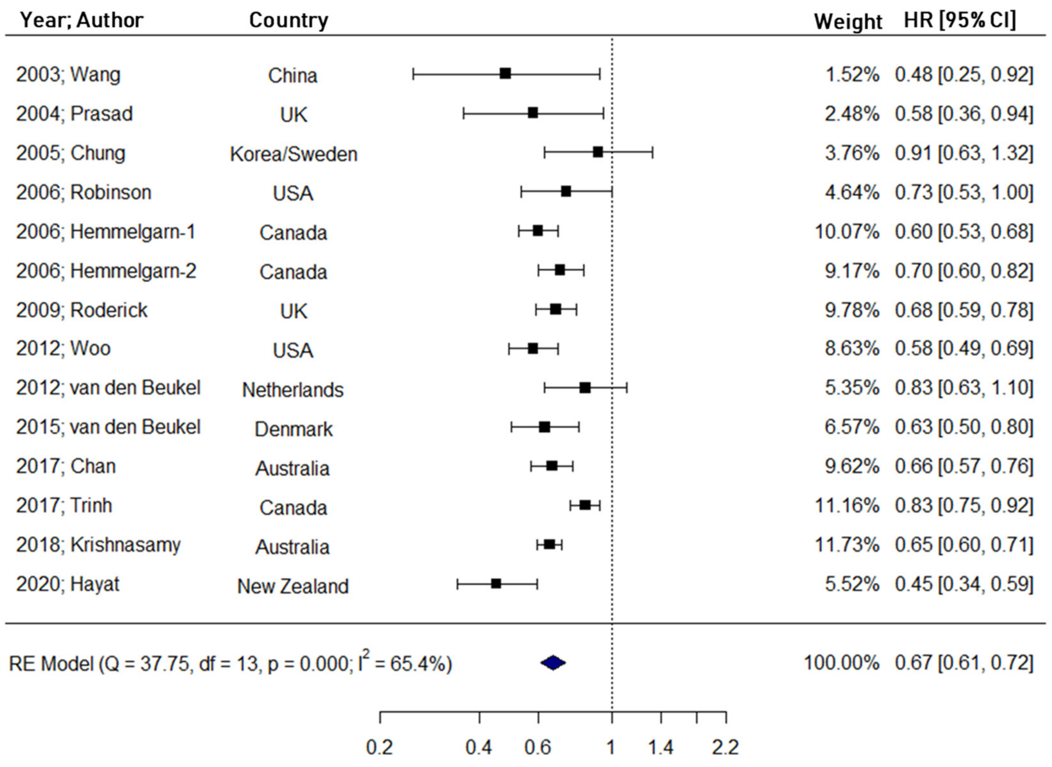

Suppl. Figure S25. Forest plot comparing Asian and White patients (reference) regarding dialysis mortality.

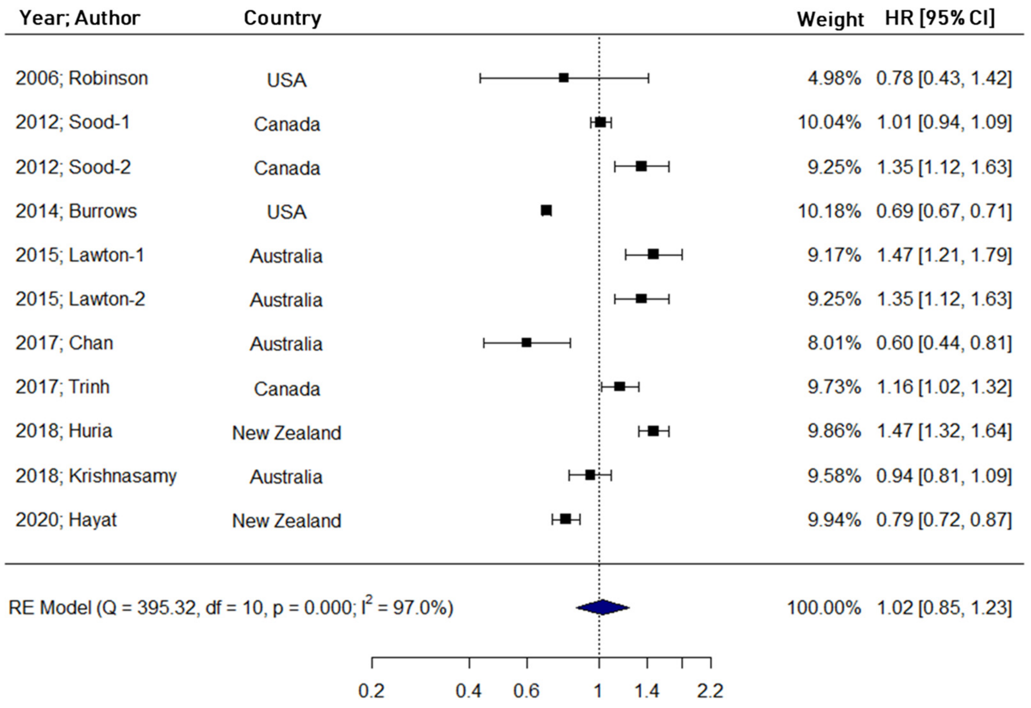

Suppl. Figure S26. Forest plot comparing Indigenous and White patients (reference) regarding dialysis mortality.

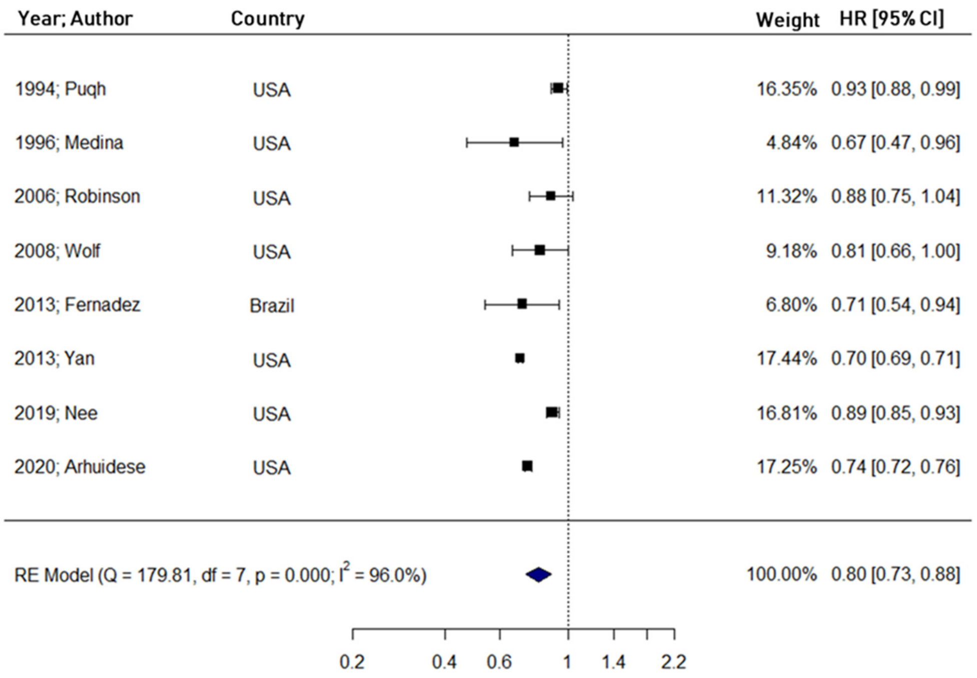

Suppl. Figure S27. Forest plot of comparing Hispanic and White patients (reference) regarding dialysis mortality.

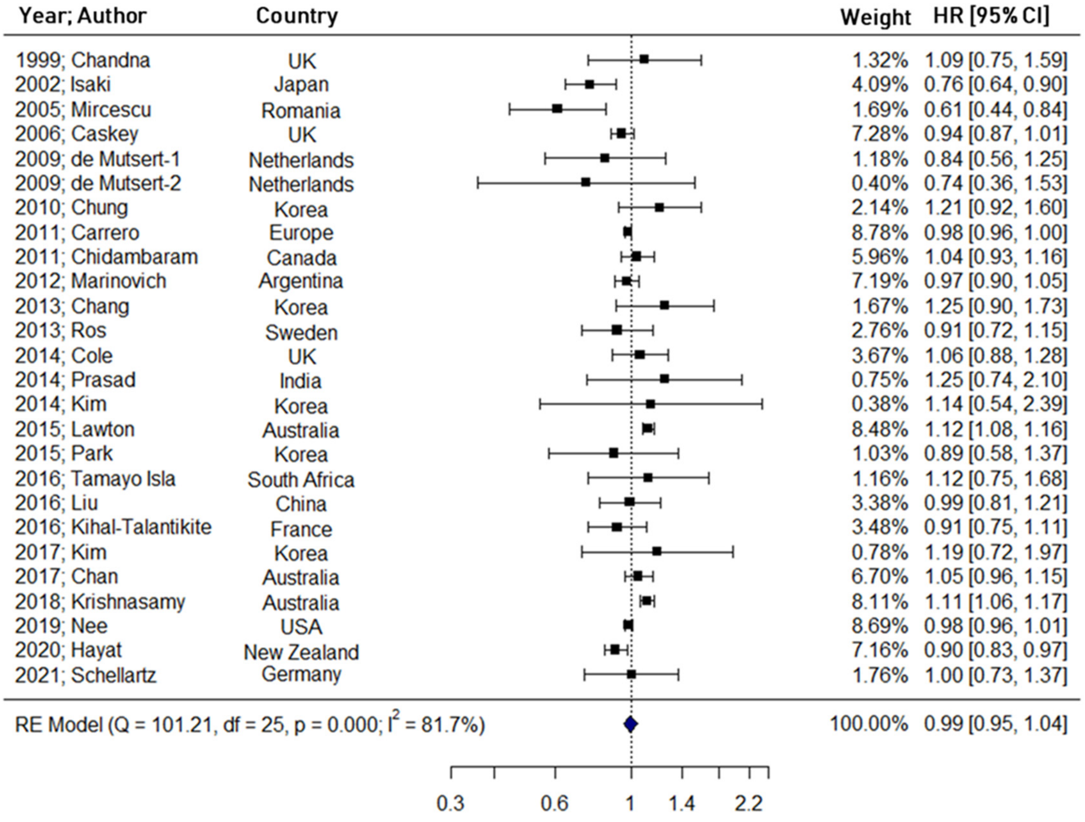

Suppl. Figure S28. Forest plot comparing female and male patients (reference) regarding dialysis mortality.

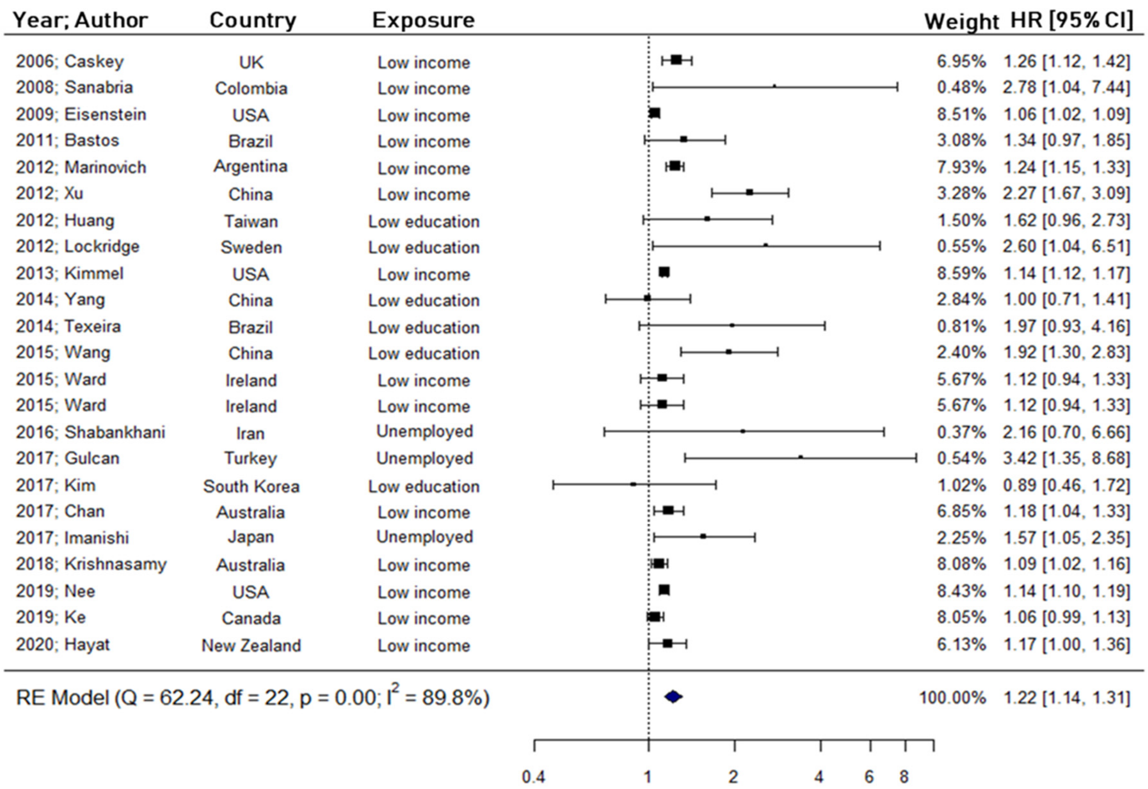

Suppl. Figure S29. Forest plot comparing low and high socioeconomic status patients (reference) regarding dialysis mortality.

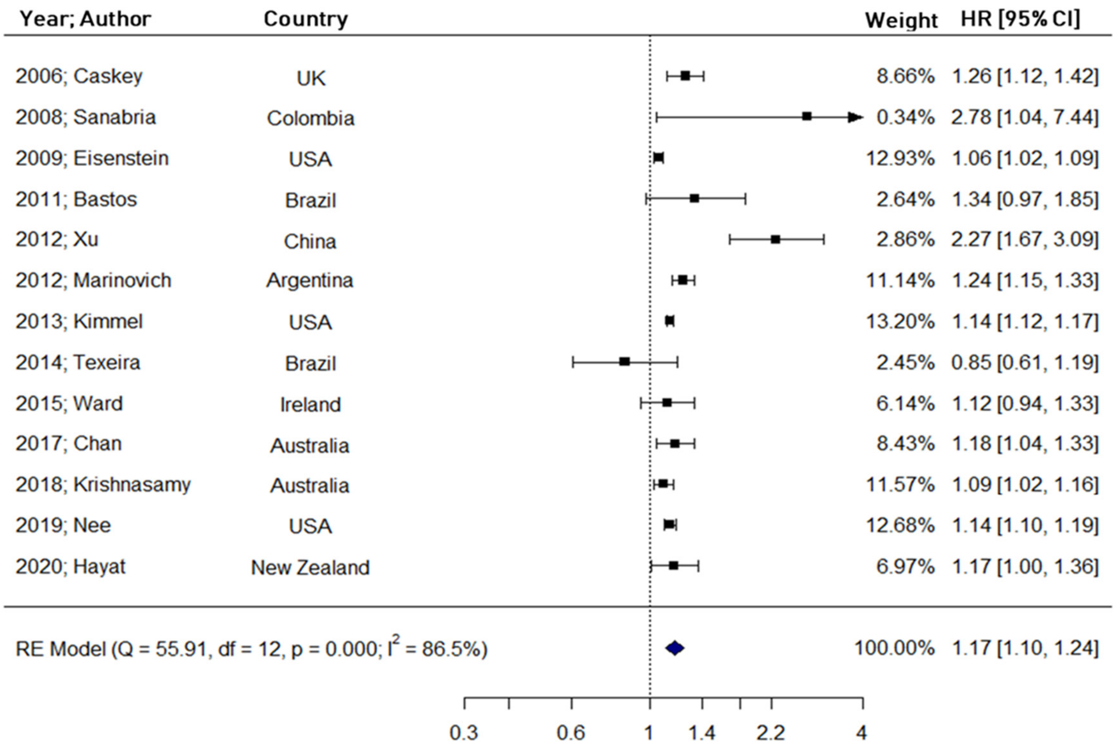

Suppl. Figure S30. Forest plot comparing low- and high-income patients (reference) regarding dialysis mortality.

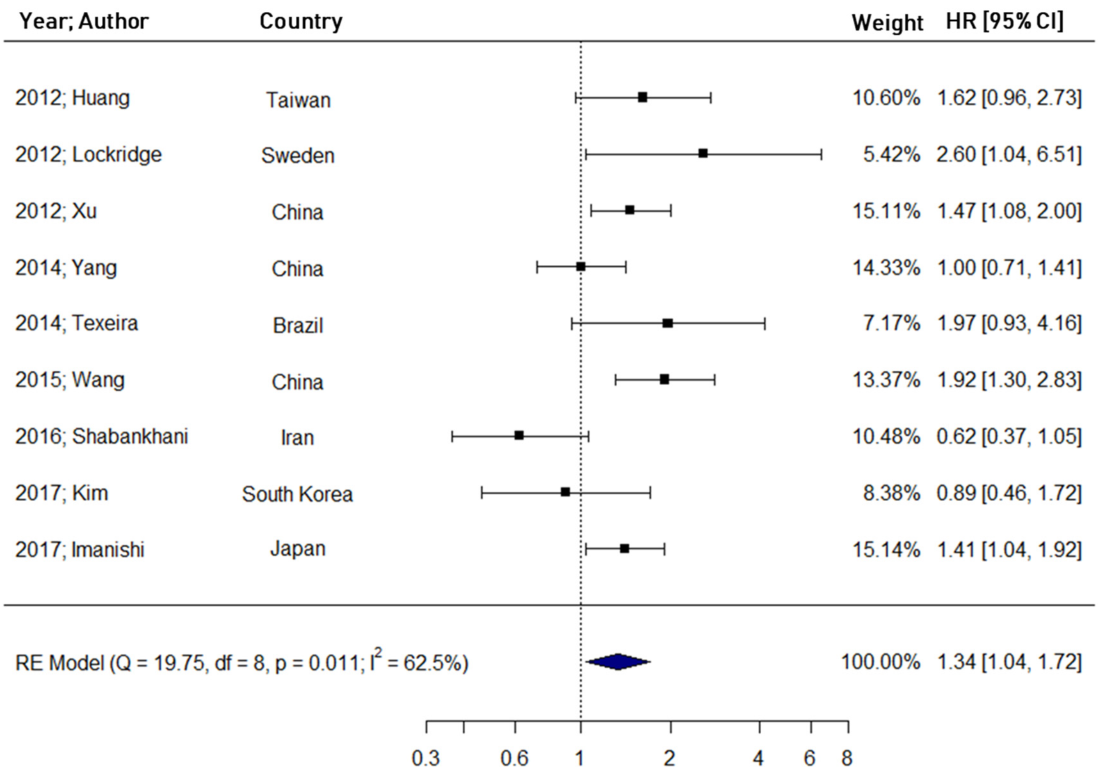

Suppl. Figure S31. Forest plot comparing low and high education patients (reference) regarding on dialysis mortality.

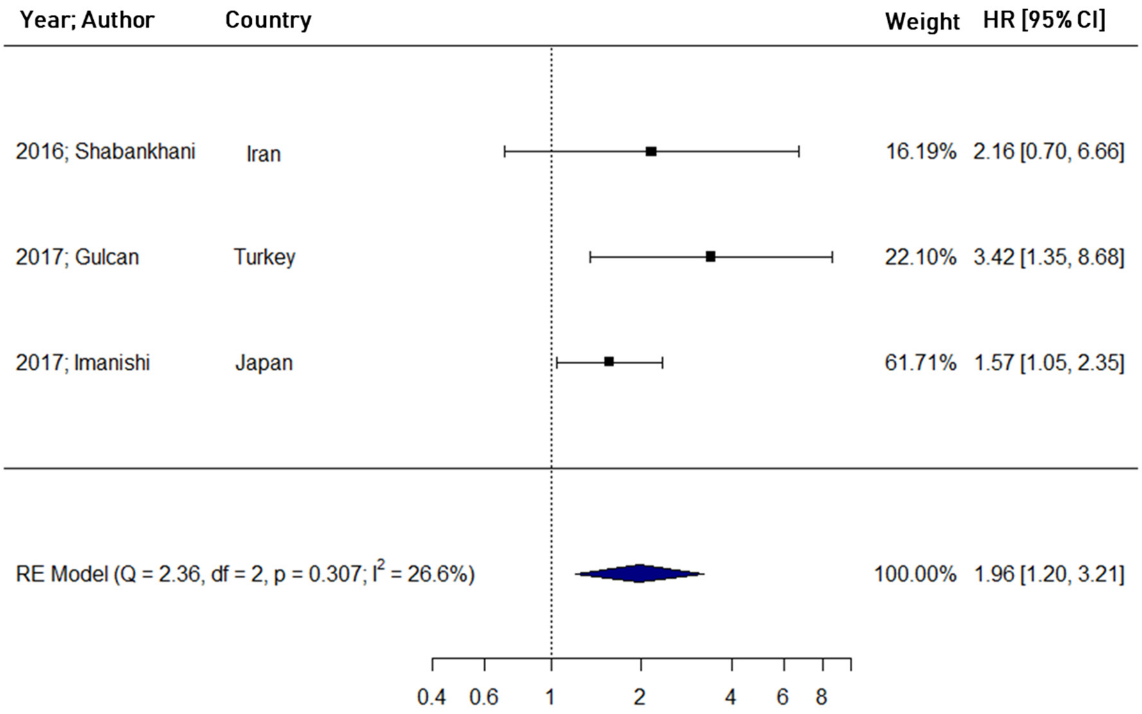

Suppl. Figure S32. Forest plot comparing unemployed and employed patients (reference) regarding dialysis mortality.

5.9. Waitlisting

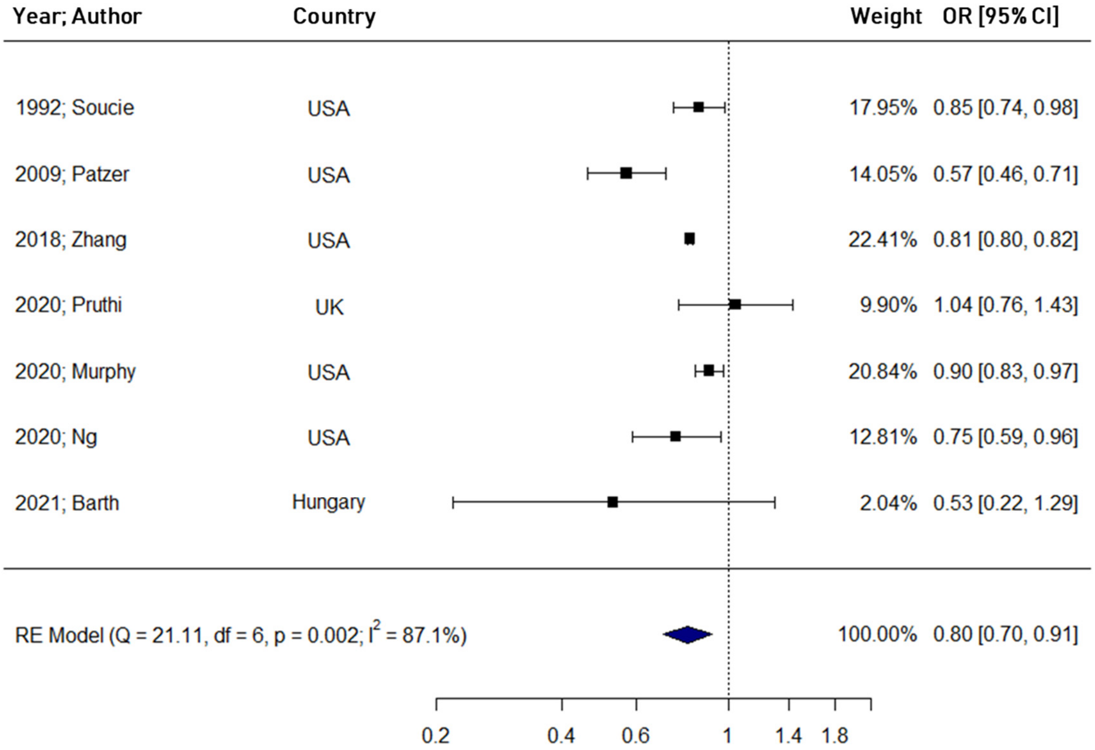

Suppl. Figure S33. Forest plot comparing Black and White patients (reference) regarding waitlisting.

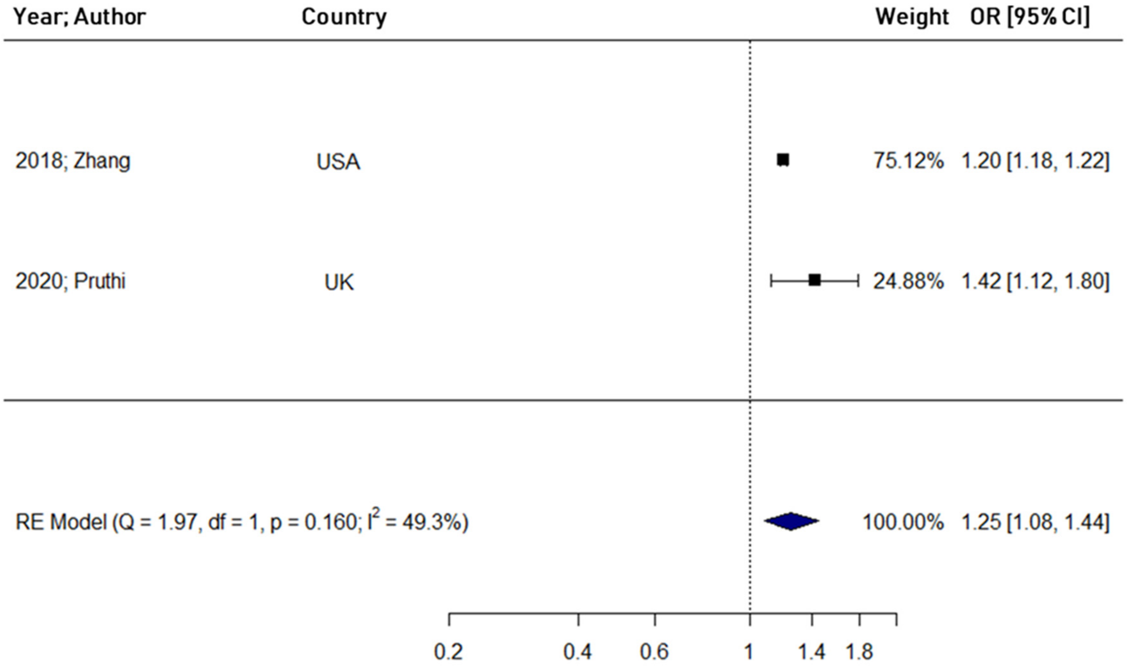

Suppl. Figure S34. Forest plot comparing Asian and White patients (reference) regarding waitlisting.

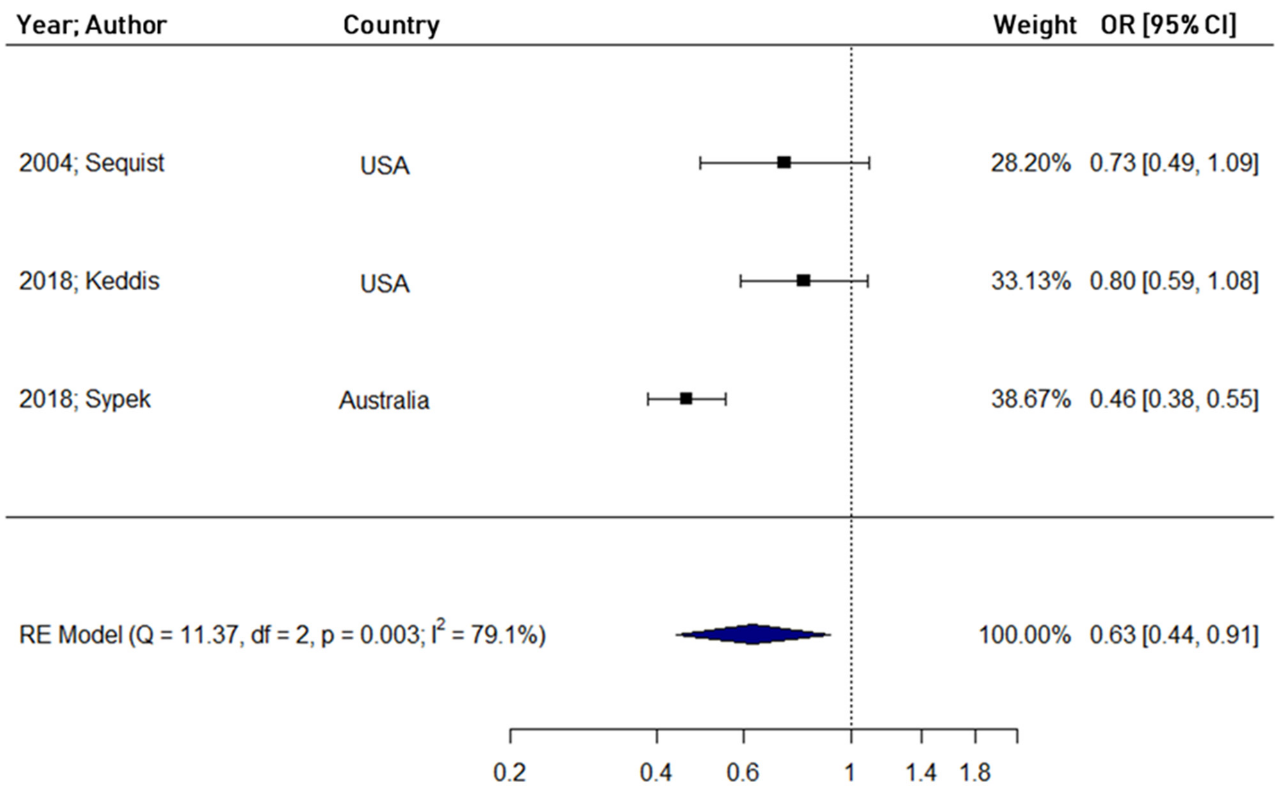

Suppl. Figure S35. Forest plot comparing Indigenous and White patients (reference) regarding waitlisting.

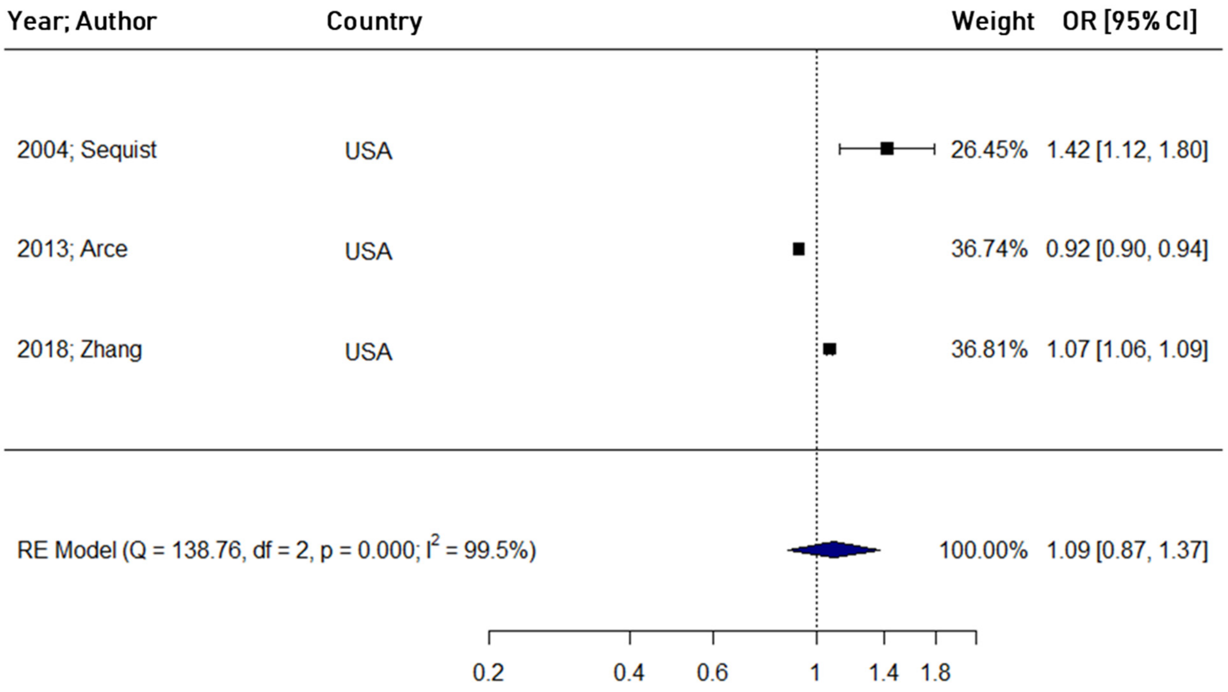

Suppl. Figure S36. Forest plot comparing Hispanic and White patients (reference) regarding waitlisting.

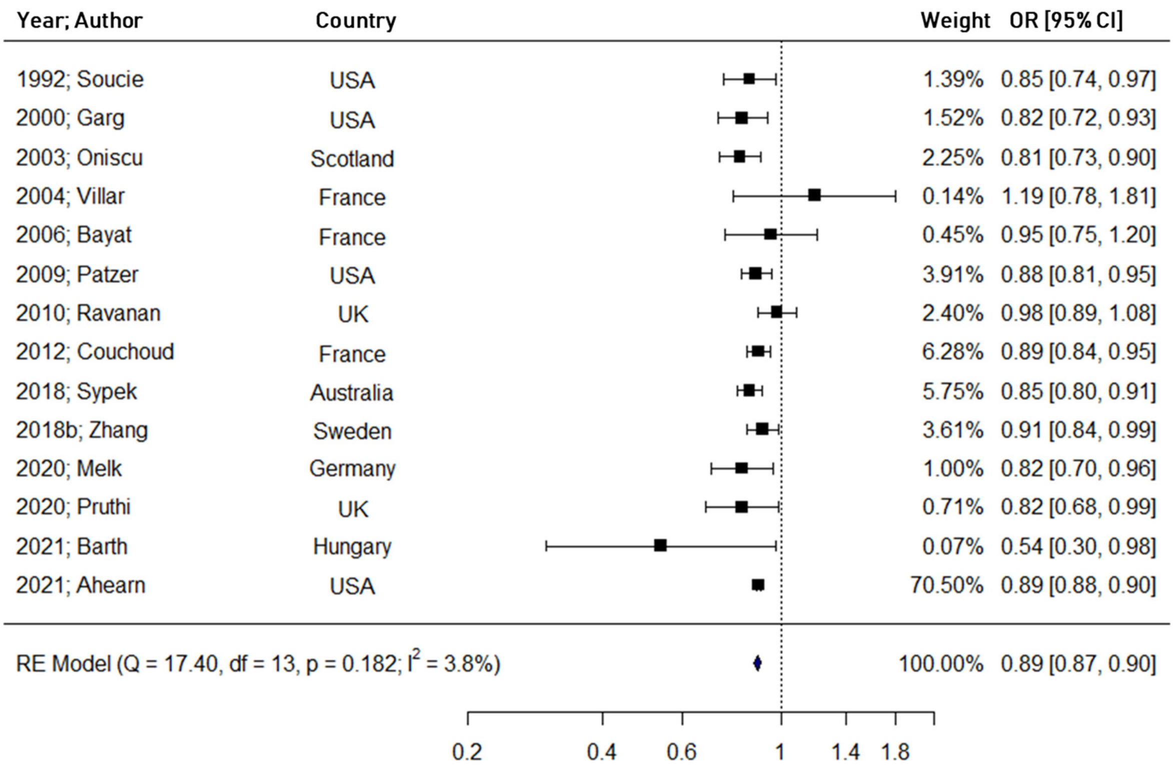

Suppl. Figure S37. Forest plot comparing female and male patients (reference) regarding waitlisting.

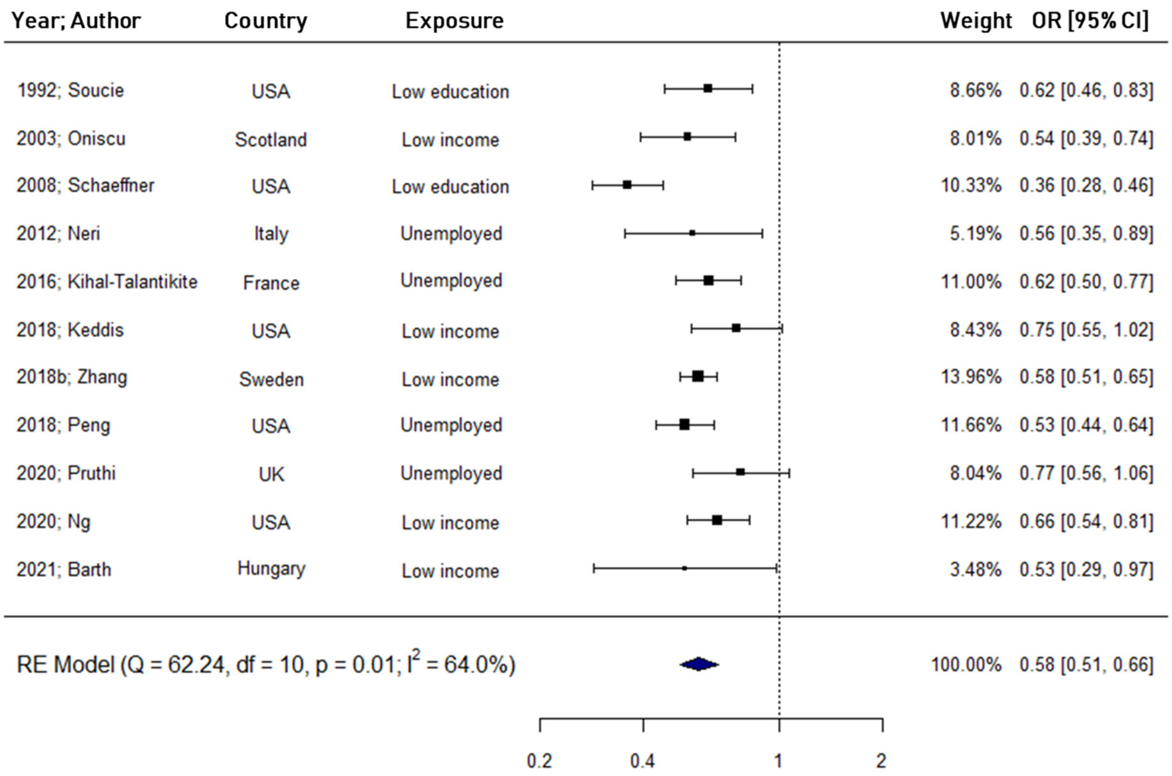

Suppl. Figure S38. Forest plot comparing low and high socioeconomic status patients (reference) regarding waitlisting.

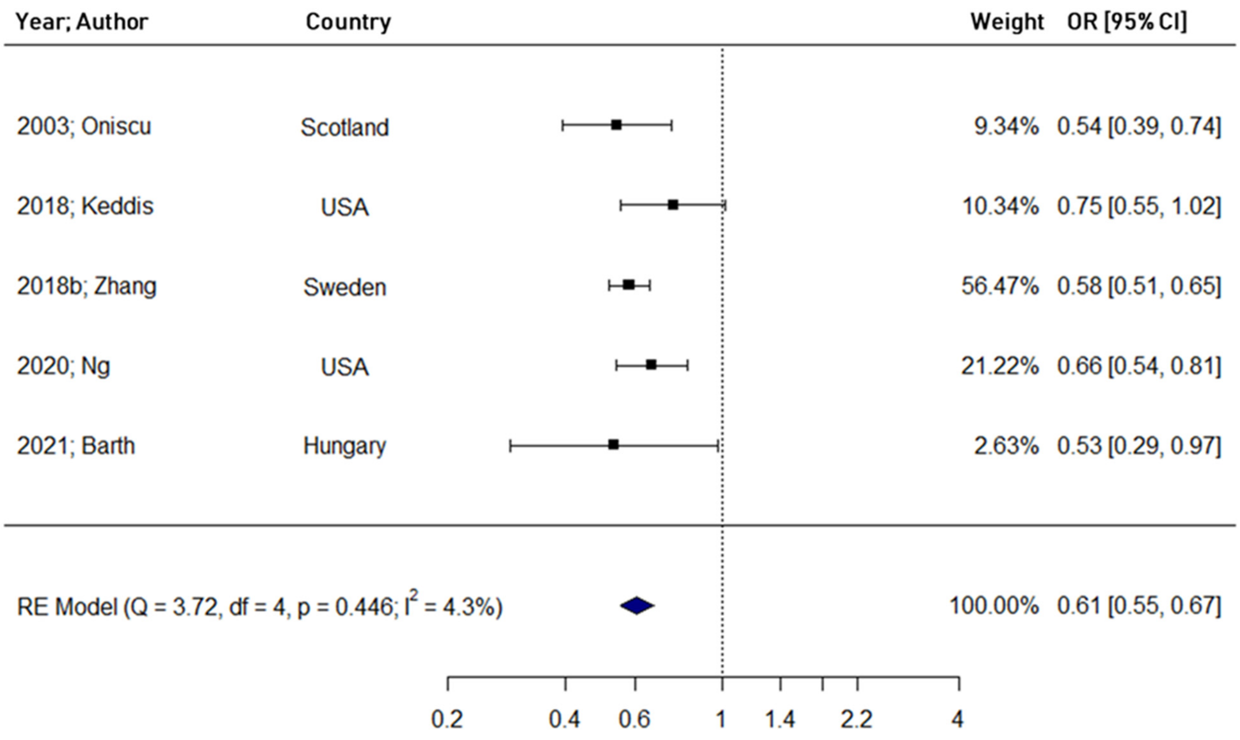

Suppl. Figure S39. Forest plot comparing low- and high-income patients (reference) regarding waitlisting.

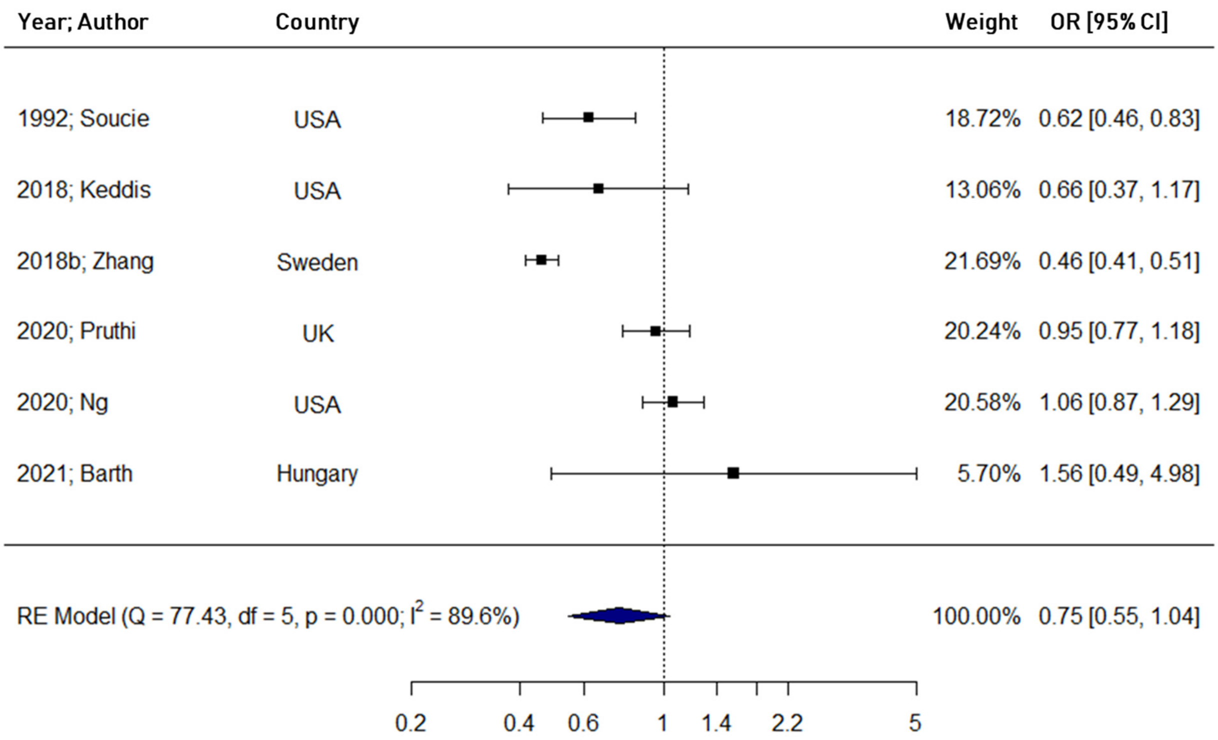

Suppl. Figure S40. Forest plot comparing low and high education patients (reference) regarding waitlisting.

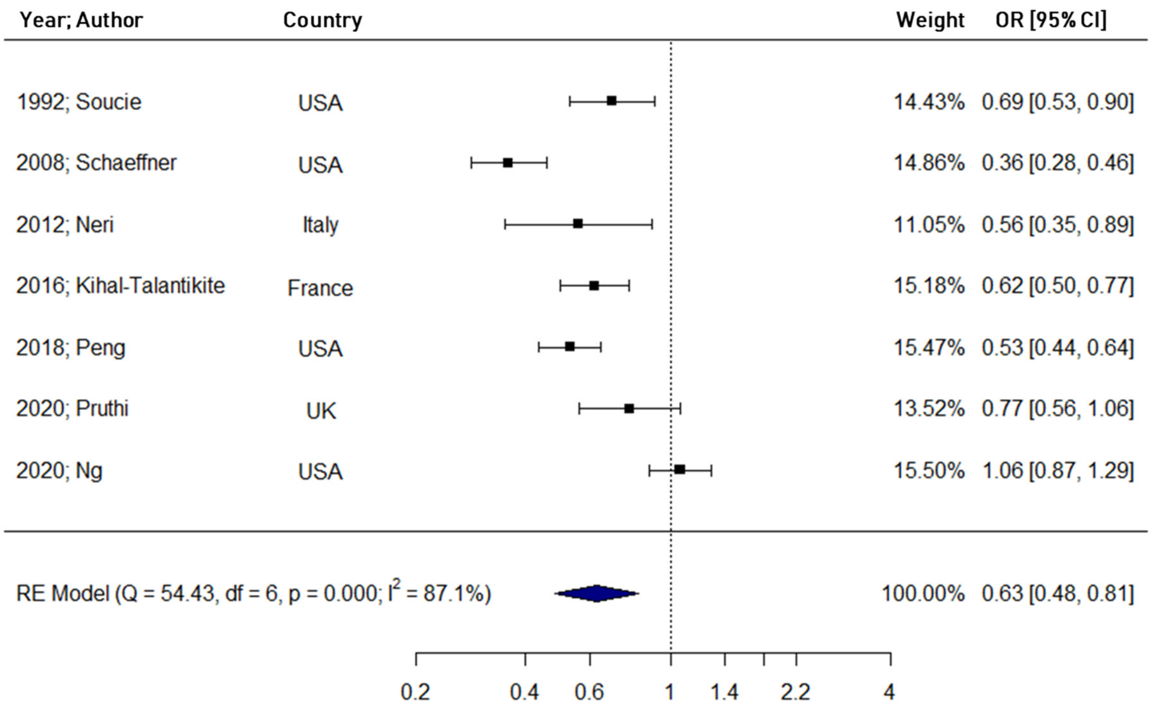

Suppl. Figure S41. Forest plot comparing unemployed and employed patients (reference) regarding on waitlisting.

5.10. Kidney transplantation

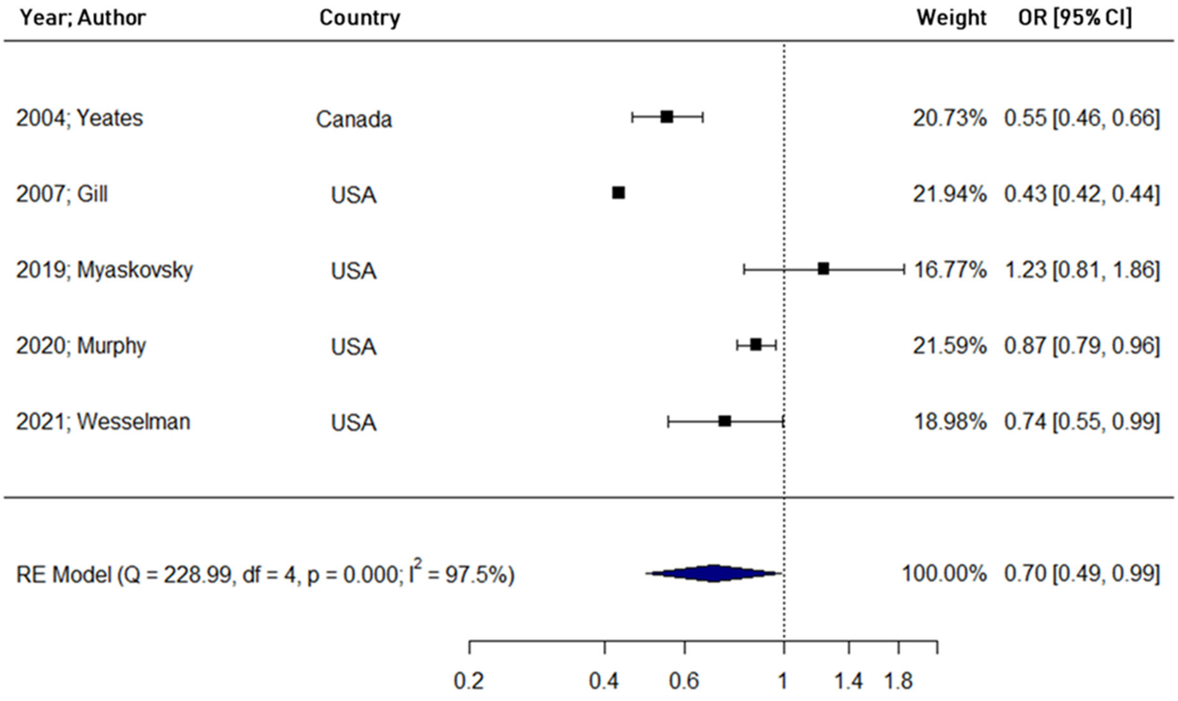

Suppl. Figure S42. Forest plot comparing Black and White patients (reference) regarding kidney transplantation.

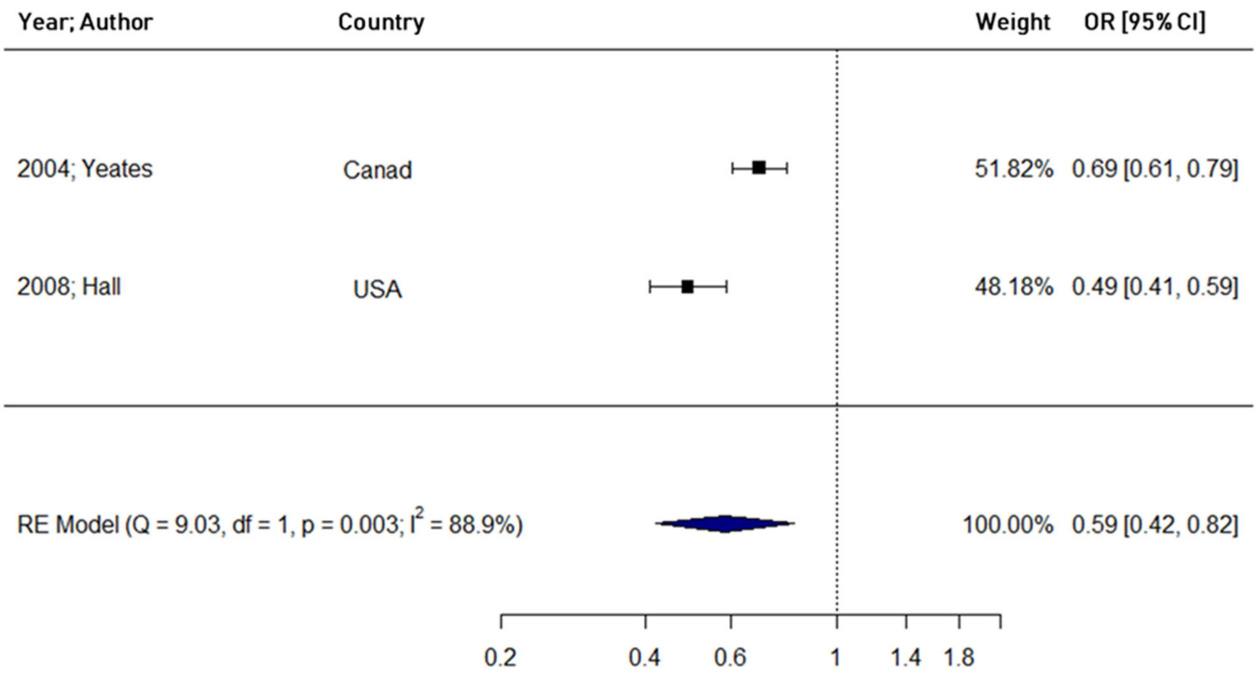

Suppl. Figure S43. Forest plot comparing Asian and White patients (reference) regarding kidney transplantation.

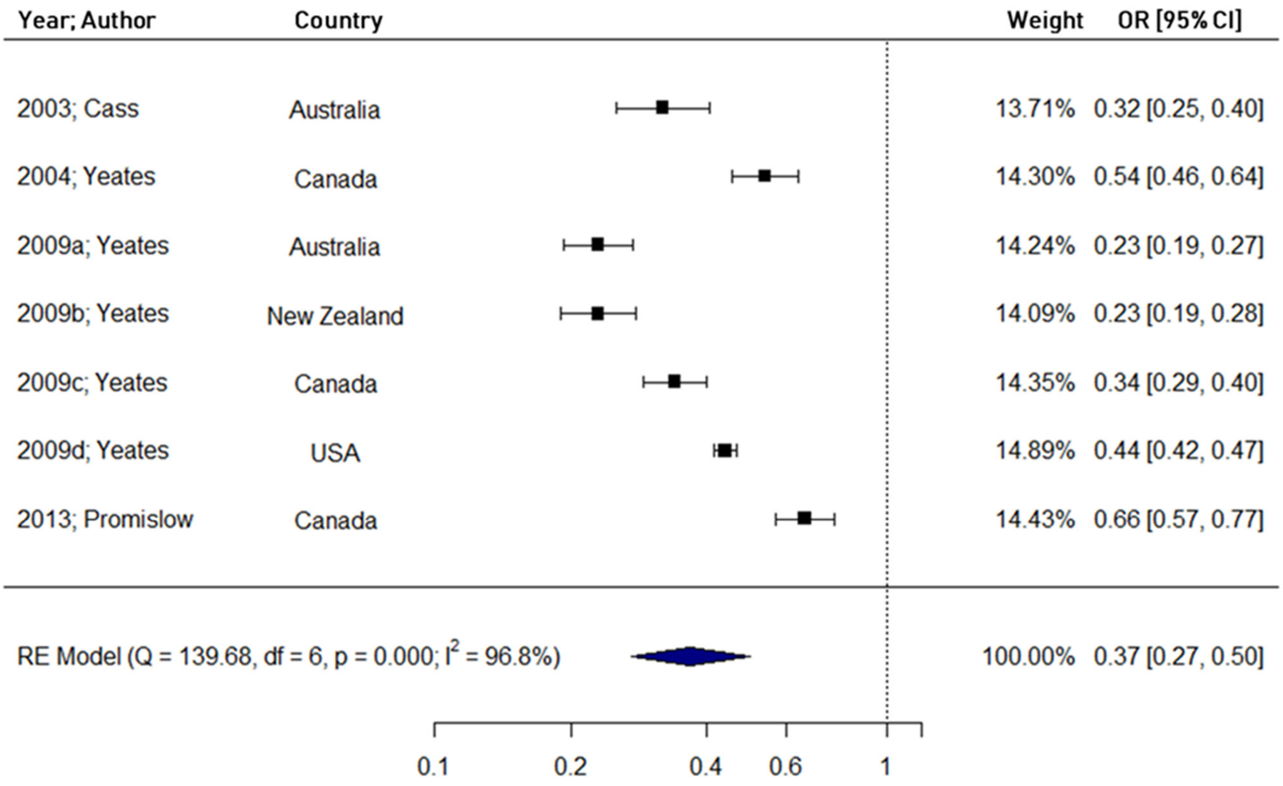

Suppl. Figure S44. Forest plot comparing Indigenous and White patients (reference) regarding kidney transplantation.

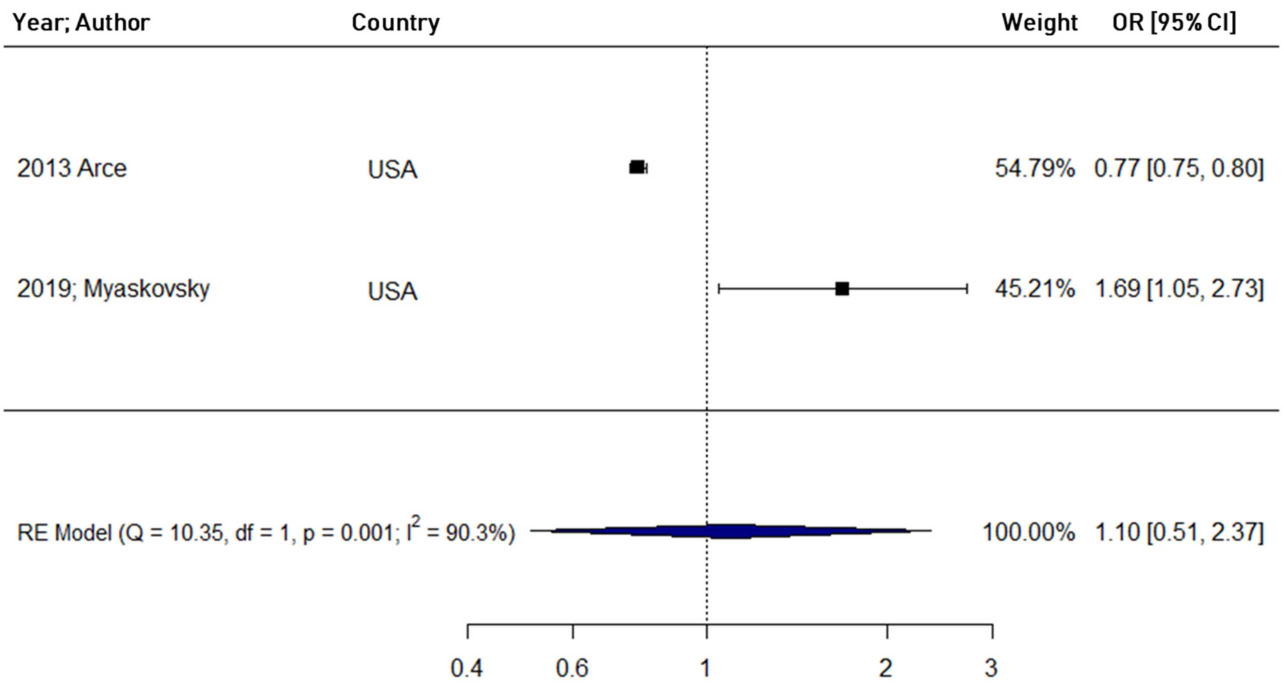

Suppl. Figure S45. Forest plot comparing Hispanic and White patients (reference) regarding kidney transplantation.

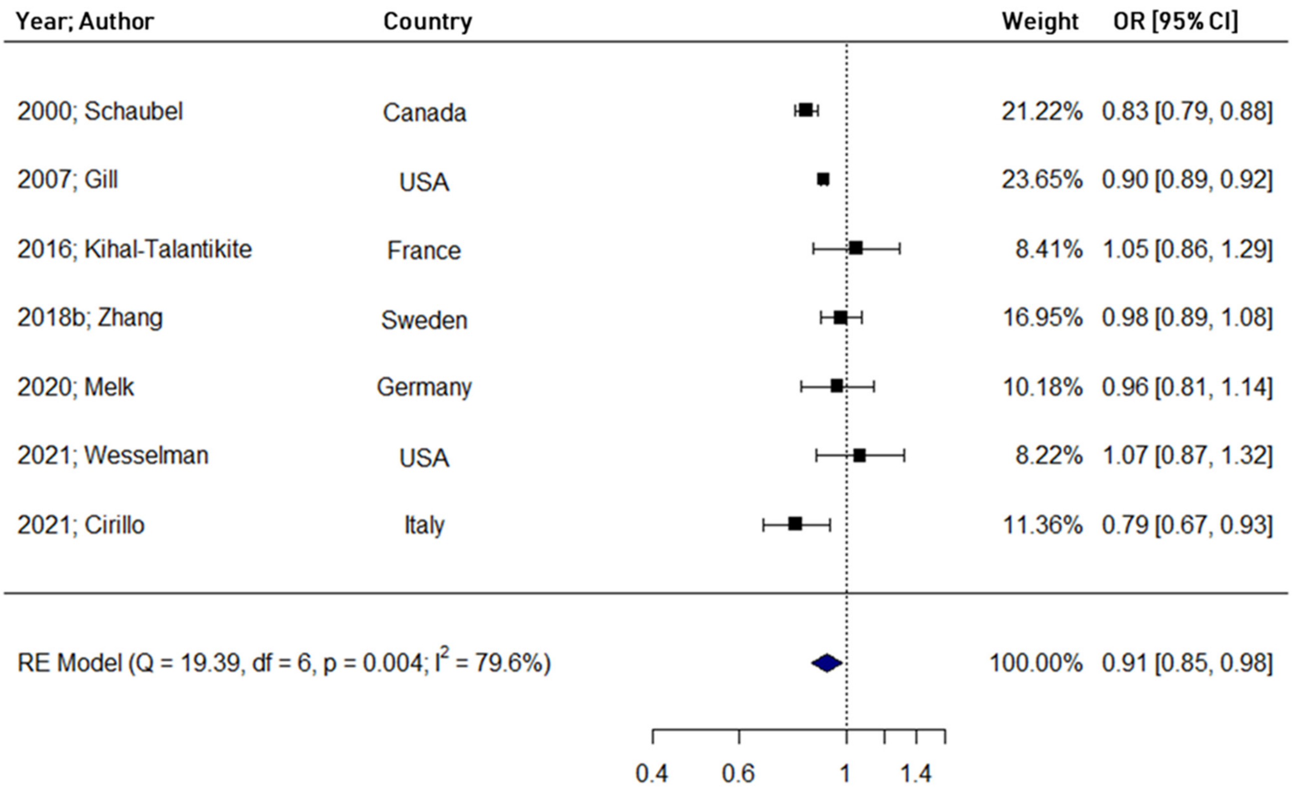

Suppl. Figure S46. Forest plot comparing female and male patients (reference) regarding kidney transplantation.

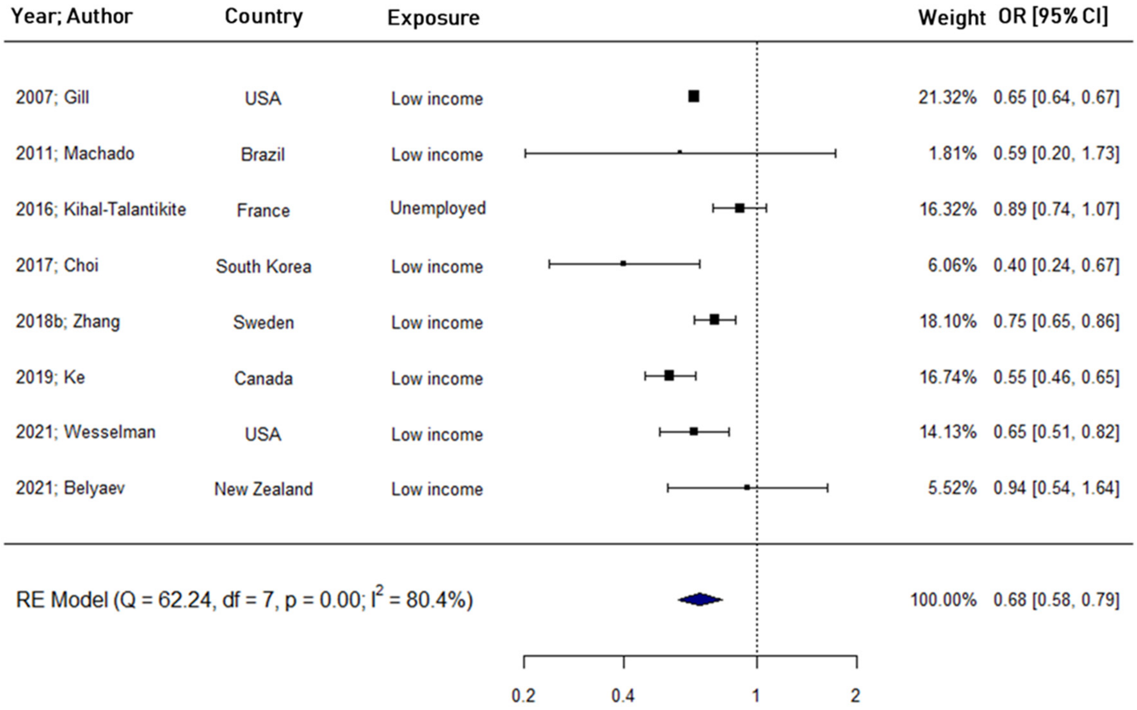

Suppl. Figure S47. Forest plot comparing low and high socioeconomic status patients (reference) regarding kidney transplantation.

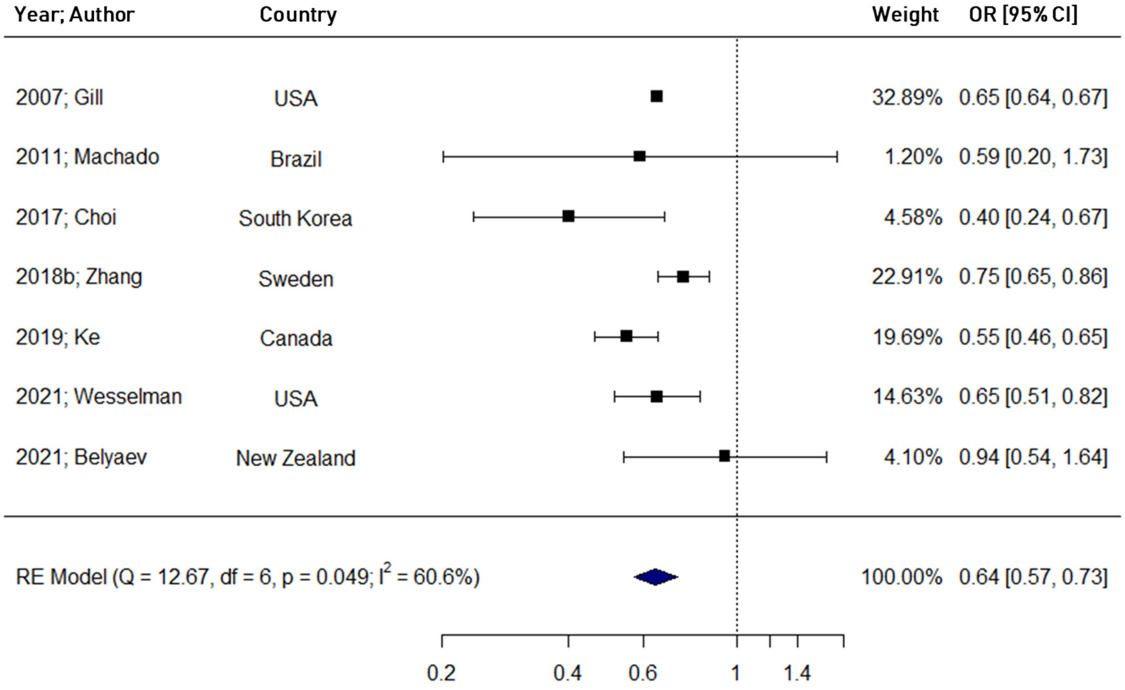

Suppl. Figure S48. Forest plot comparing low- and high- income patients (reference) regarding kidney transplantation.

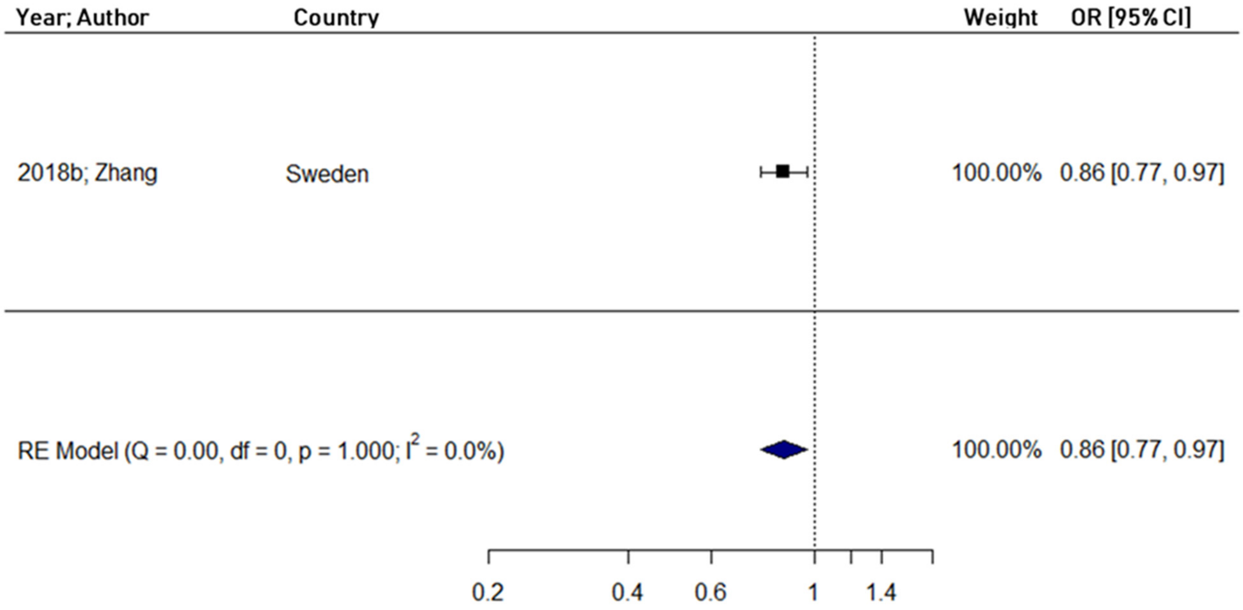

Suppl. Figure S49. Forest plot comparing low and high education patients (reference) regarding kidney transplantation.

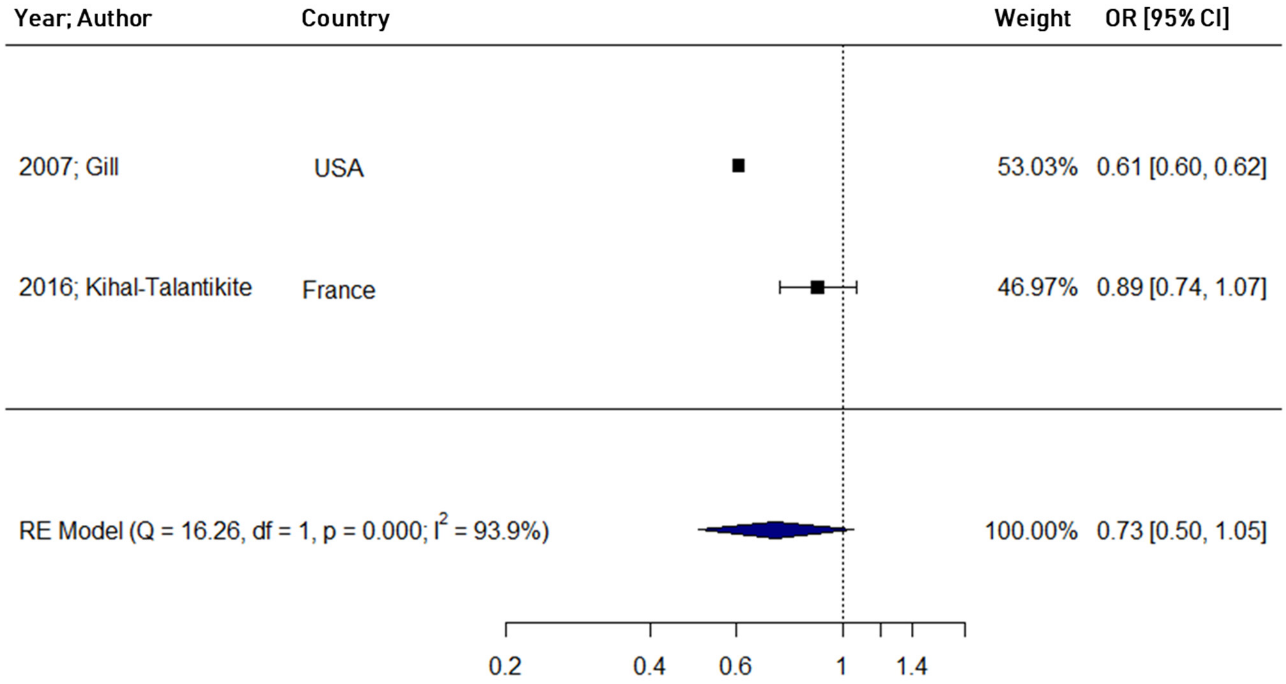

Suppl. Figure S50. Forest plot comparing unemployed and employed patients (reference) regarding kidney transplantation.

5.11. Living-donor transplantation

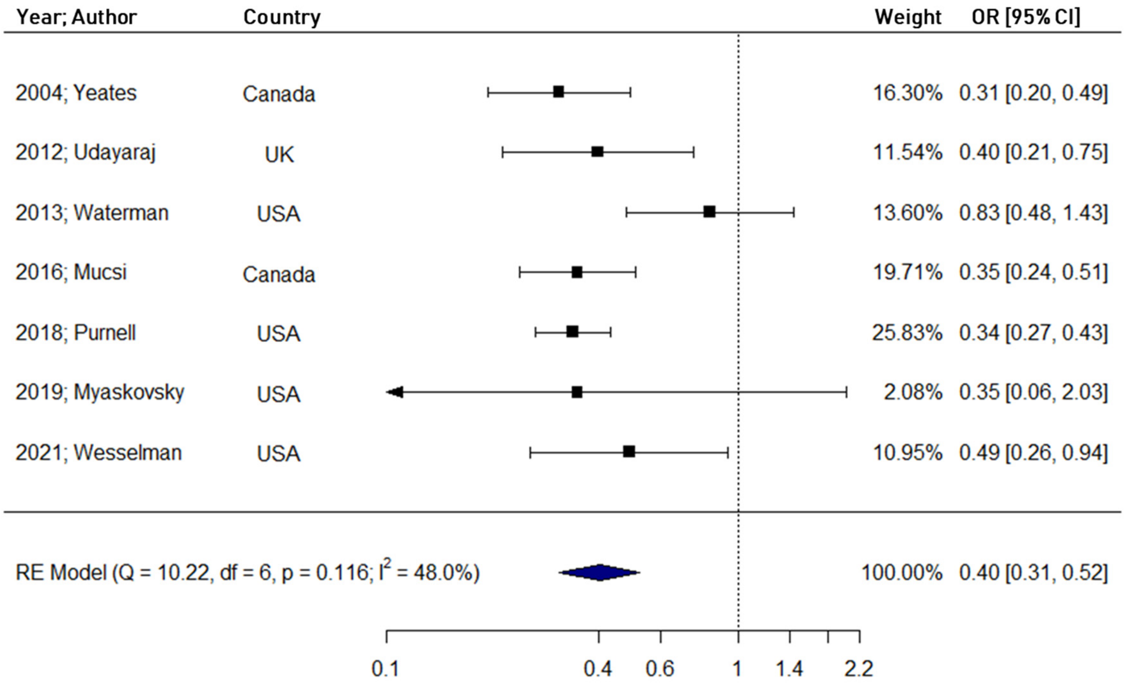

Suppl. Figure S51. Forest plot comparing Black and White patients (reference) regarding living-donor transplantation.

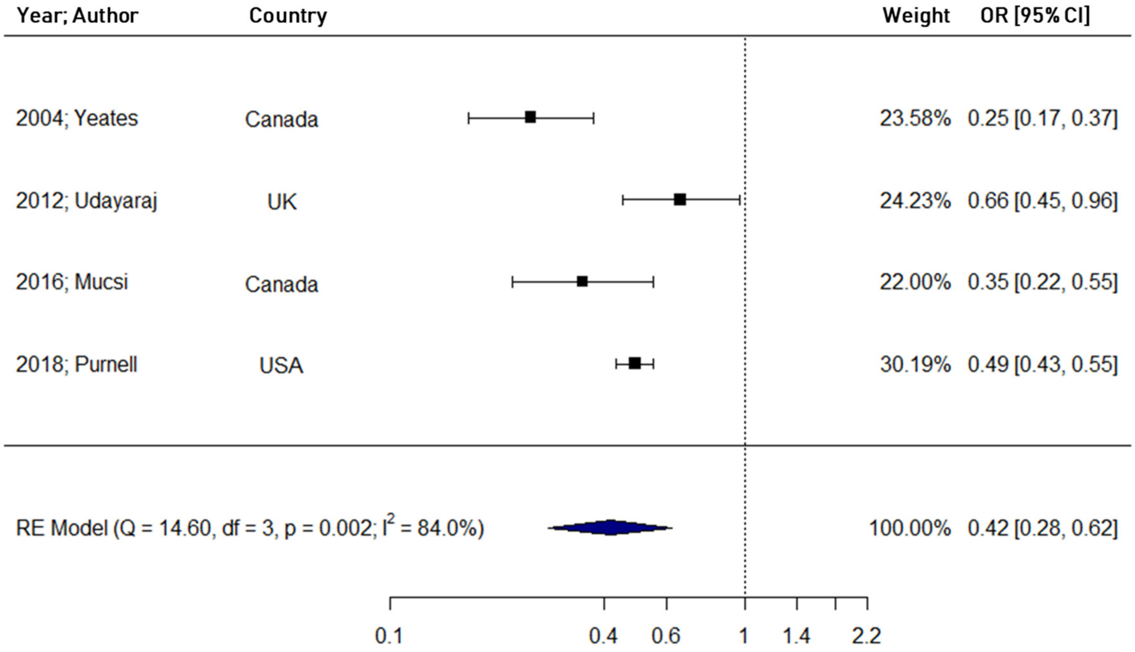

Suppl. Figure S52. Forest plot comparing Asian and White patients (reference) regarding living-donor transplantation.

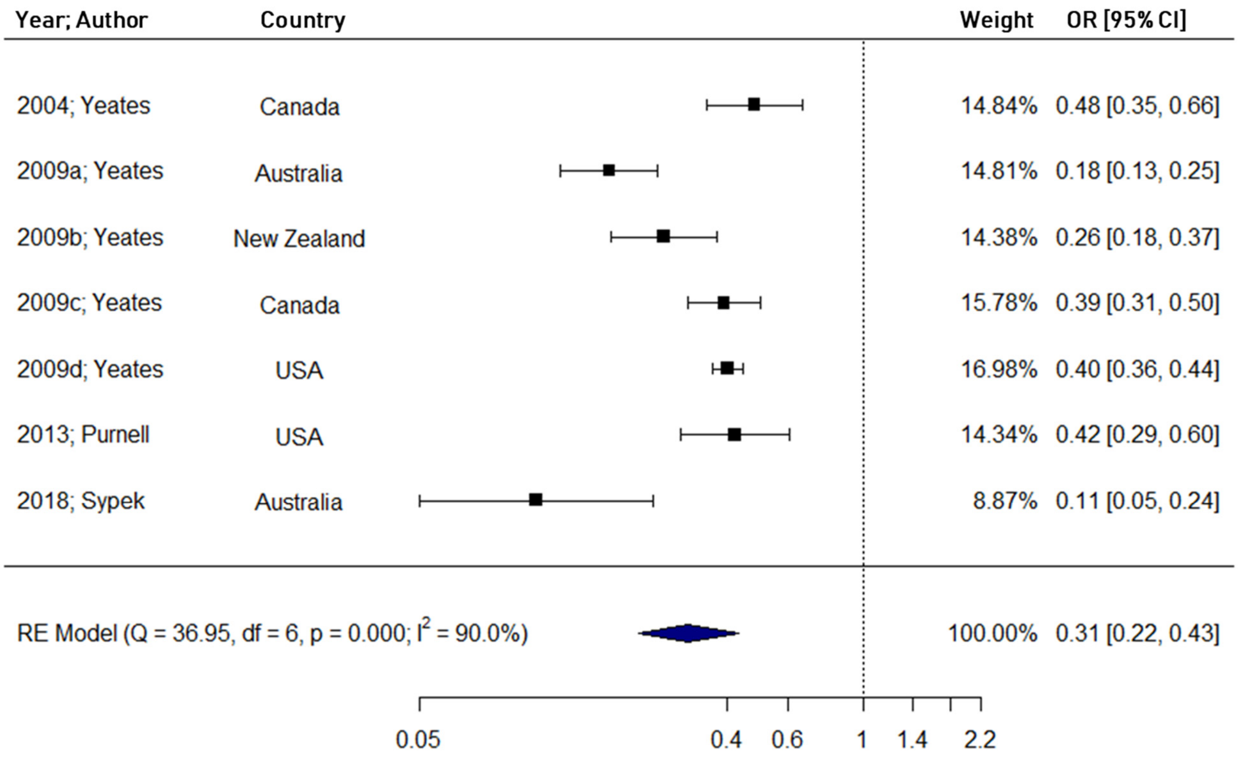

Suppl. Figure S53. Forest plot comparing Indigenous and White patients (reference) regarding living-donor transplantation.

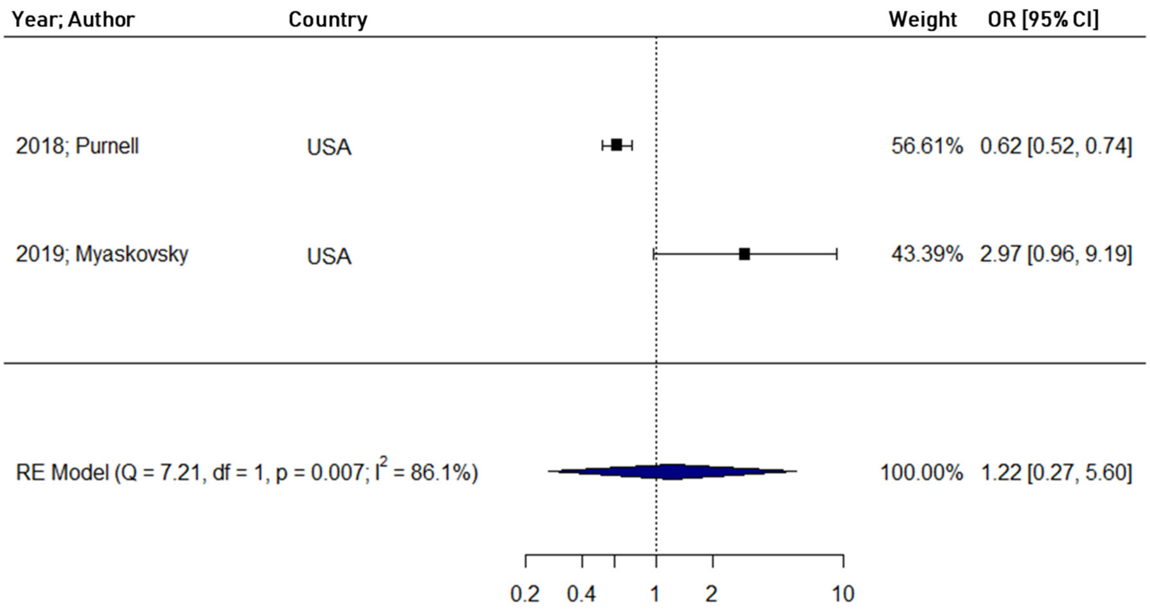

Suppl. Figure S54. Forest plot comparing Hispanic and White patients (reference) regarding living-donor transplantation.

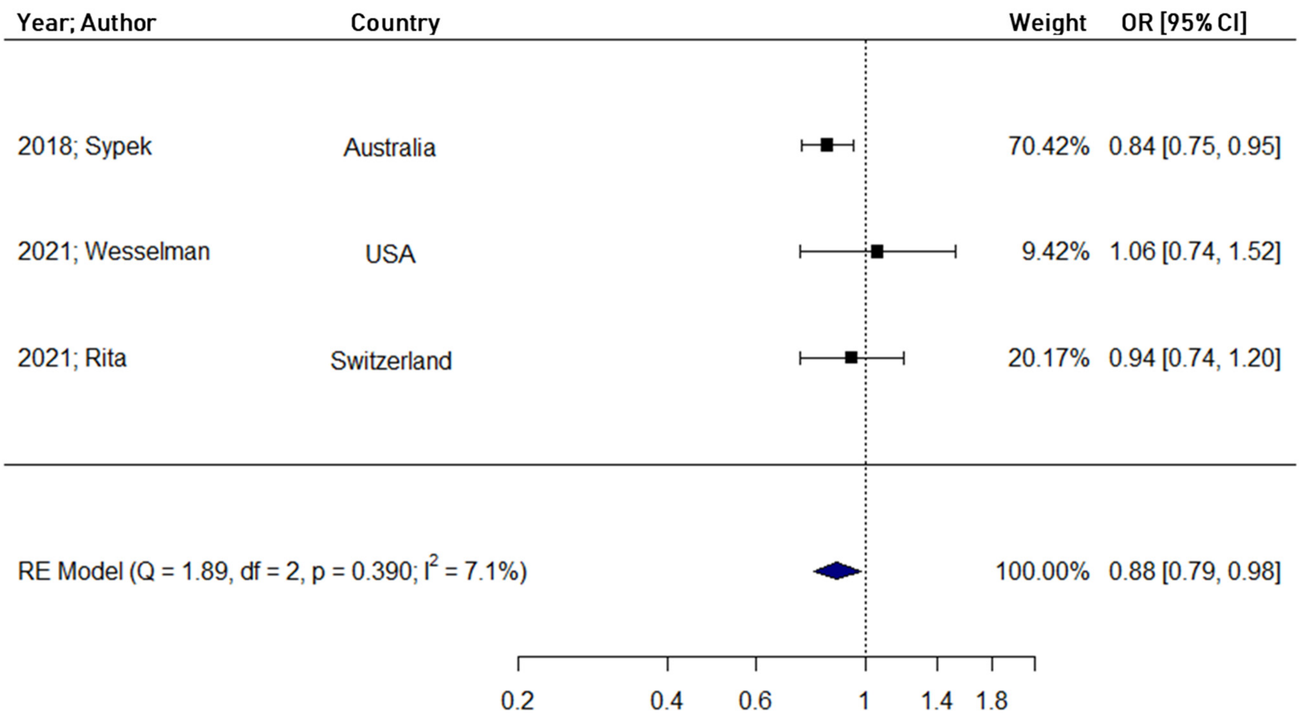

Suppl. Figure S55. Forest plot comparing female and male patients (reference) regarding living-donor transplantation.

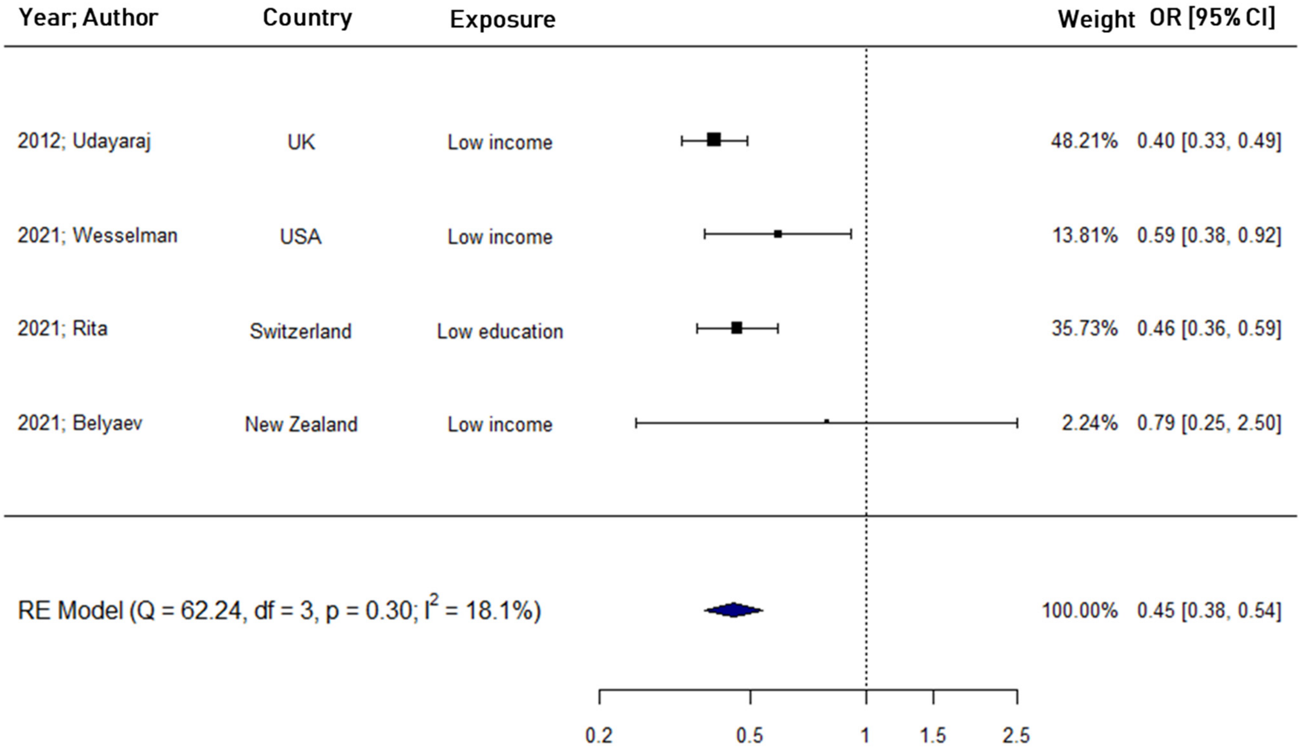

Suppl. Figure S56. Forest plot comparing low and high socioeconomic status patients (reference) regarding living-donor transplantation.

5.12. Deceased-donor transplantation

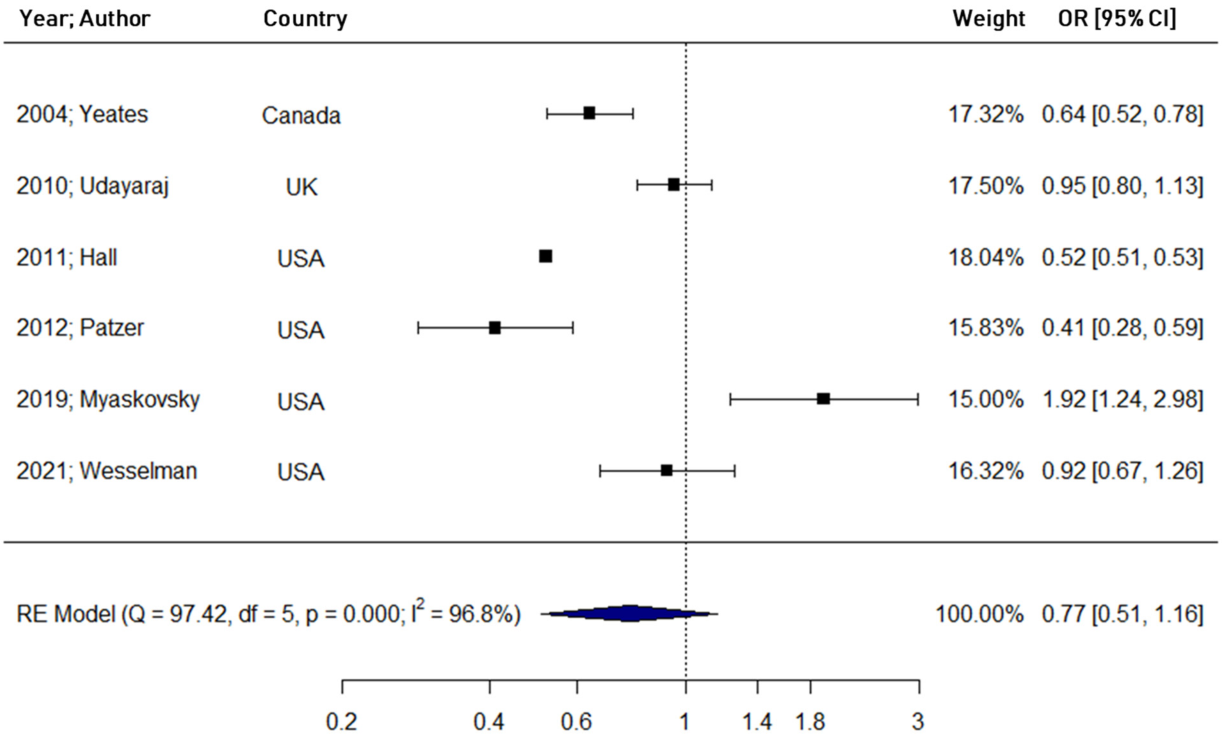

Suppl. Figure S57. Forest plot comparing Black and White patients (reference) regarding deceased-donor transplantation.

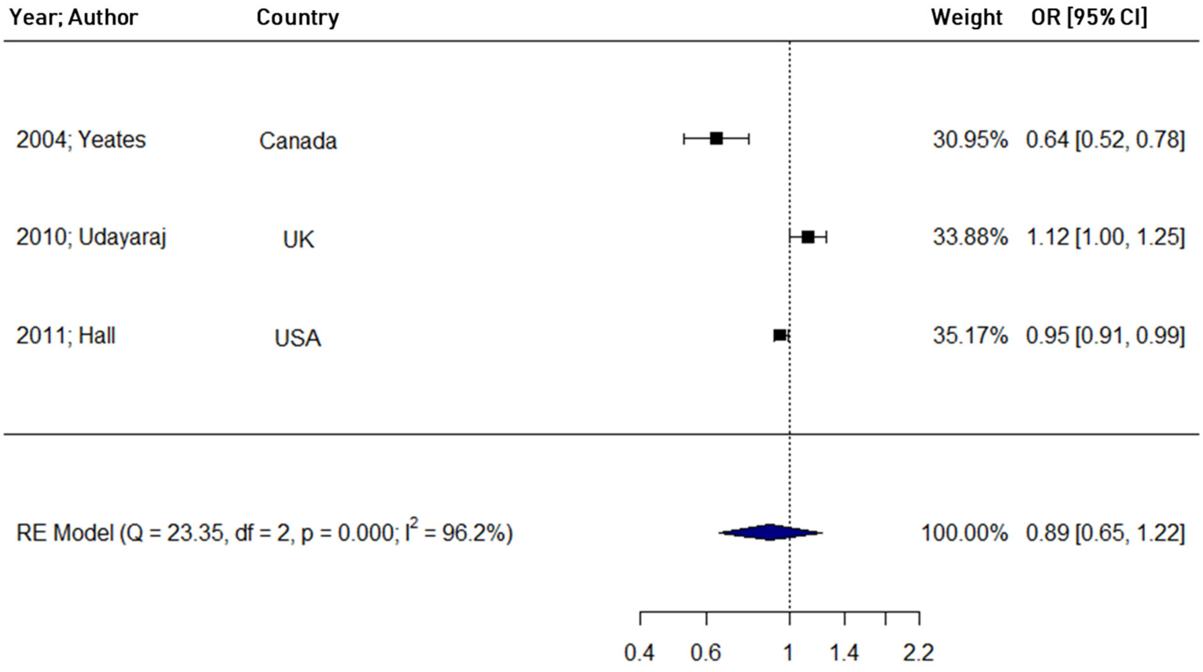

Suppl. Figure S58. Forest plot comparing Asian and White patients (reference) regarding deceased-donor transplantation.

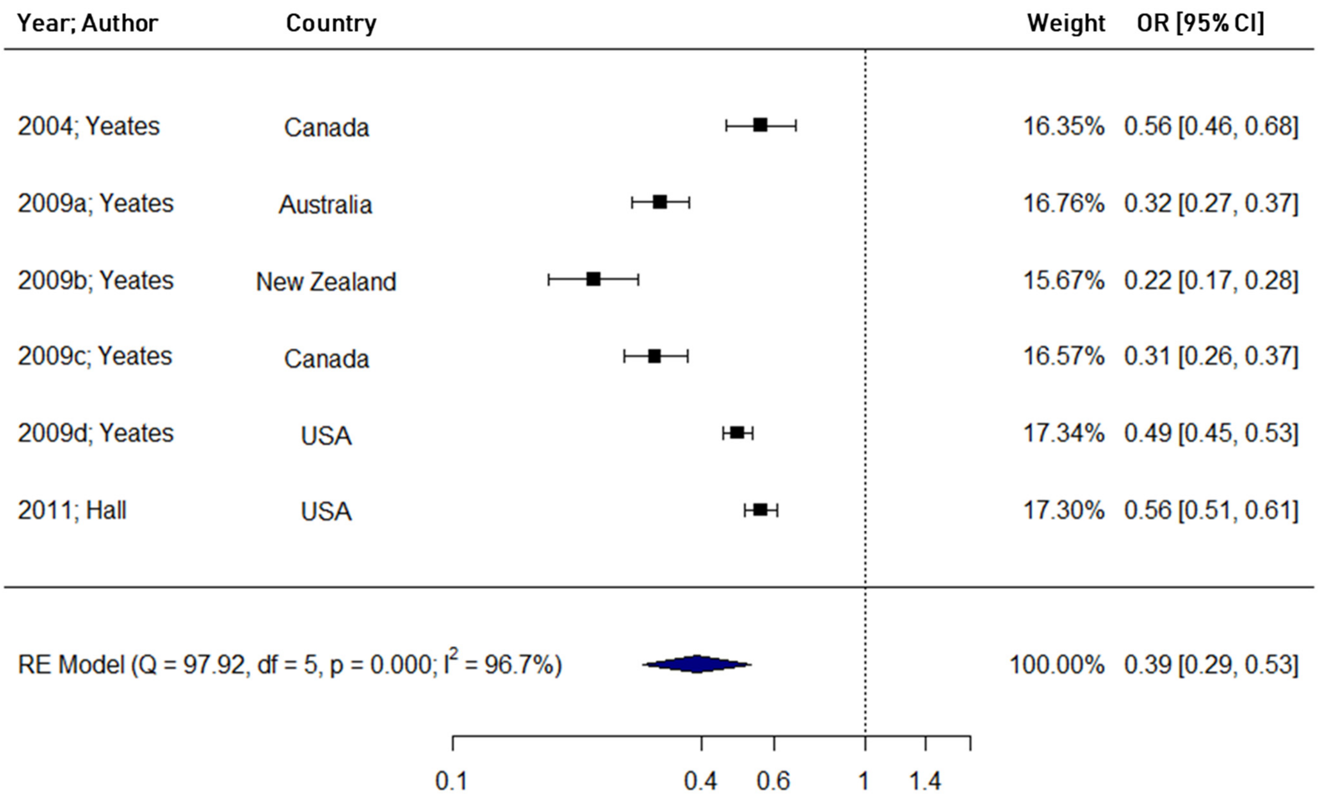

Suppl. Figure S59. Forest plot comparing Indigenous and White patients (reference) regarding deceased-donor transplantation.

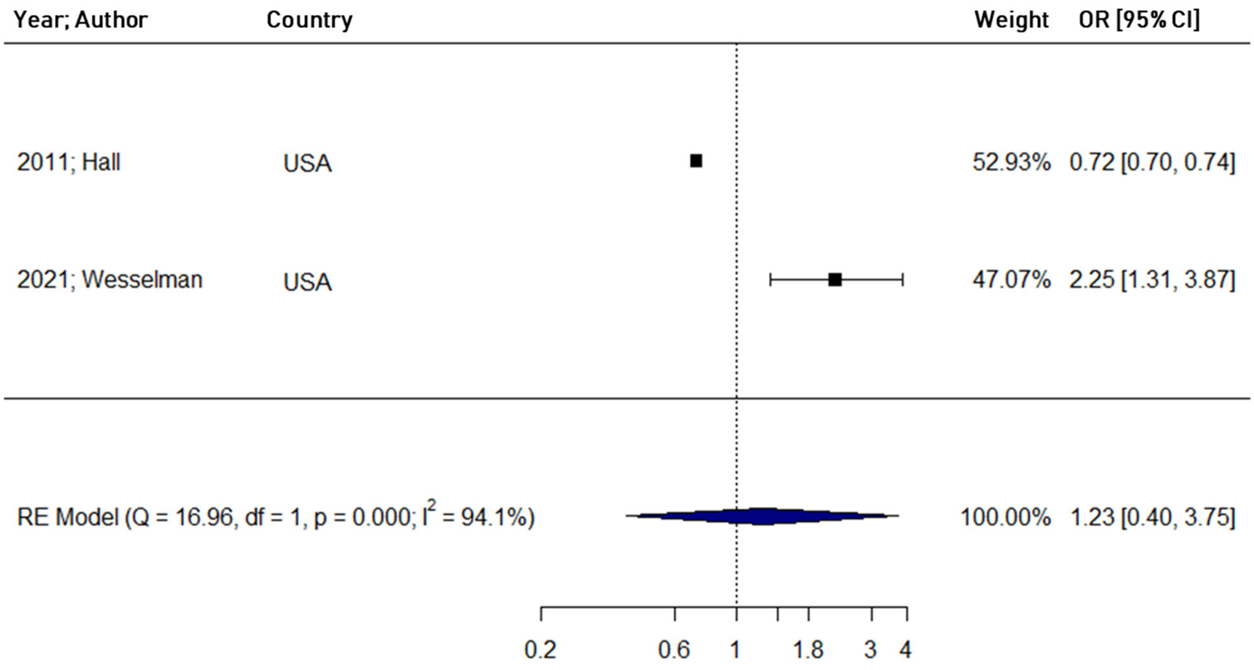

Suppl. Figure S60. Forest plot comparing Hispanic and White patients (reference) regarding deceased-donor transplantation.

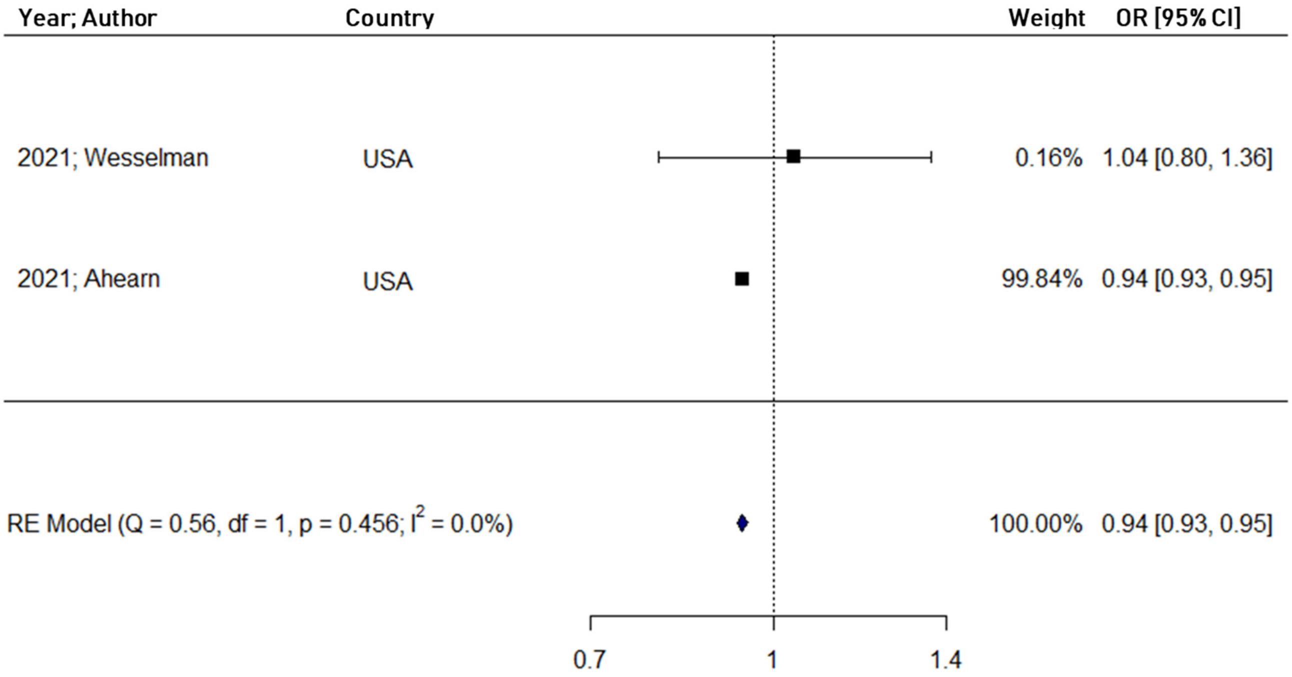

Suppl. Figure S61. Forest plot comparing female and male patients (reference) regarding deceased-donor transplantation.

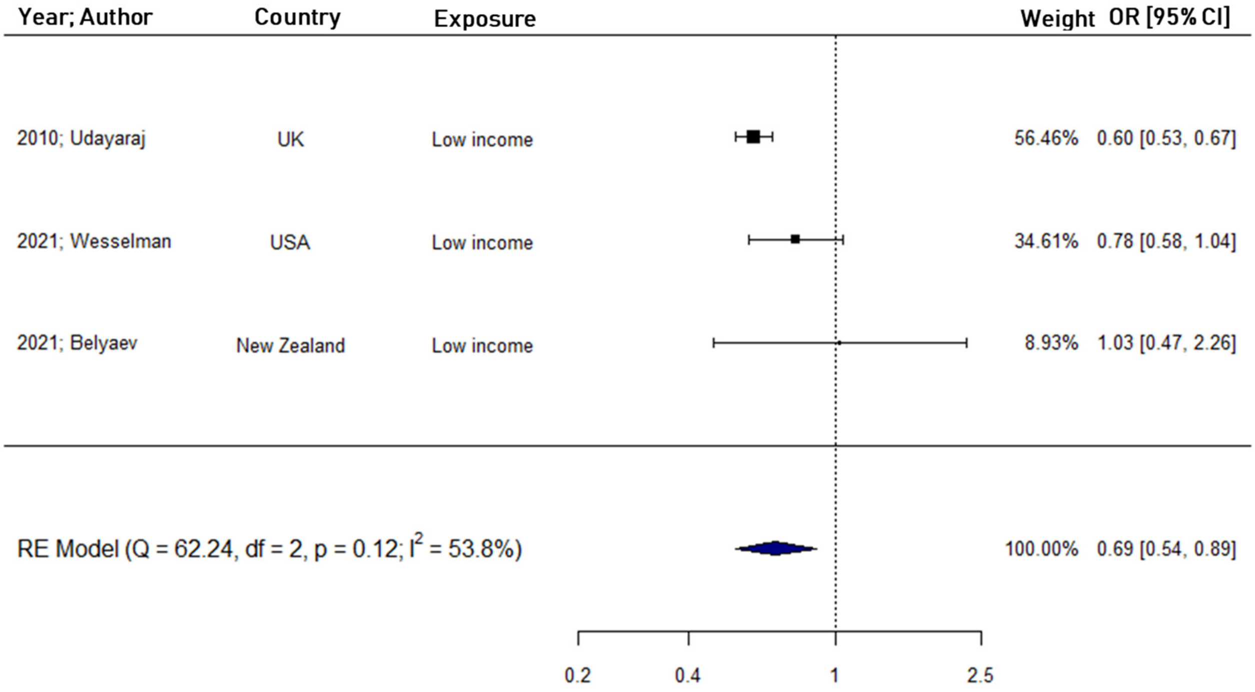

Suppl. Figure S62. Forest plot comparing low and high socioeconomic status patients (reference) regarding deceased-donor transplantation.

5.13. Preemptive transplantation

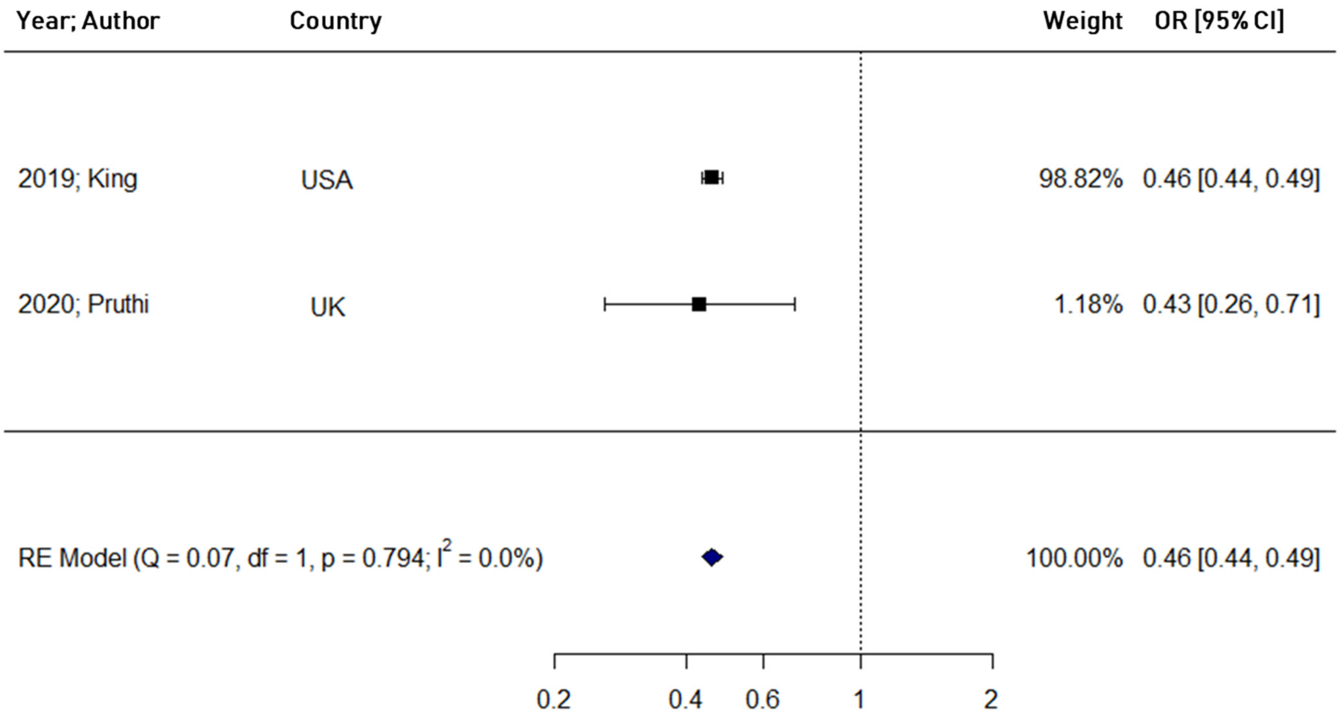

Suppl. Figure S63. Forest plot comparing Black and White patients (reference) regarding preemptive transplantation.

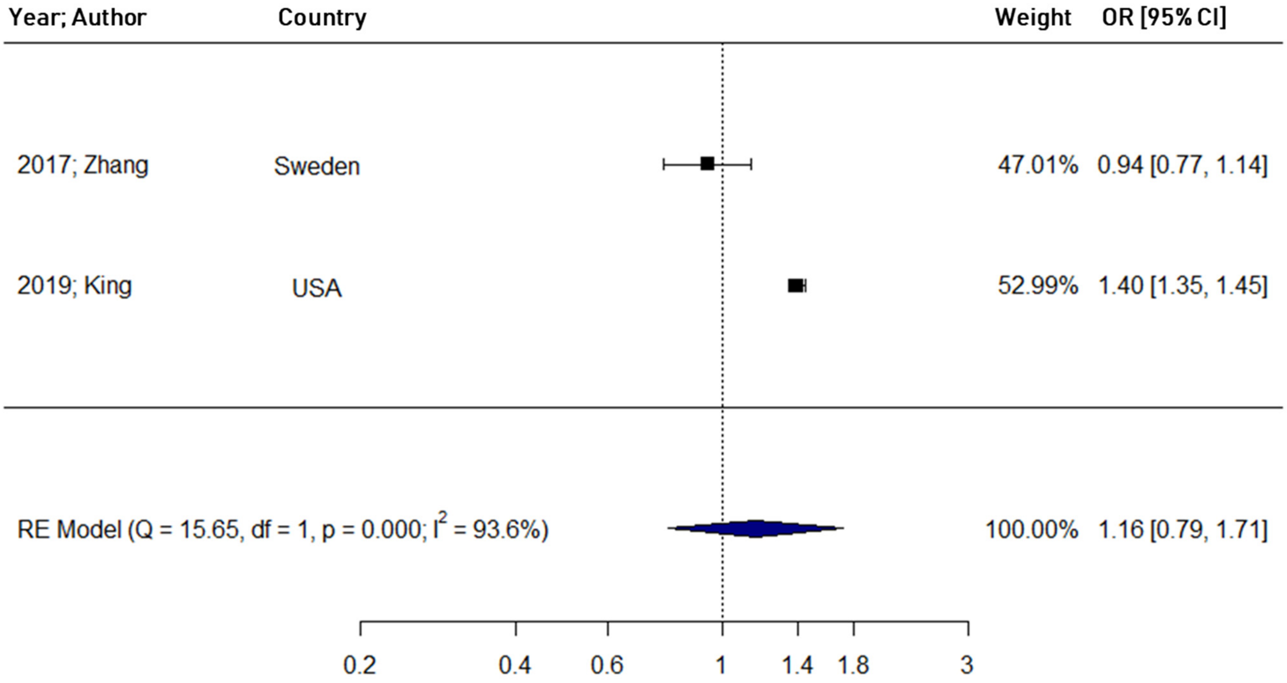

Suppl. Figure S64. Forest plot comparing female and male patients (reference) regarding preemptive transplantation.

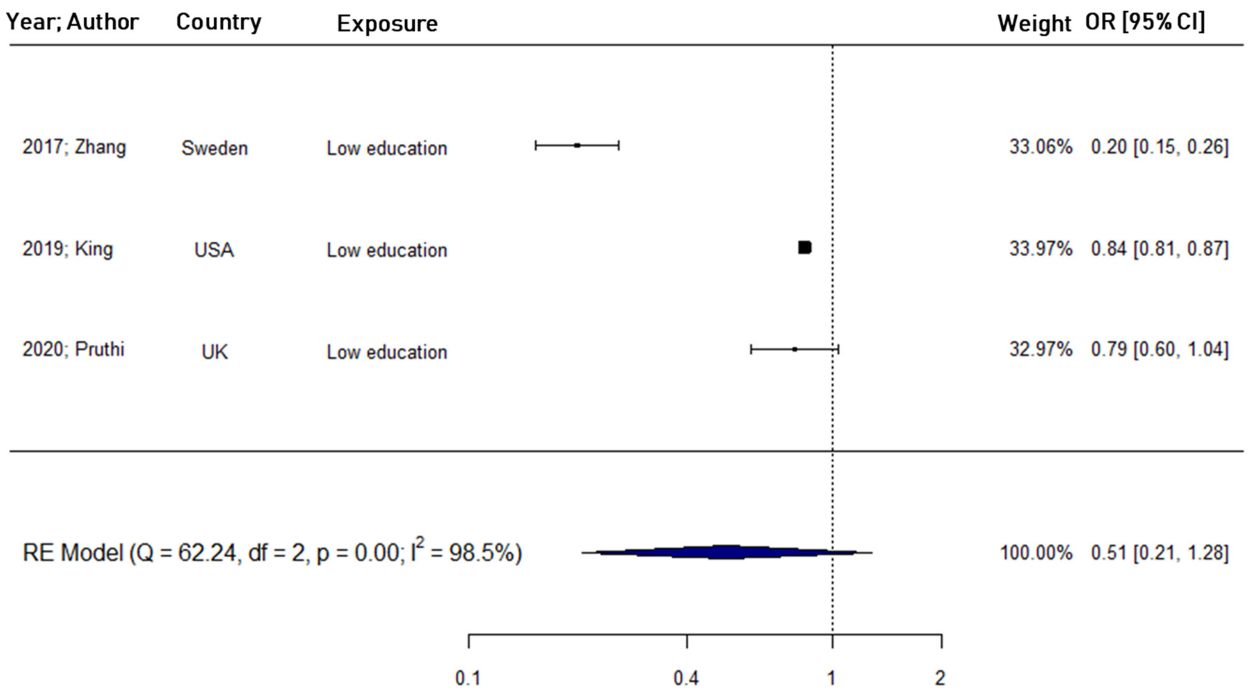

Suppl. Figure S65. Forest plot comparing low and high socioeconomic status patients (reference) regarding preemptive transplantation.

5.14. Graft survival

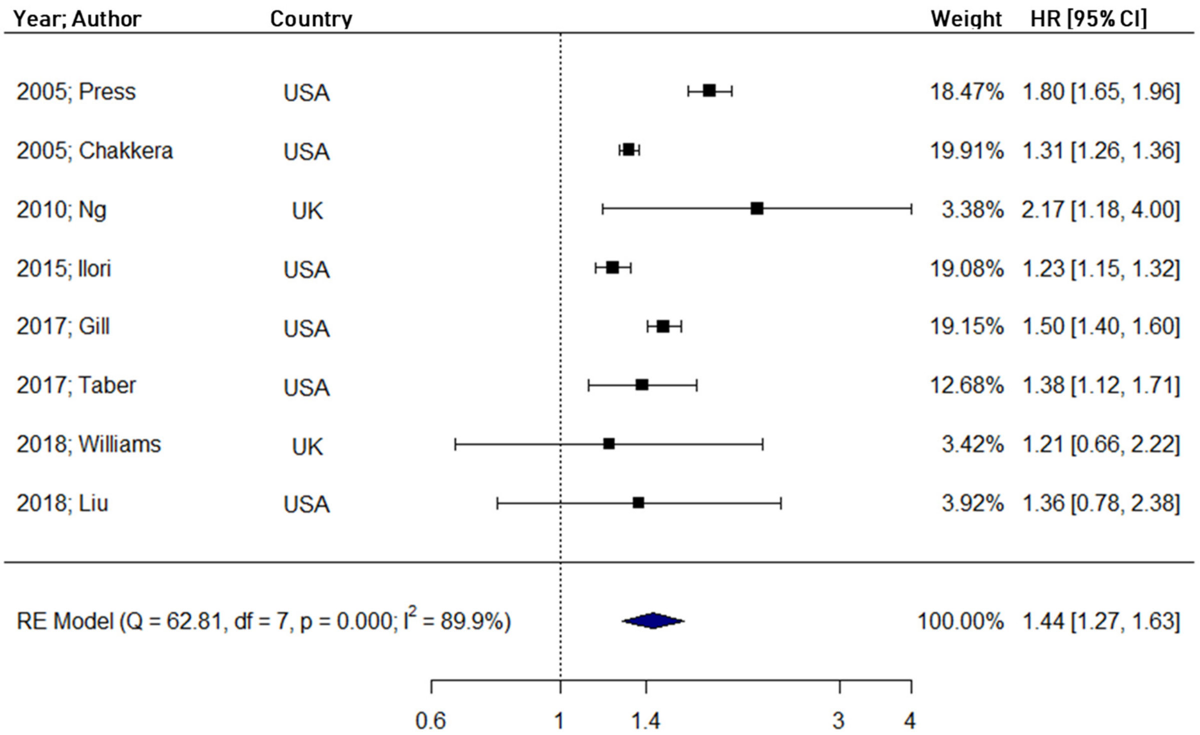

Suppl. Figure S66. Forest plot comparing Black and White patients (reference) regarding graft survival.

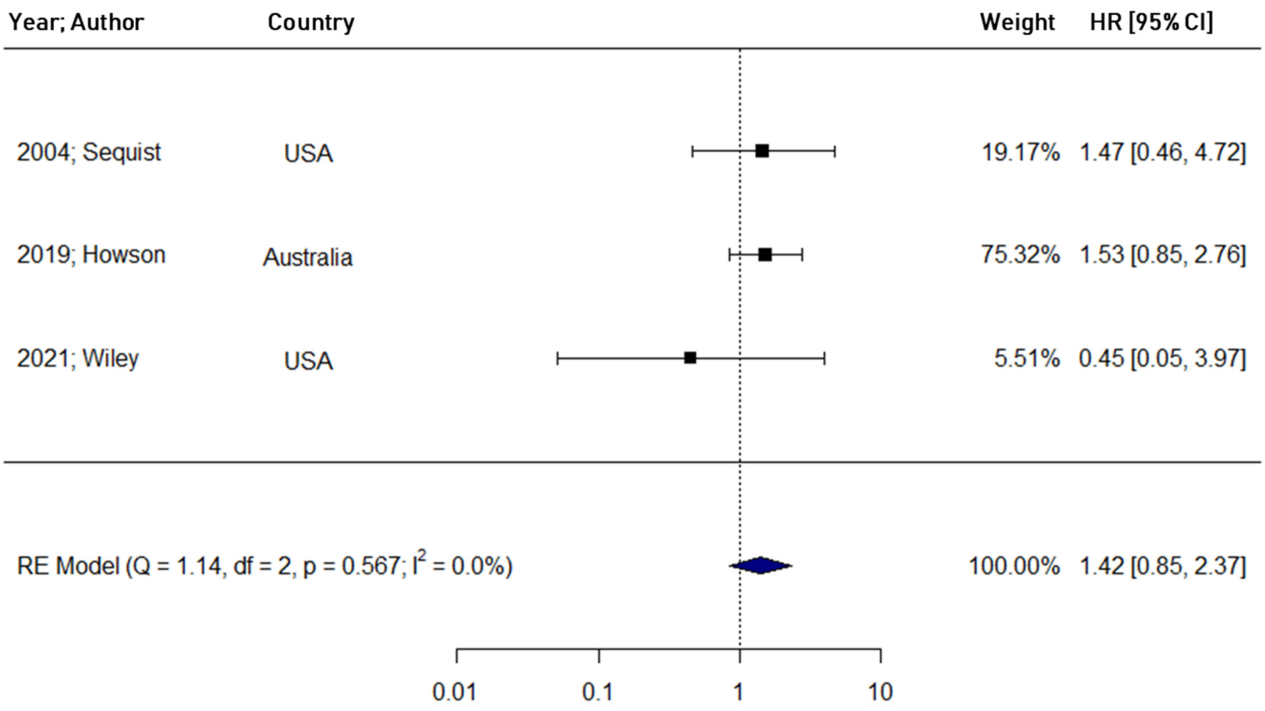

Suppl. Figure S67. Forest plot comparing Indigenous and White patients (reference) regarding graft survival.

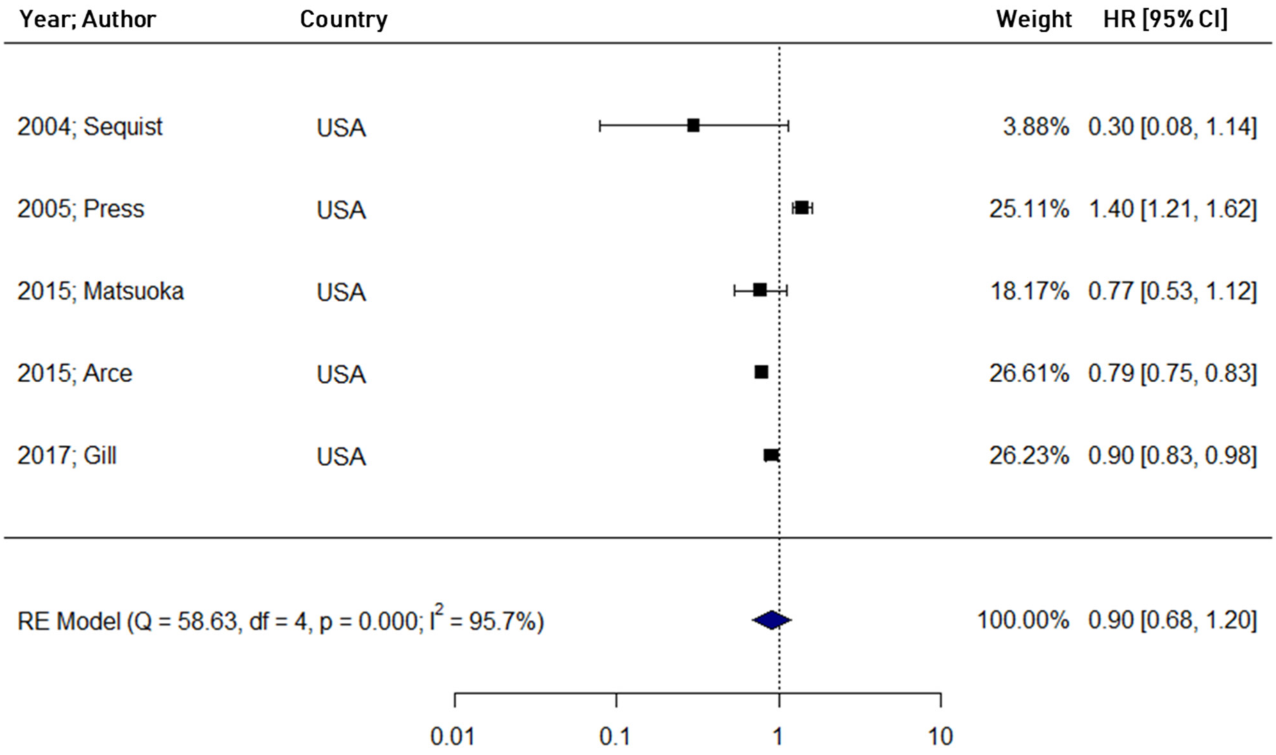

Suppl. Figure S68. Forest plot comparing Hispanic and White patients (reference) regarding graft survival.

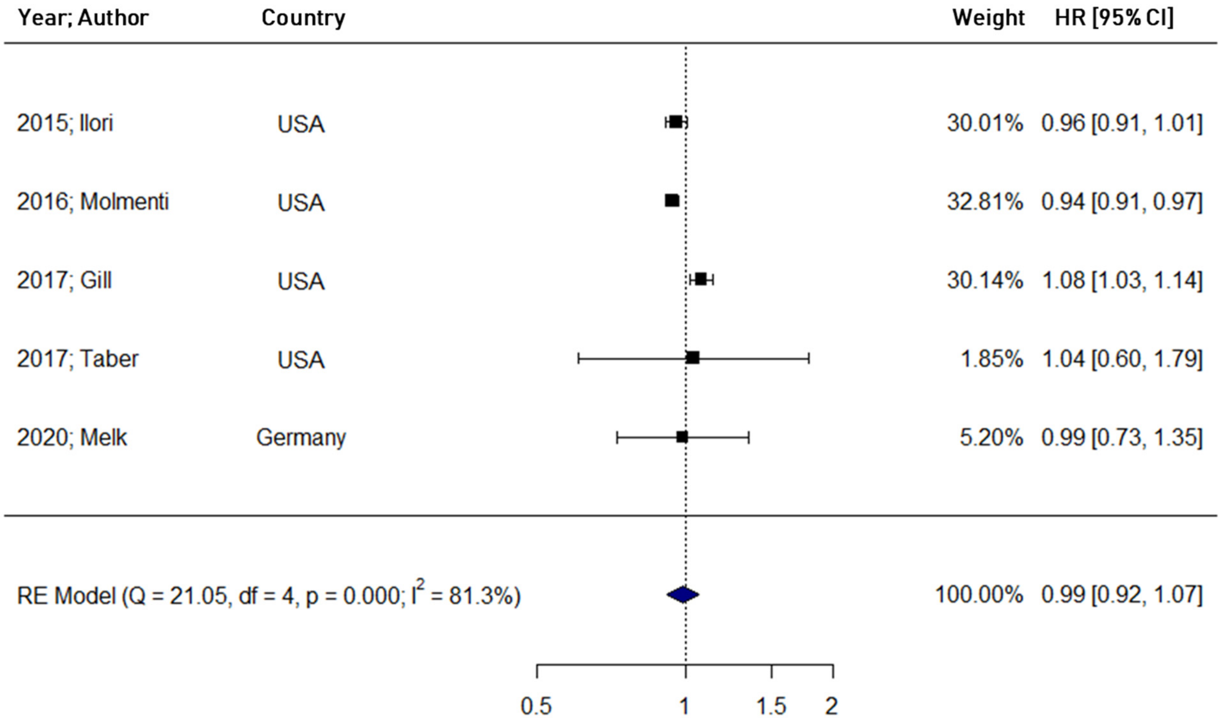

Suppl. Figure S69. Forest plot comparing female and male patients (reference) regarding n graft survival.

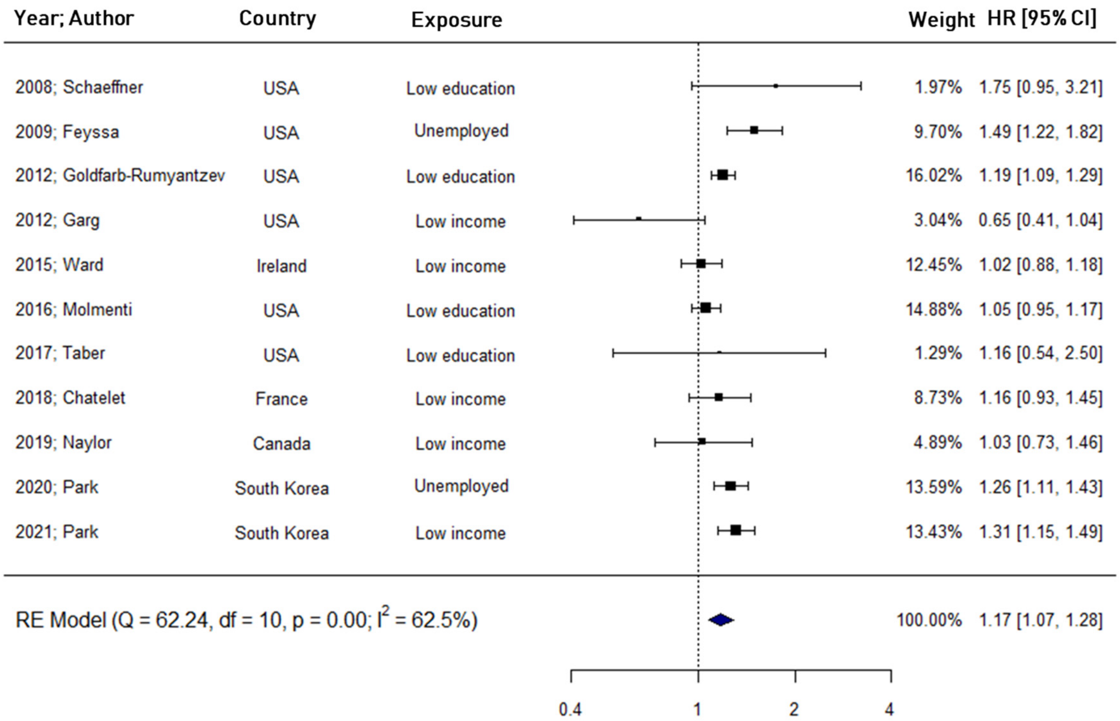

Suppl. Figure S70. Forest plot comparing low and high socioeconomic status patients (reference) regarding graft survival.

5.15. Kidney transplantation survival

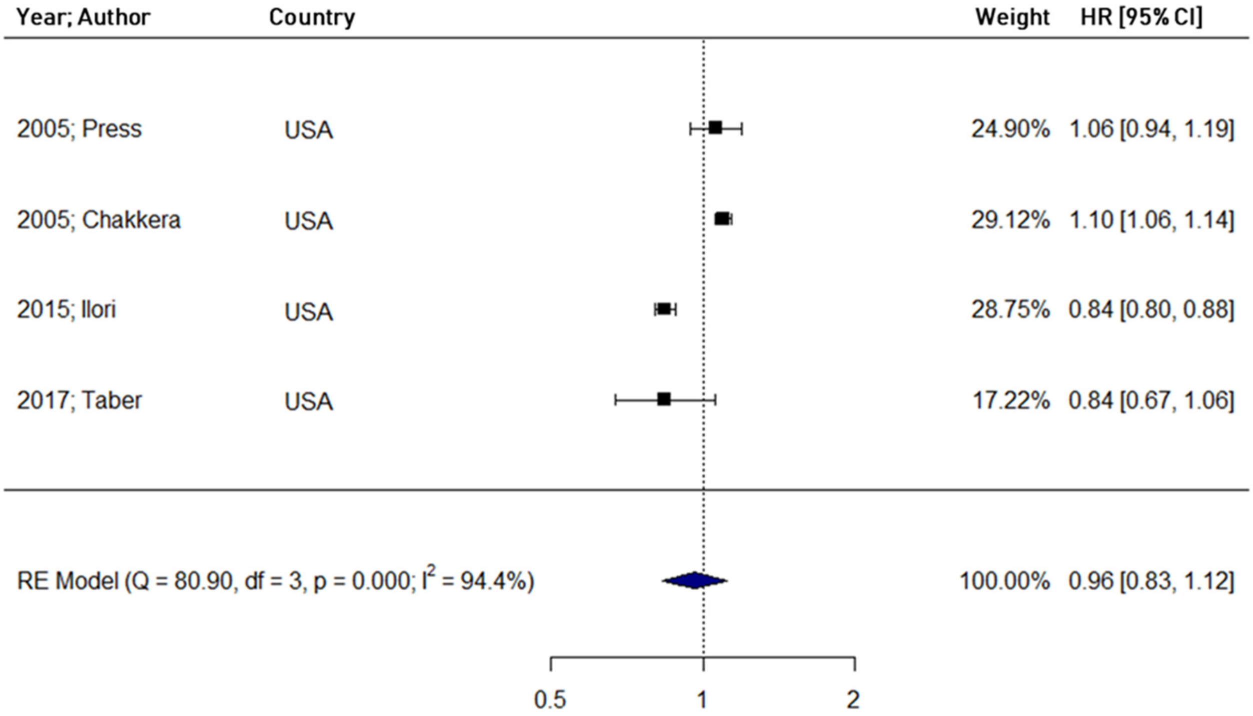

Suppl. Figure S71. Forest plot comparing Black and White patients (reference) regarding kidney transplantation survival.

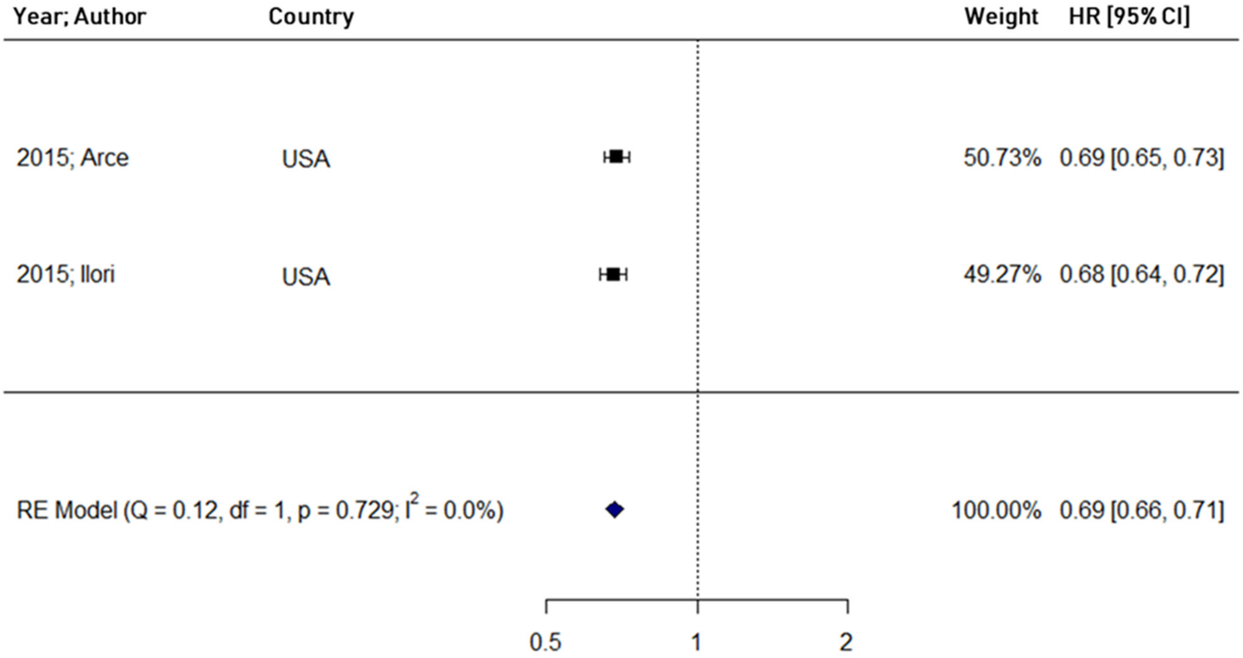

Suppl. Figure S72. Forest plot comparing Hispanic and White patients (reference) regarding kidney transplantation survival.

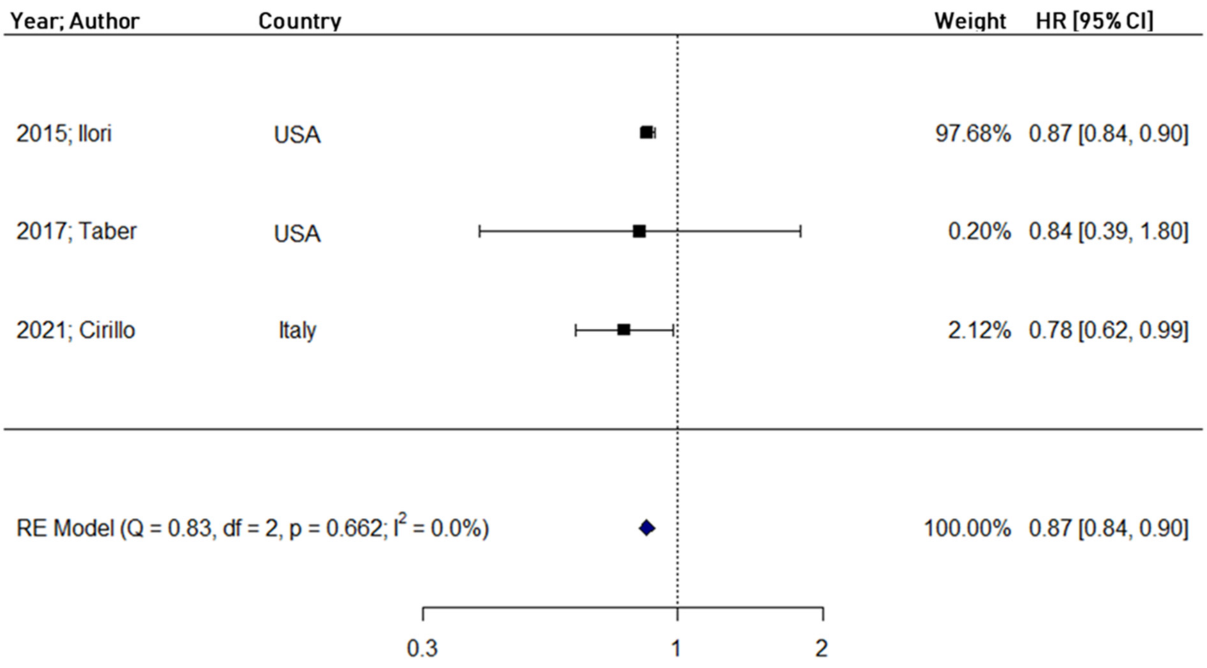

Suppl. Figure S73. Forest plot comparing female and male patients (reference) regarding kidney transplantation survival.

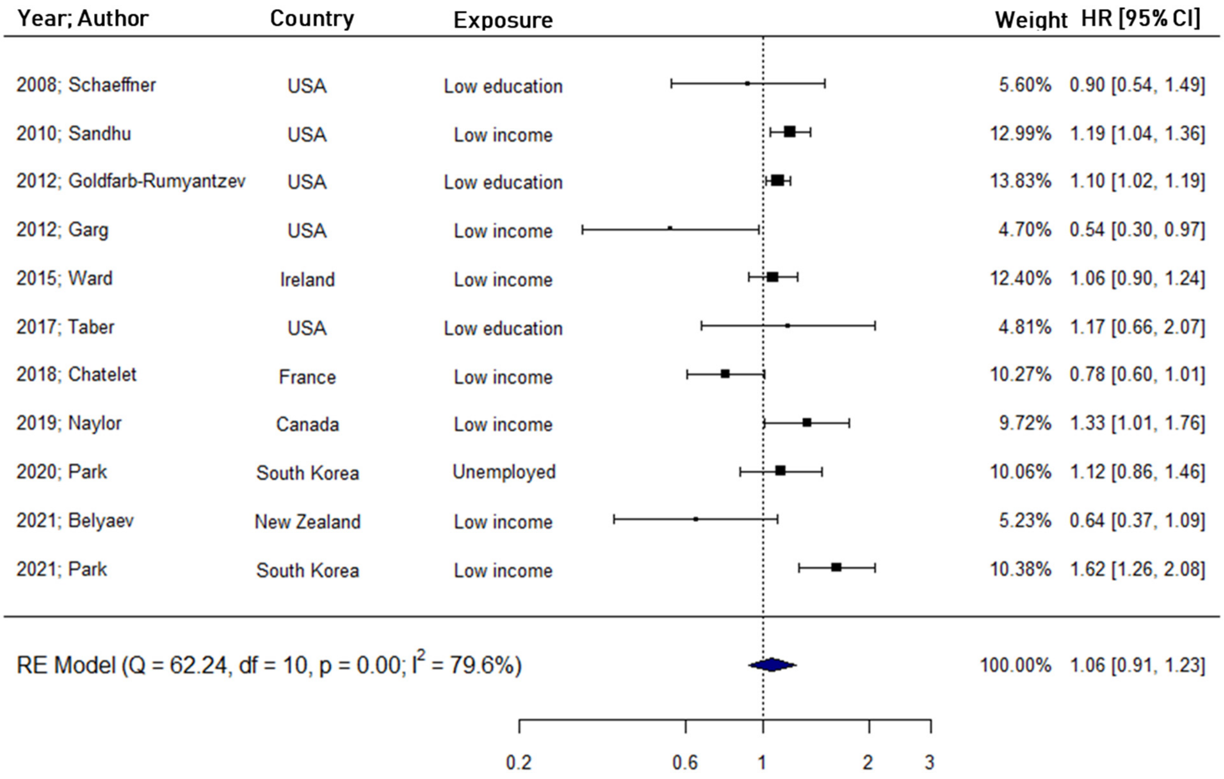

Suppl. Figure S74. Forest plot comparing low and high socioeconomic status patients (reference) regarding kidney transplantation survival.

Supplementary S6: Funnel plots

6.1. Arteriovenous fistula/graft *vs*. Central venous catheter

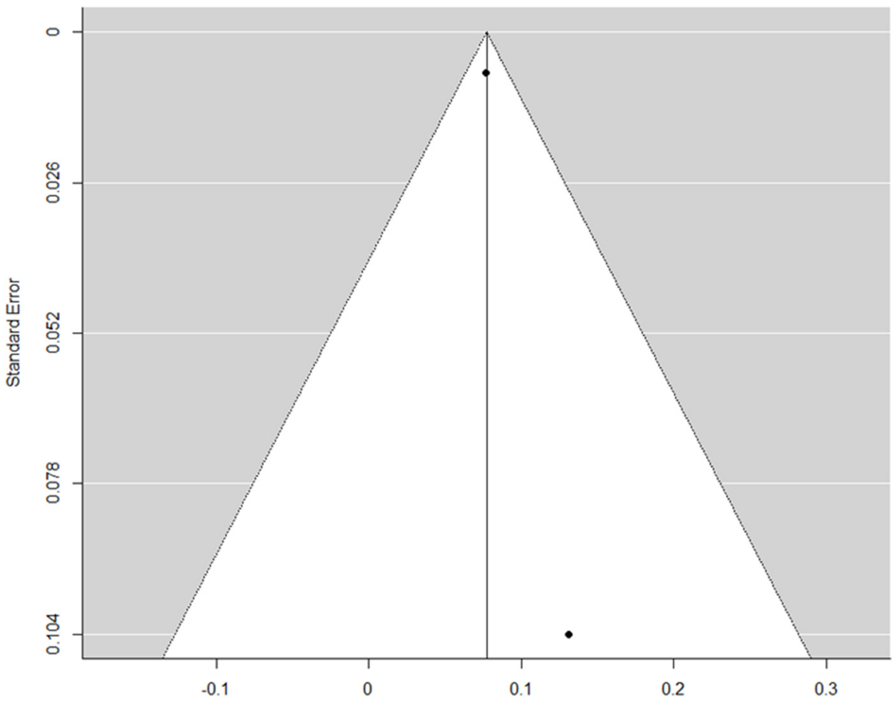

Suppl. Figure S75. Funnel plot comparing Black and White patients (reference) regarding dialysis initiation with arteriovenous fistula or graft.

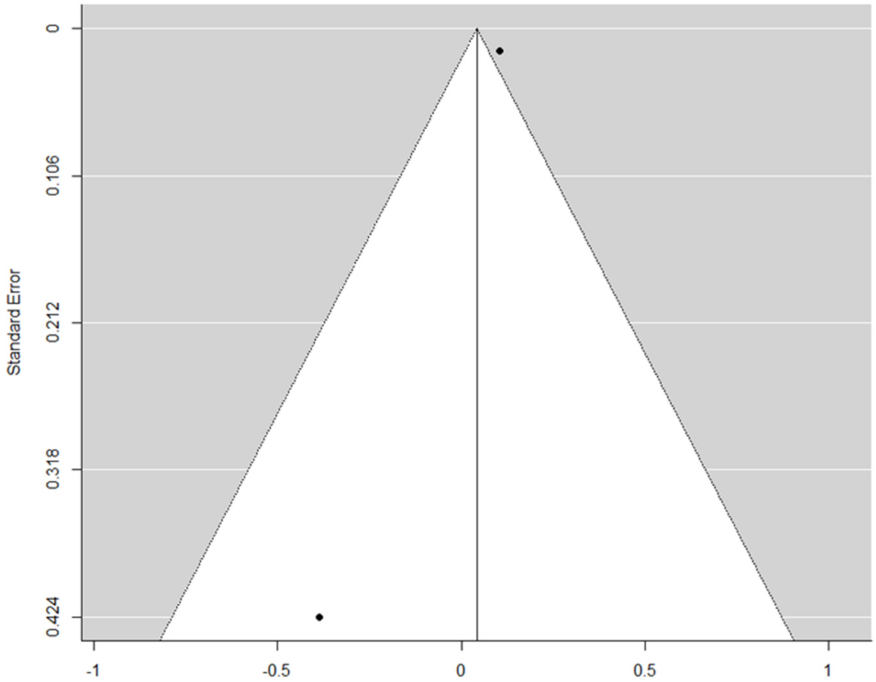

Suppl. Figure S76. Funnel plot comparing Asian and White patients (reference) regarding dialysis initiation with arteriovenous fistula or graft.

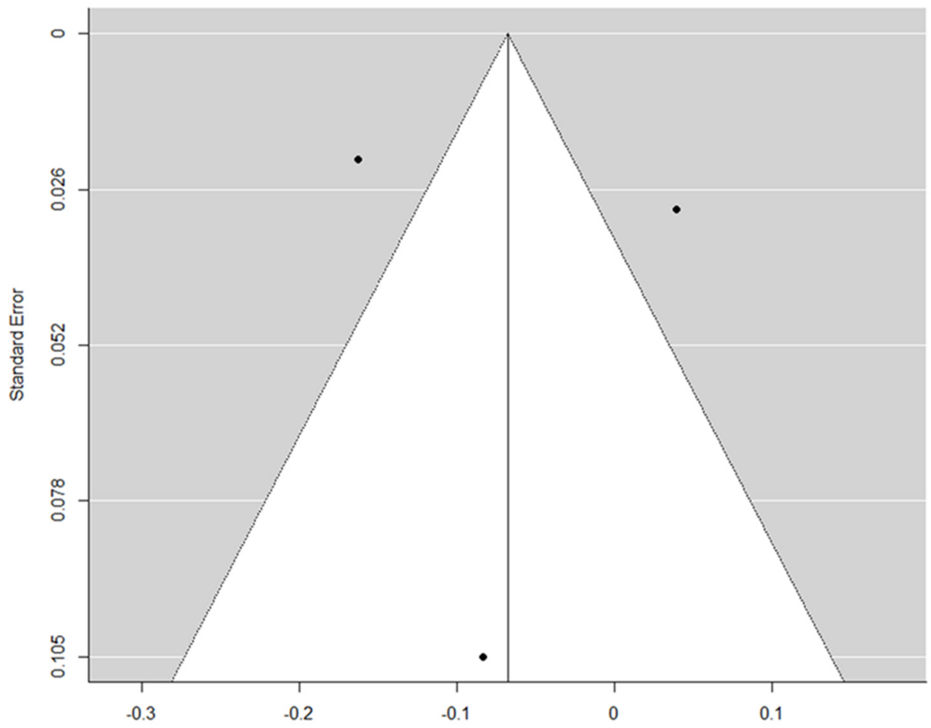

Suppl. Figure S77. Funnel plot comparing Indigenous and White patients (reference) regarding dialysis initiation with arteriovenous fistula or graft.

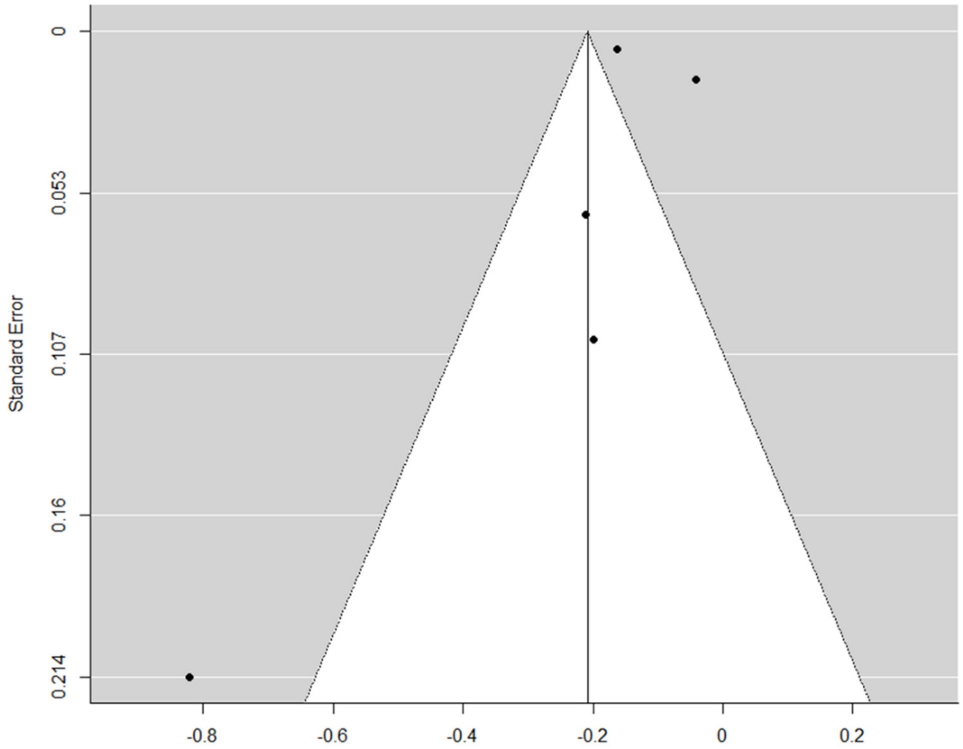

Suppl. Figure S78. Funnel plot of female patients (reference) and dialysis initiation with arteriovenous fistula or graft.

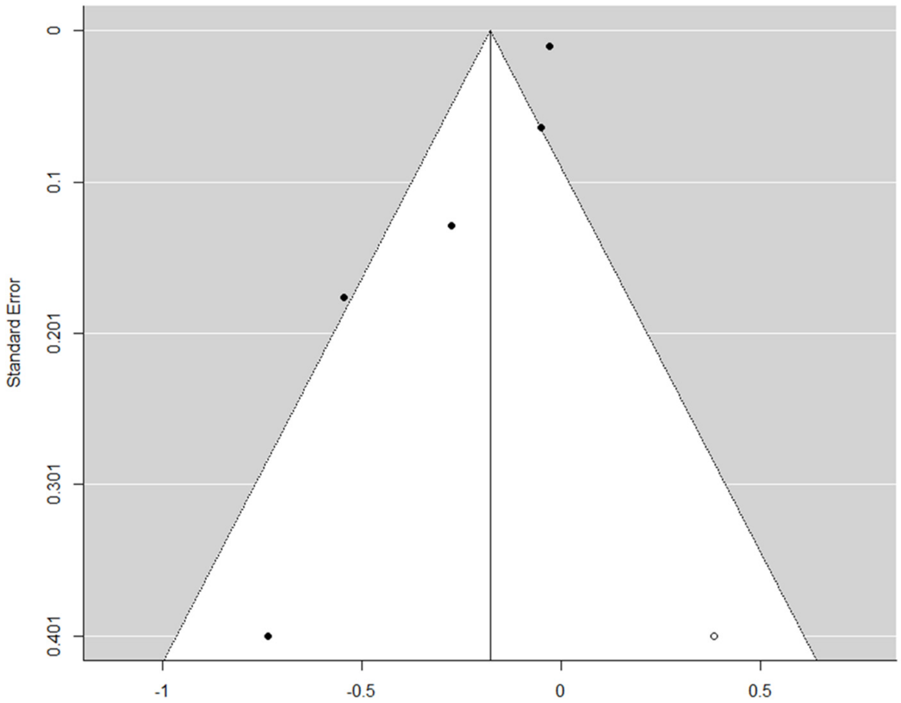

Suppl. Figure S79. Funnel plot comparing low and high socioeconomic status patients (reference) regarding dialysis initiation with arteriovenous fistula or graft.

6.2. Arteriovenous fistula *vs*. Arteriovenous graft

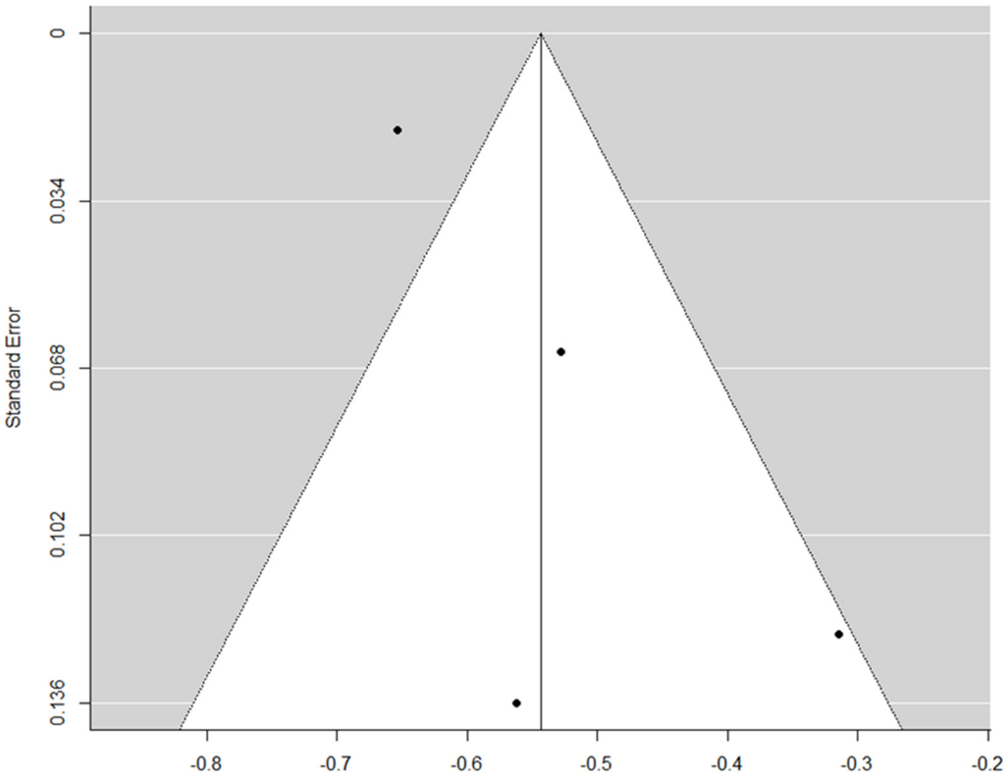

Suppl. Figure S80. Funnel plot comparing Black and White patients (reference) regarding dialysis initiation with arteriovenous fistula *vs*. arteriovenous graft.

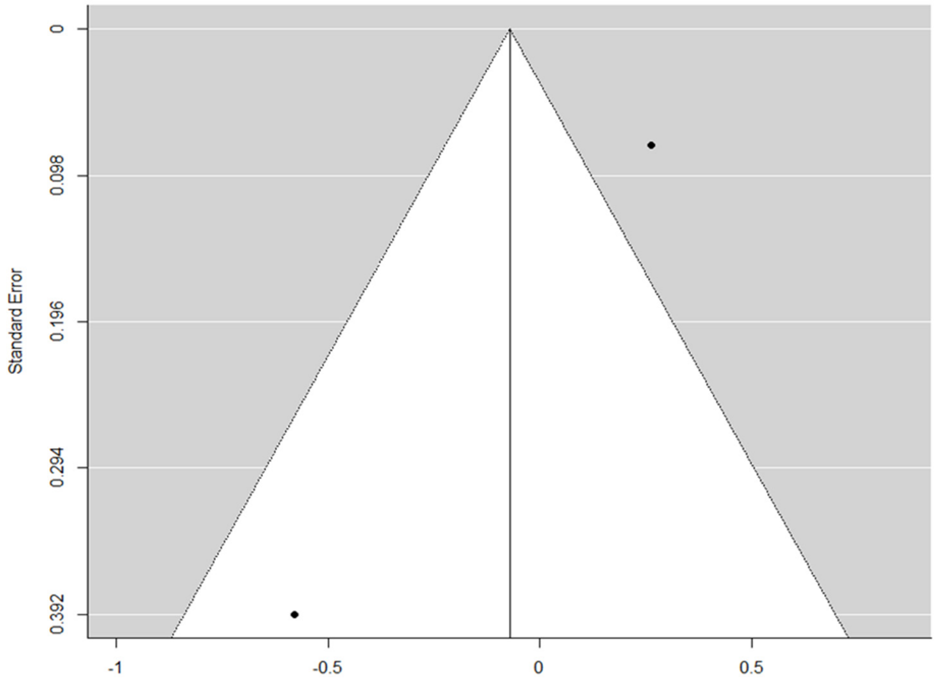

Suppl. Figure S81. Funnel plot comparing Indigenous and White patients (reference) regarding dialysis initiation with arteriovenous fistula *vs*. arteriovenous graft.

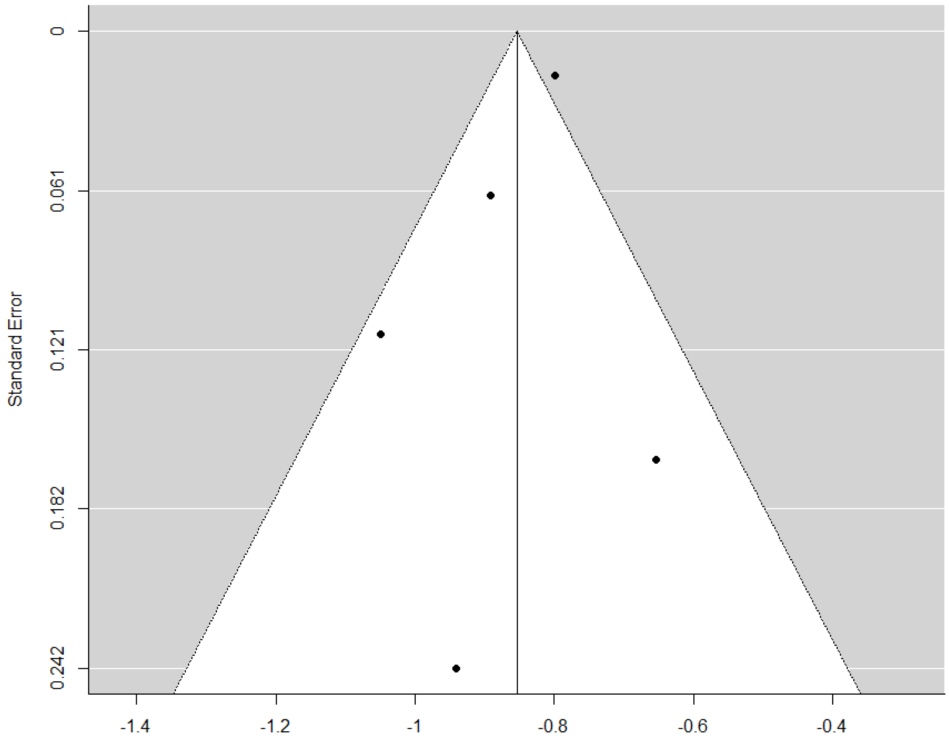

Suppl. Figure S82. Funnel plot of female patients (reference) and dialysis initiation with arteriovenous fistula *vs*. arteriovenous graft.

6.3. Successful arteriovenous fistula use

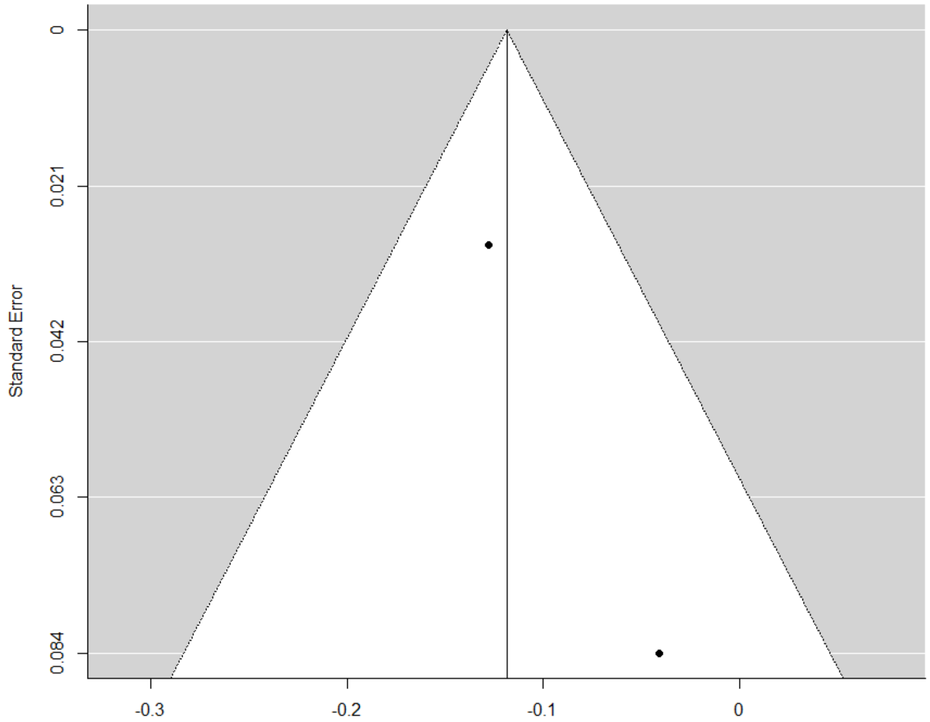

Suppl. Figure S83. Funnel plot comparing Black and White patients (reference) regarding successful arteriovenous fistula use.

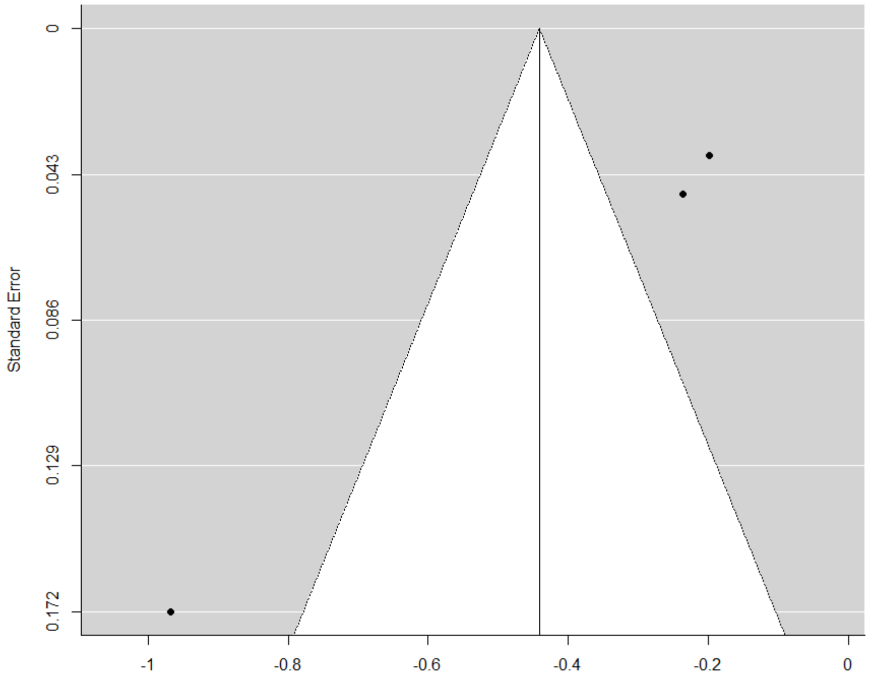

Suppl. Figure S84. Funnel plot of female patients (reference) and successful arteriovenous fistula.

6.4. Primary arteriovenous fistula patency

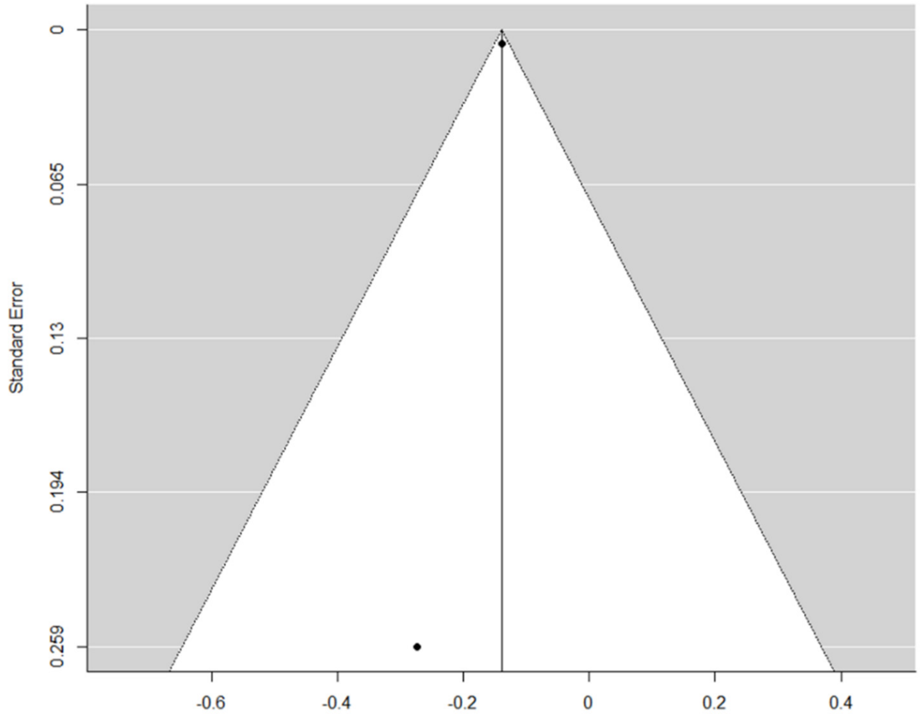

Suppl. Figure S85. Funnel plot comparing Black and White patients (reference) regarding primary arteriovenous fistula patency.

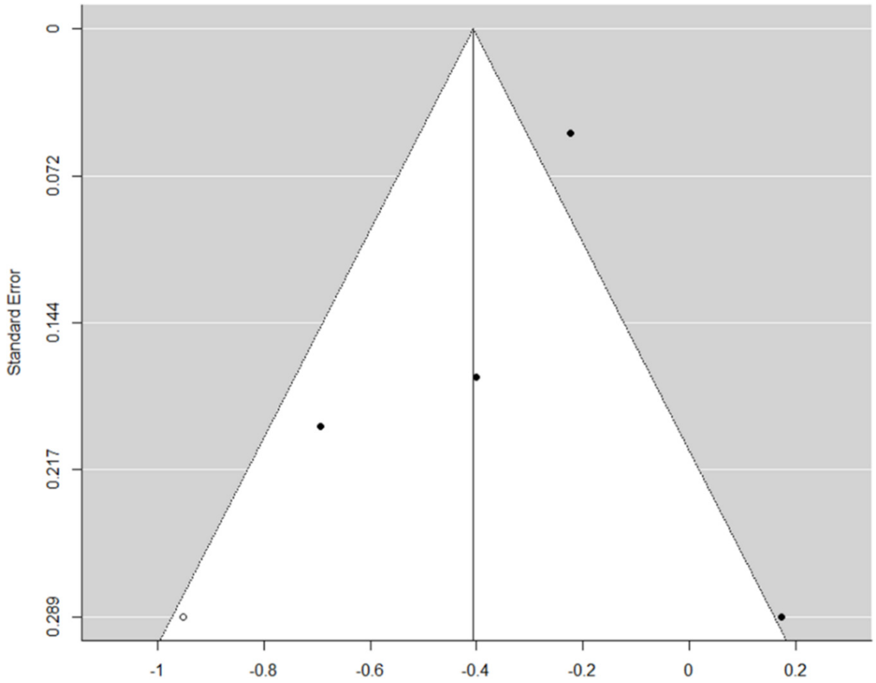

Suppl. Figure S86. Funnel plot of female patients (reference) and primary arteriovenous fistula patency.

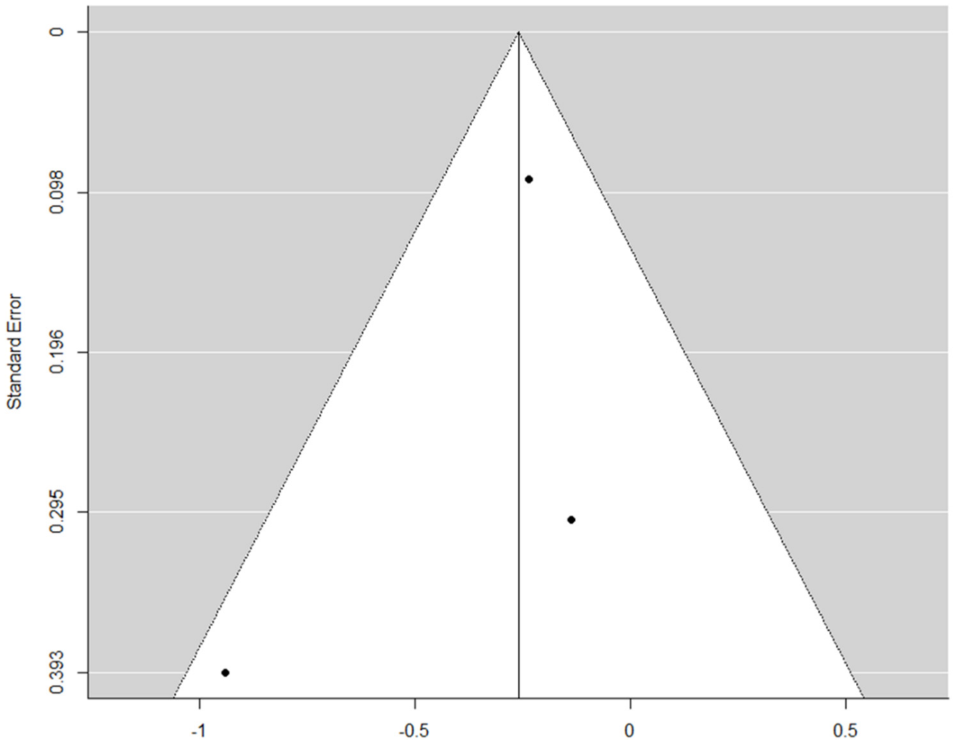

Suppl. Figure S87. Funnel plot comparing low and high socioeconomic status patients (reference) regarding primary arteriovenous fistula patency.

6.5. Transition from central venous catheter to arteriovenous fistula/graft

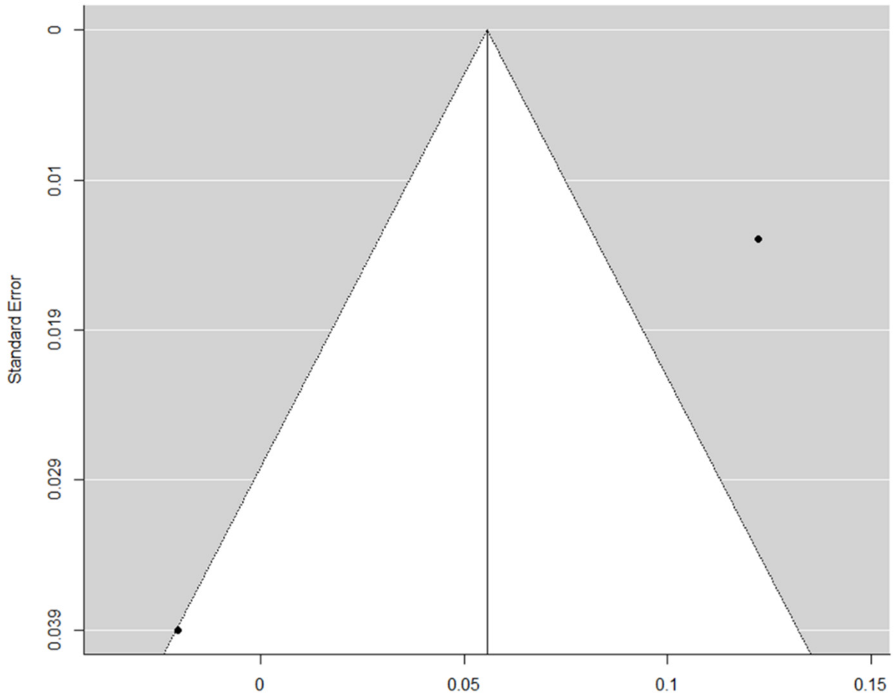

Suppl. Figure S88. Funnel plot comparing Black and White patients (reference) regarding the transition from central venous catheter to arteriovenous fistula/graft.

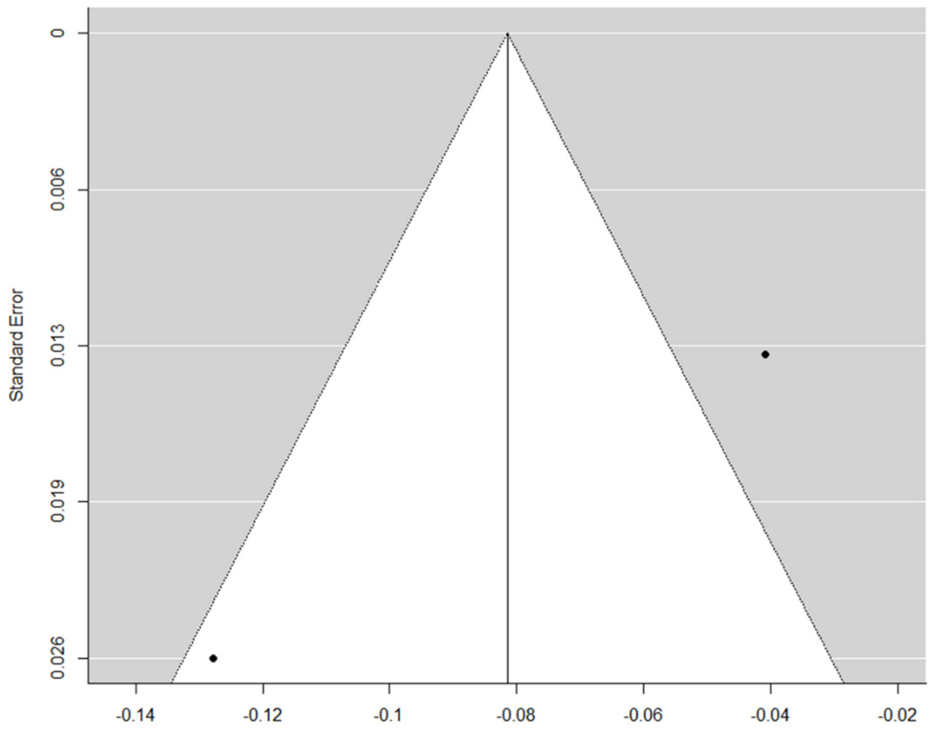

Suppl. Figure S89. Funnel plot of female patients (reference) and the transition from central venous catheter to arteriovenous fistula/graft.

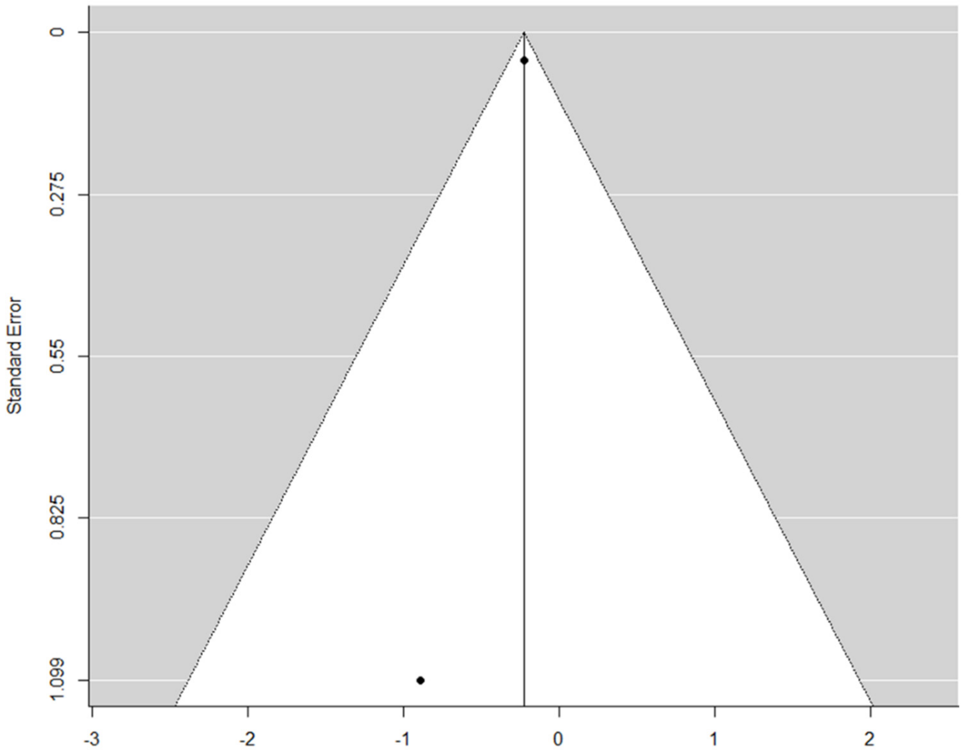

Suppl. Figure S90. Funnel plot comparing low and high socioeconomic status patients (reference) regarding the transition from central venous catheter to arteriovenous fistula/graft.

6.6. Home dialysis

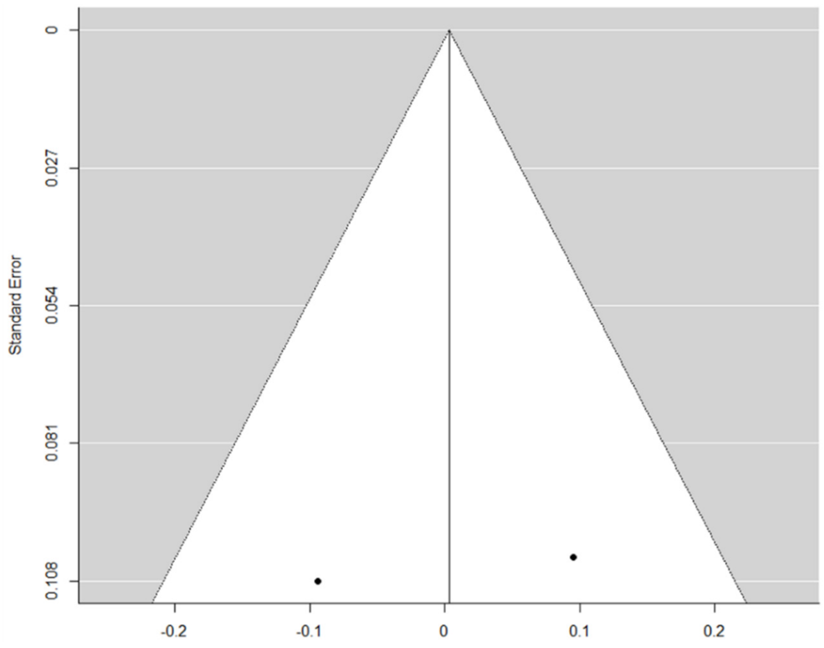

Suppl. Figure S91. Funnel plot comparing Black and White patients (reference) regarding home dialysis.

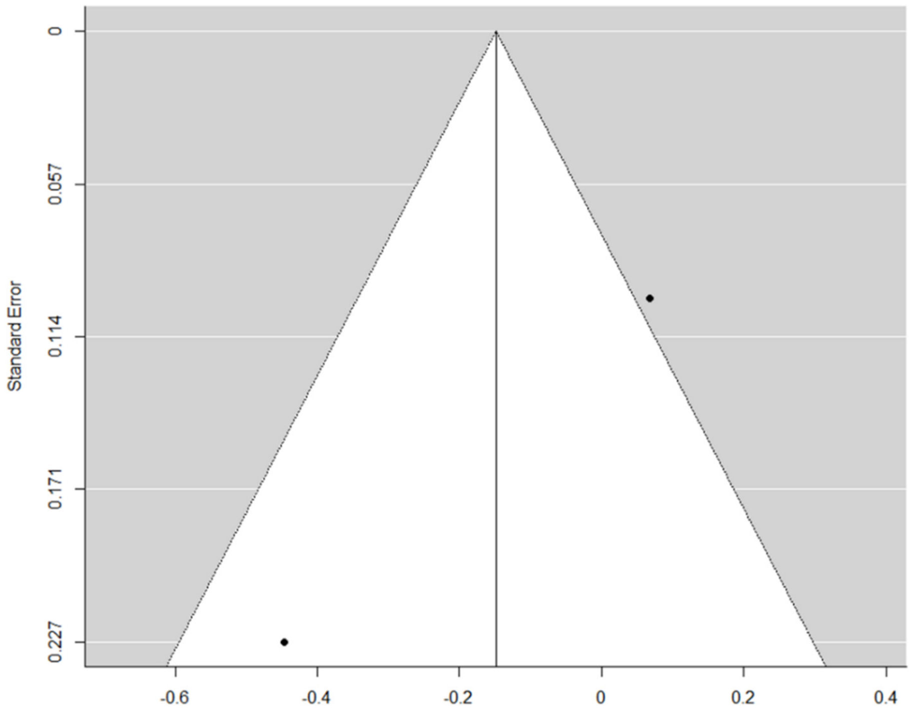

Suppl. Figure S92. Funnel plot comparing Asian and White patients (reference) regarding home dialysis.

6.7. Peritoneal dialysis

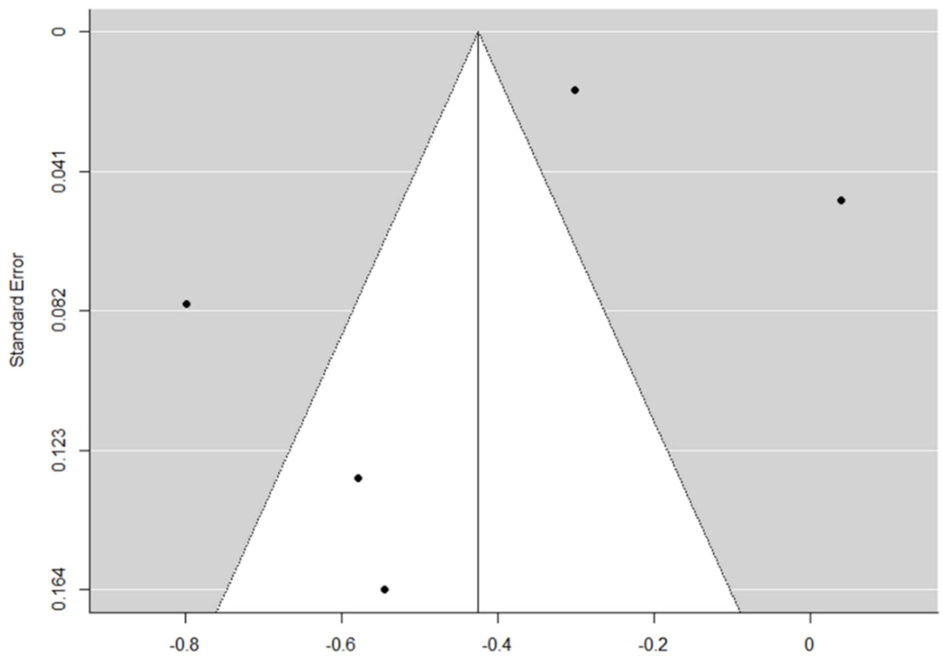

Suppl. Figure S93. Funnel plot comparing Black and White patients (reference) regarding peritoneal dialysis use.

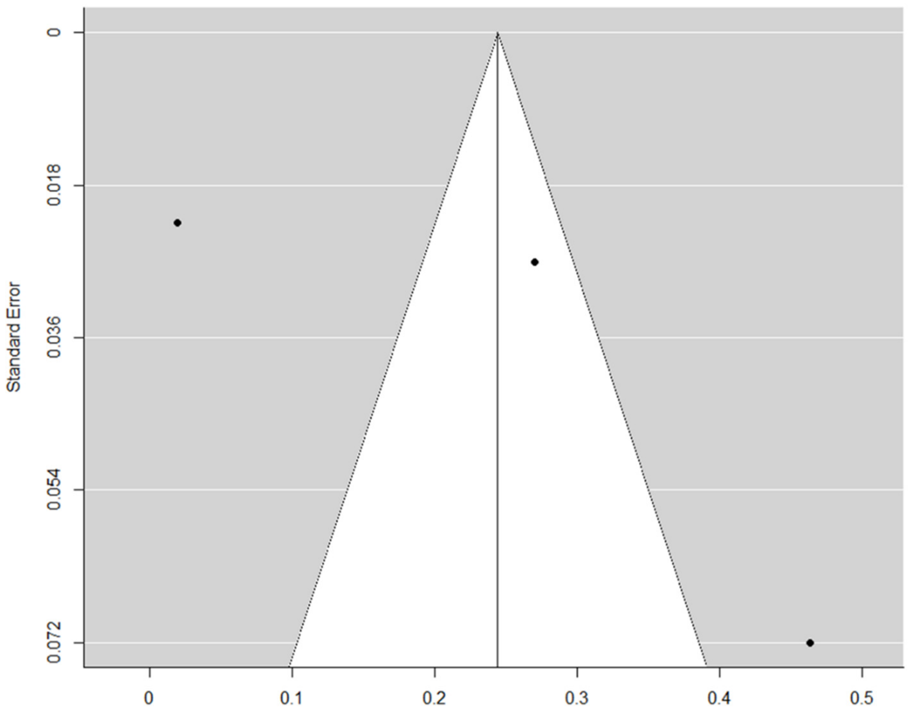

Suppl. Figure S94. Funnel plot comparing Asian and White patients (reference) regarding peritoneal dialysis use.

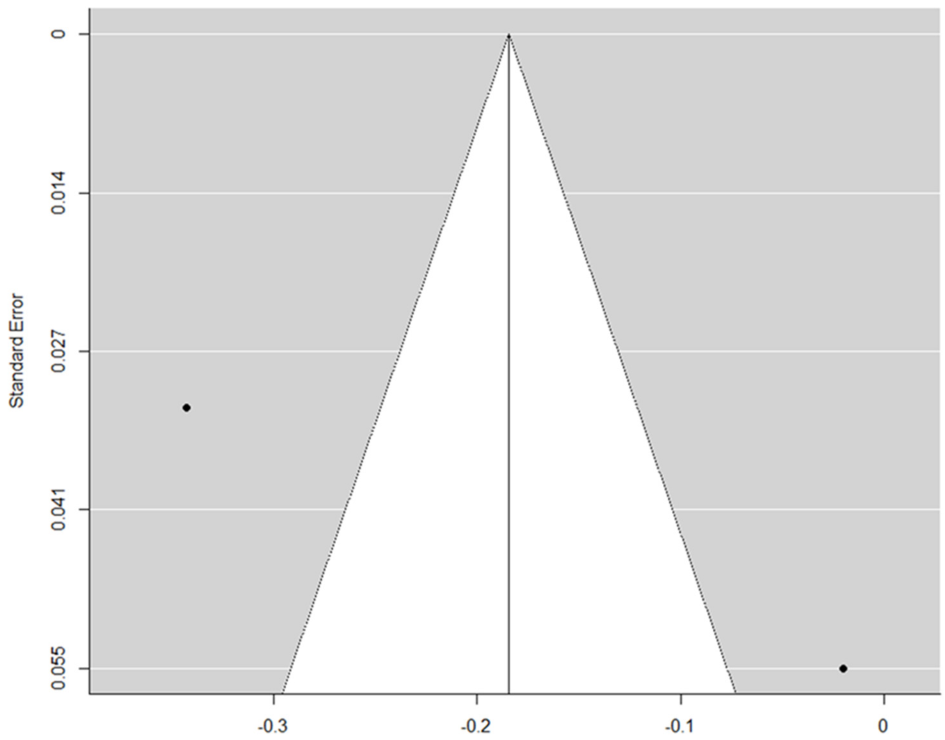

Suppl. Figure S95. Funnel plot comparing Indigenous and White patients (reference) regarding peritoneal dialysis use.

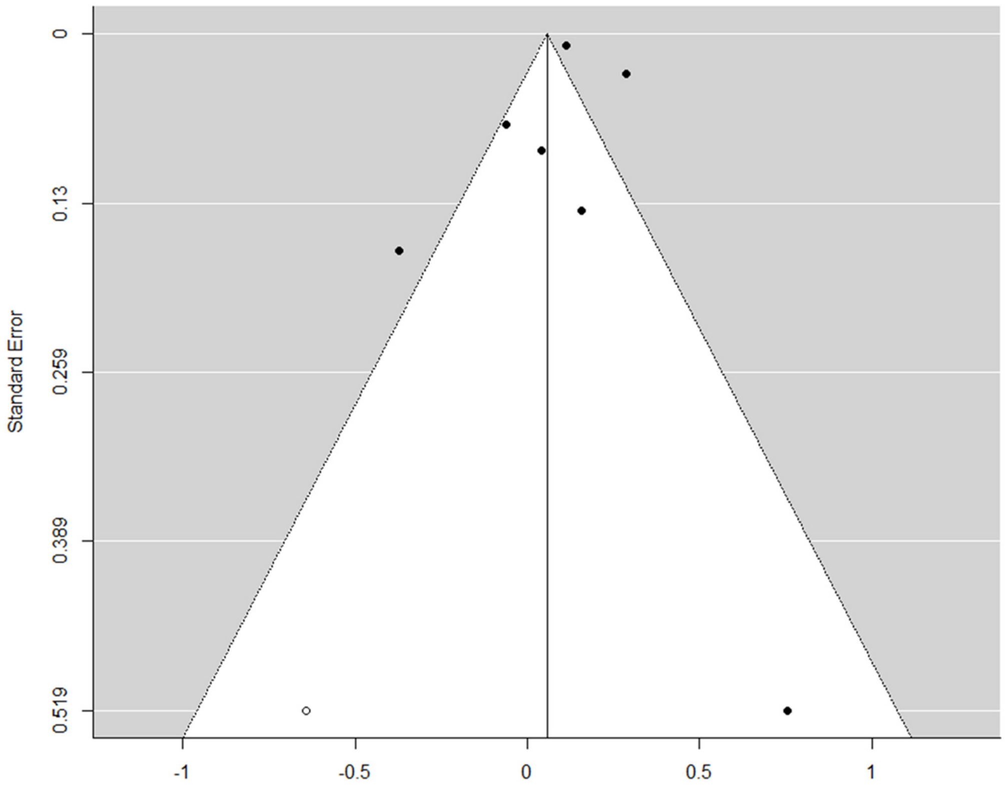

Suppl. Figure S96. Funnel plot of female patients (reference) and peritoneal dialysis use.

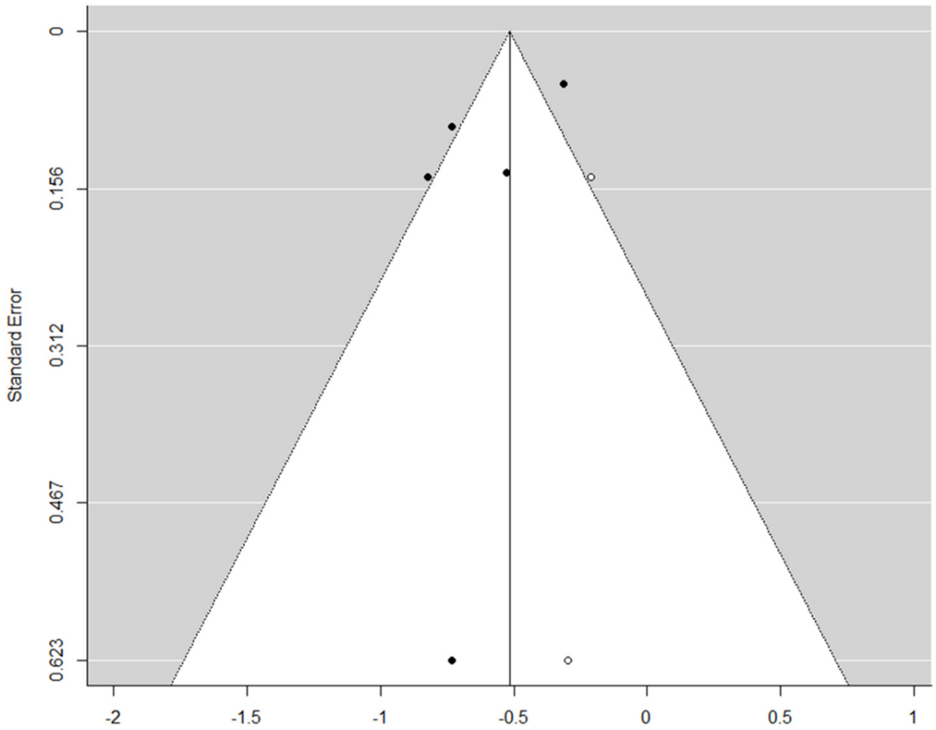

Suppl. Figure S97. Funnel plot comparing low and high socioeconomic status patients (reference) regarding peritoneal dialysis use.

6.8. Dialysis survival

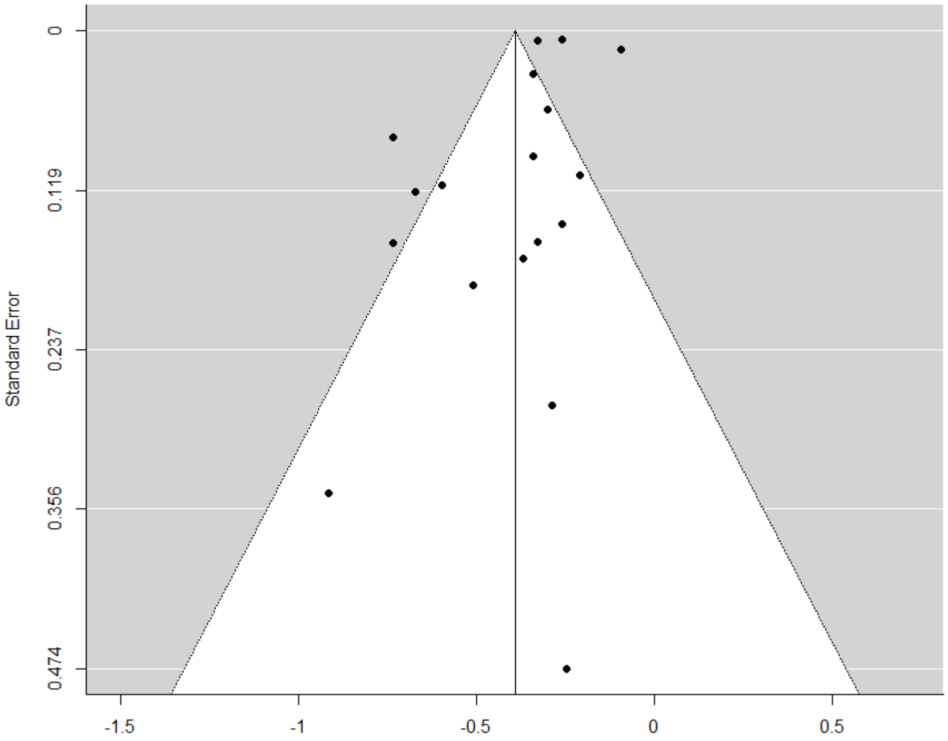

Suppl. Figure S98. Funnel plot comparing Black and White patients (reference) regarding dialysis mortality.

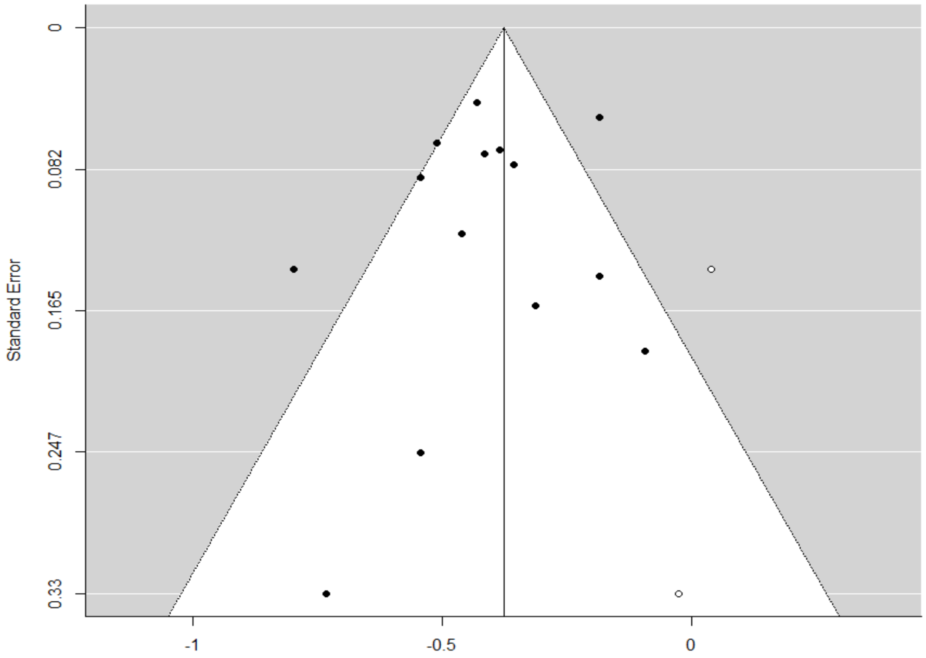

Suppl. Figure S99. Funnel plot comparing Asian and White patients (reference) regarding dialysis mortality.

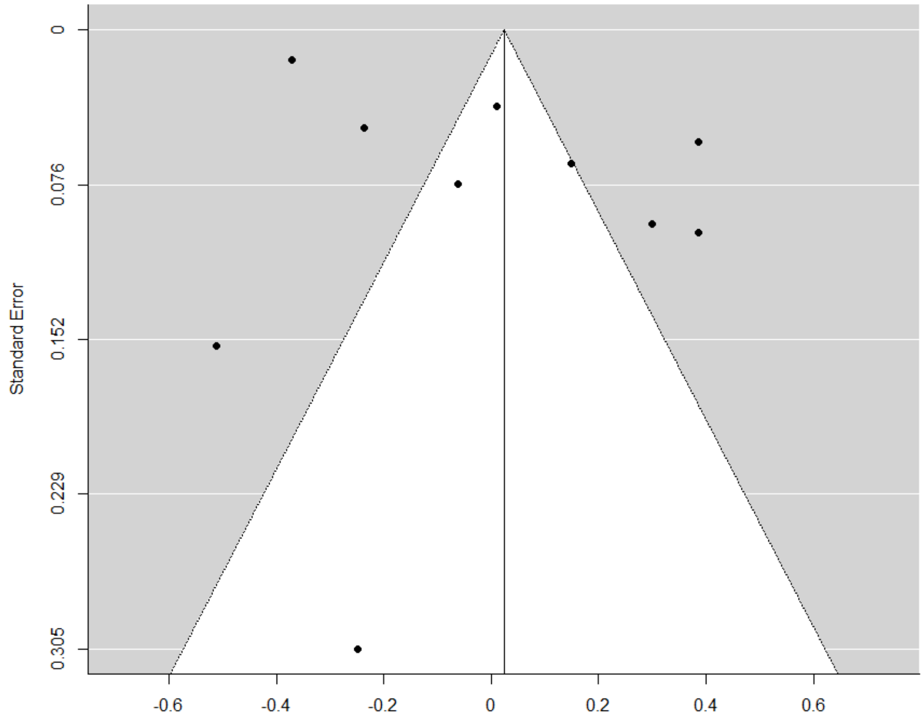

Suppl. Figure S100. Funnel plot comparing Indigenous and White patients (reference) regarding dialysis mortality.

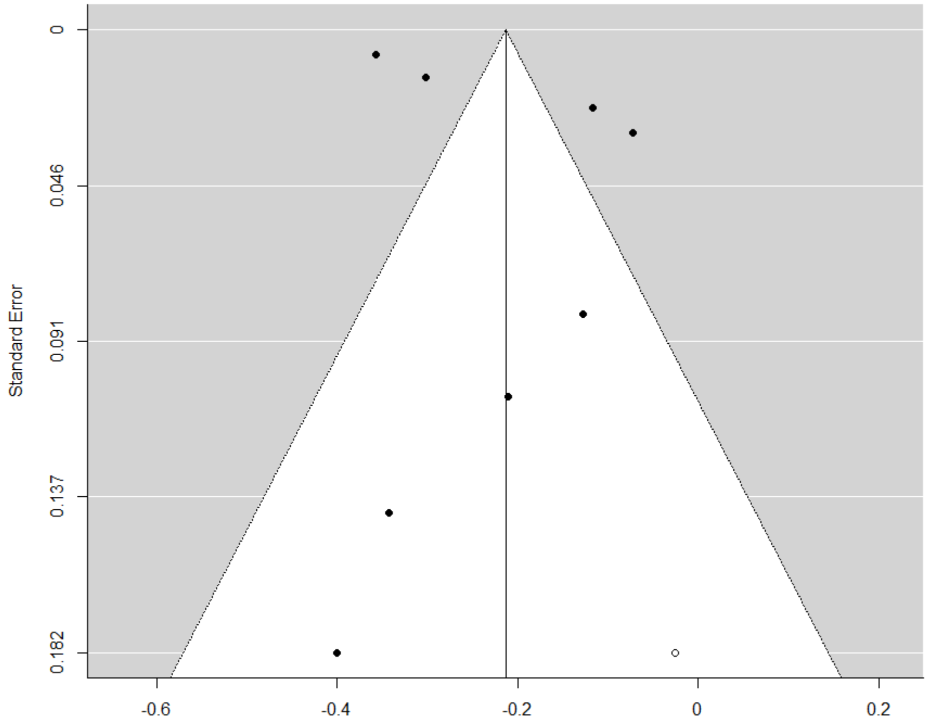

Suppl. Figure S101. Funnel plot comparing Hispanic and White patients (reference) regarding dialysis mortality.

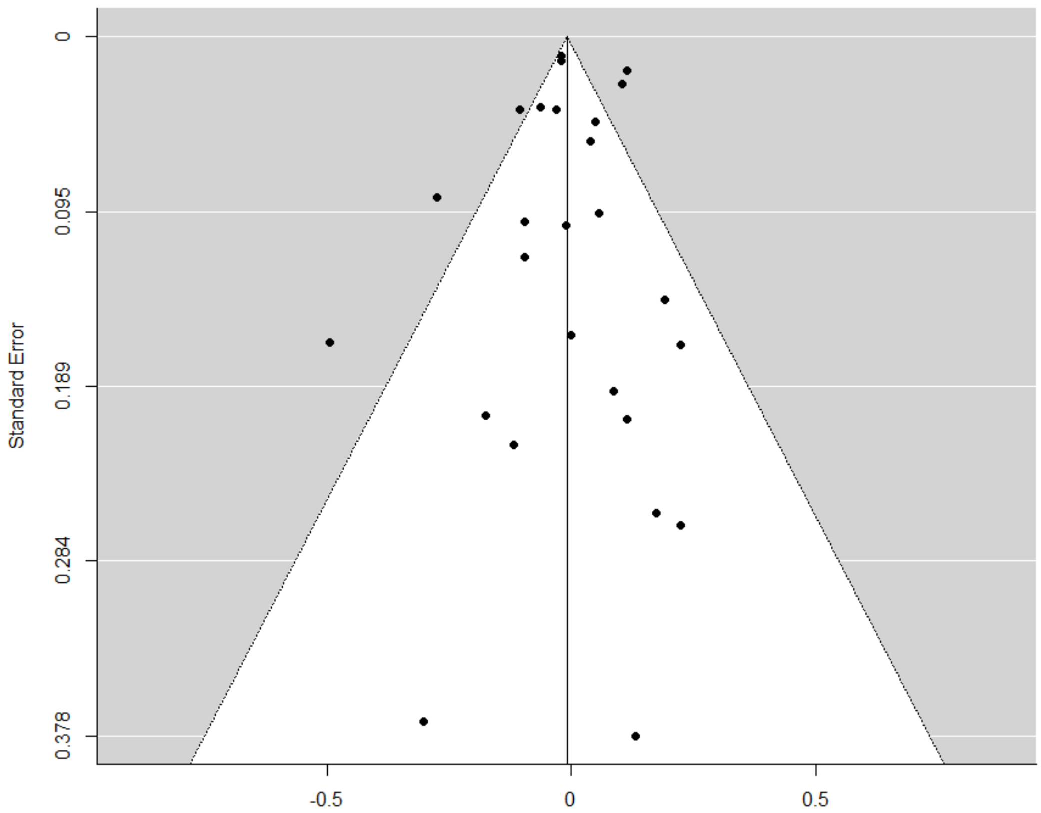

Suppl. Figure S102. Funnel plot of female patients (reference) and dialysis mortality.

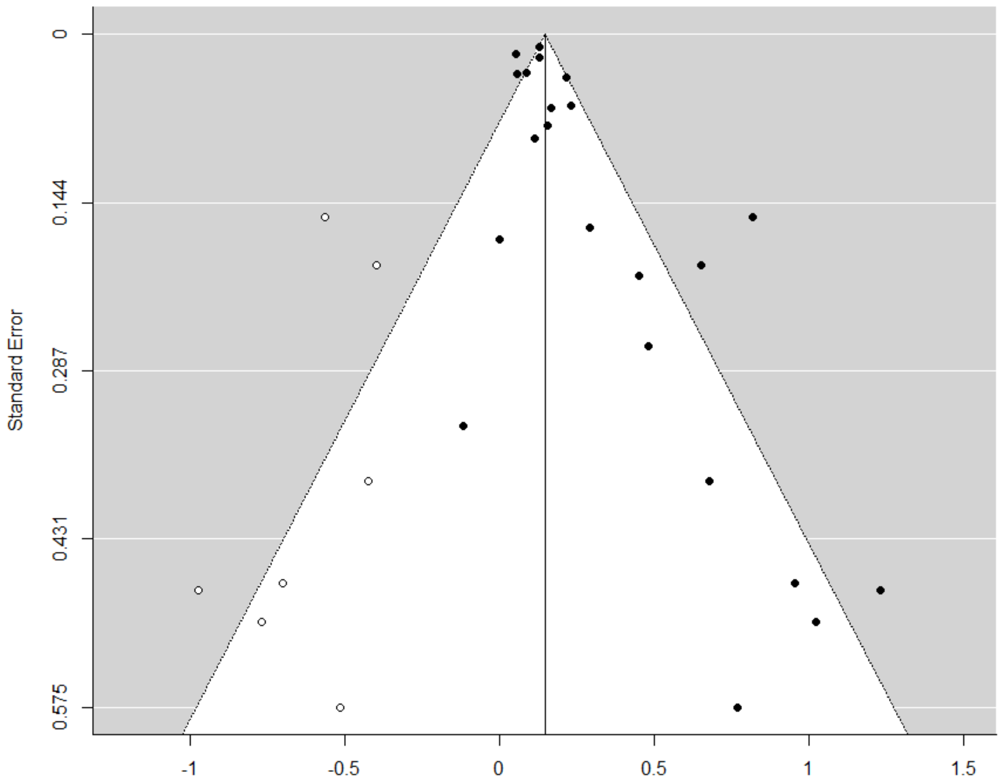

Suppl. Figure S103. Funnel plot comparing low and high socioeconomic status patients (reference) regarding dialysis mortality.

6.9. Waitlisting

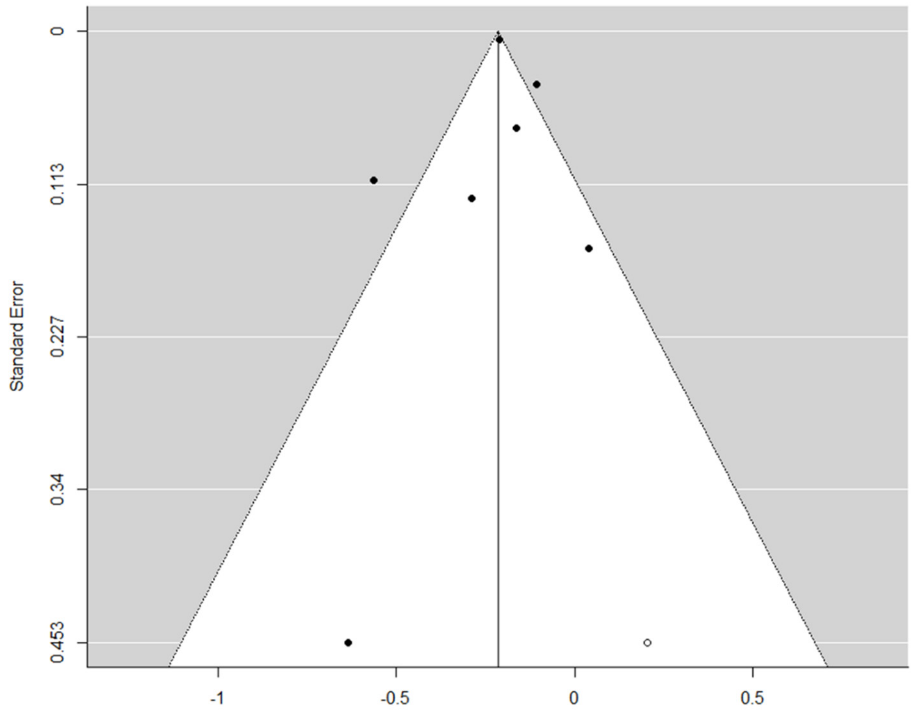

Suppl. Figure S104. Funnel plot comparing Black and White patients (reference) regarding waitlisting.

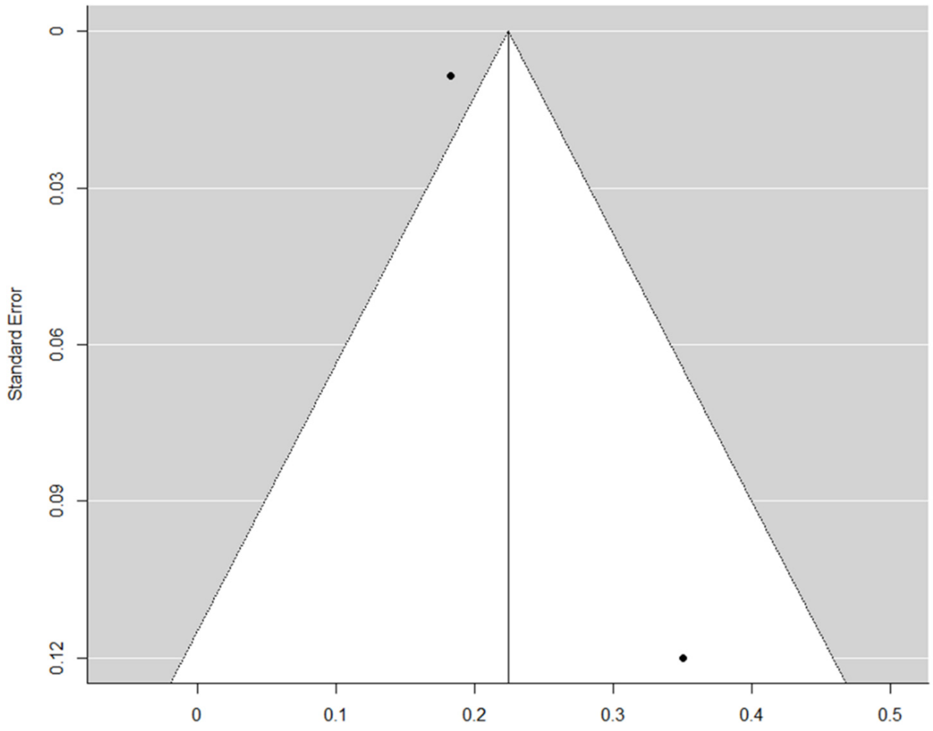

Suppl. Figure S105. Funnel plot comparing Asian and White patients (reference) regarding waitlisting.

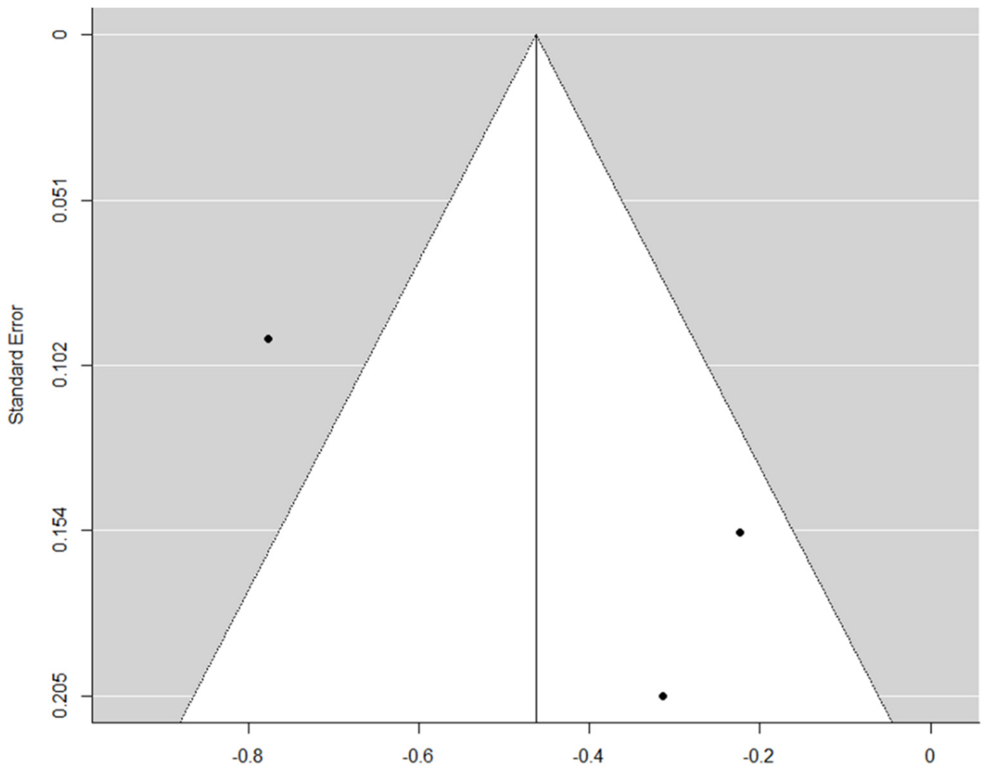

Suppl. Figure S106. Funnel plot comparing Indigenous and White patients (reference) regarding waitlisting.

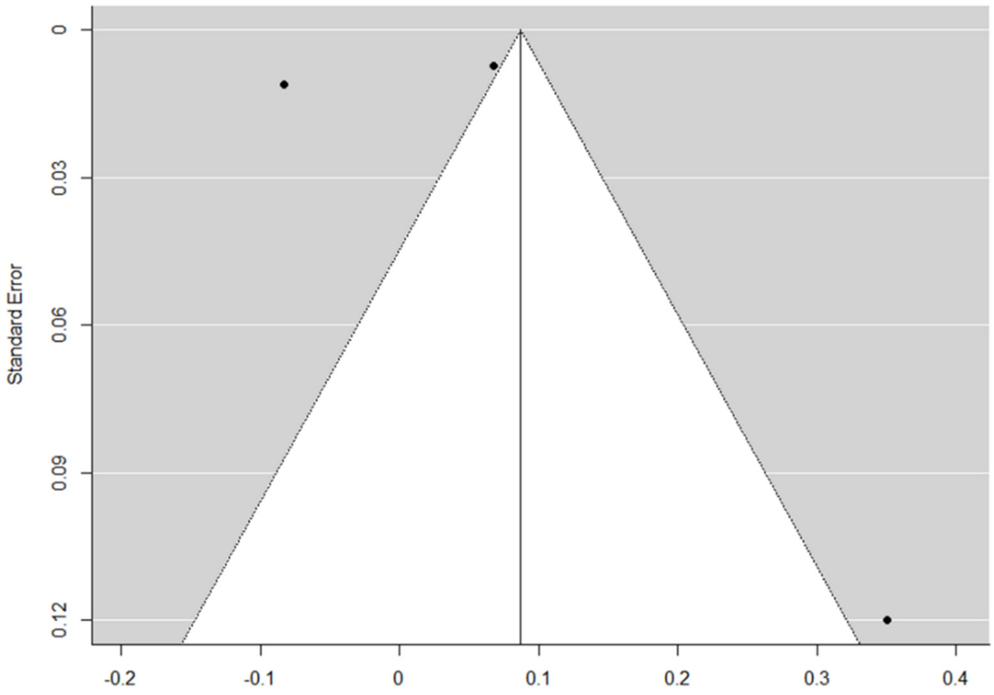

Suppl. Figure S107. Funnel plot comparing Hispanic and White patients (reference) regarding waitlisting.

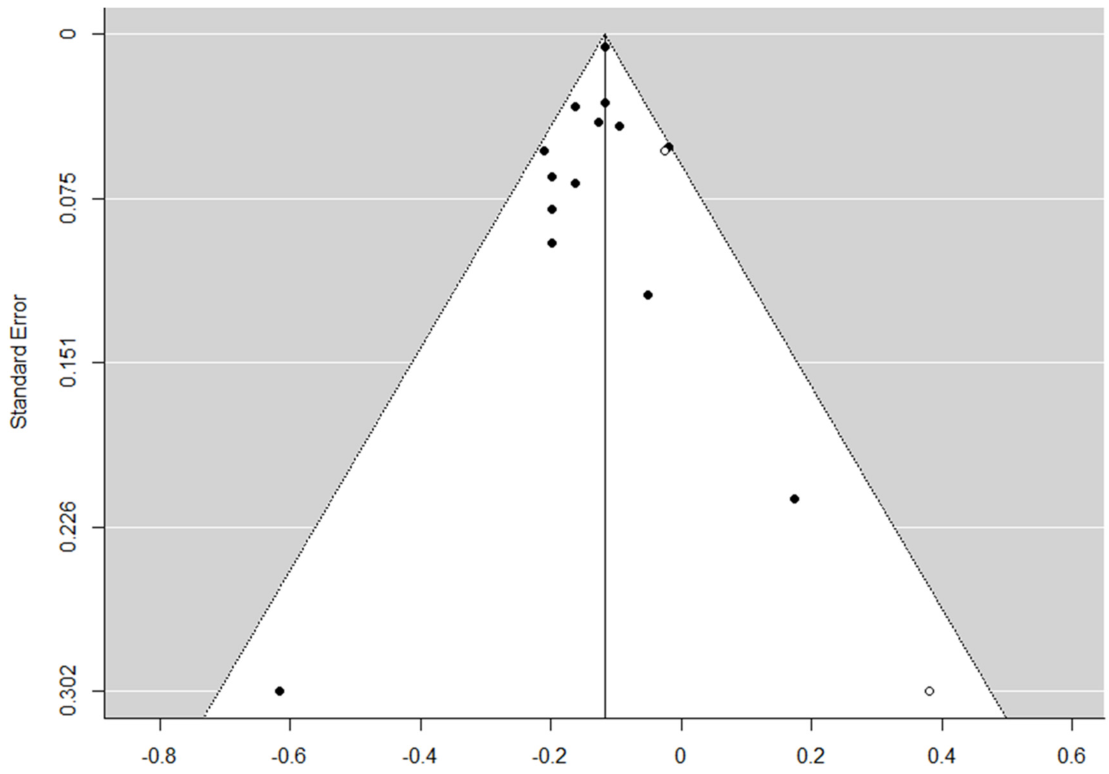

Suppl. Figure S108. Funnel plot of female patients (reference) and waitlisting.

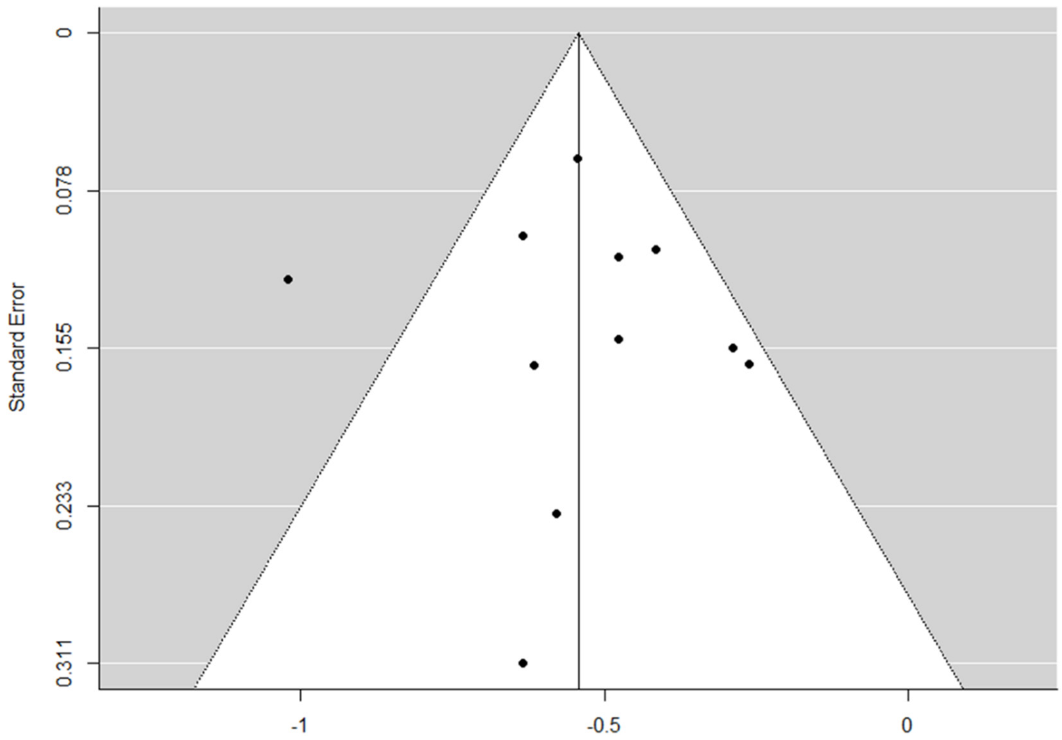

Suppl. Figure S109. Funnel plot comparing low and high socioeconomic status patients (reference) regarding waitlisting.

6.10. Kidney transplantation

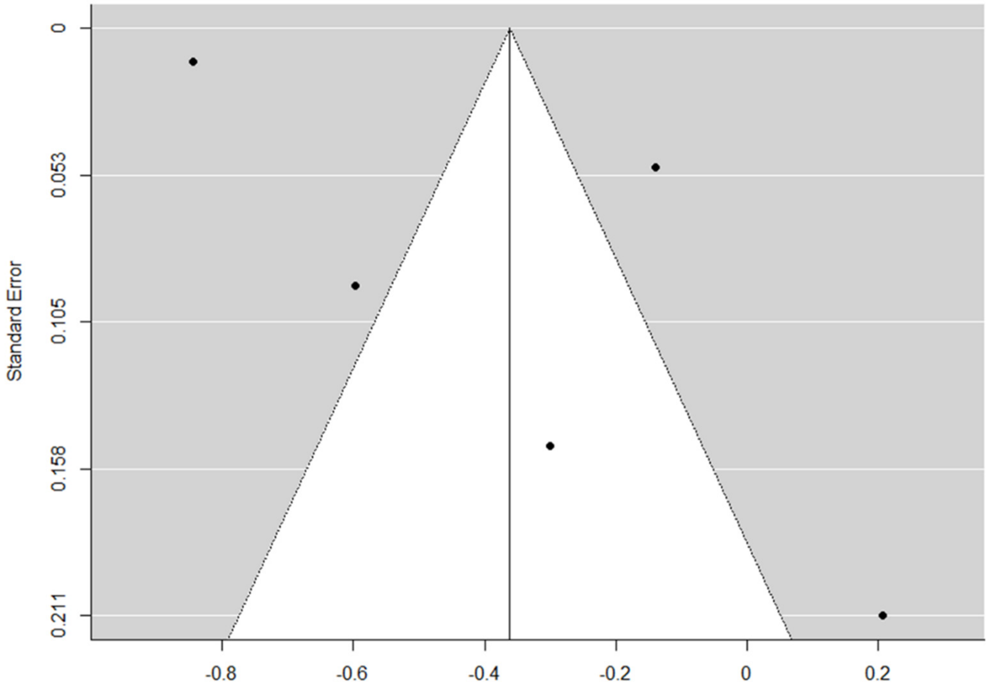

Suppl. Figure S110. Funnel plot comparing Black and White patients (reference) regarding kidney transplantation.

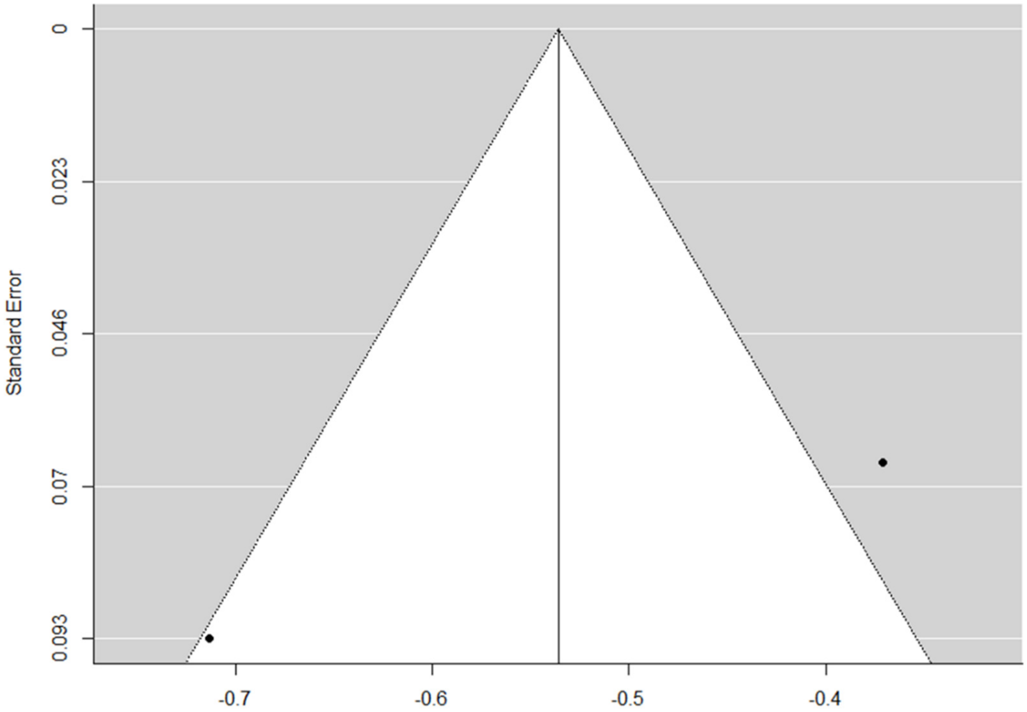

Suppl. Figure S111. Funnel plot comparing Asian and White patients (reference) regarding kidney transplantation.

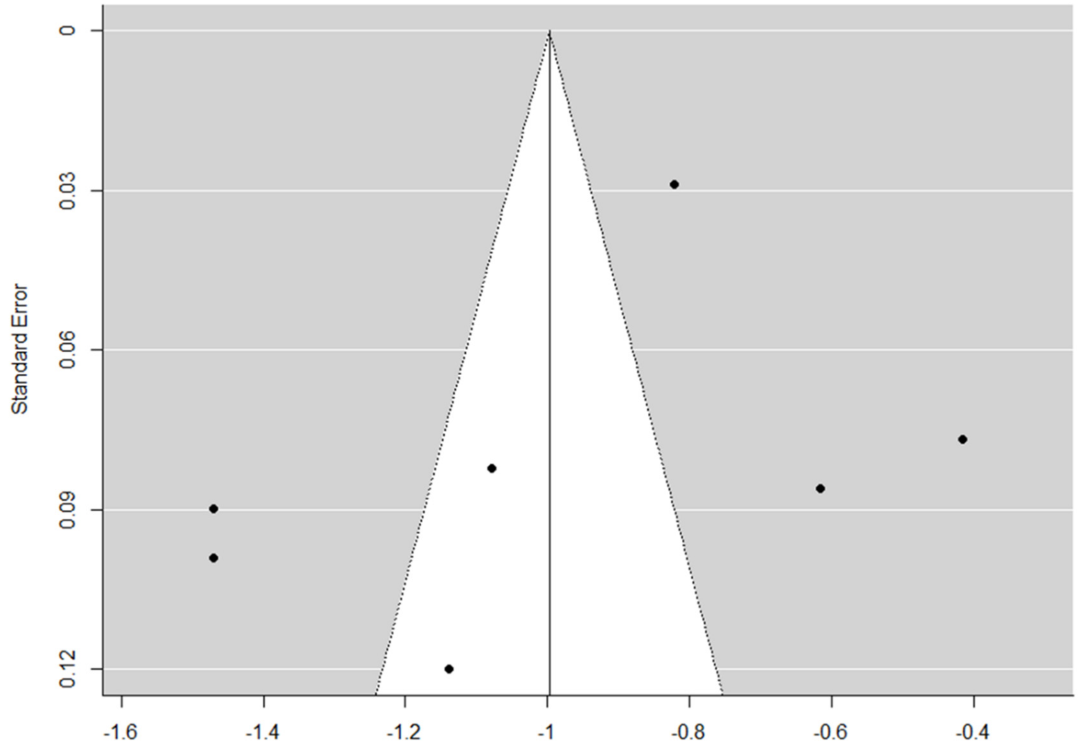

Suppl. Figure S112. Funnel plot comparing Indigenous and White patients (reference) regarding kidney transplantation.

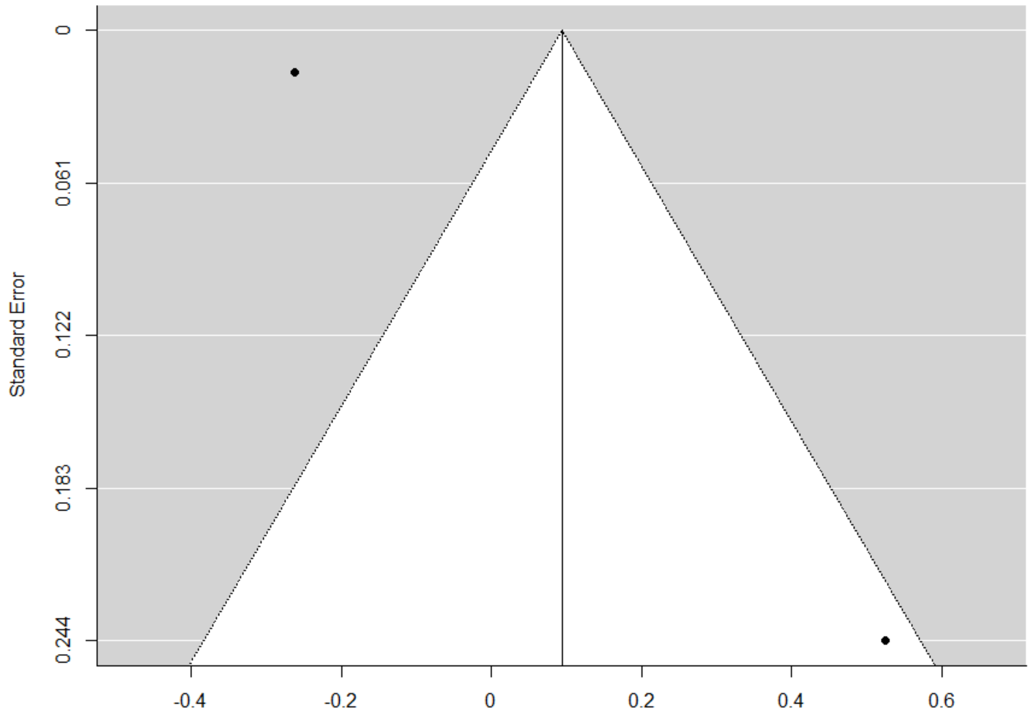

Suppl. Figure S113. Funnel plot comparing Hispanic and White patients (reference) regarding kidney transplantation.

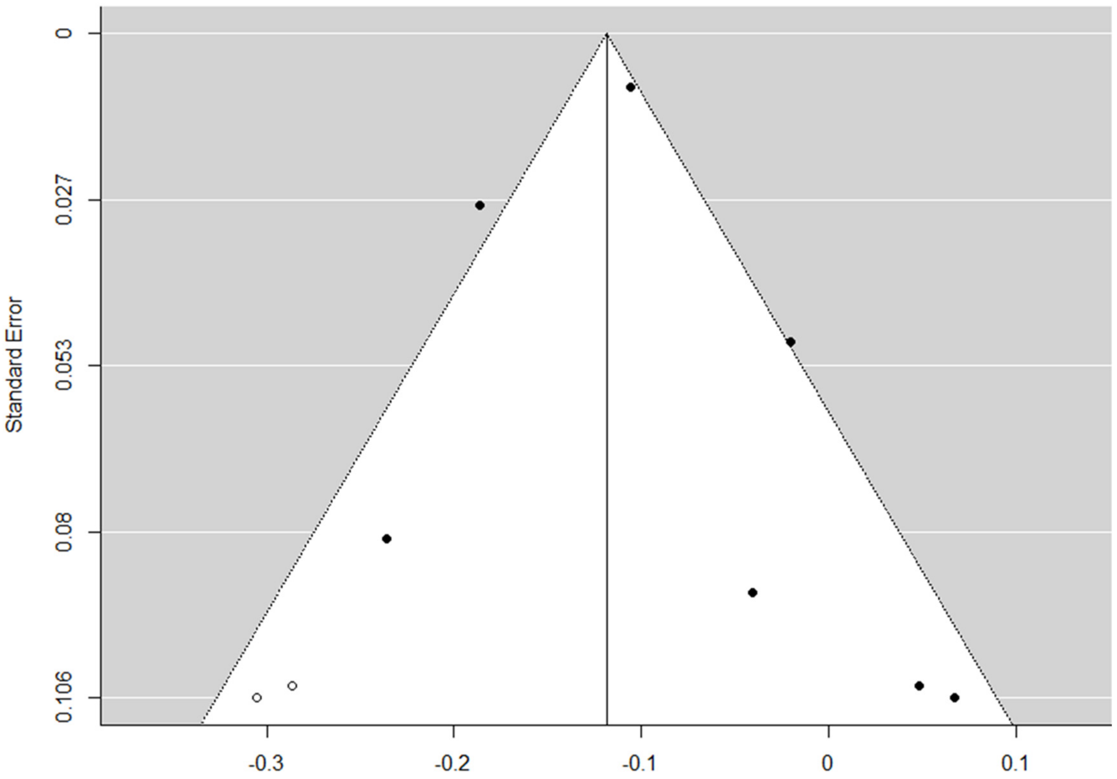

Suppl. Figure S114. Funnel plot of female patients (reference) and kidney transplantation.

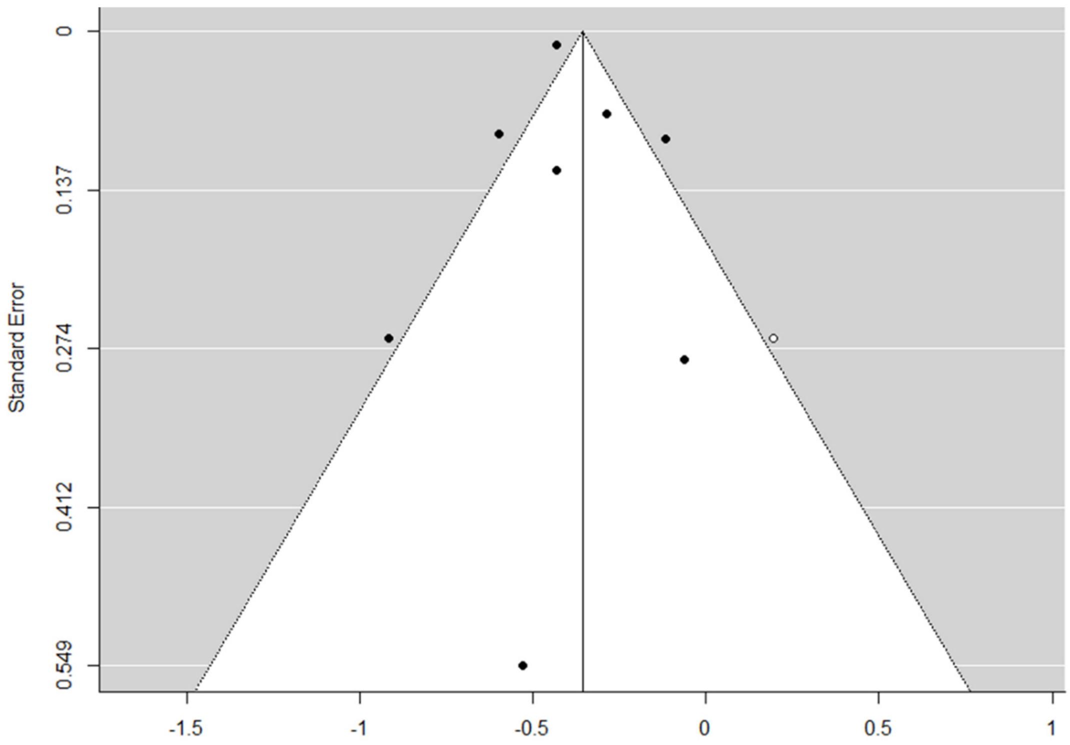

Suppl. Figure S115. Funnel plot comparing low and high socioeconomic status patients (reference) regarding kidney transplantation.

6.11. Living-donor transplantation

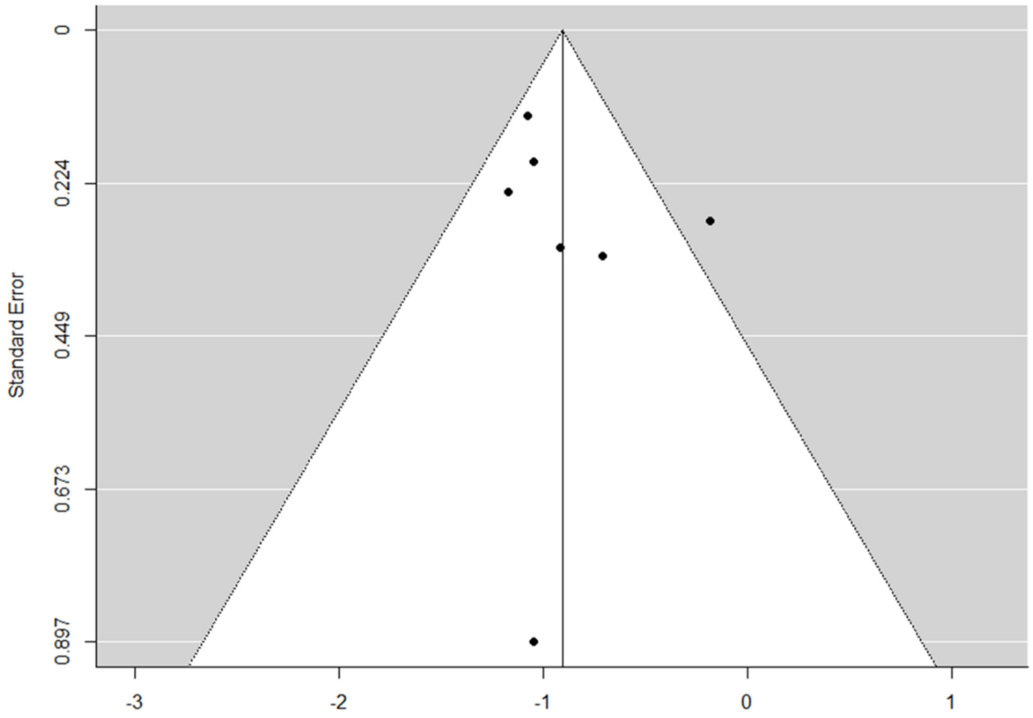

Suppl. Figure S116. Funnel plot comparing Black and White patients (reference) regarding living-donor transplantation.

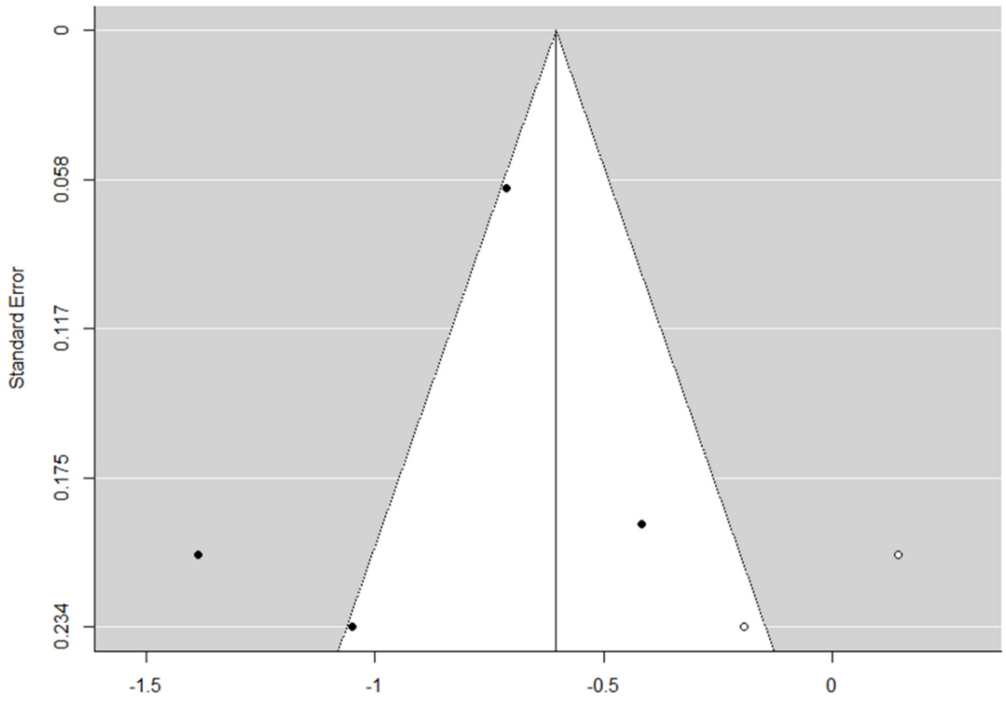

Suppl. Figure S117. Funnel plot comparing Asian and White patients (reference) regarding living-donor transplantation.

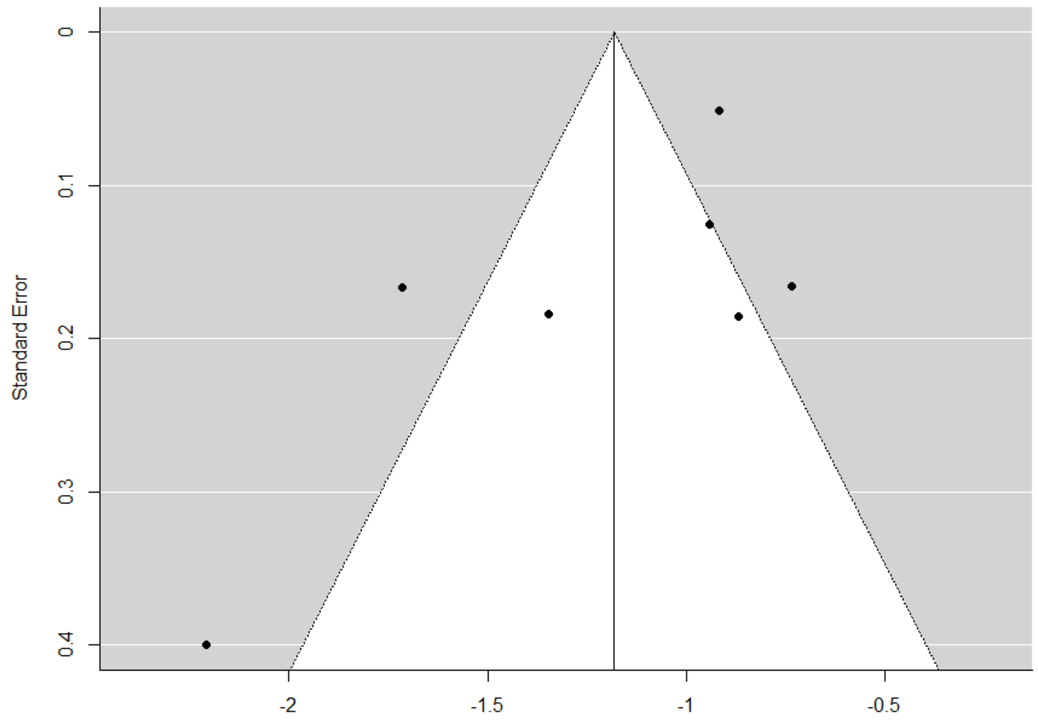

Suppl. Figure S118. Funnel plot comparing Indigenous and White patients (reference) regarding living-donor transplantation.

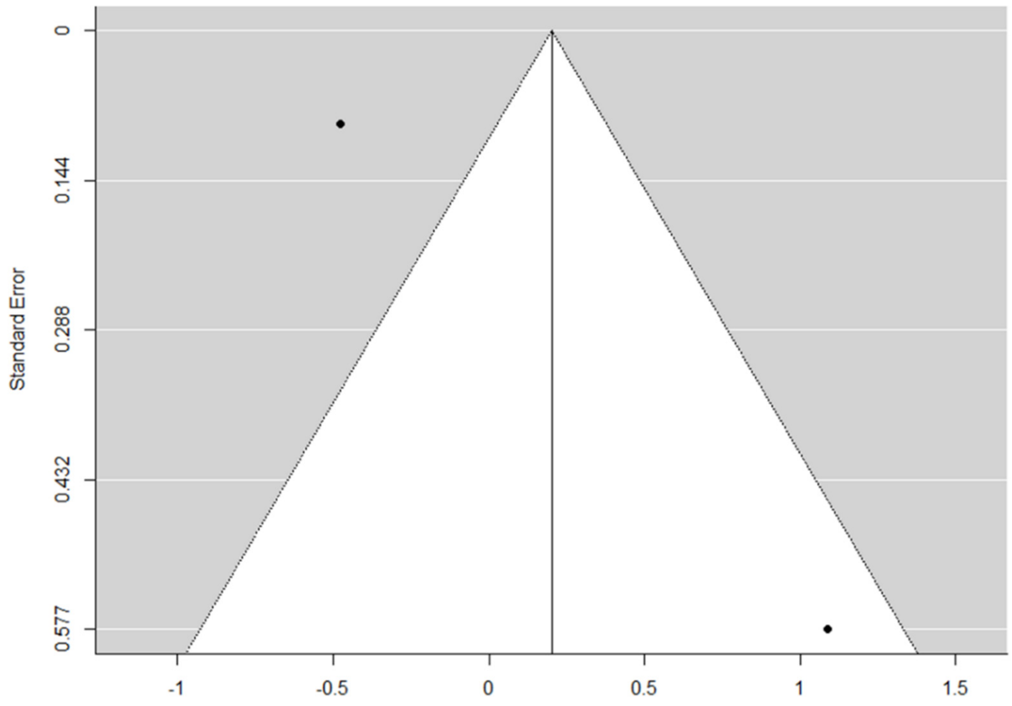

Suppl. Figure S119. Funnel plot comparing Hispanic and White patients (reference) regarding living-donor transplantation.

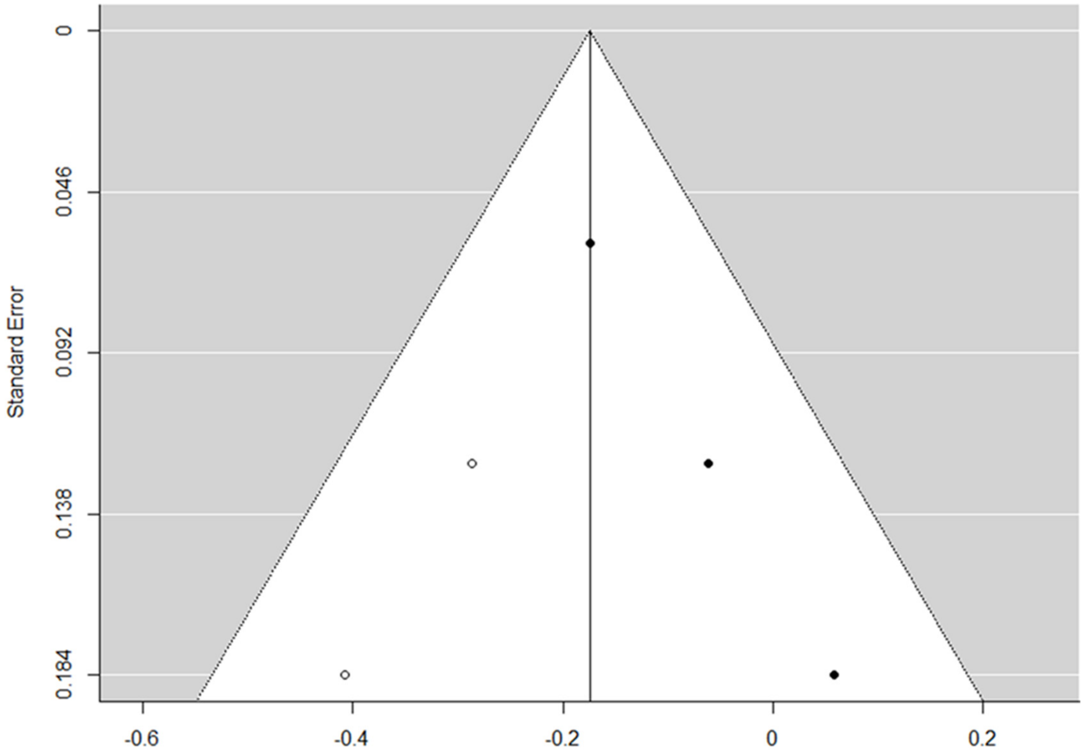

Suppl. Figure S120. Funnel plot of female patients (reference) and living-donor transplantation.

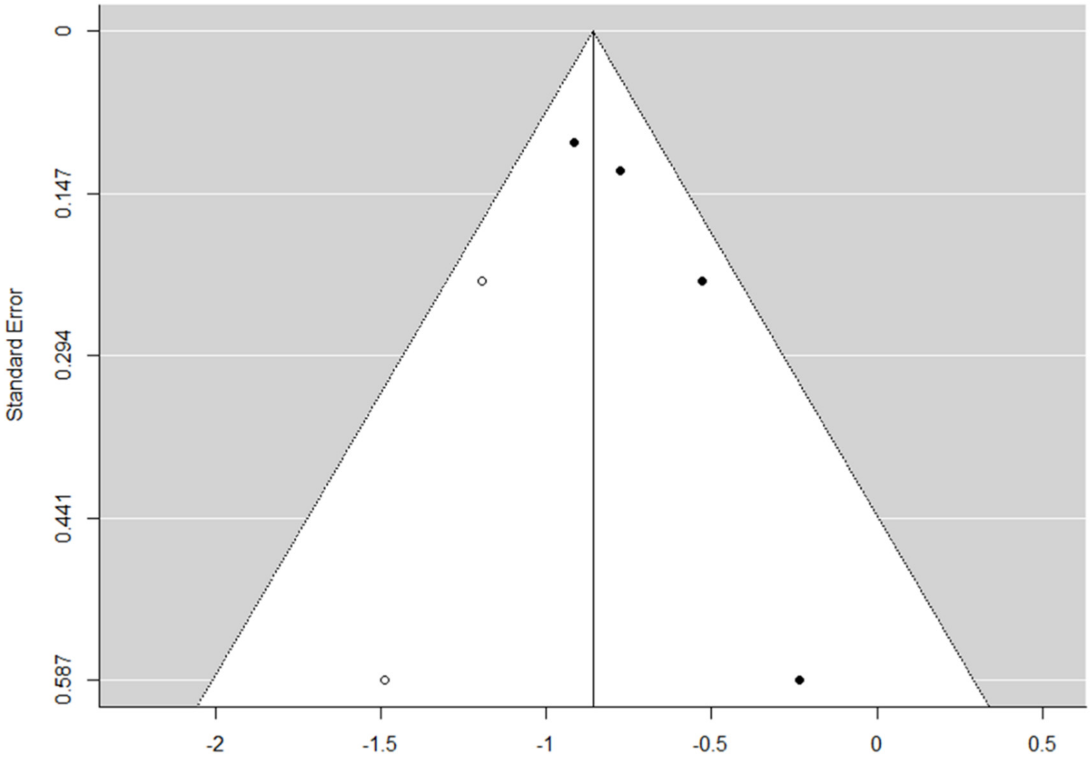

Suppl. Figure S121. Funnel plot comparing low and high socioeconomic status patients (reference) regarding living-donor transplantation.

6.12. Deceased-donor transplantation

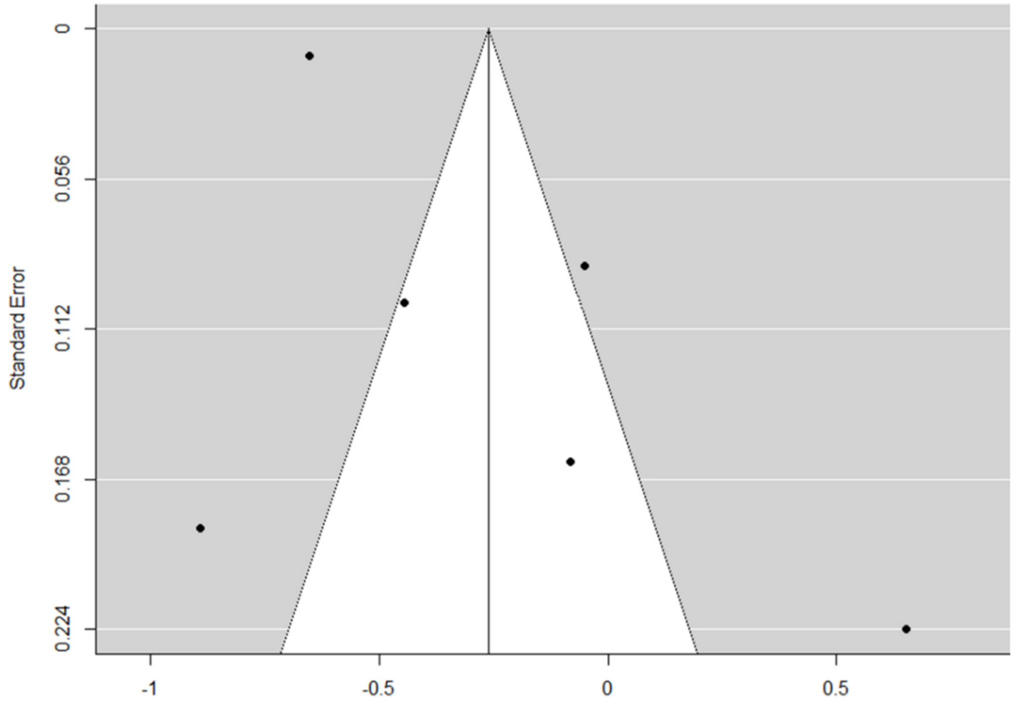

Suppl. Figure S122. Funnel plot comparing Black and White patients (reference) regarding deceased-donor transplantation.

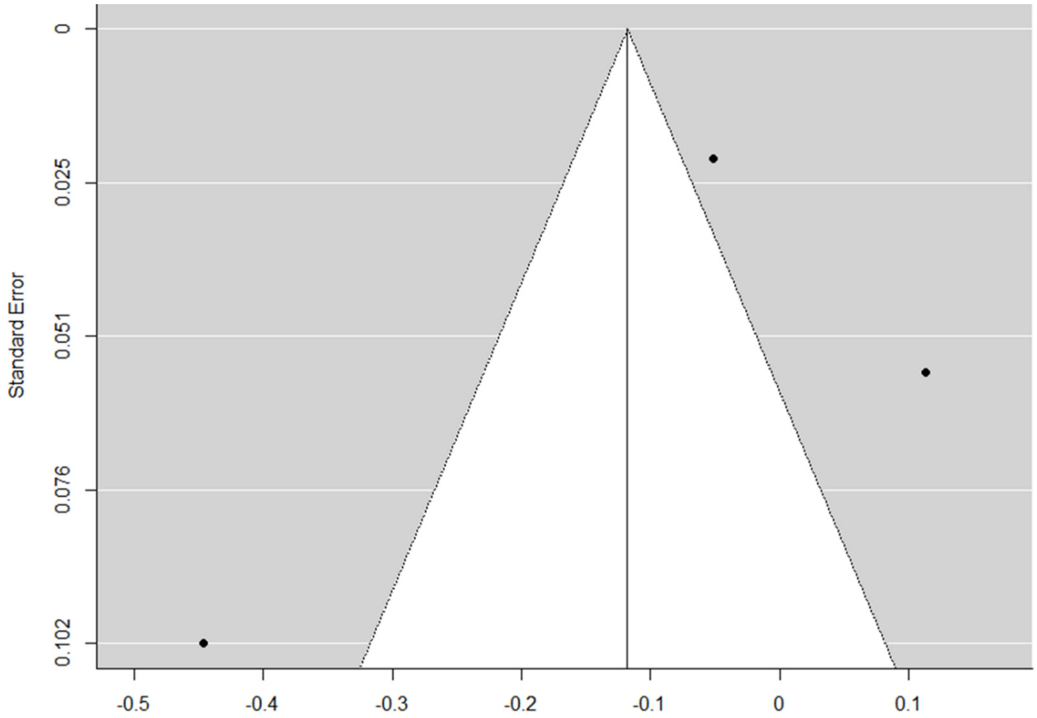

Suppl. Figure S123. Funnel plot comparing Asian and White patients (reference) regarding deceased-donor transplantation.

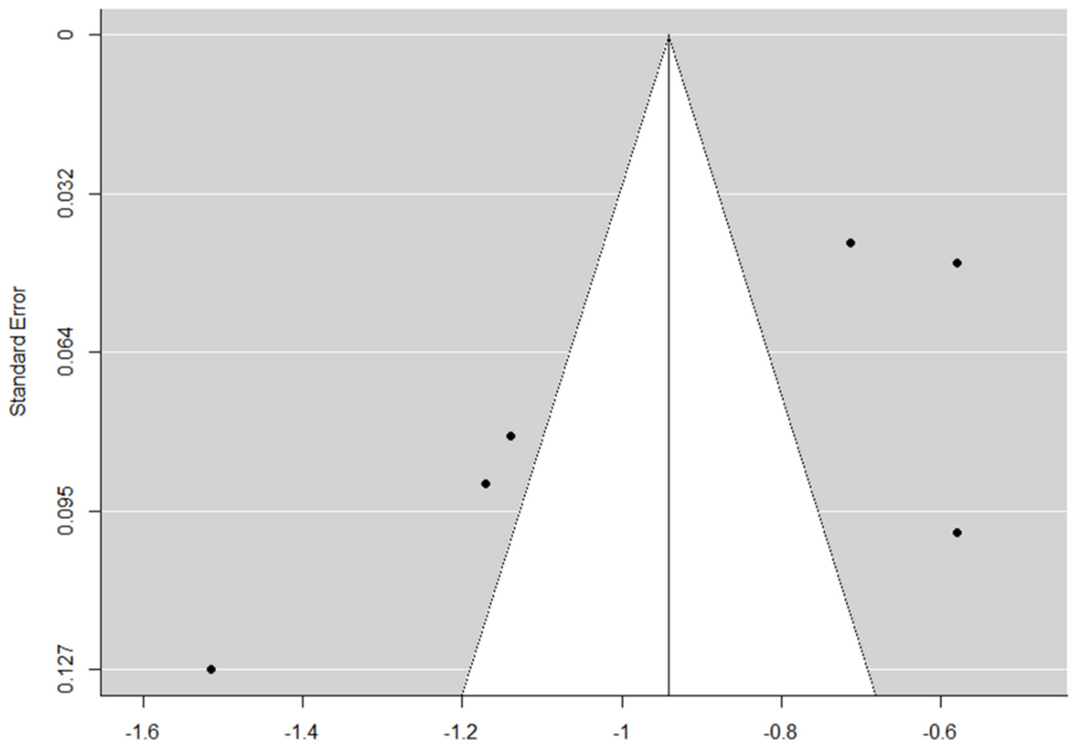

Suppl. Figure S124. Funnel plot comparing Indigenous and White patients (reference) regarding deceased-donor transplantation.

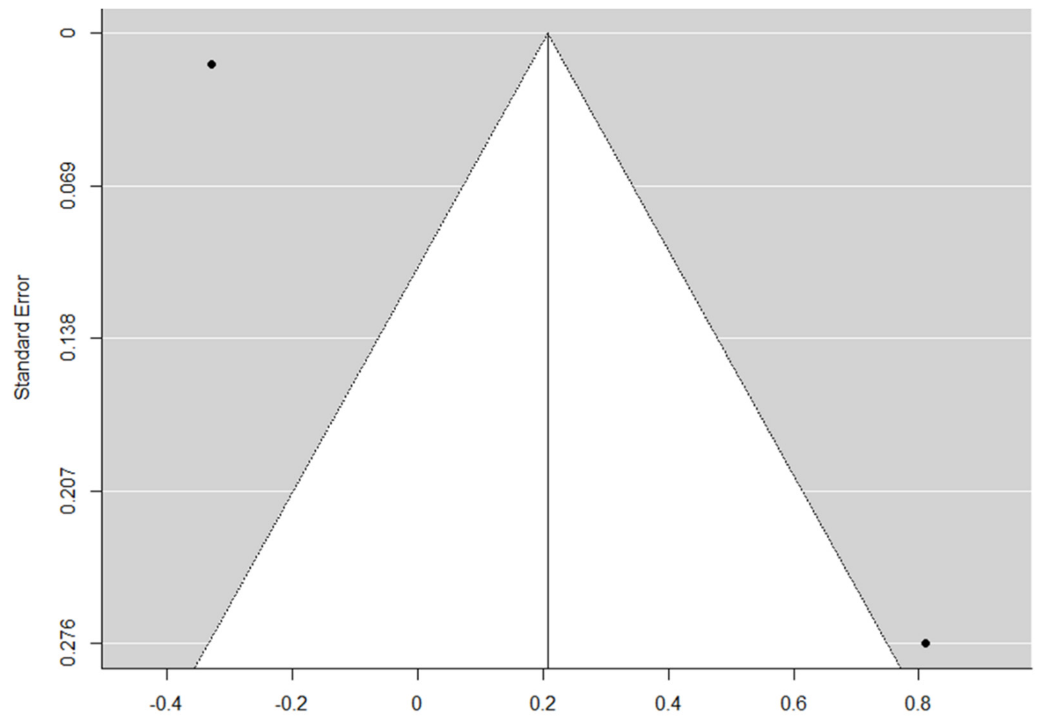

Suppl. Figure S125. Funnel plot comparing Hispanic and White patients (reference) regarding deceased-donor transplantation.

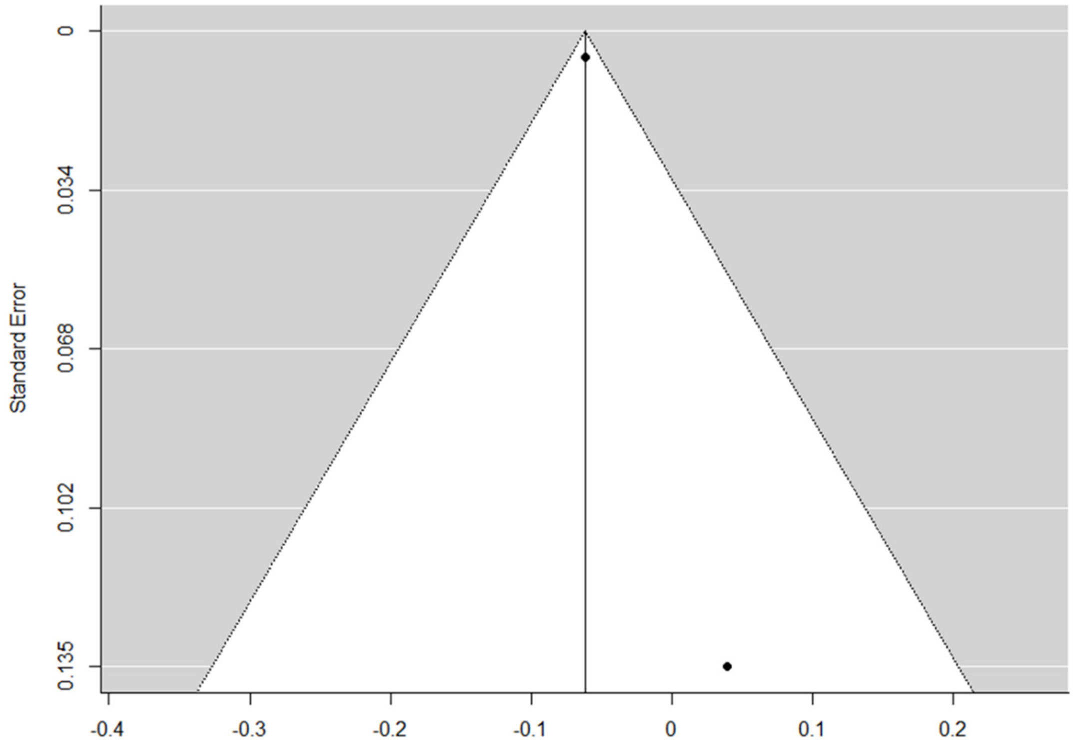

Suppl. Figure S126. Funnel plot of female patients (reference) and deceased-donor transplantation.

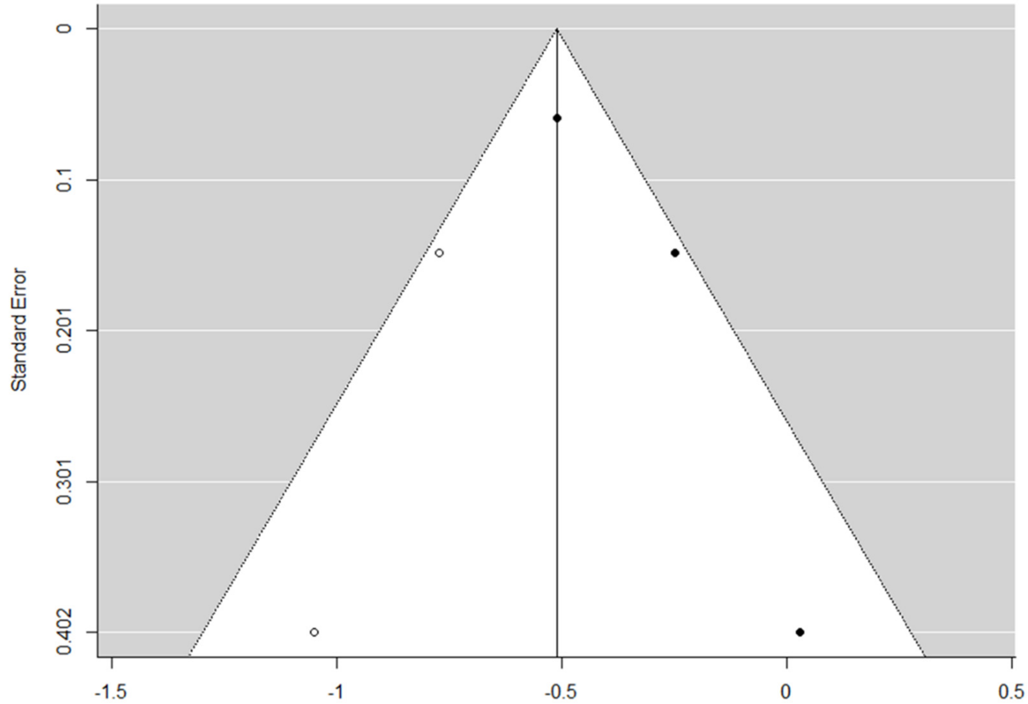

Suppl. Figure S127. Funnel plot comparing low and high socioeconomic status patients (reference) regarding deceased-donor transplantation.

6.13. Preemptive transplantation

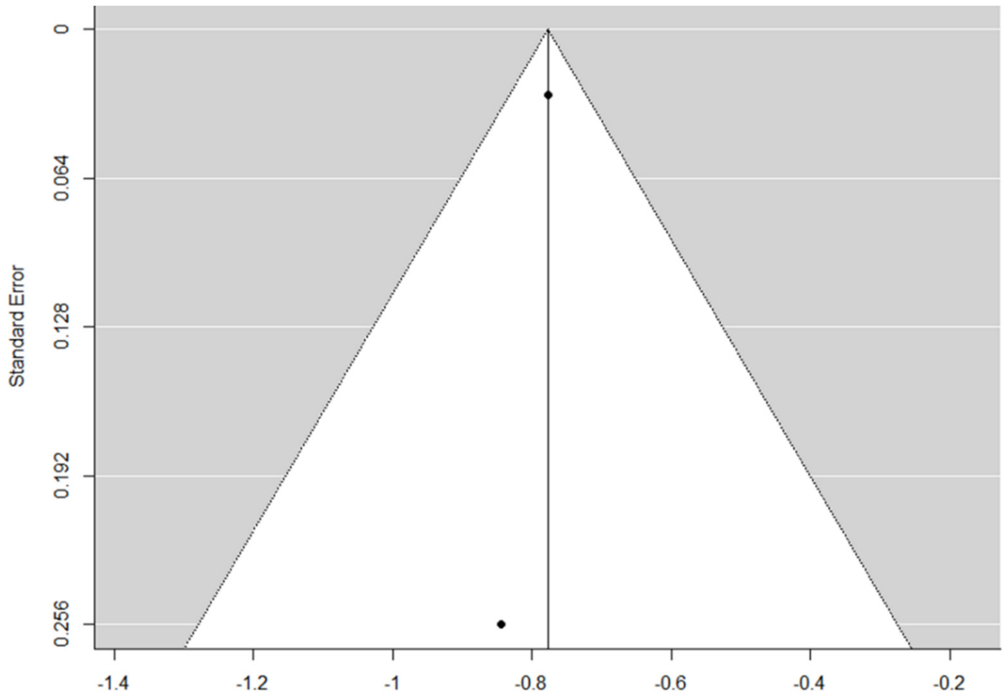

Suppl. Figure S128. Funnel plot comparing Black and White patients (reference) regarding preemptive transplantation.

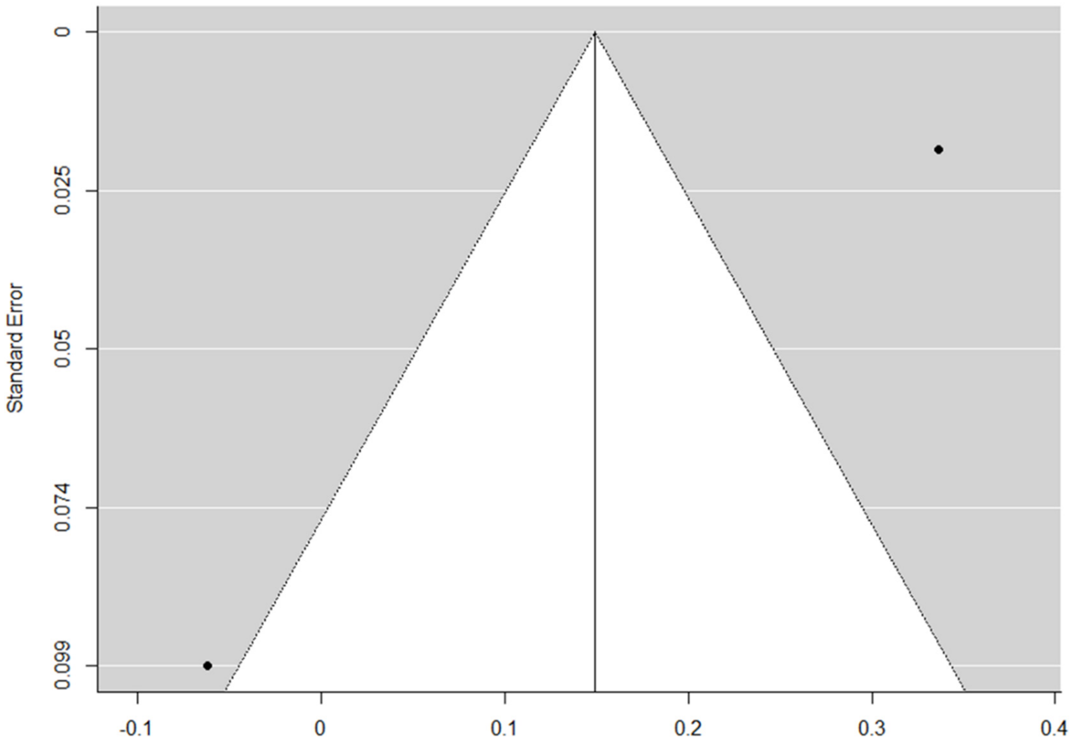

Suppl. Figure S129. Funnel plot of female patients (reference) and preemptive transplantation.

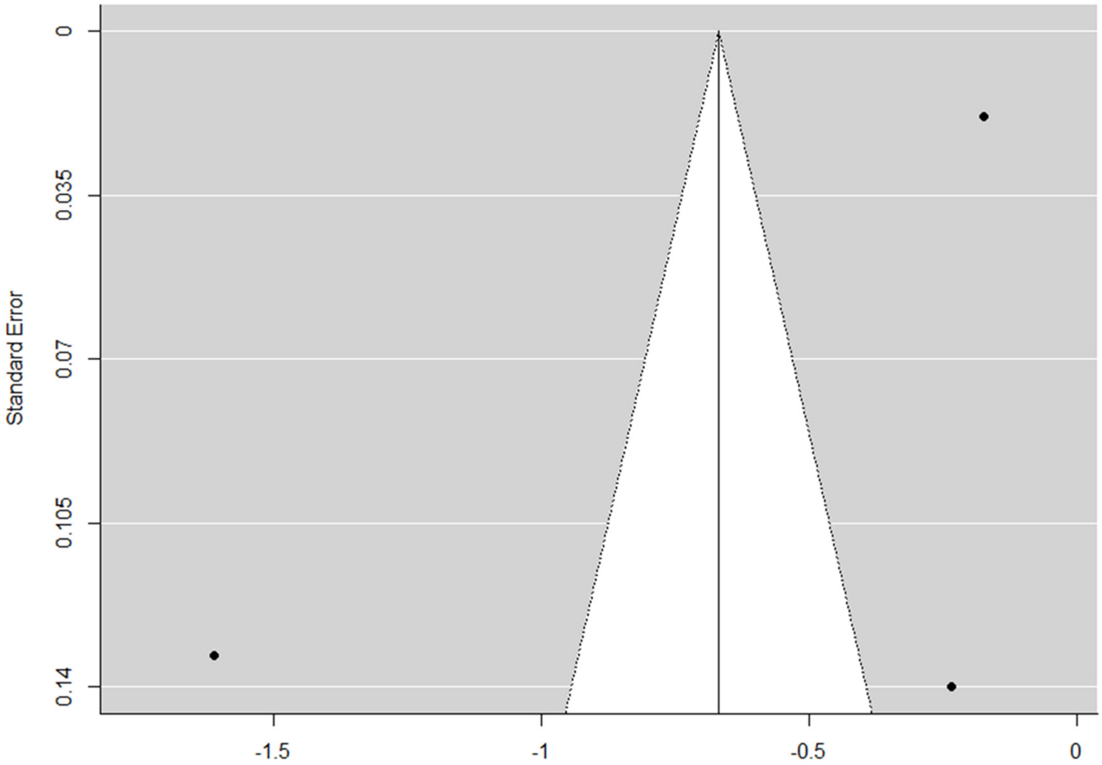

Suppl. Figure S130. Funnel plot comparing low and high socioeconomic status patients (reference) regarding preemptive transplantation.

6.14. Graft survival

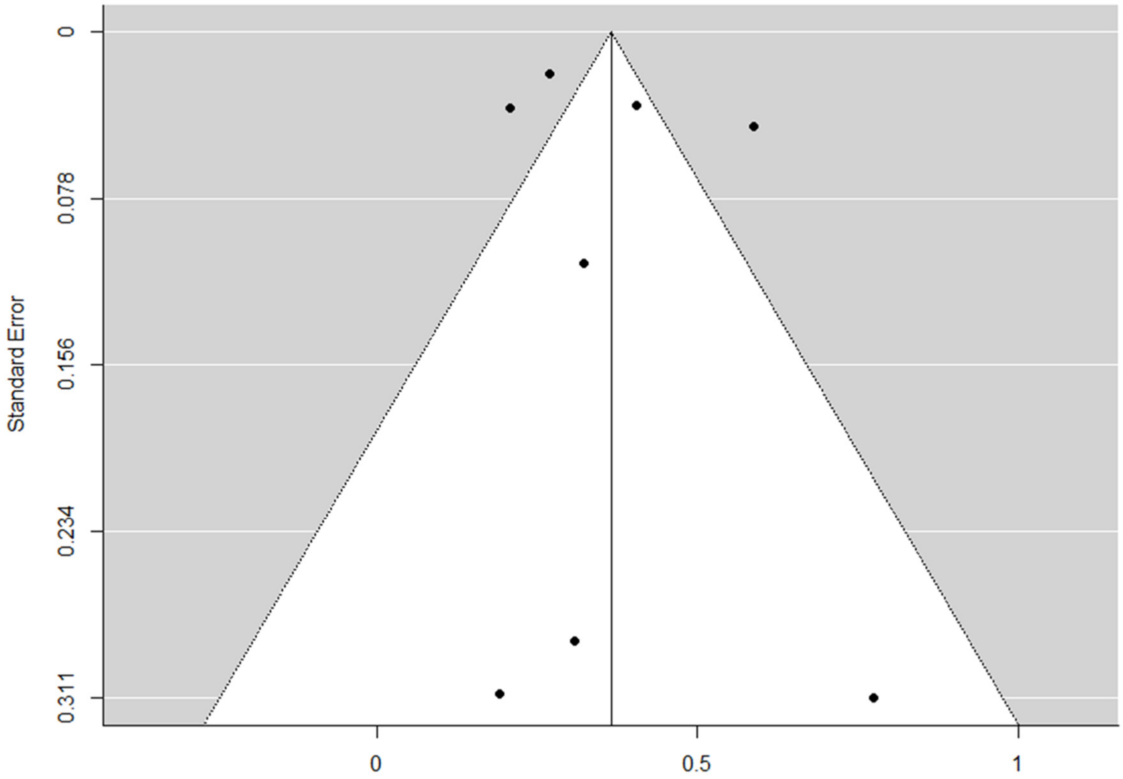

Suppl. Figure S131. Funnel plot comparing Black and White patients (reference) regarding graft survival.

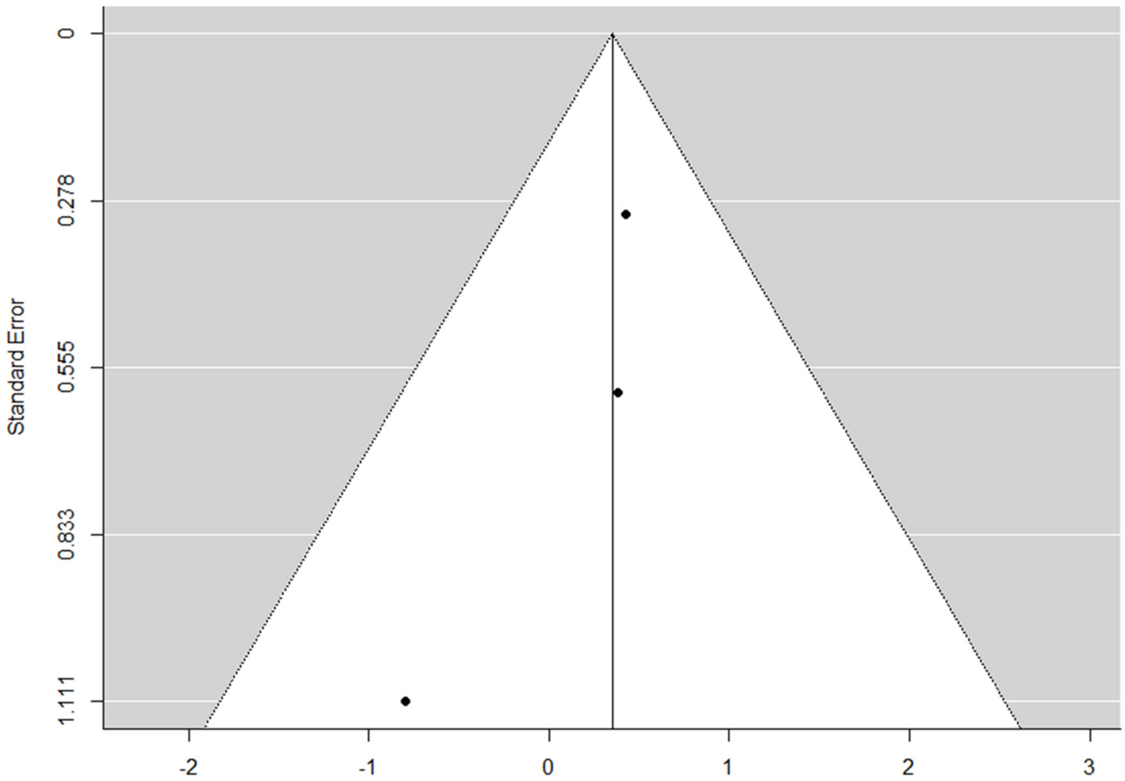

Suppl. Figure S132. Funnel plot comparing Indigenous and White patients (reference) regarding graft survival.

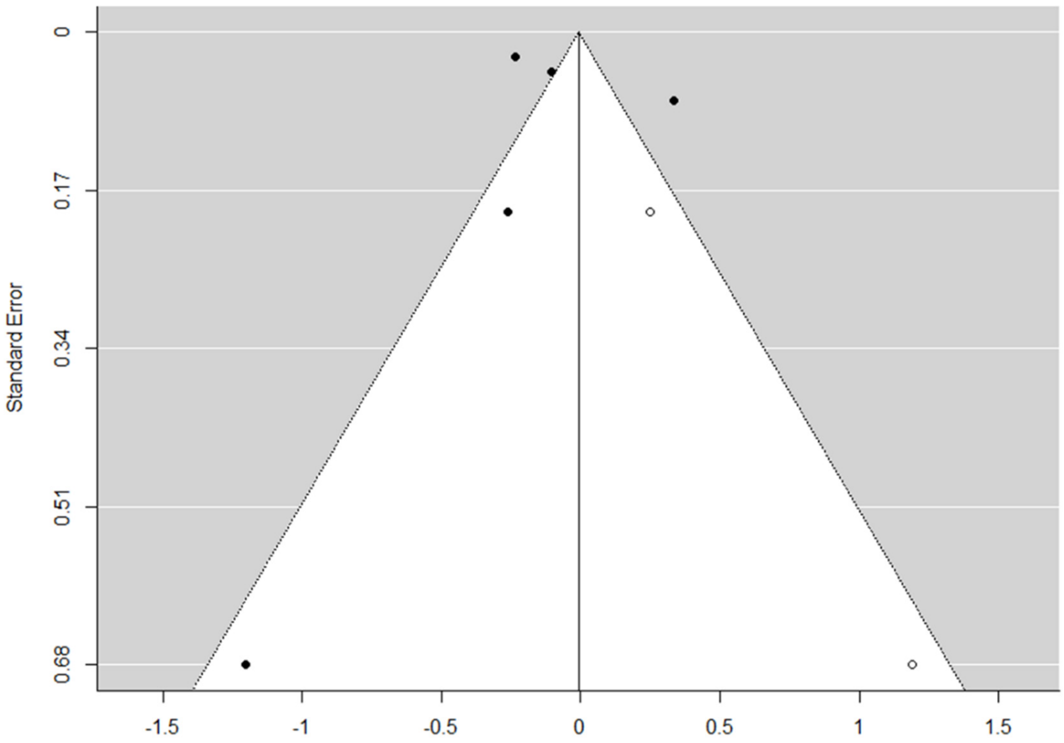

Suppl. Figure S133. Funnel plot comparing Hispanic and White patients (reference) regarding graft survival.

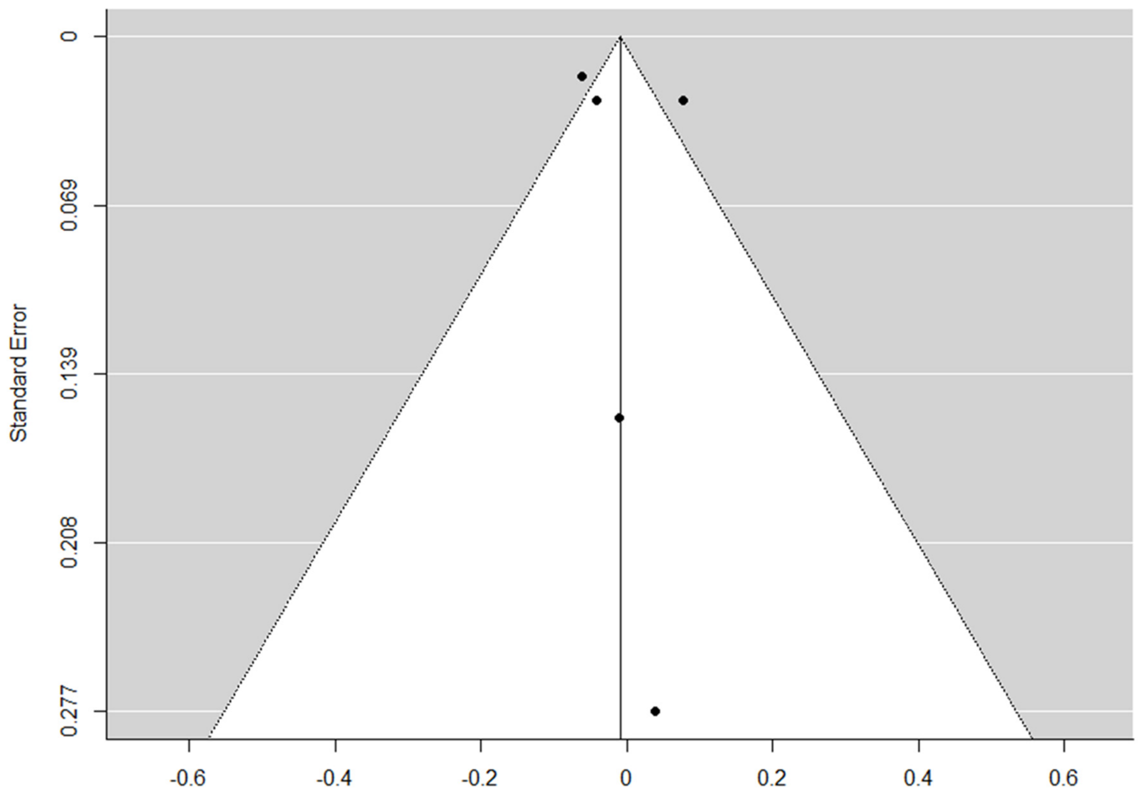

Suppl. Figure S134. Funnel plot of female patients (reference) and graft survival.

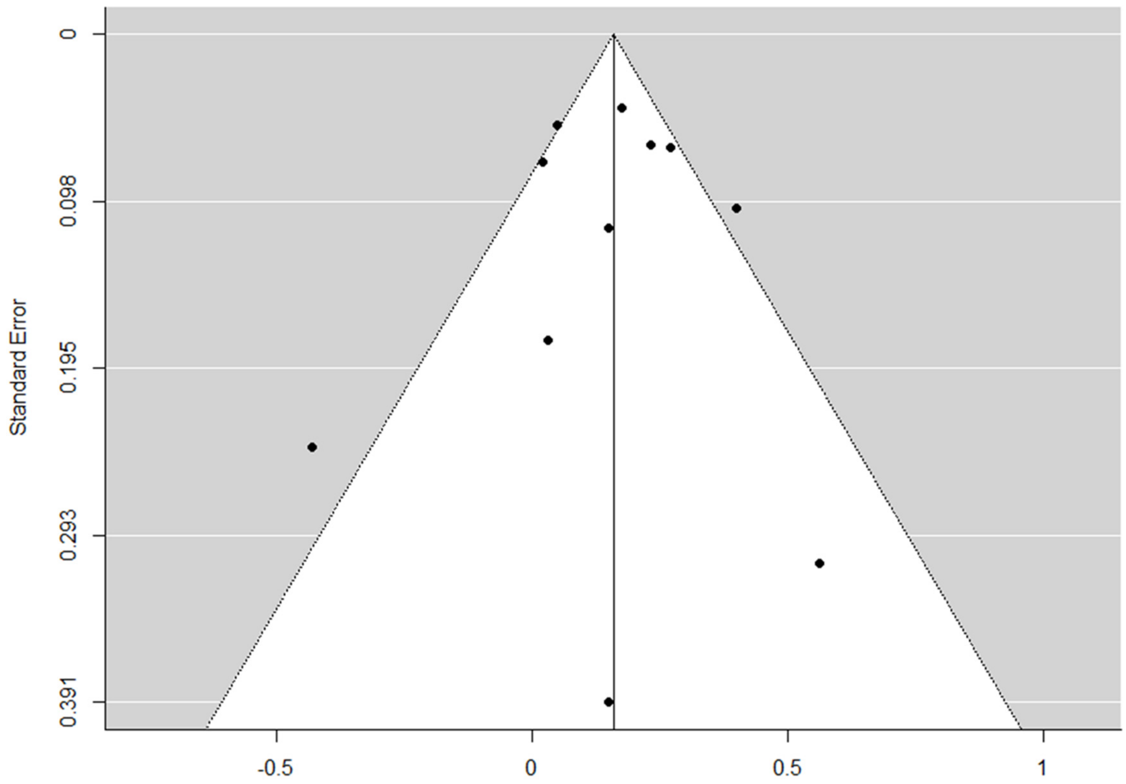

Suppl. Figure S135. Funnel plot comparing low and high socioeconomic status patients (reference) regarding graft survival.

6.15. Kidney transplantation survival

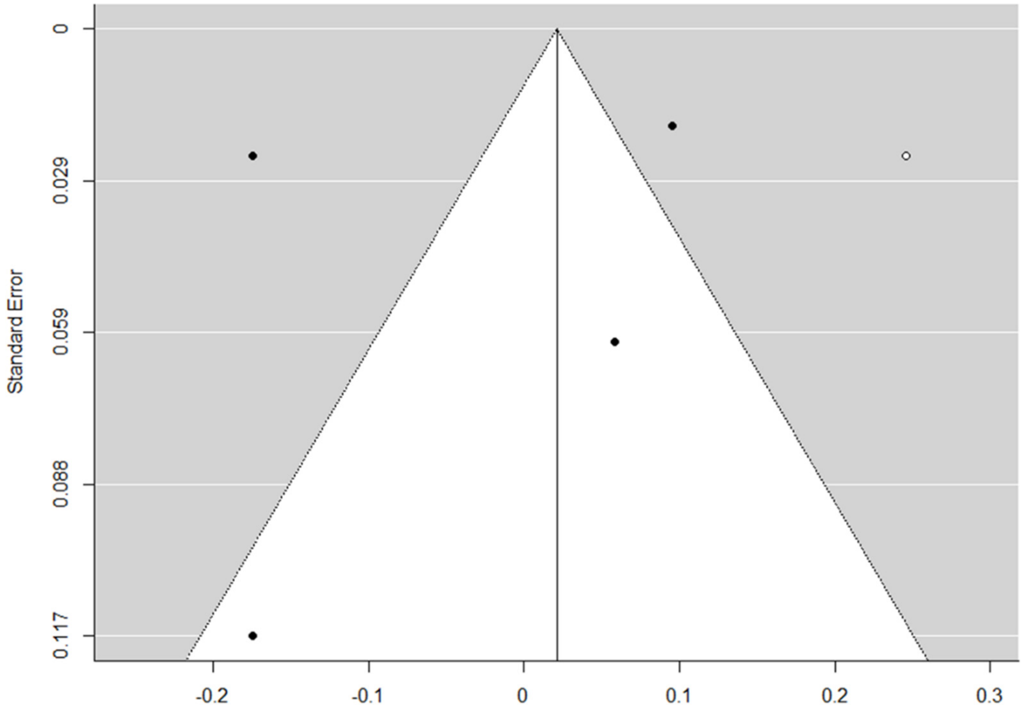

Suppl. Figure S136. Funnel plot comparing Black and White patients (reference) regarding kidney transplantation survival.

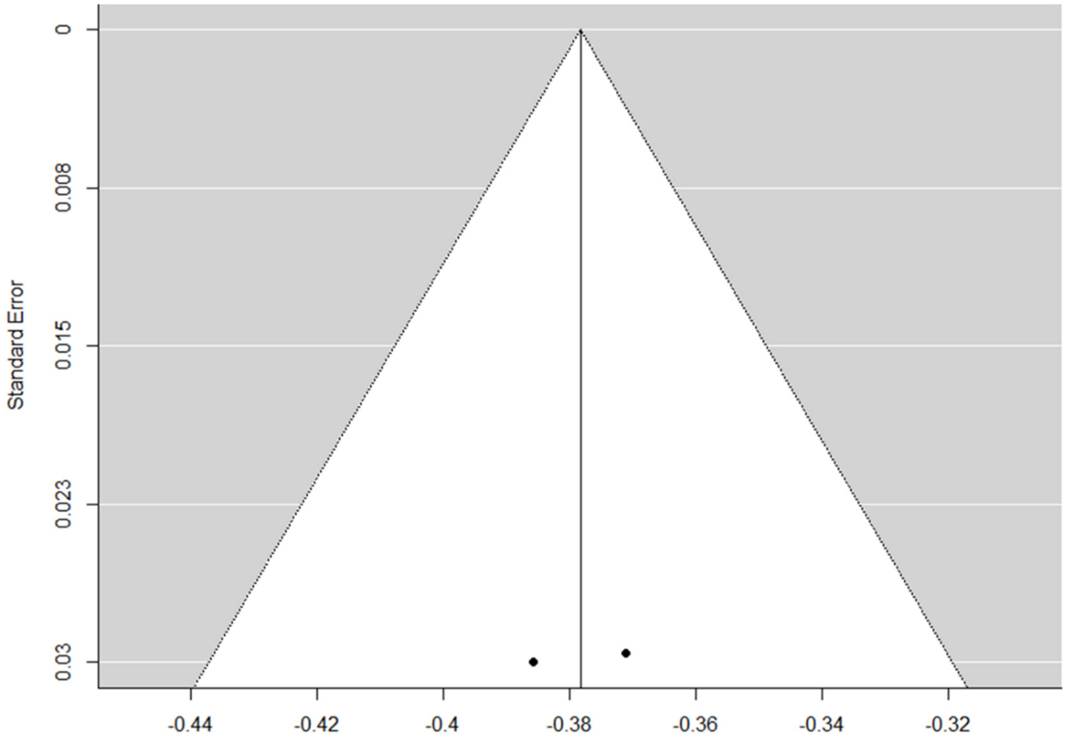

Suppl. Figure S137. Funnel plot comparing Hispanic and White patients (reference) regarding kidney transplantation survival.

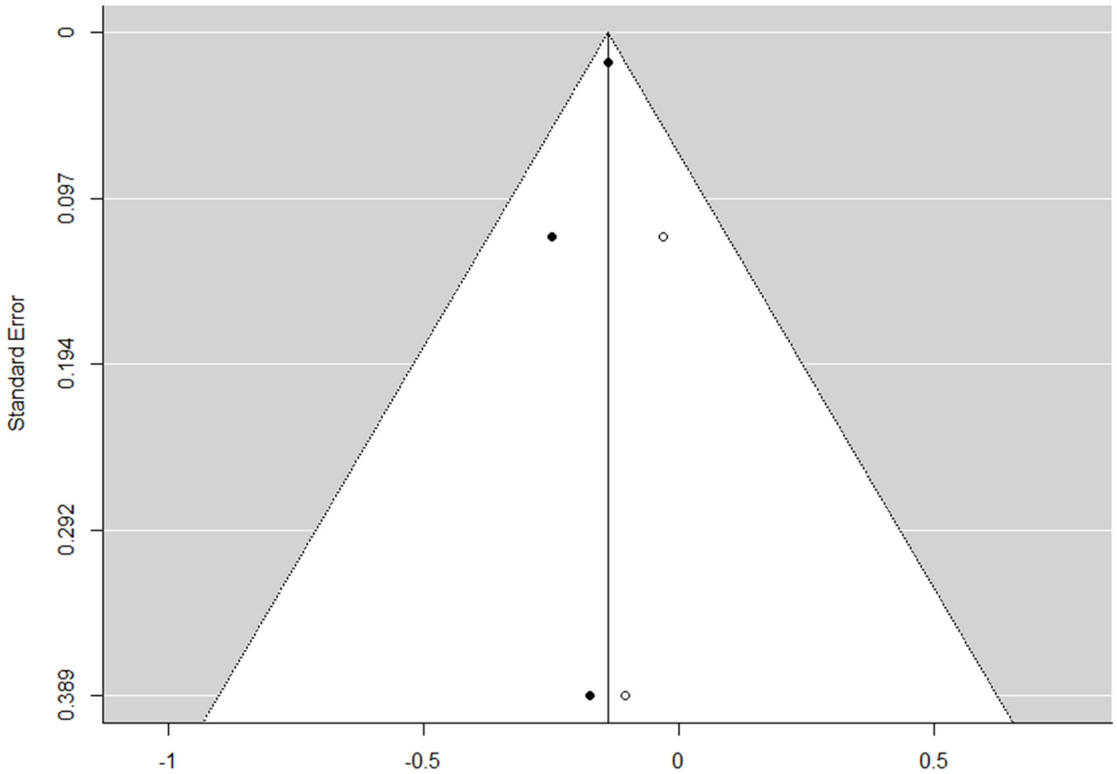

Suppl. Figure S138. Funnel plot of female patients (reference) and kidney transplantation survival.

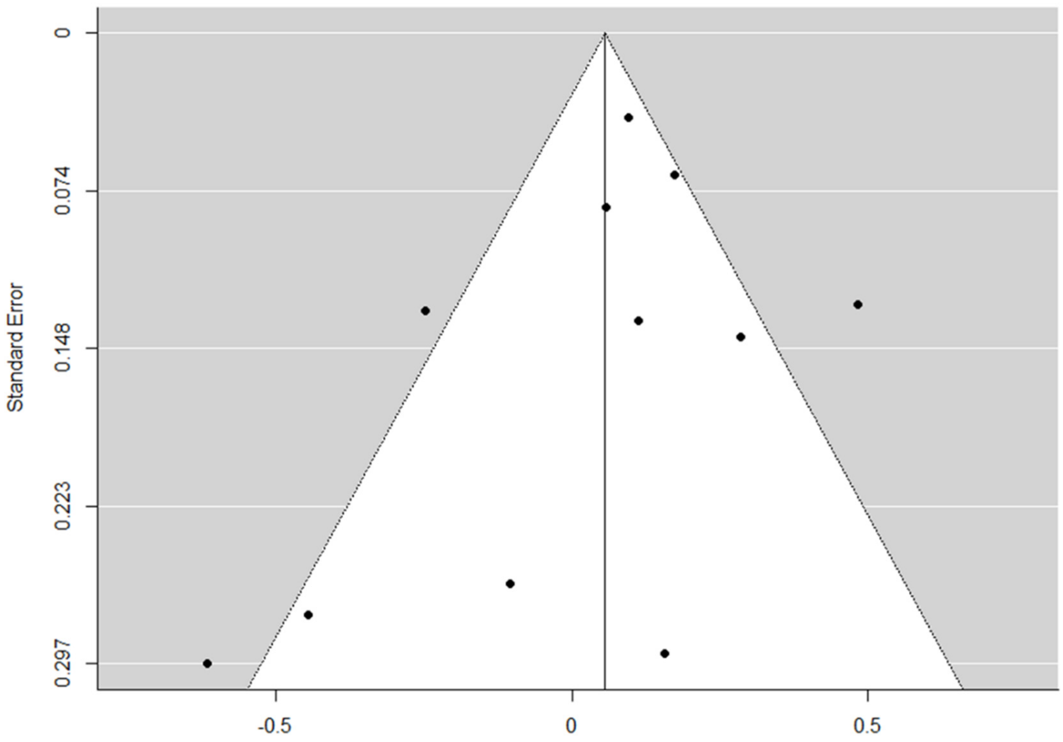

Suppl. Figure S139. Funnel plot comparing low and high socioeconomic status patients (reference) regarding kidney transplantation survival.

Supplementary S7: Subgroup analyses

Suppl. Table S5. Subgroup analysis of the meta-analysis regarding female patients and dialysis initiation with arteriovenous fistula or graft.

Subgroup	Studies no.	OR (95% CI)	*P* for subgroup difference
**Study location**			
North America	2	0.85 (0.84-0.86)	0.611
Oceania	2	0.67 (0.31-1.43)	
**Risk of bias**			
Low	4	0.78 (0.60-1.02)	0.899
Moderate	1	0.81 (0.72-0.91)	

Suppl. Table S6. Subgroup analysis of the meta-analysis regarding low socioeconomic status and dialysis initiation with arteriovenous fistula or graft.

Subgroup	Studies no.	OR (95% CI)	*P* for subgroup difference
**Study location**			
North America	4	0.84 (0.69-1.03)	0.216
Oceania	1	0.48 (0.22-1.05)	
**Exposure**			
Low income	2	0.89 (0.71-1.12)	
Low education	2	0.56 (0.41-0.77)	0.063
Unemployment	1	0.95 (0.84-1.08)	

Suppl. Table S7. Subgroup analysis of the meta-analysis regarding female patients and primary arteriovenous fistula patency.

Subgroup	Studies no.	HR (95% CI)	*P* for subgroup difference
**Study location**			
North America	2	0.79 (0.72-0.87)	
Europe	1	0.50 (0.34-0.73)	**0.025**
International	1	1.19 (0.68-2.10)	
**Risk of bias**			
Low	3	0.80 (0.73-0.88)	**0.020**
Moderate	1	0.50 (0.34-0.73)	

Suppl. Table S8. Subgroup analysis of the meta-analysis regarding low socioeconomic status and primary arteriovenous fistula patency.

Subgroup	Studies no.	OR (95% CI)	*P* for subgroup difference
**Study location**			
Oceania	1	079 (0.66-0.94)	N/A
Europe	1	0.87 (0.48-1.56)	
Asia	1	0.39 (0.18-0.84)	
**Risk of bias**			
Low	1	0.79 (0.66-0.94)	0.663
Moderate	2	0.61 (0.28-1.33)	
**Exposure**			
Low income	2	0.80 (0.67-0.94)	0.076
Low education	1	0.39 (0.18-0.84)	

Suppl. Table S9. Subgroup analysis of the meta-analysis regarding Black patients and peritoneal dialysis use.

Subgroup	Studies no.	OR (95% CI)	*P* for subgroup difference
**Study location**			
USA	4	0.58 (0.46-0.73)	**0.019**
Canada	1	1.04 (0.94-1.15)	
**Risk of bias**			
Low	2	0.87 (0.63-1.22)	
Moderate	2	0.49 (0.40-0.60)	**0.026**
Serious	1	0.58 (0.42-0.80)	

Suppl. Table S10. Subgroup analysis of the meta-analysis regarding female patients and peritoneal dialysis use.

Subgroup	Studies no.	OR (95% CI)	*P* for subgroup difference
**Study location**			
North America	4	1.06 (0.97-1.17)	
Europe	1	0.69 (0.50-0.96)	**0.002**
Asia	1	2.13 (0.77-5.89)	
Oceania	1	1.33 (1.25-1.41)	
**Risk of bias**			
Low	2	1.04 (0.88-1.23)	
Moderate	4	1.08 (0.76-1.53)	0.925
Serious	1	1.17 (0.90-1.53)	

Suppl. Table S11. Subgroup analysis of the meta-analysis regarding low socioeconomic status and peritoneal dialysis use.

**Subgroup**	**Studies no.**	**OR (95% CI)**	***P* for subgroup difference**
**Study location**			
North America	2	0.47 (0.40-0.55)	**<0.001**
Europe	1	0.59 (0.45-0.78)	
Asia	1	0.48 (0.14-1.63)	
Oceania	1	0.73 (0.66-0.81)	
**Risk of bias**			
Moderate	4	0.59 (0.46-0.76)	0.280
Serious	1	0.44 (0.33-0.58)	
**Exposure**			
Low income	4	0.49 (0.43-0.57)	**<0.001**
Low education	1	0.73 (0.66-0.81)	

Suppl. Table S12. Subgroup analysis of the meta-analysis regarding Black patients and dialysis mortality.

**Subgroup**	**Studies no.**	**HR (95% CI)**	***P* for subgroup difference**
**Study location**			
North America	11	0.74 (0.69-0.80)	**<0.001**
South America	1	0.69 (0.50-0.96)	
Europe	6	0.50 (0.44-0.56)	
**Population**			
Hemodialysis	6	0.72 (0.71-0.73)	0.932
Peritoneal dialysis	1	0.69 (0.50-0.96)	
Mixed	11	0.66 (0.57-0.76)	
**Risk of bias**			
Low	12	0.68 (0.60-0.77)	0.815
Moderate	6	0.65 (0.56-0.76)	

Suppl. Table S13. Subgroup analysis of the meta-analysis regarding Asian patients and dialysis mortality.

Subgroup	Studies no.	HR (95% CI)	*P* for subgroup difference
**Study location**			
North America	5	0.68 (0.59-0.79)	0.381
Europe	4	0.68 (0.61-0.76)	
Oceania	3	0.60 (0.49-0.73)	
Asia	1	0.48 (0.25-0.92)	
**Population**			
Hemodialysis	2	0.62 (0.51-0.77)	0.541
Peritoneal dialysis	4	0.61 (0.45-0.84)	
Mixed	8	0.69 (0.63-0.76)	
**Risk of bias**			
Low	10	0.67 (0.61-0.74)	0.628
Moderate	3	0.66 (0.50-0.89)	
Serious	1	0.48 (0.25-0.92)	

Suppl. Table S14. Subgroup analysis of the meta-analysis regarding Indigenous patients and dialysis mortality.

Subgroup	Studies no.	HR (95% CI)	*P* for subgroup difference
**Study location**			
North America	5	0.99 (0.76-1.27)	0.725
Oceania	6	1.05 (0.79-1.41)	
**Population**			
Hemodialysis	2	0.69 (0.67-0.71)	**<0.001**
Peritoneal dialysis	2	0.72 (0.55-0.93)	
Mixed	7	1.22 (1.07-1.40)	
**Risk of bias**			
Low	9	0.99 (0.79-1.24)	0.533
Moderate	2	1.15 (0.87-0.53)	

Suppl. Table S15. Subgroup analysis of the meta-analysis regarding Hispanic patients and dialysis mortality.

Subgroup	Studies no.	HR (95% CI)	*P* for subgroup difference
**Study location**			
North America	7	0.81 (0.73-0.89)	0.495
South America	1	0.71 (0.54-0.94)	
**Population**			
Hemodialysis	4	0.78 (0.70-0.86)	0.676
Peritoneal dialysis	1	0.71 (0.54-0.94)	
Mixed	3	0.83 (0.70-0.99)	
**Risk of bias**			
Low	8	0.81 (0.73-0.89)	0.495
Moderate	1	0.71 (0.54-0.94)	

Suppl. Table S16. Subgroup analysis of the meta-analysis regarding female patients and dialysis mortality.

Subgroup	Studies no.	HR (95% CI)	*P* for subgroup difference
**Study location**			
North America	2	0.98 (0.95-1.01)	0.663
South America	1	0.97 (0.90-1.05)	
Europe	10	0.96 (0.93-1.00)	
Asia	8	1.02 (0.87-1.21)	
Oceania	4	1.05 (0.95-1.15)	
Africa	1	1.12 (0.75-1.68)	
**Population**			
Hemodialysis	3	0.88 (0.73-1.05)	0.203
Peritoneal dialysis	11	1.00 (0.91-1.09)	
Mixed	12	1.02 (0.96-1.08)	
**Risk of bias**			
Low	14	0.98 (0.92-1.04)	0.815
Moderate	6	0.98 (0.96-1.00)	
Serious	6	0.98 (0.80-1.20)	

Suppl. Table S17. Subgroup analysis of the meta-analysis regarding low socioeconomic status and dialysis mortality.

Subgroup	Studies no.	HR (95% CI)	*P* for subgroup difference
**Study location**			
North America	4	1.10 (1.06-1.15)	
South America	4	1.28 (1.14-1.43)	
Europe	4	1.20 (1.10-1.31)	**<0.001**
Asia	8	1.62 (1.23-2.14)	
Oceania	3	1.12 (1.06-1.19)	
**Population**			
Hemodialysis	10	1.16 (1.09-1.25)	
Peritoneal dialysis	8	1.41 (1.11-1.79)	0.207
Mixed	5	1.12 (1.06-1.19)	
**Risk of bias**			
Low	18	1.16 (1.11-1.22)	**<0.001**
Moderate	5	2.12 (1.58-2.85)	

Suppl. Table S18. Subgroup analysis of the meta-analysis regarding Black patients and waitlisting.

Subgroup	Studies no.	OR (95% CI)	*P* for subgroup difference
**Study location**			
North America	5	0.78 (0.68-0.90)	0.426
Europe	2	0.85 (0.46-1.56)	
**Risk of bias**			
Low	5	0.84 (0.77-0.92)	0.242
Moderate	2	0.70 (0.47-1.04)	

Suppl. Table S19. Subgroup analysis of the meta-analysis regarding Indigenous patients and waitlisting.

Subgroup	Studies no.	OR (95% CI)	*P* for subgroup difference
**Study location**			
North America	2	0.77 (0.61-0.99)	**<0.001**
Oceania	1	0.46 (0.38-0.55)	
**Risk of bias**			
Low	2	0.56 (0.36-0.87)	0.366
Moderate	1	0.80 (0.59-1.08)	

Suppl. Table S20. Subgroup analysis of the meta-analysis regarding female patients and waitlisting.

**Subgroup**	**Studies no.**	**OR (95% CI)**	***P* for subgroup difference**
**Study location**			
North America	4	0.89 (0.88-0.90)	
Europe	9	0.89 (0.84-0.94)	0.560
Oceania	1	0.85 (0.80-0.91)	
**Risk of bias**			
Low	10	0.88 (0.86-0.90)	0.584
Moderate	4	0.89 (0.83-0.96)	

Suppl. Table S21. Subgroup analysis of the meta-analysis regarding low socioeconomic status and waitlisting.

**Subgroup**	**Studies no.**	**OR (95% CI)**	***P* for subgroup difference**
**Study location**			
North America	5	0.55 (0.44-0.72)	0.587
Europe	6	0.60 (0.54-0.65)	
**Risk of bias**			
Low	7	0.55 (0.44-0.66)	0.289
Moderate	4	0.64 (0.55-0.74)	

Suppl. Table S22. Subgroup analysis of the meta-analysis regarding Indigenous patients and kidney transplantation.

**Subgroup**	**Studies no.**	**OR (95% CI)**	***P* for subgroup difference**
**Study location**			
North America	4	0.48 (0.36-0.63)	**0.001**
Oceania	3	0.25 (0.21-0.31)	
**Risk of bias**			
Low	6	0.38 (0.27-0.53)	0.733
Moderate	1	0.32 (0.25-0.40)	

Suppl. Table S23. Subgroup analysis of the meta-analysis regarding female patients and kidney transplantation.

Subgroup	Studies no.	OR (95% CI)	*P* for subgroup difference
**Study location**			
North America	3	0.90 (0.81-0.99)	0.574
Europe	4	0.94 (0.84-1.05)	
**Risk of bias**			
Low	5	0.90 (0.82-0.97)	0.241
Moderate	2	1.00 (0.87-1.14)	

Suppl. Table S24. Subgroup analysis of the meta-analysis regarding low socioeconomic status and kidney transplantation.

Subgroup	Studies no.	OR (95% CI)	*P* for subgroup difference
**Study location**			
North America	3	0.63 (0.56-0.69)	**0.015**
Europe	2	0.81 (0.68-0.95)	
Asia	1	0.40 (0.24-0.67)	
Oceania	1	0.94 (0.54-1.64)	
South America	1	0.59 (0.20-1.73)	
**Risk of bias**			
Low	6	0.63 (0.56-0.72)	**0.012**
Moderate	2	0.89 (0.75-1.06)	

Suppl. Table S25. Subgroup analysis of the meta-analysis regarding Black patients and living-donor transplantation.

Subgroup	Studies no.	OR (95% CI)	*P* for subgroup difference
**Study location**			
North America	6	0.41 (0.30-0.56)	0.964
Europe	1	0.40 (0.21-0.75)	
**Risk of bias**			
Low	5	0.35 (0.29-0.42)	**0.015**
Moderate	2	0.59 (0.29-1.20)	

Suppl. Table S26. Subgroup analysis of the meta-analysis regarding Asian patients and living-donor transplantation.

**Subgroup**	**Studies no.**	**OR (95% CI)**	***P* for subgroup difference**
**Study location**			
North America	3	0.36 (0.24-0.55)	0.161
Europe	1	0.66 (0.45-0.96)	
**Risk of bias**			
Low	3	0.36 (0.24-0.55)	0.161
Moderate	1	0.66 (0.45-0.96)	

Suppl. Table S27. Subgroup analysis of the meta-analysis regarding Indigenous patients and living-donor transplantation.

**Subgroup**	**Studies no.**	**OR (95% CI)**	***P* for subgroup difference**
**Study location**			
North America	4	0.41 (0.37-0.44)	**<0.001**
Europe	3	0.19 (0.13-0.28)	

Suppl. Table S28. Subgroup analysis of the meta-analysis regarding female patients and living-donor transplantation.

**Subgroup**	**Studies no.**	**OR (95% CI)**	***P* for subgroup difference**
**Study location**			
North America	1	1.06 (0.74-1.52)	
Europe	1	0.94 (0.74-1.20)	N/A
Oceania	1	0.84 (0.75-0.95)	
**Risk of bias**			
Low	2	0.88 (0.73-1.07)	0.738
Moderate	1	0.94 (0.74-1.20)	

Suppl. Table S29. Subgroup analysis of the meta-analysis regarding low socioeconomic status and living-donor transplantation.

**Subgroup**	**Studies no.**	**OR (95% CI)**	***P* for subgroup difference**
**Study location**			
North America	1	0.59 (0.38-0.92)	
Europe	2	0.42 (0.36-0.49)	0.229
Oceania	1	0.79 (0.25-2.50)	
**Risk of bias**			
Low	1	0.59 (0.38-0.92)	0.176
Moderate	3	0.43 (0.37-0.50)	
Exposure			
Low income	3	0.48 (0.34-0.69)	0.873
Low education	1	0.46 (0.36-0.59)	

Suppl. Table S30. Subgroup analysis of the meta-analysis regarding Black patients and deceased-donor transplantation.

Subgroup	Studies no.	OR (95% CI)	*P* for subgroup difference
**Study location**			
North America	5	0.74 (0.45-1.22)	0.684
Europe	1	0.95 (0.80-1.13)	
**Risk of bias**			
Low	4	0.85 (0.49-1.46)	0.550
Moderate	2	0.63 (0.28-1.44)	

Suppl. Table S31. Subgroup analysis of the meta-analysis regarding Asian patients and deceased-donor transplantation.

Subgroup	Studies no.	OR (95% CI)	*P* for subgroup difference
**Study location**			
North America	2	0.79 (0.54-1.16)	0.302
Europe	1	1.12 (1.00-1.25)	
**Risk of bias**			
Low	2	0.79 (0.54-1.16)	0.302
Moderate	1	1.12 (1.00-1.25)	

Suppl. Table S32. Subgroup analysis of the meta-analysis regarding Indigenous patients and deceased-donor transplantation.

Subgroup	Studies no.	OR (95% CI)	*P* for subgroup difference
**Study location**			
North America	4	0.47 (0.36-0.61)	**0.019**
Oceania	2	0.27 (0.19-0.39)	

Suppl. Table S33. Subgroup analysis of the meta-analysis regarding female patients and deceased-donor transplantation.

Subgroup	Studies no.	OR (95% CI)	*P* for subgroup difference
**Study location**			
North America	1	0.78 (0.58-1.04)	
Europe	1	0.60 (0.53-0.67)	N/A
Oceania	1	1.03 (0.47-2.26)	
**Risk of bias**			
Low	1	0.78 (0.58-1.04)	0.709
Moderate	2	0.68 (0.44-1.06)	

Suppl. Table S34. Subgroup analysis of the meta-analysis regarding low socioeconomic status and preemptive transplantation.

**Subgroup**	**Studies no.**	**OR (95% CI)**	***P* for subgroup difference**
**Study location**			
North America	1	0.84 (0.81-0.87)	0.526
Europe	2	0.40 (0.10-1.53)	

Suppl. Table S35. Subgroup analysis of the meta-analysis regarding Black patients and graft failure.

**Subgroup**	**Studies no.**	**HR (95% CI)**	***P* for subgroup difference**
**Study location**			
North America	6	1.43 (1.26-1.62)	0.619
Europe	2	1.62 (0.91-2.87)	
**Risk of bias**			
Low	5	1.35 (1.23-1.47)	**0.004**
Moderate	3	1.79 (1.65-1.95)	

Suppl. Table S36. Subgroup analysis of the meta-analysis regarding Indigenous patients and graft failure.

**Subgroup**	**Studies no.**	**HR (95% CI)**	***P* for subgroup difference**
**Study location**			
North America	2	1.13 (0.40-3.16)	0.615
Oceania	1	1.53 (0.85-2.76)	

Suppl. Table S37. Subgroup analysis of the meta-analysis regarding Hispanic patients and graft failure.

**Subgroup**	**Studies no.**	**HR (95% CI)**	***P* for subgroup difference**
**Risk of bias**			
Low	3	0.83 (0.73-0.95)	0.248
Moderate	2	1.07 (0.59-1.91)	

Suppl. Table S38. Subgroup analysis of the meta-analysis regarding female patients and graft failure.

Subgroup	Studies no.	HR (95% CI)	*P* for subgroup difference
**Study location**			
North America	4	0.99 (0.91-1.08)	0.995
Europe	1	0.99 (0.73-1.35)	
**Risk of bias**			
Low	3	1.02 (0.92-1.13)	0.361
Moderate	2	0.94 (0.91-0.97)	

Suppl. Table S39. Subgroup analysis of the meta-analysis regarding low socioeconomic status and graft failure.

Subgroup	Studies no.	HR (95% CI)	*P* for subgroup difference
**Study location**			
North America	7	1.15 (0.96-1.37)	
Europe	2	1.06 (0.94-1.20)	0.389
Asia	2	1.28 (1.17-1.41)	
**Risk of bias**			
Low	6	1.18 (1.07-1.31)	
Moderate	4	1.05 (0.86-1.28)	0.128
Serious	1	1.49 (1.22-1.82)	
Exposure			
Low income	5	1.08 (0.91-1.28)	
Low education	4	1.14 (1.01-1.28)	0.220
Unemployment	2	1.34 (1.15-1.58)	

Suppl. Table S40. Subgroup analysis of the meta-analysis regarding Black patients and kidney transplantation mortality.

Subgroup	Studies no.	HR (95% CI)	*P* for subgroup difference
**Risk of bias**			
Low	3	0.93 (0.77-1.12)	0.499
Moderate	1	1.06 (0.94-1.19)	

Suppl. Table S41. Subgroup analysis of the meta-analysis regarding female patients and kidney transplantation mortality.

Subgroup	Studies no.	HR (95% CI)	*P* for subgroup difference
**Study location**			
North America	2	0.87 (0.84-0.90)	0.366
Europe	1	0.78 (0.62-0.99)	

Suppl. Table S42. Subgroup analysis of the meta-analysis regarding low socioeconomic status and kidney transplantation mortality.

Subgroup	Studies no.	HR (95% CI)	*P* for subgroup difference
**Study location**			
North America	6	1.12 (1.05-1.19)	0.090
Europe	2	0.92 (0.69-1.25)	
Asia	2	1.35 (0.94-1.94)	
Oceania	1	0.64 (0.37-1.09	
**Risk of bias**			
Low	7	1.11 (0.94-1.31)	0.405
Moderate	3	0.90 (0.60-1.36)	
Exposure			
Low income	7	1.01 (0.78-1.32)	0.961
Low education	3	1.10 (1.02-1.18)	
Unemployment	1	1.12 (0.86-1.46)	

Supplementary S8: Sensitivity analysis

Suppl. Table S43. Meta-analysis outcomes of studies conducted in the USA.

Outcome	Black race	Asian race	Indigenous people	Hispanic ethnicity	Female sex	Low SES
AVF/AVG vs. CVC	**1.08 (1.07-1.10)**	**1.11 (1.08-1.15)**	1.04 (0.98-1.10)	-	**0.85 (0.84-0.86)**	0.84 (0.69-1.03)
AVF vs. AVG	**0.58 (0.51-0.66)**	-	**1.30 (1.12-1.51)**	-	**0.43 (0.38-0.47)**	-
Successful AVF use	**0.88 (0.83-0.93)**	-	-	-	**0.82 (0.76-0.88)**	-
Primary AVF patency	**0.87 (0.86-0.88)**	-	-	-	**0.79 (0.72-0.87)**	-
Transition to AVF/AVG	1.06 (0.92-1.22)	-	-	-	**0.92 (0.85-1.00)**	**0.80 (0.73-0.88)**
Home dialysis	0.91 (0.74-1.12)	**0.64 (0.41-1.00)**	-	-	-	-
Peritoneal dialysis	**0.58 (0.46-0.73)**	1.02 (0.98-1.07)			1.06 (0.97-1.17)	**0.47 (0.40-0.55)**
Dialysis survival	**0.74 (0.68-0.81)**	**0.62 (0.51-0.77)**	**0.69 (0.67-0.71)**	**0.81 (0.73-0.89)**	0.98 (0.96-1.01)	**1.11 (1.06-1.17)**
Waitlisting	**0.78 (0.68-0.90)**	**1.20 (1.18-1.22)**	**0.77 (0.61-0.99)**	1.09 (0.87-1.37)	**0.89 (0.88-0.90)**	**0.55 (0.44-0.72)**
Kidney transplantation	0.74 (0.48-1.14)	**0.49 (0.41-0.59)**	**0.44 (0.42-0.47)**	1.10 (0.51-2.37)	0.95 (0.81-1.11)	**0.65 (0.64-0.66)**
Living-donor transplantation	**0.48 (0.29-0.79)**	**0.49 (0.43-0.55)**	**0.40 (0.36-0.44)**	1.22 (0.57-5.60)	1.06 (0.74-1.52)	**0.59 (0.38-0.92)**
Deceased-donor transplantation	0.77 (0.40-1.49)	**0.95 (0.91-0.99)**	**0.52 (0.46-0.60)**	1.23 (0.40-3.75)	**0.94 (0.93-0.95)**	0.78 (0.58-1.04)
Preemptive transplantation	**0.46 (0.44-0.49)**	-	-	-	**1.40 (1.35-1.45)**	**0.84 (0.81-0.87)**
Graft survival	**1.43 (1.26-1.62)**	-	1.13 (0.40-3.16)	0.90 (0.68-1.20)	0.99 (0.91-1.08)	1.16 (0.94-1.44)
Kidney transplantation survival	0.96 (0.83-1.12)	-	-	**0.69 (0.66-0.71)**	**0.87 (0.84-0.90)**	**1.11 (1.04-1.18)**

Effect estimates are odds ratios or hazard ratios (95% confidence intervals). Bolt text indicated statistical significance.

SES: socioeconomic status; AVF: arteriovenous fistula; AVG: arteriovenous graft

Supplementary S9: Summary of findings

Suppl. Table S44. Summary of findings and certainty of evidence assessment

				GRADE					
	**Studies**	**ES (95% CI)**	**95% PI**	***Study limitations***	***Directness***	***Consistency***	***Precision***	***Publication bias***	***Certainty of evidence***
**<u>Dialysis</u>**									
**Mortality**									
Black race	18	0.68 (0.61-0.75)	0.61-0.75	Low risk	Yes	No	Yes	Undetected	***Moderate***
Asian race	12	0.67 (0.61-0.72)	0.52-0.85	Moderate risk	Yes	Yes	Yes	Undetected	***Moderate***
Indigenous race	11	1.02 (0.85-1.23)	0.56-1.89	Low risk	Yes	No	No	Undetected	***Low***
Hispanic ethnicity	8	0.80 (0.73-0.88)	0.63-1.02	Low risk	Yes	No	Yes	Undetected	***Moderate***
Female sex	26	0.99 (0.95-1.04)	0.84-1.17	Moderate risk	Yes	No	Yes	Undetected	***Low***
Low SES	23	1.22 (1.14-1.31)	0.95-1.57	Moderate risk	Yes	No	Yes	Suspected	***Very Low***
**Arteriovenous fistula/graft *vs.* Central venous catheter**									
Black race	2	1.08 (1.07-1.10)	1.07-1.10	Low risk	Yes	Yes	Yes	Undetected	***High***
Asian race	2	1.04 (0.76-1.44)	0.65-1.66	Low risk	Yes	Yes	No	Undetected	***Moderate***
Indigenous race	3	0.93 (0.82-1.07)	0.73-1.20	Low risk	Yes	No	Yes	Undetected	***Moderate***
Female sex	5	0.81 (0.69-0.95)	0.57-1.15	Low risk	Yes	No	Yes	Suspected	***Low***
Low SES	5	0.81 (0.65-1.00)	0.52-1.26	Low risk	Yes	No	Yes	Suspected	***Low***
**Arteriovenous fistula *vs.* graft**									
Black race	4	0.58 (0.51-0.66)	0.45-0.75	Low risk	Yes	Yes	Yes	Undetected	***High***
Indigenous race	2	0.93 (0.42-2.09)	0.25-3.44	Low risk	Yes	No	No	Undetected	***Low***
Female sex	5	0.43 (0.39-0.47)	0.36-0.51	Low risk	Yes	Yes	Yes	Undetected	***High***
**Successful arteriovenous fistula use**									
Black race	2	0.89 (0.84-0.94)	0.84-0.94	Low risk	Yes	Yes	Yes	Undetected	***High***
Female sex	3	0.64 (0.40-1.02)	0.26-1.59	Low risk	Yes	No	No	Suspected	***Very Low***

**Primary arteriovenous fistula patency**									
Black race	2	0.87 (0.86-0.88)	0.86-0.88	Low risk	Yes	Yes	Yes	Undetected	***High***
Female sex	4	0.73 (0.56-0.96)	0.72-0.87	Low risk	Yes	No	No	Undetected	***Low***
Low SES	3	0.77 (0.65-0.91)	0.65-0.91	Moderate risk	Yes	Yes	Yes	Undetected	***Moderate***
**Transition to arteriovenous fistula/graft**									
Black race	2	1.06 (0.92-1.22)	0.84-1.34	Low risk	Yes	No	No	Undetected	***Low***
Female sex	2	0.92 (0.85-1.00)	0.80-1.06	Low risk	Yes	No	Yes	Undetected	***Moderate***
Low SES	2	0.80 (0.73-0.88)	0.73-0.88	Moderate risk	Yes	Yes	Yes	Undetected	***Moderate***
**Home dialysis**									
Black race	2	1.00 (0.83-1.21)	0.78-1.28	Low risk	Yes	Yes	Yes	Undetected	***High***
Asian race	2	0.86 (0.52-1.42)	0.39-1.91	Low risk	Yes	No	No	Undetected	***Low***
**Peritoneal dialysis**									
Black race	5	0.65 (0.49-0.88)	0.33-1.30	Moderate risk	Yes	No	Yes	Undetected	***Low***
Asian race	3	1.28 (1.00-1.64)	0.78-2.09	Low risk	Yes	No	No	Undetected	***Low***
Indigenous race	2	0.83 (0.61-1.14)	0.48-1.43	Low risk	Yes	No	No	Undetected	***Low***
Female sex	7	1.07 (0.92-1.25)	0.74-1.55	Moderate risk	Yes	No	Yes	Undetected	***Low***
Low SES	5	0.56 (0.44-0.70)	0.38-0.89	Moderate risk	Yes	No	Yes	Undetected	***Low***
**Kidney transplantation**									
**Waitlisting**									
Black race	7	0.80 (0.70-0.91)	0.59-1.09	Low risk	Yes	No	Yes	Undetected	***Moderate***
Asian race	2	1.25 (1.08-1.44)	1.01-1.55	Low risk	Yes	Yes	No	Undetected	***Moderate***
Indigenous race	3	0.63 (0.44-0.91)	0.32-1.22	Low risk	Yes	No	No	Undetected	***Low***
Hispanic ethnicity	3	1.09 (0.87-1.37)	0.70-1.69	Low risk	Yes	No	No	Undetected	***Low***
Female sex	14	0.89 (0.87-0.90)	0.87-0.91	Low risk	Yes	Yes	Yes	Undetected	***High***
Low SES	11	0.58 (0.51-0.66)	0.41-0.82	Low risk	Yes	No	Yes	Undetected	***Moderate***
**Kidney transplantation**									
Black race	5	0.70 (0.49-0.99)	0.31-1.58	Low risk	Yes	No	No	Undetected	***Low***

Asian race	2	0.59 (0.42-0.82)	0.33-1.02	Low risk	Yes	No	No	Undetected	***Low***
Indigenous race	7	0.37 (0.27-0.50)	0.16-0.85	Low risk	Yes	No	Yes	Undetected	***Moderate***
Hispanic ethnicity	2	1.10 (0.51-2.37)	0.30-3.99	Low risk	Yes	No	No	Undetected	***Very Low***
Female sex	7	0.91 (0.85-0.98)	0.77-1.08	Low risk	Yes	No	Yes	Undetected	***Moderate***
Low SES	8	0.68 (0.58-0.79)	0.47-0.97	Low risk	Yes	No	Yes	Undetected	***Moderate***
**Living-donor transplantation**									
Black race	7	0.40 (0.31-0.50)	0.24-0.68	Low risk	Yes	Yes	Yes	Undetected	***High***
Asian race	4	0.42 (0.28-0.62)	0.18-0.95	Low risk	Yes	No	No	Undetected	***Low***
Indigenous race	7	0.31 (0.22-0.43)	0.13-0.74	Low risk	Yes	No	Yes	Suspected	***Low***
Hispanic ethnicity	3	1.22 (0.27-5.60)	0.10-15.28	Low risk	Yes	No	No	Undetected	***Low***
Female sex	3	0.88 (0.79-0.98)	0.77-0.99	Moderate risk	Yes	Yes	Yes	Undetected	***Moderate***
Low SES	4	0.45 (0.38-0.54)	0.36-0.57	Moderate risk	Yes	Yes	Yes	Undetected	***Moderate***
**Deceased-donor transplantation**									
Black race	6	0.77 (0.51-1.16)	0.27-2.21	Low risk	Yes	No	No	Undetected	***Low***
Asian race	3	0.89 (0.65-1.22)	0.48-1.64	Low risk	Yes	No	No	Undetected	***Low***
Indigenous race	6	0.39 (0.29-0.53)	0.18-0.85	Low risk	Yes	No	No	Undetected	***Low***
Hispanic ethnicity	2	1.23 (0.40-3.75)	0.19-8.19	Low risk	Yes	No	No	Undetected	***Low***
Female sex	2	0.94 (0.93-0.95)	0.93-0.95	Low risk	Yes	Yes	Yes	Undetected	***High***
Low SES	3	0.69 (0.54-0.89)	0.46-1.03	Moderate risk	Yes	No	Yes	Undetected	***Low***
**Preemptive transplantation**									
Black race	2	0.46 (0.44-0.49)	0.44-0.49	Low risk	Yes	Yes	Yes	Undetected	***High***
Female sex	2	1.16 (0.79-1.71)	0.60-2.25	Low risk	Yes	No	No	Undetected	***Low***
Low SES	3	0.51 (0.21-1.28)	0.08-3.15	Low risk	Yes	No	No	Undetected	***Low***
**Graft failure**									
Black race	8	1.44 (1.27-1.63)	1.07-1.94	Low risk	Yes	No	Yes	Undetected	***Moderate***
Indigenous race	3	1.42 (0.85-2.37)	0.85-2.37	Low risk	Yes	Yes	No	Undetected	***Moderate***
Hispanic ethnicity	5	0.90 (0.68-1.20)	0.49-1.68	Low risk	Yes	No	No	Undetected	***Low***

Female sex	5	0.99 (0.92-1.07)	0.85-1.15	Low risk	Yes	No	Yes	Undetected	***Moderate***
Low SES	11	1.17 (1.07-1.28)	0.93-1.47	Low risk	Yes	No	Yes	Undetected	***Moderate***
**Mortality**									
Black race	4	0.96 (0.83-1.12)	0.71-1.31	Low risk	Yes	No	Yes	Undetected	***Moderate***
Hispanic ethnicity	2	0.69 (0.66-0.71)	0.66-0.71	Low risk	Yes	Yes	Yes	Undetected	***High***
Female sex	3	0.87 (0.84-0.90)	0.84-0.90	Low risk	Yes	Yes	Yes	Undetected	***High***
Low SES	11	1.06 (0.91-1.23)	0.69-1.63	Low risk	Yes	No	Yes	Suspected	***Low***

ES: effect size; PI: prediction intervals; GRADE: Grading of Recommendations, Assessment, Development and Evaluations; SES: socioeconomic status

Supplementary S10: PRISMA checklist

1Section and Topic	Item #	Checklist item	Location where item is reported
**TITLE**			
Title	1	Identify the report as a systematic review.	1
**ABSTRACT**			
Abstract	2	See the PRISMA 2020 for Abstracts checklist.	1
**INTRODUCTION**			
Rationale	3	Describe the rationale for the review in the context of existing knowledge.	2
Objectives	4	Provide an explicit statement of the objective(s) or question(s) the review addresses.	2
**METHODS**			
Eligibility criteria	5	Specify the inclusion and exclusion criteria for the review and how studies were grouped for the syntheses.	2, 3
Information sources	6	Specify all databases, registers, websites, organisations, reference lists and other sources searched or consulted to identify studies. Specify the date when each source was last searched or consulted.	3
Search strategy	7	Present the full search strategies for all databases, registers and websites, including any filters and limits used.	Appendix 1
Selection process	8	Specify the methods used to decide whether a study met the inclusion criteria of the review, including how many reviewers screened each record and each report retrieved, whether they worked independently, and if applicable, details of automation tools used in the process.	3
Data collection process	9	Specify the methods used to collect data from reports, including how many reviewers collected data from each report, whether they worked independently, any processes for obtaining or confirming data from study investigators, and if applicable, details of automation tools used in the process.	4
Data items	10a	List and define all outcomes for which data were sought. Specify whether all results that were compatible with each outcome domain in each study were sought (e.g. for all measures, time points, analyses), and if not, the methods used to decide which results to collect.	2, 3
	10b	List and define all other variables for which data were sought (e.g. participant and intervention characteristics, funding sources). Describe any assumptions made about any missing or unclear information.	4
Study risk of bias assessment	11	Specify the methods used to assess risk of bias in the included studies, including details of the tool(s) used, how many reviewers assessed each study and whether they worked independently, and if applicable, details of automation tools used in the process.	4
Effect measures	12	Specify for each outcome the effect measure(s) (e.g. risk ratio, mean difference) used in the synthesis or presentation of results.	4
Synthesis methods	13a	Describe the processes used to decide which studies were eligible for each synthesis (e.g. tabulating the study intervention characteristics and comparing against the planned groups for each synthesis (item #5)).	4
	13b	Describe any methods required to prepare the data for presentation or synthesis, such as handling of missing summary statistics, or data conversions.	4
	13c	Describe any methods used to tabulate or visually display results of individual studies and syntheses.	4
	13d	Describe any methods used to synthesize results and provide a rationale for the choice(s). If meta-analysis was performed, describe the model(s), method(s) to identify the presence and extent of statistical heterogeneity, and software package(s) used.	4

1Section and Topic	Item #	Checklist item	Location where item is reported
	13e	Describe any methods used to explore possible causes of heterogeneity among study results (e.g. subgroup analysis, meta-regression).	4
	13f	Describe any sensitivity analyses conducted to assess robustness of the synthesized results.	4
Reporting bias assessment	14	Describe any methods used to assess risk of bias due to missing results in a synthesis (arising from reporting biases).	4
Certainty assessment	15	Describe any methods used to assess certainty (or confidence) in the body of evidence for an outcome.	4
**RESULTS**			
Study selection	16a	Describe the results of the search and selection process, from the number of records identified in the search to the number of studies included in the review, ideally using a flow diagram.	5, Figure 1
	16b	Cite studies that might appear to meet the inclusion criteria, but which were excluded, and explain why they were excluded.	Appendix 2
Study characteristics	17	Cite each included study and present its characteristics.	5-6, Appendix 3
Risk of bias in studies	18	Present assessments of risk of bias for each included study.	Appendix 4
Results of individual studies	19	For all outcomes, present, for each study: (a) summary statistics for each group (where appropriate) and (b) an effect estimate and its precision (e.g. confidence/credible interval), ideally using structured tables or plots.	Appendix 5
Results of syntheses	20a	For each synthesis, briefly summarise the characteristics and risk of bias among contributing studies.	7-12
	20b	Present results of all statistical syntheses conducted. If meta-analysis was done, present for each the summary estimate and its precision (e.g. confidence/credible interval) and measures of statistical heterogeneity. If comparing groups, describe the direction of the effect.	7-12
	20c	Present results of all investigations of possible causes of heterogeneity among study results.	Appendix 7
	20d	Present results of all sensitivity analyses conducted to assess the robustness of the synthesized results.	Appendix 8
Reporting biases	21	Present assessments of risk of bias due to missing results (arising from reporting biases) for each synthesis assessed.	NA
Certainty of evidence	22	Present assessments of certainty (or confidence) in the body of evidence for each outcome assessed.	Appendix 9
**DISCUSSION**			
Discussion	23a	Provide a general interpretation of the results in the context of other evidence.	12-14
	23b	Discuss any limitations of the evidence included in the review.	14-15
	23c	Discuss any limitations of the review processes used.	15
	23d	Discuss implications of the results for practice, policy, and future research.	15
**OTHER INFORMATION**			
Registration and protocol	24a	Provide registration information for the review, including register name and registration number, or state that the review was not registered.	2
	24b	Indicate where the review protocol can be accessed, or state that a protocol was not prepared.	2

1Section and Topic	Item #	Checklist item	Location where item is reported
	24c	Describe and explain any amendments to information provided at registration or in the protocol.	NA
Support	25	Describe sources of financial or non-financial support for the review, and the role of the funders or sponsors in the review.	17
Competing interests	26	Declare any competing interests of review authors.	17
Availability of data, code and other materials	27	Report which of the following are publicly available and where they can be found: template data collection forms; data extracted from included studies; data used for all analyses; analytic code; any other materials used in the review.	17

From: Page MJ, McKenzie JE, Bossuyt PM, Boutron I, Hoffmann TC, Mulrow CD, et al. The PRISMA 2020 statement: an updated guideline for reporting systematic reviews. BMJ 2021;372:n71. doi: 10.1136/bmj.n71

For more information, visit: http://www.prisma-statement.org/
